# Incidence, mortality, and DALYs of global pharyngeal cancer: systematic analysis and projections Based on global burden of disease study 2021

**DOI:** 10.1080/07853890.2025.2547092

**Published:** 2025-08-19

**Authors:** Tianjiao Zhou, Ying Shen, Enhui Zhou, Jingyu Zhang, Xiaoting Wang, Fan Song, Lili Xiao, Chen Xu, Wen Lu, Haibo Ye, Kaiming Su, Hongliang Yi, Weijun Huang

**Affiliations:** a Department of Otorhinolaryngology Head and Neck Surgery, Shanghai Sixth People’s Hospital, Affiliated to Shanghai Jiao Tong University School of Medicine, Shanghai, China; b Shanghai Key Laboratory of Sleep Disordered Breathing, Shanghai, China; c Otolaryngology Institute of Shanghai Jiao Tong University, Shanghai, China; d Department of Nursing, Shanghai Sixth People’s Hospital Affiliated to Shanghai Jiao Tong, University School of Medicine, Shanghai, China; e Department of Otolaryngology, Shanghai Tenth People’s Hospital, School of Medicine, Tongji University, Shanghai, China

**Keywords:** Global burden of disease study, pharyngeal cancer, incidence, death, disability-adjusted life-years

## Abstract

**Introduction:**

Compared to other head and neck cancers, pharyngeal cancer (PC) has poorer survival, representing a significant health burden. This study aimed to assess the burden and trends of PC at global, regional, and national levels and analyze mortality-related factors.

**Methods:**

Data on PC, including incidence, mortality, disability-adjusted life-years (DALYs), and death-related risk factors from 1990 to 2021, were obtained from the Global Burden of Disease Study 2021. Estimated annual percentage changes (EAPCs) were calculated to assess trends.

**Results:**

In 2021, PC incidence was 169,820, with 98,435 deaths and 2,843,781 DALYs. Age-standardized rates for incidence, death, and DALYs were 1.93, 1.13, and 32.30 per 100,000, respectively. South Asia had the highest death and DALYs rates (3.23 and 93.00). Low-middle socio-demographic index (SDI) regions showed the highest death rate (2.19) and the greatest EAPC for death rates (0.684%). A positive correlation between SDI and death rates was observed globally (*R* = 0.26, *p* < 0.05), particularly in males (*R* = 0.3, *p* < 0.05), but not in females. Males exhibited a trend toward younger ages at death by aclohol, peaking in the 35–39-year group.

**Conclusion:**

In 2021, global PC incidence, deaths, and DALYs increased significantly, with notable regional disparities, especially in low-middle SDI regions. Alcohol-related mortality disproportionately affected younger males. Strengthening oral health resources, controlling alcohol and tobacco use are essential to reducing the global PC burden.

## Introduction

Common sites of head and neck cancers include the oral cavity, sinonasal cavity, pharynx, and larynx. We excluded nasopharyngeal carcinoma due to its distinct EBV-driven etiology [1], which fundamentally differs from the HPV/tobacco-dominated pathogenesis of other pharyngeal cancers [2,3]. Excluding nasopharyngeal cancers, other pharyngeal cancers (PC) mainly include tonsillar, oropharyngeal, and hypopharyngeal cancers [4,5]. In 2019, the incidence of total PC was 167,000 (95% uncertainty interval [UI], 153,000–180,000), with an estimated 114,000 (95% UI, 103,000–126,000) deaths [5]. Although the 5-year survival rate for head and neck cancer has significantly improved in recent years, the survival rate for hypopharyngeal cancer remains notably low at only 30% [6]. Most PCs severely impair patients’ daily physiological functions such as swallowing, affect their psychological well-being and social interactions, and contribute to the overall healthcare burden [7]. Independent risk factors for PC include tobacco, alcohol, and human papillomavirus (HPV) infection, with varying global lifestyle habits contributing to differences in the global burden of PC. Real-time updates on pharyngeal cancer burden data are essential for informing healthcare policies such as National Cancer Control Plans (NCCPs) and the ‘3 by 35 Initiative’ to improve cancer diagnosis and treatment.

Currently, epidemiological studies on PC are constrained by limitations in both spatial and temporal dimensions, frequently relying on cross-sectional or regional data that are prone to significant biases and methodological constraints [8–10]. The Global Burden of Disease (GBD) Study is a collaborative effort involving over 12,000 scientists from more than 160 countries that has provided timely, effective, and relevant assessments of key health outcomes for over 30 years [11]. The GBD study offers higher spatial resolution and more detailed age groups than any other study [12]. This enhances the estimation and description of epidemiological processes and improves our understanding of their impact on population health and reforms [13,14]. In addition to serving as the most recent objective data for assessing the PC burden, the updated PC data in the 2021 GBD can provide a foundation for a better understanding of the changes before and after the corona virus disease 2019 (COVID-19) pandemic. However, to the best of our knowledge, there is currently no study on PC that uses the 2021 GBD database. This study aimed to utilize the 2021 GBD study to analyze the incidence, deaths, and disability-adjusted life years (DALYs) of PC globally, stratified by sociodemographic index (SDI), age, and sex. Additionally, we assessed the trends and death-related risk factors from 1990 to 2021.

## Methods

### Data source

Data relevant to the keywords were retrieved and downloaded from the GBD Results Tool on the GBD database website (https://ghdx.healthdata.org/gbd-2021). Other PCs were defined according to the International Classification of Diseases, Tenth Revision (ICD-10), encompassing tonsil cancer (C09), oropharyngeal cancer (C10), piriform sinus cancer (C12), and hypopharyngeal cancer (C13), but excluding cancers of the base of tongue (C01), soft palate (C05.1), and uvula (C05.2). In this analysis, the term “pharyngeal cancer” (synonymous with “other pharynx cancers” in the GBD-2021 study) was used, as nasopharyngeal cancer was analyzed separately. Nasopharyngeal cancer was excluded from this study due to its distinct epidemiological profile compared to cancers occurring in other pharyngeal sites. We acquired specific data on the incidence, deaths, and DALYs of patients with PC. The estimates are presented by sex, age, and locations for the years 1990 to 2021. The Ethics Committee of the Sixth People’s Hospital of Shanghai determined that the study did not need approval because it used publicly available data (No: MS-2024-001).

### Estimates groups

For age group, we categorized the age groups into 5-year increments from 0 to 95 years (<5, 5–9, 10–14, 15–19, …, 90–94, ≥95 years). For location groups, we used three geographic classifications: GBD super-regions, GBD world regions, and individual countries or territories. According to the SDI levels defined by the GBD study, regions were categorized into five SDI levels: Low SDI (≤ 0.4658), Low-middle SDI (0.4658–0.6188), Middle SDI (0.6188–0.7120), High-middle SDI (0.7120–0.8103), and High SDI (> 0.8103). The GBD-2021 comparative risk assessment framework was used to estimate the proportion of deaths attributable to specific risk factors, such as alcohol consumption, due to PC [5].

### Burden quantification indicators

We defined the older-adult ratio as the ratio of disease burden in individuals aged 65 years and older to that in individuals younger than 65 years [15]. A higher ratio suggests that population aging may have a more substantial influence on the incidence or mortality rate of PC. Age-standardized rates (ASR) were calculated per 100,000 population. We analyzed trends using a linear regression model with the natural logarithm of the ASR as the dependent variable and calendar year as the independent variable: *y* = *α* + *βx* + *ε*. where *y* = In(ASR) and *x* = calendar year. The EAPC was calculated from the *β* coefficient using the formula: EAPC = 100 × (exp(*β*)−1) [16]. An EAPC value greater than zero indicates an increasing trend in age-standardized indicators, while a value less than zero suggests a decreasing trend. Frontier analysis was employed to determine the lowest achievable ASIR, ASDR, and age-standardized DALYs rate, as defined by the SDI [17]. Additionally, the Slope Index of Inequality (SII) and Concentration Index were used to quantify cross-country inequalities in PC burden, measuring absolute and relative gradient inequality, respectively. The GBD comparative risk assessment (CRA) framework was employed to evaluate the exposure to risk factors associated with pharyngeal cancer and their consequent disease burden [18]. The CRA consists of seven major interrelated methodological components. The effect size was initially estimated by quantifying the relative risk (RR) of specified health outcomes associated with exposure to identified risk factors. By considering the exposure distributions across different ages, genders, locations, and years, as well as the RR associated with each exposure level, the population attributable fractions (PAFs) for Deaths were calculated for each risk factor [19]. For missing data imputation, we adopted the standardized analytical approaches well-documented in previous GBD methodological publications [20].

### BAPC model projections from 2022 to 2050

This study used the Bayesian Age-Period-Cohort (BAPC) model to predict future disease burdens, leveraging its ability to handle complex, high-dimensional data from large-scale epidemiological studies like the GBD-2021 [21]. The model integrates age, period, and cohort effects dynamically within a Bayesian framework, improving prediction accuracy through smoothing techniques. It employs the Integrated Nested Laplace Approximation (INLA) method for efficient computation, avoiding challenges of traditional Markov Chain Monte Carlo approaches. Using the “BAPC” R package, GBD-2021 data, and IHME demographic projections, the study forecasts future burdens by accounting for the interplay of age, period, and cohort effects.

### Statistical analysis and visualization

Data on incidence, deaths, DALYs, ASIR, ASDR, and age-standardized DALYs rates were presented with 95% uncertainty intervals (UIs). EAPCs were reported with 95% confidence intervals (CIs). Values were displayed in bar charts and categorized by age, location, and SDI subgroups. Annual trends were visualized using line graphs, and regional variations were depicted through heat maps. Fitted curves were used to illustrate relationships and annual trends between SDI and disease burden indicators. Trends were classified as “increasing” or “decreasing” if the slope was statistically significant; otherwise, they were labeled as “stable”. Missing values were processed in accordance with established GBD study protocols [14,21]. All statistical analyses were conducted using R software (version 4.3.2; R Foundation, Vienna, Austria), with statistical significance set at *p* < 0.05 (two-sided).

## Results

### Overall global burden

**Figure 1.**
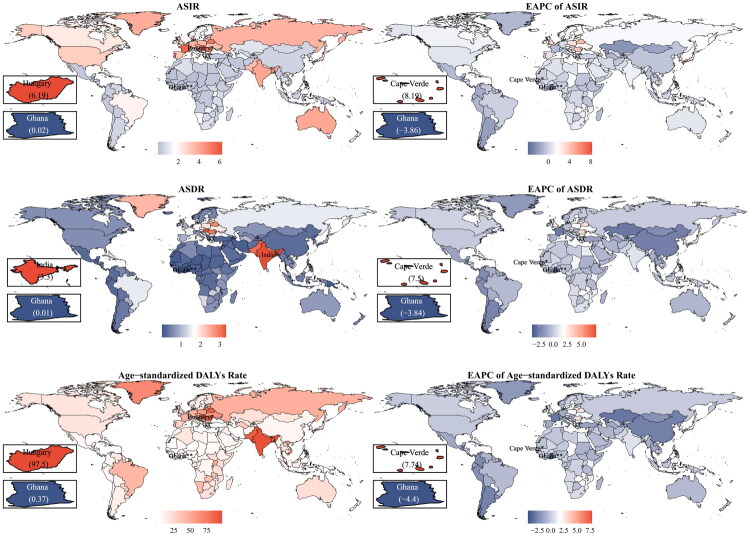
Age-standardized disease burden and estimated annual percentage change of pharyngeal cancer across 204 countries and territories from 1990 to 2021. ASIR, age-standardized incidence rate; ASDR, age-standardized death rate; DALYs, disability-adjusted life years; EAPC, estimated annual percentage change

In 2021, the incidence of PC was 169,820 cases (95% UI: 159,847–179,704), with 98,435 (91,567–105,485) deaths and 2,843,781 (2,622,259–3,063,043) DALYs. The ASIR was 1.93 (1.82–2.05), the ASDR was 1.13 (1.05–1.21), and the age-standardized DALYs rate was 32.30 (29.92–35.17). From 1990 to 2021, the EAPC for ASIR of PC was 0.68 (95% CI: 0.60–0.75), the ASDR was 0.06 (−0.02–0.14), and the age-standardized DALYs rate was −0.09 (−0.17–0.01) (Table 1). The region with the highest ASIR was Central Europe at 3.94 (3.57–4.34), while the lowest was Oceania at 0.15 (0.12–0.19). Both the ASDR and age-standardized DALY rates were highest in South Asia, at 3.23 (2.84–3.62) and 93.00 (81.36–104.74), respectively. The highest EAPCs for ASIR, ASDR, and age-standardized DALYs rate were found in the high-income Asia Pacific region, at 3.07 (2.66–3.48), 2.03 (1.73–2.34), and 1.61 (1.26–1.96), respectively. The country with the ­highest ASIR was Hungary, at 6.19 (5.01–7.39), while the highest ASDR was observed in India, at 3.30 (2.87–3.74) (Figure 1, Supplementary Table 1, 2).

**Table 1.** Global burden and trends of pharyngeal cancer from 1990 to 2021 by regions.

Characteristics	1990		2021			1990		2021			1990		2021		
	Incidence cases	ASIR	Incidence cases	ASIR	EAPC	Death cases	ASDR	Death cases	ASDR	EAPC	DALYs cases	Age_standardised DALYs Rate	DALYs cases	Age_standardised DALYs	EAPC
	(95%UI)	per 100,000 (95%UI)	(95%UI)	per 100,000 (95%UI)	(95%CI)	(95%UI)	per 100,000 (95%UI)	(95%UI)	per 100,000 (95%UI)	(95%CI)	(95%UI)	per 100,000 (95%UI)	(95%UI)	per 100,000 (95%UI)	(95%CI)
Global	64529 (60829 − 68920)	1.552 (1.463 − 1.657)	169820 (159847 − 179704)	1.933 (1.818 − 2.047)	0.677 (0.603–0.751)	44344 (40994 − 48190)	1.082 (1.001 − 1.176)	98435 (91567 − 105485)	1.127 (1.048 − 1.207)	0.061 (–0.018–0.141)	1372478 (1270671 − 1494393)	32.302 (29.919 − 35.17)	2843781 (2622259 − 3063043)	32.377 (29.854 − 34.87)	−0.087 (–0.165to–0.009)
Gender															
Female	12012 (10617 − 13743)	0.556 (0.493 − 0.635)	32753 (28867 − 39597)	0.72 (0.635 − 0.871)	0.869 (0.791–0.947)	8173 (6970 − 9702)	0.382 (0.327 − 0.452)	17998 (15452 − 22431)	0.394 (0.339 − 0.491)	0.092 (0.027–0.158)	243179 (203246 − 292981)	11.058 (9.281 − 13.332)	509884 (440655 − 635095)	11.379 (9.796 − 14.188)	0.072 (0.003–0.142)
Male	52517 (49128 − 56661)	2.641 (2.47 − 2.851)	137066 (128148 − 146458)	3.257 (3.047 − 3.478)	0.636 (0.561–0.712)	36171 (33086 − 39869)	1.867 (1.71 − 2.053)	80437 (73959 − 87131)	1.939 (1.786 − 2.099)	0.046 (–0.038–0.131)	1129299 (1033227 − 1247782)	54.845 (50.204 − 60.593)	2333897 (2128450 − 2539518)	54.775 (50.003 − 59.57)	−0.108 (–0.189to–0.026)
SDI regions															
Low SDI	3122 (2453 − 3935)	1.251 (0.982 − 1.573)	8451 (6898 − 10299)	1.534 (1.258 − 1.863)	0.735 (0.621–0.848)	2903 (2274 − 3672)	1.196 (0.938 − 1.507)	7290 (5962 − 8895)	1.374 (1.127 − 1.658)	0.554 (0.465–0.644)	92542 (72604 − 117034)	35.052 (27.452 − 44.476)	224509 (182350 − 273523)	38.197 (31.135 − 46.735)	0.338 (0.26–0.416)
Low-middle SDI	12941 (10742 − 15398)	1.93 (1.602 − 2.296)	39181 (34333 − 43831)	2.553 (2.245 − 2.854)	0.989 (0.918–1.06)	11901 (9868 − 14192)	1.82 (1.511 − 2.168)	32744 (28803 − 36751)	2.186 (1.929 − 2.457)	0.684 (0.638–0.729)	378985 (315257 − 452015)	53.717 (44.496 − 64.062)	992079 (865349 − 1116934)	62.445 (54.645 − 70.133)	0.582 (0.535–0.628)
Middle SDI	11484 (10543 − 12384)	1.03 (0.946 − 1.113)	36177 (32433 − 39860)	1.279 (1.146 − 1.407)	0.513 (0.372–0.655)	9925 (9110 − 10724)	0.92 (0.848 − 0.994)	25243 (22497 − 27817)	0.909 (0.811 − 1.001)	−0.225 (–0.325to–0.126)	309950 (285027 − 334698)	26.341 (24.209 − 28.439)	740167 (659682 − 815220)	25.794 (23.012 − 28.389)	−0.269 (–0.372to–0.166)
High-middle SDI	13990 (13310 − 14842)	1.35 (1.284 − 1.43)	30094 (27807 − 32443)	1.525 (1.41 − 1.644)	0.198 (0.055–0.341)	9395 (8873 − 10015)	0.917 (0.866 − 0.978)	14512 (13403 − 15596)	0.729 (0.673 − 0.783)	−1.012 (–1.138to–0.886)	290231 (274239 − 309030)	27.796 (26.26 − 29.61)	415671 (383316 − 448188)	21.201 (19.557 − 22.83)	−1.183 (–1.311to–1.054)
High SDI	22912 (22113 − 23678)	2.185 (2.107 − 2.261)	55742 (52690 − 58417)	2.97 (2.821 − 3.115)	1.054 (0.978–1.131)	10162 (9765 − 10550)	0.956 (0.918 − 0.994)	18547 (17371 − 19569)	0.931 (0.875 − 0.981)	−0.096 (–0.198–0.005)	299016 (286772 − 311110)	28.939 (27.733 − 30.158)	468574 (441016 − 495807)	25.542 (24.006 − 27.046)	−0.449 (–0.538to–0.36)
GBD regions															
North Africa and Middle East	558 (479 − 653)	0.315 (0.272 − 0.371)	1777 (1496 − 2114)	0.363 (0.307 − 0.431)	0.541 (0.513–0.569)	471 (403 − 551)	0.28 (0.241 − 0.329)	1116 (941 − 1327)	0.245 (0.208 − 0.29)	−0.364 (–0.387to–0.342)	14241 (12171 − 16690)	7.477 (6.405 − 8.771)	32990 (27658 − 39706)	6.407 (5.387 − 7.646)	−0.443 (–0.473to–0.413)
South Asia	18673 (15722 − 22060)	2.905 (2.442 − 3.44)	61370 (53891 − 69004)	3.891 (3.423 − 4.369)	0.955 (0.892–1.018)	17017 (14299 − 20170)	2.722 (2.286 − 3.232)	49844 (43790 − 56082)	3.234 (2.844 − 3.625)	0.579 (0.542–0.615)	547876 (463107 − 646548)	80.638 (67.806 − 95.505)	1511059 (1320727 − 1703791)	92.995 (81.355 − 104.742)	0.483 (0.448–0.518)
Central Asia	496 (454 − 545)	0.997 (0.911 − 1.101)	688 (598 − 789)	0.774 (0.674 − 0.884)	−0.929 (–1.136to–0.723)	412 (376 − 456)	0.844 (0.769 − 0.94)	516 (449 − 595)	0.601 (0.525 − 0.689)	−1.223 (–1.429to–1.017)	12827 (11764 − 14069)	25.039 (22.948 − 27.465)	15698 (13556 − 18181)	16.998 (14.723 − 19.63)	−1.435 (–1.639to–1.23)
Central Europe	3054 (2907 − 3216)	2.048 (1.952 − 2.156)	7657 (6929 − 8425)	3.942 (3.568 − 4.34)	2.098 (1.976–2.219)	2233 (2123 − 2353)	1.492 (1.421 − 1.569)	4253 (3842 − 4672)	2.114 (1.906 − 2.326)	1.106 (1.029–1.183)	70996 (67529 − 74888)	47.897 (45.622 − 50.524)	121156 (108898 − 133611)	64.152 (57.755 − 70.774)	0.866 (0.754–0.977)
Eastern Europe	5805 (5445 − 6354)	2.045 (1.921 − 2.238)	11942 (10704 − 13241)	3.665 (3.302 − 4.066)	1.708 (1.433–1.983)	3624 (3390 − 3992)	1.267 (1.186 − 1.393)	5661 (5042 − 6384)	1.671 (1.491 − 1.881)	0.587 (0.409–0.765)	114946 (107427 − 127082)	40.598 (37.983 − 44.914)	171422 (152421 − 193609)	52.756 (46.888 − 59.51)	0.495 (0.304–0.686)
Australasia	637 (578 − 698)	2.787 (2.53 − 3.054)	1716 (1471 − 1969)	3.558 (3.057 − 4.093)	0.775 (0.442–1.109)	193 (177 − 211)	0.834 (0.762 − 0.913)	356 (305 − 411)	0.69 (0.593 − 0.793)	−0.694 (–1.096to–0.29)	5348 (4893 − 5859)	23.578 (21.533 − 25.744)	9269 (7989 − 10633)	19.412 (16.755 − 22.195)	−0.663 (–1.049to–0.277)
High-income Asia Pacific	1890 (1808 − 1965)	0.906 (0.868 − 0.943)	9709 (8927 − 10342)	2.347 (2.179 − 2.501)	3.068 (2.656–3.482)	880 (847 − 909)	0.429 (0.412 − 0.444)	3804 (3476 − 4039)	0.818 (0.757 − 0.868)	2.034 (1.727–2.342)	24214 (23381 − 25062)	11.557 (11.156 − 11.963)	78591 (72884 − 83220)	19.466 (18.139 − 20.612)	1.609 (1.26–1.959)
High-income North America	7929 (7687 − 8125)	2.452 (2.384 − 2.513)	17707 (16870 − 18363)	2.896 (2.775 − 3.001)	0.727 (0.569–0.885)	2230 (2136 − 2291)	0.664 (0.639 − 0.682)	3992 (3788 − 4166)	0.62 (0.589 − 0.646)	−0.058 (–0.319–0.204)	61730 (59597 − 63620)	19.255 (18.644 − 19.848)	104570 (100011 − 109206)	17.286 (16.561 − 18.027)	−0.156 (–0.401–0.09)
Southern Latin America	586 (521 − 656)	1.256 (1.116 − 1.41)	556 (476 − 645)	0.658 (0.562 − 0.766)	−1.727 (–2.11to–1.341)	449 (403 − 501)	0.97 (0.867 − 1.084)	339 (289 − 394)	0.394 (0.337 − 0.458)	−2.474 (–2.868to–2.078)	12972 (11507 − 14563)	27.742 (24.592 − 31.226)	8903 (7584 − 10417)	10.657 (9.05 − 12.502)	−2.692 (–3.079to–2.303)
Western Europe	13384 (12655 − 14112)	2.626 (2.481 − 2.77)	25581 (23411 − 27861)	3.304 (3.034 − 3.595)	0.595 (0.438–0.752)	7005 (6633 − 7344)	1.335 (1.262 − 1.401)	9189 (8366 − 10079)	1.1 (1.002 − 1.205)	−0.829 (–0.966to–0.692)	210860 (199251 − 221802)	42.285 (39.862 − 44.561)	237066 (216157 − 261058)	31.182 (28.54 − 34.313)	−1.243 (–1.382to–1.105)
Andean Latin America	88 (77 − 100)	0.415 (0.363 − 0.475)	194 (153 − 245)	0.323 (0.256 − 0.407)	−0.925 (–1.336to–0.511)	80 (70 − 91)	0.391 (0.343 − 0.446)	141 (113 − 175)	0.24 (0.192 − 0.296)	−1.683 (–2.062to–1.304)	2293 (2009 − 2609)	10.261 (9.011 − 11.715)	3726 (2957 − 4667)	6.106 (4.844 − 7.632)	−1.819 (–2.2to–1.437)
Caribbean	451 (419 − 486)	1.736 (1.611 − 1.87)	817 (697 − 943)	1.507 (1.287 − 1.741)	−0.201 (–0.578–0.178)	364 (337 − 394)	1.427 (1.322 − 1.545)	580 (497 − 675)	1.071 (0.916 − 1.246)	−0.659 (–1.038to–0.278)	9725 (8981 − 10623)	36.708 (33.928 − 40.106)	15411 (13026 − 18179)	28.525 (24.095 − 33.625)	−0.553 (–0.929to–0.175)
Central Latin America	399 (382 − 417)	0.477 (0.455 − 0.498)	1040 (921 − 1179)	0.411 (0.364 − 0.466)	−0.742 (–0.961to–0.522)	353 (337 − 368)	0.438 (0.417 − 0.456)	775 (687 − 875)	0.312 (0.277 − 0.351)	−1.325 (–1.544to–1.106)	9643 (9242 − 10074)	10.808 (10.343 − 11.286)	19868 (17530 − 22607)	7.729 (6.83 − 8.782)	−1.32 (–1.547to–1.093)
Tropical Latin America	1905 (1809 − 2003)	1.949 (1.844 − 2.052)	5290 (4921 − 5634)	2.001 (1.861 − 2.132)	−0.015 (–0.155–0.126)	1608 (1525 − 1695)	1.697 (1.602 − 1.79)	3829 (3547 − 4080)	1.46 (1.351 − 1.555)	−0.539 (–0.678to–0.399)	49850 (47335 − 52442)	48.762 (46.324 − 51.357)	111176 (103800 − 118474)	41.79 (38.996 − 44.5)	−0.609 (–0.774to–0.443)
East Asia	5448 (4517 − 6554)	0.598 (0.5 − 0.716)	14279 (11679 − 17537)	0.638 (0.523 − 0.779)	−0.033 (–0.389–0.323)	4570 (3780 − 5498)	0.522 (0.437 − 0.626)	6889 (5625 − 8433)	0.314 (0.258 − 0.382)	−2.086 (–2.418to–1.752)	136914 (112557 − 166109)	14.242 (11.761 − 17.213)	185965 (150251 − 229687)	8.327 (6.778 − 10.207)	−2.17 (–2.513to–1.826)
Oceania	5 (4 − 7)	0.154 (0.121 − 0.199)	13 (10 − 16)	0.152 (0.12 − 0.191)	0.147 (0.048–0.245)	4 (3 − 6)	0.142 (0.112 − 0.185)	11 (9 − 14)	0.137 (0.108 − 0.173)	0.088 (–0.016–0.192)	150 (115 − 200)	4.063 (3.169 − 5.373)	368 (282 − 472)	3.903 (3.03 − 4.977)	0.046 (–0.054–0.146)
Southeast Asia	2226 (1868 − 2620)	0.825 (0.699 − 0.971)	6915 (5765 − 8225)	0.985 (0.825 − 1.162)	0.462 (0.354–0.571)	1942 (1634 − 2283)	0.747 (0.635 − 0.879)	4956 (4149 − 5853)	0.734 (0.618 − 0.862)	−0.175 (–0.253to–0.096)	58567 (49243 − 68732)	20.422 (17.156 − 23.983)	144945 (120816 − 172263)	19.961 (16.635 − 23.669)	−0.184 (–0.27to–0.097)
Central Sub-Saharan Africa	77 (58 − 98)	0.311 (0.239 − 0.393)	204 (149 − 270)	0.33 (0.24 − 0.437)	0.31 (0.101–0.519)	72 (54 − 92)	0.305 (0.235 − 0.383)	181 (131 − 242)	0.308 (0.224 − 0.414)	0.169 (–0.013–0.351)	2268 (1693 − 2925)	8.538 (6.483 − 10.87)	5820 (4199 − 7837)	8.565 (6.208 − 11.454)	0.145 (–0.037–0.328)
Eastern Sub-Saharan Africa	572 (438 − 685)	0.691 (0.534 − 0.826)	1448 (1031 − 1921)	0.744 (0.532 − 0.981)	0.167 (0.132–0.202)	531 (407 − 636)	0.66 (0.512 − 0.789)	1251 (896 − 1671)	0.669 (0.482 − 0.884)	−0.007 (–0.03–0.017)	17017 (12927 − 20414)	19.342 (14.807 − 23.214)	41282 (29151 − 55546)	19.666 (14.047 − 26.302)	−0.012 (–0.039–0.015)
Southern Sub-Saharan Africa	183 (155 − 222)	0.625 (0.527 − 0.762)	522 (446 − 598)	0.824 (0.705 − 0.941)	0.989 (0.847–1.132)	156 (132 − 190)	0.546 (0.461 − 0.669)	413 (354 − 473)	0.67 (0.575 − 0.765)	0.754 (0.549–0.959)	5047 (4298 − 6092)	16.428 (13.966 − 19.978)	13029 (11051 − 15044)	19.793 (16.871 − 22.807)	0.684 (0.479–0.889)
Western Sub-Saharan Africa	164 (130 − 202)	0.168 (0.133 − 0.207)	394 (305 − 497)	0.168 (0.133 − 0.21)	0.011 (–0.072–0.093)	151 (120 − 186)	0.158 (0.126 − 0.195)	339 (265 − 425)	0.151 (0.121 − 0.188)	−0.124 (–0.2to–0.048)	4997 (3945 − 6220)	4.882 (3.853 − 6.062)	11465 (8778 − 14599)	4.53 (3.523 − 5.704)	−0.236 (–0.32to–0.153)

### Incidence and trends of PC

**Figure 2.**
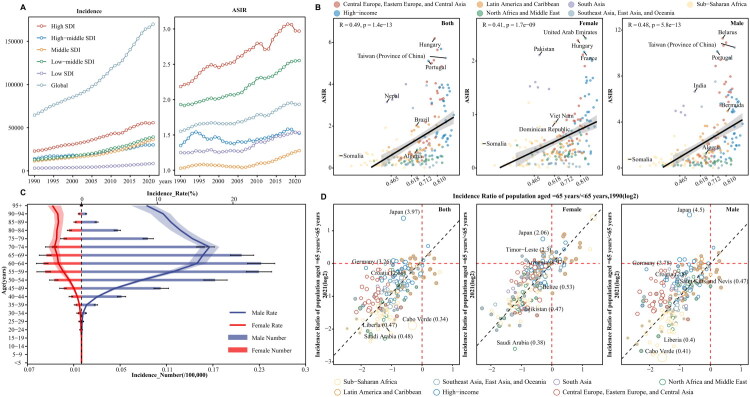
Incidence burden and trends of pharyngeal cancer from 1990 to 2021 across different SDI regions, age groups, and genders. **A**: Incidence numbers and ASIR of pharyngeal cancer globally and across 5 SDI level regions; **B**: Correlation between the ASIR of pharyngeal cancer and SDI index across 204 countries and territories globally by gender. R, the correlation coefficient; **C**: Incidence burden of pharyngeal cancer across different age groups (5 year intervals) by gender in 2021; **D**: Age-related incidence ratio of pharyngeal cancer between 2000 and 2021 in 204 countries and territories. Points filled by grey color represent an annual change less than 0. Different colors indicate 7 GBD super-regions. Point size represents the absolute annual change of the age-related incidence rate from 2000 to 2021. ASIR, age-standardized incidence rate.

In 2021, the incidence of PC was highest in males, with 137,066 (128,148–146,458) cases, compared to 32,753 (28,867–39,597) cases in females. However, from 1990 to 2021, the EAPC for male ASIR was 0.64 (0.56–0.71), which was lower than females with EAPC at 0.87 (0.79–0.95) (Table 2). In 2021, high SDI regions had the highest ASIR of PC at 2.97 (2.82–3.12), while middle SDI regions had the lowest at 1.28 (1.15–1.41) (Table 1, Figure 2A)**.** A positive correlation between SDI and ASIR was observed across 204 countries in 2021 (*R* = 0.49, *p* < 0.05), with similar patterns evident in both males (*R* = 0.48, *p* < 0.05) and females (*R* = 0.41, *p* < 0.05) (Figure 2B). In 2021, the global incidence rate of PC was highest among individuals aged 70–74 years, at 9.81 (9.22–10.47) (Supplementary Figure 1A), with a consistent annual increase from 1990 to 2021 (Supplementary Figure 2A). The highest incidence rate was 16.9 (15.79–18.2) for males aged 70–74 years and 3.83 (2.72–4.49) for females aged 95 years and older. Males aged 70–74 years showed an increased incidence between 1990 and 2021 (Figure 2C). In Japan, the age-related incidence rate in 2021 was 3.97-times higher than that in 1990, whereas in Cabo Verde, this ratio was the lowest at 0.34-times that in 1990 (Figure 2D).

**Table 2.** Global burden and trends of pharyngeal cancer from 1990 to 2021 by regions and gender.

Characteristics	Gender	1990		2021			1990		2021			1990		2021		
		Incidence cases	ASIR	Incidence cases	ASIR	EAPC	Death cases	ASDR	Death cases	ASDR	EAPC	DALYs cases	Age_standardised DALYs Rate	DALYs cases	Age_standardised DALYs	EAPC
		(95%UI)	per 100,000 (95%UI)	(95%UI)	per 100,000 (95%UI)	(95%CI)	(95%UI)	per 100,000 (95%UI)	(95%UI)	per 100,000 (95%UI)	(95%CI)	(95%UI)	per 100,000 (95%UI)	(95%UI)	per 100,000 (95%UI)	(95%CI)
Global	Female	12012 (10617 − 13743)	0.556 (0.493 − 0.635)	32753 (28867 − 39597)	0.72 (0.635 − 0.871)	0.869 (0.791–0.947)	8173 (6970 − 9702)	0.382 (0.327 − 0.452)	17998 (15452 − 22431)	0.394 (0.339 − 0.491)	0.092 (0.027–0.158)	243179 (203246 − 292981)	11.058 (9.281 − 13.332)	509884 (440655 − 635095)	11.379 (9.796 − 14.188)	0.072 (0.003–0.142)
	Male	52517 (49128 − 56661)	2.641 (2.47 − 2.851)	137066 (128148 − 146458)	3.257 (3.047 − 3.478)	0.636 (0.561–0.712)	36171 (33086 − 39869)	1.867 (1.71 − 2.053)	80437 (73959 − 87131)	1.939 (1.786 − 2.099)	0.046 (–0.038–0.131)	1129299 (1033227 − 1247782)	54.845 (50.204 − 60.593)	2333897 (2128450 − 2539518)	54.775 (50.003 − 59.57)	−0.108 (–0.189to–0.026)
SDI regions																
Low SDI	Female	861 (596 − 1144)	0.705 (0.498 − 0.922)	2218 (1631 − 2915)	0.782 (0.573 − 1.026)	0.386 (0.299–0.473)	777 (545 − 1034)	0.659 (0.473 − 0.859)	1810 (1328 − 2373)	0.668 (0.492 − 0.878)	0.123 (0.066–0.181)	25115 (17070 − 33975)	19.137 (13.306 − 25.491)	57054 (42330 − 74847)	18.599 (13.667 − 24.367)	−0.034 (–0.086–0.018)
	Male	2262 (1696 − 3086)	1.773 (1.327 − 2.406)	6233 (4965 − 8006)	2.314 (1.831 − 2.951)	0.948 (0.824–1.072)	2126 (1593 − 2902)	1.71 (1.277 − 2.325)	5480 (4355 − 7013)	2.111 (1.678 − 2.687)	0.798 (0.694–0.901)	67427 (50446 − 92180)	50.211 (37.551 − 68.556)	167455 (133061 − 215414)	58.329 (46.315 − 74.753)	0.547 (0.458–0.635)
Low-middle SDI	Female	2999 (2324 − 4030)	0.911 (0.707 − 1.215)	8269 (6829 − 10926)	1.045 (0.867 − 1.388)	0.481 (0.414–0.549)	2665 (2070 − 3565)	0.836 (0.646 − 1.114)	6471 (5366 − 8576)	0.841 (0.7 − 1.127)	0.075 (0.038–0.113)	86247 (65914 − 115678)	24.543 (18.966 − 33.026)	197492 (163149 − 257532)	24.044 (19.889 − 31.463)	−0.01 (–0.048–0.029)
	Male	9942 (7940 − 12449)	2.91 (2.322 − 3.646)	30912 (26352 − 35679)	4.168 (3.56 − 4.794)	1.264 (1.191–1.338)	9236 (7370 − 11560)	2.77 (2.205 − 3.471)	26273 (22421 − 30220)	3.639 (3.11 − 4.188)	0.989 (0.938–1.04)	292738 (234521 − 366165)	81.584 (65.258 − 102.11)	794586 (676662 − 919383)	103.005 (87.91 − 118.807)	0.862 (0.81–0.915)
Middle SDI	Female	2258 (1871 − 2701)	0.406 (0.34 − 0.488)	6849 (5596 − 9147)	0.476 (0.387 − 0.634)	0.38 (0.275–0.486)	1876 (1564 − 2253)	0.352 (0.298 − 0.424)	4349 (3608 − 5741)	0.307 (0.253 − 0.405)	−0.555 (–0.609to–0.501)	56900 (46487 − 68429)	9.627 (7.953 − 11.552)	123313 (102485 − 162424)	8.535 (7.074 − 11.234)	−0.518 (–0.586to–0.449)
	Male	9226 (8334 − 10119)	1.677 (1.523 − 1.838)	29328 (26084 − 32575)	2.146 (1.911 − 2.385)	0.605 (0.453–0.757)	8049 (7285 − 8819)	1.521 (1.383 − 1.664)	20893 (18508 − 23334)	1.571 (1.398 − 1.754)	−0.089 (–0.2–0.022)	253050 (229445 − 277618)	43.175 (39.073 − 47.197)	616853 (545011 − 688151)	44.093 (39.037 − 49.071)	−0.141 (–0.251to–0.03)
High-middle SDI	Female	1666 (1537 − 1803)	0.3 (0.277 − 0.324)	4609 (4070 − 5250)	0.448 (0.397 − 0.51)	1.341 (1.208–1.475)	1122 (1028 − 1219)	0.203 (0.186 − 0.22)	2032 (1781 − 2298)	0.19 (0.167 − 0.215)	−0.33 (–0.426to–0.235)	30758 (28043 − 33362)	5.53 (5.04 − 5.992)	52605 (46738 − 60012)	5.213 (4.638 − 5.963)	−0.319 (–0.41to–0.228)
	Male	12324 (11644 − 13136)	2.58 (2.436 − 2.753)	25485 (23267 − 27774)	2.713 (2.479 − 2.954)	−0.048 (–0.195–0.101)	8273 (7749 − 8893)	1.792 (1.679 − 1.924)	12480 (11415 − 13523)	1.344 (1.23 − 1.455)	−1.198 (–1.329to–1.067)	259473 (243423 − 278326)	53.122 (49.793 − 56.971)	363067 (332010 − 393250)	38.593 (35.316 − 41.768)	−1.342 (–1.474to–1.209)
High SDI	Female	4215 (3983 − 4463)	0.72 (0.683 − 0.761)	10775 (9885 − 11477)	1.101 (1.028 − 1.169)	1.5 (1.395–1.605)	1724 (1614 − 1845)	0.28 (0.263 − 0.299)	3319 (2928 − 3574)	0.302 (0.275 − 0.323)	0.278 (0.112–0.444)	43921 (41666 − 46612)	7.666 (7.301 − 8.134)	78995 (73022 − 84415)	8.257 (7.723 − 8.793)	0.275 (0.127–0.424)
	Male	18697 (17979 − 19454)	3.875 (3.727 − 4.031)	44967 (42602 − 47173)	4.989 (4.742 − 5.233)	0.871 (0.798–0.944)	8438 (8097 − 8817)	1.767 (1.696 − 1.847)	15228 (14311 − 16071)	1.637 (1.539 − 1.726)	−0.255 (–0.349to–0.161)	255095 (243951 − 267352)	52.732 (50.411 − 55.292)	389579 (366155 − 413104)	43.983 (41.387 − 46.631)	−0.636 (–0.719to–0.554)
GBD regions																
Central Asia	Female	138 (123 − 156)	0.492 (0.436 − 0.556)	230 (194 − 270)	0.469 (0.396 − 0.548)	−0.278 (–0.62–0.066)	109 (97 − 125)	0.392 (0.346 − 0.45)	154 (129 − 180)	0.323 (0.273 − 0.377)	−0.785 (–1.084to–0.486)	3353 (2990 − 3769)	11.762 (10.498 − 13.22)	4901 (4070 − 5896)	9.795 (8.165 − 11.742)	−0.759 (–1.055to–0.463)
	Male	358 (323 − 400)	1.706 (1.54 − 1.908)	458 (402 − 523)	1.177 (1.038 − 1.335)	−1.273 (–1.475to–1.071)	303 (273 − 338)	1.498 (1.35 − 1.687)	363 (317 − 417)	0.972 (0.854 − 1.113)	−1.48 (–1.69to–1.269)	9474 (8549 − 10586)	42.704 (38.621 − 47.642)	10797 (9382 − 12473)	26.097 (22.866 − 29.974)	−1.739 (–1.952to–1.525)
Central Europe	Female	412 (386 − 449)	0.503 (0.47 − 0.548)	1403 (1268 − 1533)	1.316 (1.189 − 1.445)	3.147 (2.988–3.307)	277 (258 − 302)	0.334 (0.312 − 0.364)	642 (582 − 704)	0.551 (0.498 − 0.604)	1.61 (1.427–1.792)	7686 (7187 − 8401)	9.558 (8.939 − 10.43)	16229 (14661 − 17731)	15.719 (14.16 − 17.23)	1.574 (1.417–1.732)
	Male	2642 (2505 − 2793)	3.849 (3.655 − 4.063)	6254 (5613 − 6899)	6.878 (6.169 − 7.582)	1.853 (1.715–1.992)	1956 (1853 − 2069)	2.88 (2.734 − 3.041)	3610 (3255 − 3988)	3.91 (3.523 − 4.317)	0.972 (0.881–1.063)	63310 (60016 − 66988)	91.418 (86.822 − 96.545)	104928 (93755 − 116151)	117.194 (104.826 − 129.575)	0.722 (0.592–0.853)
Eastern Europe	Female	558 (521 − 610)	0.335 (0.312 − 0.369)	1405 (1240 − 1595)	0.791 (0.693 − 0.905)	2.813 (2.422–3.204)	348 (331 − 374)	0.199 (0.189 − 0.214)	616 (552 − 683)	0.311 (0.278 − 0.346)	1.216 (0.999–1.433)	9319 (8837 − 10093)	5.653 (5.354 − 6.133)	17199 (15272 − 19239)	9.69 (8.569 − 10.921)	1.569 (1.343–1.795)
	Male	5248 (4910 − 5767)	4.525 (4.239 − 4.96)	10537 (9365 − 11751)	7.47 (6.657 − 8.331)	1.504 (1.251–1.757)	3275 (3048 − 3625)	2.926 (2.727 − 3.23)	5046 (4457 − 5753)	3.566 (3.15 − 4.062)	0.421 (0.262–0.581)	105626 (98241 − 117224)	89.793 (83.704 − 99.67)	154223 (135560 − 176945)	109.154 (95.936 − 124.986)	0.351 (0.178–0.524)
Australasia	Female	134 (121 − 147)	1.094 (0.99 − 1.197)	328 (281 − 379)	1.285 (1.112 − 1.475)	0.5 (0.239–0.761)	40 (36 − 43)	0.309 (0.28 − 0.336)	67 (56 − 78)	0.237 (0.201 − 0.272)	−0.872 (–1.259to–0.483)	996 (906 − 1079)	8.223 (7.499 − 8.913)	1608 (1386 − 1832)	6.327 (5.52 − 7.178)	−0.858 (–1.212to–0.503)
	Male	503 (453 − 555)	4.63 (4.176 − 5.109)	1388 (1180 − 1608)	5.988 (5.082 − 6.956)	0.81 (0.445–1.177)	154 (140 − 169)	1.431 (1.307 − 1.579)	288 (246 − 333)	1.186 (1.016 − 1.365)	−0.719 (–1.135to–0.302)	4351 (3950 − 4803)	40.028 (36.193 − 44.248)	7661 (6571 − 8821)	33.382 (28.71 − 38.3)	−0.63 (–1.034to–0.225)
High-income Asia Pacific	Female	349 (326 − 370)	0.31 (0.29 − 0.328)	1248 (1071 − 1372)	0.607 (0.534 − 0.66)	2.21 (1.911–2.51)	145 (133 − 153)	0.128 (0.118 − 0.136)	429 (344 − 479)	0.161 (0.139 − 0.176)	0.76 (0.578–0.942)	3738 (3512 − 3946)	3.342 (3.14 − 3.531)	8505 (7291 − 9309)	4.214 (3.787 − 4.544)	0.773 (0.569–0.978)
	Male	1541 (1470 − 1603)	1.641 (1.566 − 1.708)	8462 (7805 − 9052)	4.305 (4.003 − 4.599)	3.089 (2.661–3.519)	735 (708 − 762)	0.817 (0.788 − 0.847)	3375 (3119 − 3567)	1.599 (1.483 − 1.692)	2.088 (1.76–2.417)	20477 (19720 − 21264)	21.413 (20.647 − 22.209)	70086 (65280 − 74211)	36.421 (33.993 − 38.635)	1.62 (1.253–1.988)
High-income North America	Female	1892 (1779 − 1961)	1.038 (0.987 − 1.072)	3407 (3164 − 3585)	1.046 (0.976 − 1.098)	0.101 (–0.066–0.268)	618 (572 − 645)	0.317 (0.297 − 0.329)	898 (808 − 955)	0.254 (0.232 − 0.268)	−0.67 (–0.898to–0.441)	15496 (14667 − 16083)	8.631 (8.247 − 8.924)	21596 (20230 − 22771)	6.739 (6.359 − 7.086)	−0.697 (–0.945to–0.447)
	Male	6036 (5879 − 6183)	4.108 (3.999 − 4.209)	14300 (13741 − 14819)	4.93 (4.738 − 5.11)	0.8 (0.631 − 0.969)	1612 (1564 − 1652)	1.093 (1.059 − 1.121)	3094 (2952 − 3215)	1.036 (0.988 − 1.076)	0.016 (–0.265–0.298)	46234 (44864 − 47665)	31.569 (30.62 − 32.565)	82975 (79444 − 86523)	28.854 (27.643 − 30.077)	−0.078 (–0.332–0.177)
Southern Latin America	Female	85 (75 − 96)	0.335 (0.297 − 0.379)	129 (108 − 152)	0.276 (0.232 − 0.325)	−0.056 (–0.624–0.515)	64 (57 − 73)	0.253 (0.224 − 0.288)	75 (62 − 88)	0.153 (0.127 − 0.179)	−1.008 (–1.596to–0.416)	1620 (1440 − 1825)	6.439 (5.726 − 7.232)	1759 (1489 − 2064)	3.847 (3.263 − 4.516)	−1.042 (–1.623to–0.457)
	Male	501 (446 − 560)	2.338 (2.081 − 2.616)	427 (369 − 495)	1.106 (0.953 − 1.283)	−2.091 (–2.431to–1.749)	385 (346 − 428)	1.831 (1.644 − 2.037)	264 (227 − 303)	0.685 (0.59 − 0.789)	−2.765 (–3.117to–2.411)	11351 (10070 − 12743)	52.173 (46.289 − 58.581)	7145 (6124 − 8325)	18.488 (15.806 − 21.602)	−2.986 (–3.336to–2.635)
Western Europe	Female	1888 (1766 − 2072)	0.652 (0.613 − 0.716)	5658 (5148 − 6199)	1.385 (1.272 − 1.508)	2.592 (2.427–2.758)	916 (850 − 1011)	0.293 (0.273 − 0.323)	1804 (1596 − 1994)	0.387 (0.35 − 0.426)	0.918 (0.755–1.081)	23348 (21835 − 25648)	8.342 (7.812 − 9.171)	43601 (39589 − 47997)	10.865 (9.956 − 11.909)	0.846 (0.697–0.995)
	Male	11495 (10847 − 12204)	4.859 (4.587 − 5.153)	19923 (18225 − 21725)	5.371 (4.917 − 5.864)	0.13 (–0.026–0.287)	6089 (5756 − 6439)	2.563 (2.425 − 2.709)	7384 (6718 − 8119)	1.89 (1.723 − 2.079)	−1.206 (–1.339to–1.073)	187512 (176917 − 198450)	79.776 (75.316 − 84.432)	193465 (176185 − 214598)	52.901 (48.138 − 58.54)	−1.609 (–1.746to–1.472)
Andean Latin America	Female	31 (24 − 38)	0.283 (0.222 − 0.343)	75 (57 − 103)	0.238 (0.183 − 0.327)	−0.741 (–1.023to–0.458)	27 (21 − 33)	0.256 (0.2 − 0.31)	50 (38 − 66)	0.159 (0.124 − 0.211)	−1.719 (–1.966to–1.471)	807 (622 − 977)	6.873 (5.318 − 8.33)	1328 (1009 − 1811)	4.168 (3.174 − 5.672)	−1.815 (–2.063to–1.566)
	Male	56 (49 − 66)	0.556 (0.483 − 0.647)	119 (92 − 149)	0.416 (0.323 − 0.523)	−1.028 (–1.55to–0.502)	53 (46 − 61)	0.534 (0.468 − 0.619)	92 (72 − 116)	0.329 (0.259 − 0.413)	−1.645 (–2.128to–1.159)	1486 (1296 − 1752)	13.81 (12.06 − 16.2)	2398 (1881 − 3059)	8.208 (6.466 − 10.467)	−1.809 (–2.297to–1.319)
Caribbean	Female	95 (85 − 105)	0.708 (0.632 − 0.779)	139 (115 − 166)	0.487 (0.402 − 0.582)	−1.089 (–1.341to–0.835)	74 (65 − 83)	0.566 (0.498 − 0.632)	100 (81 − 122)	0.345 (0.279 − 0.421)	−1.446 (–1.703to–1.189)	1912 (1682 − 2186)	13.917 (12.272 − 15.835)	2444 (1950 − 2994)	8.701 (6.935 − 10.686)	−1.371 (–1.623to–1.118)
	Male	356 (328 − 386)	2.836 (2.609 − 3.074)	677 (576 − 789)	2.638 (2.244 − 3.067)	0.046 (–0.373–0.467)	290 (267 − 316)	2.356 (2.169 − 2.567)	480 (407 − 564)	1.888 (1.6 − 2.216)	−0.432 (–0.848to–0.015)	7813 (7148 − 8572)	60.818 (55.631 − 66.751)	12967 (10881 − 15449)	50.193 (42.173 − 59.657)	−0.344 (–0.754–0.068)
Central Latin America	Female	116 (110 − 122)	0.267 (0.252 − 0.282)	290 (253 − 330)	0.213 (0.186 − 0.242)	−1.234 (–1.618to–0.849)	98 (93 − 104)	0.238 (0.224 − 0.251)	200 (175 − 227)	0.149 (0.13 − 0.169)	−1.949 (–2.327to–1.569)	2677 (2538 − 2816)	5.708 (5.401 − 6.008)	5015 (4346 − 5735)	3.651 (3.168 − 4.17)	−1.92 (–2.313to–1.526)
	Male	283 (271 − 296)	0.703 (0.672 − 0.733)	750 (658 − 856)	0.644 (0.565 − 0.733)	−0.459 (–0.637to–0.28)	254 (243 − 266)	0.653 (0.624 − 0.68)	575 (505 − 656)	0.505 (0.444 − 0.574)	−0.995 (–1.175to–0.814)	6966 (6668 − 7310)	16.259 (15.569 − 17.041)	14853 (13013 − 17091)	12.459 (10.926 − 14.316)	−1.02 (–1.211to–0.829)
Tropical Latin America	Female	313 (295 − 333)	0.628 (0.589 − 0.67)	858 (791 − 919)	0.604 (0.558 − 0.648)	−0.125 (–0.25–0.001)	257 (242 − 274)	0.539 (0.505 − 0.576)	587 (533 − 630)	0.412 (0.375 − 0.443)	−0.779 (–0.92to–0.637)	7373 (6988 − 7817)	13.998 (13.229 − 14.853)	15477 (14335 − 16535)	10.941 (10.137 − 11.692)	−0.801 (–0.943to–0.659)
	Male	1593 (1508 − 1681)	3.42 (3.24 − 3.605)	4433 (4118 − 4730)	3.632 (3.374 − 3.875)	0.073 (–0.089–0.236)	1351 (1282 − 1424)	3.001 (2.839 − 3.164)	3242 (3008 − 3457)	2.704 (2.506 − 2.886)	−0.418 (–0.578to–0.259)	42477 (40275 − 44746)	86.803 (82.321 − 91.411)	95698 (88783 − 102413)	77.205 (71.647 − 82.537)	−0.511 (–0.697to–0.325)
North Africa and Middle East	Female	219 (170 − 286)	0.25 (0.196 − 0.332)	706 (562 − 922)	0.292 (0.231 − 0.379)	0.588 (0.564–0.612)	177 (137 − 232)	0.215 (0.168 − 0.286)	400 (320 − 510)	0.179 (0.141 − 0.227)	−0.476 (–0.507to–0.446)	5429 (4172 − 7297)	5.727 (4.443 − 7.548)	12065 (9575 − 15314)	4.75 (3.792 − 6.03)	−0.538 (–0.578to–0.498)
	Male	339 (282 − 400)	0.378 (0.315 − 0.447)	1071 (893 − 1308)	0.434 (0.364 − 0.526)	0.517 (0.48–0.553)	294 (244 − 350)	0.344 (0.286 − 0.409)	717 (597 − 875)	0.31 (0.262 − 0.376)	−0.295 (–0.323to–0.267)	8812 (7392 − 10465)	9.154 (7.628 − 10.868)	20925 (17274 − 25854)	8.008 (6.643 − 9.817)	−0.384 (–0.415to–0.353)
South Asia	Female	4100 (3117 − 5361)	1.344 (1.017 − 1.758)	12246 (9903 − 16192)	1.532 (1.242 − 2.039)	0.412 (0.334–0.49)	3608 (2744 − 4722)	1.225 (0.926 − 1.605)	9258 (7524 − 12214)	1.189 (0.969 − 1.585)	−0.088 (–0.135to–0.041)	118169 (88940 − 156301)	36.138 (27.424 − 47.477)	284538 (229894 − 371783)	34.484 (27.886 − 45.12)	−0.133 (–0.18to–0.087)
	Male	14573 (12026 − 17915)	4.308 (3.545 − 5.288)	49124 (42000 − 56353)	6.317 (5.406 − 7.238)	1.26 (1.205–1.316)	13408 (11020 − 16518)	4.072 (3.333 − 5.027)	40586 (34660 − 46479)	5.354 (4.592 − 6.118)	0.921 (0.888–0.953)	429706 (354029 − 531512)	120.45 (99.018 − 148.546)	1226521 (1046018 − 1407575)	152.464 (130.077 − 174.744)	0.79 (0.758–0.822)
East Asia	Female	799 (581 − 986)	0.178 (0.133 − 0.219)	2382 (1678 − 3299)	0.211 (0.149 − 0.292)	0.478 (0.29–0.665)	654 (480 − 802)	0.153 (0.115 − 0.19)	1072 (739 − 1437)	0.095 (0.066 − 0.127)	−1.838 (–1.967to–1.71)	18172 (12644 − 22729)	3.824 (2.732 − 4.745)	25266 (17681 − 34885)	2.267 (1.587 − 3.137)	−1.949 (–2.067to–1.83)
	Male	4649 (3710 − 5796)	1.048 (0.85 − 1.294)	11897 (9348 − 15010)	1.093 (0.861 − 1.372)	−0.135 (–0.54–0.271)	3916 (3118 − 4886)	0.935 (0.759 − 1.148)	5817 (4596 − 7271)	0.561 (0.445 − 0.695)	−2.112 (–2.495to–1.728)	118742 (94078 − 148674)	24.83 (19.817 − 31.025)	160699 (125795 − 203152)	14.661 (11.542 − 18.39)	−2.158 (–2.55to–1.764)
Oceania	Female	1 (1 − 2)	0.092 (0.065 − 0.122)	4 (2 − 5)	0.085 (0.059 − 0.119)	−0.225 (–0.305to–0.145)	1 (1 − 2)	0.082 (0.058 − 0.107)	3 (2 − 4)	0.073 (0.051 − 0.102)	−0.289 (–0.36to–0.218)	42 (27 − 58)	2.349 (1.581 − 3.182)	100 (69 − 144)	2.118 (1.477 − 2.976)	−0.284 (–0.344to–0.224)
	Male	4 (3 − 5)	0.212 (0.157 − 0.296)	9 (7 − 12)	0.215 (0.167 − 0.284)	0.269 (0.122–0.416)	3 (2 − 5)	0.198 (0.146 − 0.279)	8 (6 − 11)	0.197 (0.152 − 0.264)	0.205 (0.057–0.353)	108 (78 − 155)	5.656 (4.177 − 8.03)	268 (202 − 364)	5.56 (4.234 − 7.465)	0.154 (0.012–0.296)
Southeast Asia	Female	598 (463 − 826)	0.426 (0.326 − 0.592)	1477 (1160 − 2254)	0.41 (0.323 − 0.63)	−0.306 (–0.366to–0.246)	505 (387 − 711)	0.375 (0.289 − 0.532)	1008 (805 − 1574)	0.291 (0.233 − 0.456)	−0.982 (–1.049to–0.915)	14860 (11480 − 20393)	9.87 (7.6 − 13.781)	27654 (21575 − 42238)	7.478 (5.876 − 11.499)	−1.072 (–1.137to–1.007)
	Male	1628 (1334 − 1978)	1.277 (1.049 − 1.546)	5439 (4450 − 6516)	1.639 (1.353 − 1.936)	0.724 (0.6–0.849)	1437 (1180 − 1744)	1.171 (0.964 − 1.402)	3948 (3274 − 4704)	1.25 (1.046 − 1.468)	0.111 (0.019–0.203)	43707 (35912 − 53082)	32.119 (26.42 − 38.927)	117291 (96567 − 141467)	33.816 (28.103 − 40.488)	0.075 (–0.027–0.177)
Central Sub-Saharan Africa	Female	22 (15 − 32)	0.175 (0.118 − 0.243)	65 (43 − 89)	0.203 (0.133 − 0.277)	0.552 (0.461–0.643)	21 (14 − 29)	0.17 (0.116 − 0.235)	56 (36 − 76)	0.186 (0.123 − 0.254)	0.39 (0.328–0.451)	649 (429 − 928)	4.62 (3.109 − 6.487)	1751 (1132 − 2405)	4.971 (3.187 − 6.771)	0.334 (0.267–0.401)
	Male	54 (40 − 69)	0.466 (0.343 − 0.594)	139 (98 − 196)	0.479 (0.339 − 0.686)	0.19 (–0.053–0.434)	51 (38 − 66)	0.458 (0.342 − 0.585)	125 (87 − 179)	0.453 (0.321 − 0.658)	0.08 (–0.139–0.299)	1620 (1176 − 2087)	13.003 (9.554 − 16.609)	4069 (2825 − 5787)	12.638 (8.854 − 18.098)	0.019 (–0.199–0.238)
Eastern Sub-Saharan Africa	Female	194 (113 − 246)	0.465 (0.282 − 0.583)	529 (320 − 686)	0.522 (0.309 − 0.668)	0.361 (0.335–0.388)	175 (104 − 223)	0.437 (0.268 − 0.547)	441 (266 − 573)	0.457 (0.27 − 0.585)	0.161 (0.138–0.184)	5727 (3279 − 7407)	12.673 (7.404 − 16.208)	14639 (8862 − 19180)	13.206 (7.914 − 17.178)	0.126 (0.101–0.15)
	Male	378 (292 − 484)	0.917 (0.712 − 1.168)	919 (646 − 1337)	0.982 (0.696 − 1.408)	0.119 (0.08–0.158)	355 (275 − 455)	0.883 (0.688 − 1.122)	810 (567 − 1182)	0.898 (0.641 − 1.286)	−0.031 (–0.061to–0.002)	11290 (8714 − 14566)	26.007 (20.119 − 33.36)	26643 (18541 − 39302)	26.514 (18.519 − 38.738)	−0.032 (–0.062to–0.002)
Southern Sub-Saharan Africa	Female	51 (38 − 66)	0.314 (0.231 − 0.415)	136 (97 − 164)	0.388 (0.273 − 0.463)	1.036 (0.897–1.174)	42 (31 − 55)	0.265 (0.195 − 0.35)	103 (72 − 122)	0.301 (0.211 − 0.355)	0.742 (0.618–0.866)	1310 (971 − 1691)	7.712 (5.78 − 10.1)	3091 (2227 − 3757)	8.536 (6.101 − 10.298)	0.714 (0.568–0.859)
	Male	133 (107 − 170)	1.012 (0.809 − 1.31)	386 (325 − 456)	1.397 (1.187 − 1.638)	1.048 (0.821–1.276)	114 (92 − 147)	0.902 (0.721 − 1.175)	310 (262 − 365)	1.168 (0.995 − 1.357)	0.839 (0.558–1.12)	3737 (3027 − 4736)	26.957 (21.75 − 34.58)	9937 (8305 − 11840)	34.08 (28.651 − 40.182)	0.748 (0.463–1.034)
Western Sub-Saharan Africa	Female	18 (12 − 22)	0.039 (0.028 − 0.049)	41 (28 − 54)	0.037 (0.027 − 0.05)	−0.183 (–0.243to–0.124)	16 (12 − 21)	0.038 (0.028 − 0.048)	36 (25 − 47)	0.035 (0.025 − 0.047)	−0.218 (–0.28to–0.156)	497 (340 − 622)	1.022 (0.711 − 1.283)	1118 (732 − 1454)	0.876 (0.604 − 1.148)	−0.571 (–0.639to–0.502)
	Male	146 (113 − 185)	0.284 (0.22 − 0.356)	352 (270 − 452)	0.313 (0.245 − 0.397)	0.363 (0.238–0.487)	135 (104 − 170)	0.269 (0.209 − 0.337)	303 (237 − 387)	0.281 (0.223 − 0.353)	0.19 (0.081–0.299)	4500 (3453 − 5752)	8.266 (6.359 − 10.505)	10347 (7951 − 13358)	8.615 (6.706 − 11.039)	0.175 (0.059–0.291)

### Deaths and trends of PC

**Figure 3.**
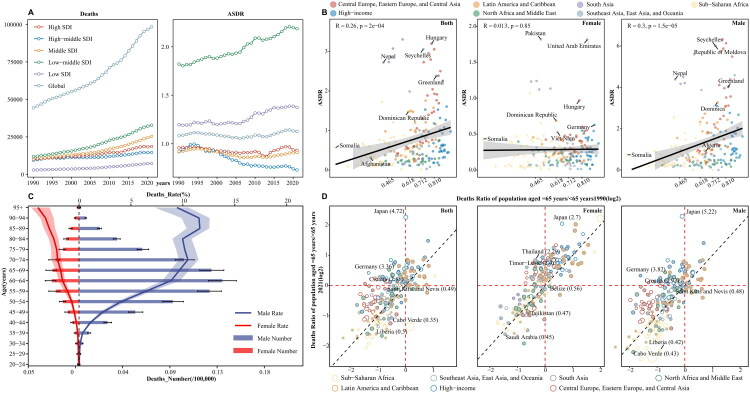
Deaths burden and trends of pharyngeal cancer from 1990 to 2021 across different SDI regions, age groups, and genders. **A**: Deaths numbers and ASDR of pharyngeal cancer globally and across 5 SDI level regions; **B**: Correlation between the ASDR of pharyngeal cancer and SDI index across 204 countries and territories globally by gender. R, the correlation coefficient; **C**: Deaths burden of pharyngeal cancer across different age groups (5 year intervals) by gender in 2021; **D**: Age-related deaths ratio of pharyngeal cancer between 2000 and 2021 in 204 countries and territories. Points filled by grey color represent an annual change less than 0. Different colors indicate 7 GBD super-regions. Point size represents the absolute annual change of the age-related Deaths rate from 2000 to 2021. ASDR, age-standardized deaths rate.

In 2021, the number of deaths due to PC in males globally was 80,437 (73,959–87,131) deaths, which was higher than that of females at 17,998 (15,452–22,431) deaths. Additionally, the ASDR for males was 1.939 (1.786–2.099) compared with 0.394 (0.339–0.491) for females. From 1990 to 2021, the EAPC of the ASDR for males was 0.046 (−0.038–0.131), which was lower than that of females at 0.092 (0.027–0.158) (Table 2). In low-middle SDI regions, the ASDR in 2021 was highest at 2.19 (1.93–2.46), with the highest EAPC for ASDR from 1990 to 2021 at 0.684 (0.638–0.729) (Figure 3A). Interestingly, the highest EAPC of the ASDR for females was 0.278 (0.112–0.444) in high SDI regions. A positive correlation was observed between SDI and ASDR across 204 countries (*R* = 0.26, *p* < 0.05), similar to that in males-only (*R* = 0.3, *p* < 0.05), but not in females-only (*R* = 0.013, *p* = 0.85) (Figure 3B). In 2021, the highest number of deaths occurred in the 60–64-year age group, totaling 16,401.78 (15,033.78–17,802.95) deaths, including 2,616.69 (2,213.08–3,260.33) females (Figure 3C, Supplementary Fig. 1B). In the 85–89-year age group, the death rate increased annually in most GBD regions, except in Latin America and the Caribbean, Southeast Asia, East Asia, and Oceania (Supplementary Fig. 2B). In Japan, the age-related death rate in 2021 was 4.72-times higher than in 1990, whereas Cabo Verde had the lowest ratio at 0.35-times that in 1990. Additionally, in Japan, males had a higher rate, at 5.22, compared to females, who had a rate of 2.7 (Figure 3D).

### DALYs and trends of PC

**Figure 4.**
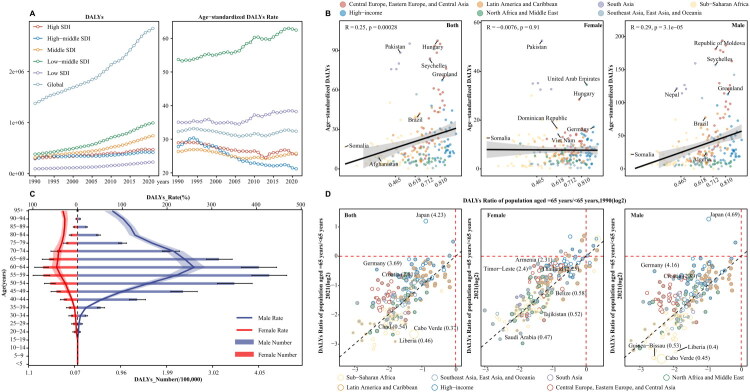
DALYs Burden and trends of pharyngeal cancer from 1990 to 2021 across different SDI regions, age groups, and genders. **A**: DALYs and age-standardized DALYs rate of pharyngeal cancer globally and across 5 SDI level regions; **B**: Correlation between the age-standardized DALYs rate of pharyngeal cancer and SDI index across 204 countries and territories globally by gender. R, the correlation coefficient; **C**: DALYs burden of pharyngeal cancer across different age groups (5 year intervals) by gender in 2021; **D**: Age-related DALYs ratio of pharyngeal cancer between 2000 and 2021 in 204 countries and territories. Points filled by grey color represent an annual change less than 0. Different colors indicate 7 GBD super-regions. Point size represents the absolute annual change of the Age-related DALYs rate from 2000 to 2021. DALYs, disability-adjusted life years.

In 2021, the global DALYs for males was 2,333,897 (2,128,450–2,539,518) DALYs, which was higher than that for females at 509,884 (440,655–635,095) DALYs. Concurrently, the age-standardized DALYs rate for males was 54.775 (50.003–59.57) compared with 11.379 (9.796–14.188) for females. From 1990 to 2021, the EAPC for the age-standardized DALYs rate was −0.108 (−0.189–0.026) for males and 0.072 (0.003–0.142) for females (Table 2). The highest age-standardized DALYs rate in low-middle SDI regions was 62.45 (54.65–70.13) with the highest EAPC at 0.582 (0.535–0.628), while the lowest age-standardized DALYs rate in high-middle SDI regions was 21.20 (19.56–22.83) with the lowest EAPC at −1.183 (−1.311–1.054) (Figure 4A). The age-standardized DALYs rate was positively correlated with SDI across 204 countries in 2021 (*R* = 0.25, *p* < 0.05), whereas there was no significant correlation for females-only (*R* = 0.0076, *p* = 0.91) (Figure 4B). The highest DALYs rate was in the 60–64-year age group at 150.72 (138.31–163.64), with males-only at 260.51 (235.63–286.13) and females-only at 46.91 (39.71–58.34) (Figure 4C, Supplementary Figure 1C). Similar to trends in deaths, the DALYs rate showed a steady increase from 1990 to 2021, except for in Latin America and the Caribbean, Southeast Asia, East Asia, and Oceania (Supplementary Figure 2C). In Japan, the age-related DALYs rate in 2021 was 4.23-times higher than that in 1990, whereas Cabo Verde had the lowest ratio at 0.37-times that in 1990.

### Inequalities in PC burden across regions

**Figure 5.**
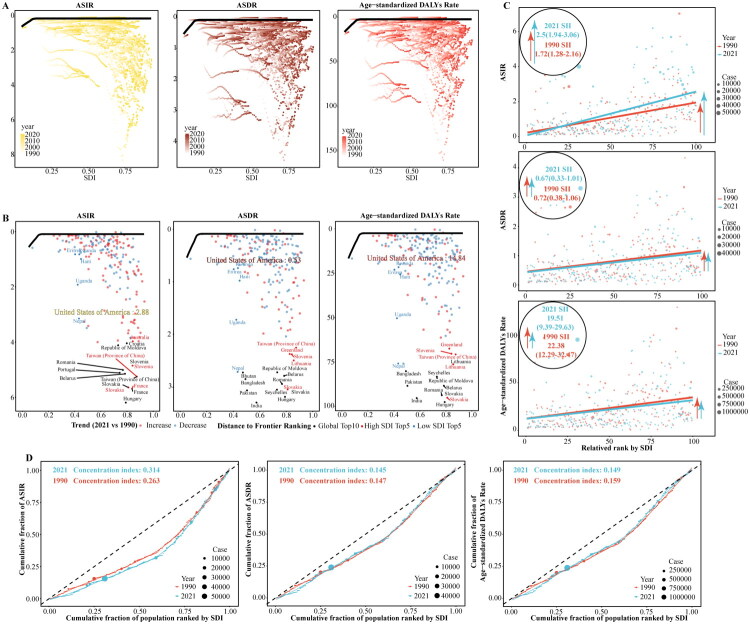
Frontier and health inequality analysis of ASIR, ASDR, and age-standardized DALYs rates for pharyngeal cancer based on SDI in 204 countries and territories. **A**. Frontier curves of ASIR, ASDR, and age-standardized DALYs rates for pharyngeal cancer, 1990–2021. **B**. Frontier curves of ASIR, ASDR, and age-standardized DALYs rates for pharyngeal cancer based on 2021 SDI. **C.** Slope index of inequality of ASIR, ASDR, and age-standardized DALYs rates for pharyngeal cancer for 1990 and 2021. **D.** Concentration index of ASIR, ASDR, and age-standardized DALYs rates for pharyngeal cancer for 1990 and 2021. ASIR: Age-standardized incidence rate; ASDR: Age-standardized death rate; DALYs: Disability-adjusted life years; SII: Slope index of inequality

Frontier analysis results indicated that the frontier curves for PC incidence, mortality, and DALYs approached zero, suggesting significant potential for improvement in most countries (Figure 5A). Hungary demonstrated the highest potential for improvement in ASIR (6.1033) and age-standardized DALYs rates (95.04), while India had the highest potential for improvement in ASDR (3.22); among the top ten regions globally for ASIR improvement, France (5.59), Slovakia (5.55), Taiwan (Province of China) (5.16), and Slovenia (4.76) were from high SDI regions, for ASDR improvement, only Slovenia (2.966) was from a high SDI region, and for age-standardized DALYs rate improvement, Slovenia (92.59) and Lithuania (73.83) were from high SDI regions (Figure 5B). The objective inequality index analysis revealed that the SII for PC ASIR in 2021 was 2.5 (1.94–3.06), higher than 1.72 (1.28–2.16) in 1990; the SII for PC ASDR in 2021 was 0.67 (0.33–1.01), lower than 0.72 (0.38–1.06) in 1990; and the SII for the age-standardized DALYs rate of PC in 2021 was 19.51 (9.39–29.63), lower than 22.38 (12.29–32.47) in 1990 (Figure 5C). In 2021, the concentration index for PC ASIR was 0.314 (0.159–0.468), higher than the value of 0.263 (0.127–0.399) in 1990; the concentration index for PC ASDR in 2021 was 0.145 (0.061–0.228), slightly lower than 0.147 (0.055–0.24) in 1990; and the concentration index for the age-standardized DALYs rate of PC in 2021 was 0.149 (0.06–0.237), slightly lower than 0.159 (0.058–0.259) in 1990 (Figure 5D).

### Prediction of PC burden to 2050

**Figure 6.**
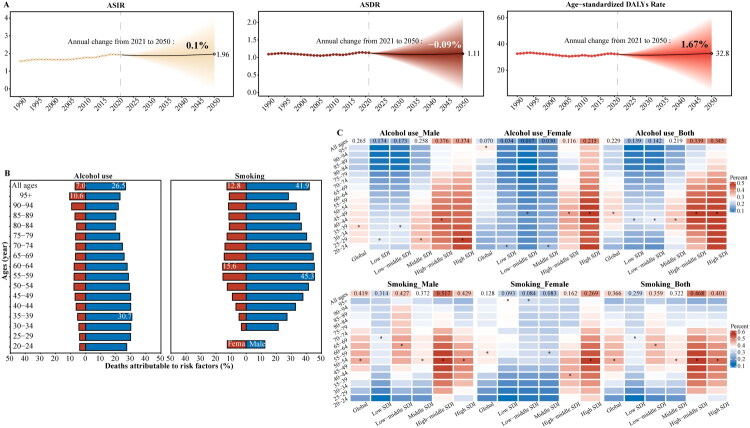
Risk factors associated with mortality and prediction of disease burden for pharyngeal cancer. **A.** Bayesian age-period-cohort projections for pharyngeal cancer from 2022 to 2050. **B.** Risk factors fordeaths by age groups associated with smoking and alcohol for global pharyngeal cancer in 2021. **C.** Risk factors for deaths by age groups associated with smoking and alcohol use for pharyngeal cancer across SDI regions and genders. BAPC: Bayesian Age-Period-Cohort

Based on the BCPA model, the ASIR of PC in 2050 was projected to be 1.96 per 100,000 population, with an annual percentage change (APC) of 0.1% from 2021 to 2050. The ASDR for PC in 2050 was predicted to be 1.11 per 100,000, with an APC of −0.09% over the same period. Additionally, the age-standardized DALYs rate was estimated to reach 32.8 per 100,000 in 2050, with an APC of 1.67% from 2021 to 2050 (Figure 6A). Importantly, validation using the Nordpred model yielded consistent epidemiological trends (Supplementary Table 4 and Supplementary Figure 4).

### Risk factors of PC

In 2021, alcohol-related PC deaths were higher in males (26.48; 19.38–33.10%) than in females (6.98; 4.77–9.28%); specifically, the highest proportion of male alcohol-related PC deaths was in the 35–39-year age group (30.68; 21.60–39.49%), while that for females was in the 95+ years age group (10.59; 6.54–14.82%) (Supplementary Table 5). For smoking-related PC deaths, males had a higher proportion of smoking-related PC deaths (41.93; 34.19–48.99%) than females (12.79; 9.50–16.14%). The highest proportion of males was in the 55–59-year age group (45.31; 37.44–52.58%), and was in the 60–64-year age group (15.62;11.40–20.18%) for females (Figure 6B). From 1990 to 2021, the highest proportion of global PC deaths related to smoking was primarily concentrated in the 50–69-year age group and has shown a decreasing trend in recent years, whereas the highest proportion of global PC deaths related to alcohol use was mainly in the 45–55-year age group, with recent trends showing minimal change. For males, however, there was a trend towards younger ages at death, with the highest death rate occurring in the 35–39-year age group (Supplementary Figure 3). In the low-middle SDI regions, the proportion of deaths attributed to smoking and alcohol use was lower than that in the high-SDI regions for both sexes (Figure 6C).

## Discussion

Our study provides an update on the global burden of PC and deaths related to risk factors from 1990 to 2021. This is the first study to analyze the global burden of PC in 2021 by subgroups, including incidence, death, and DALYs, stratified by SDI, age, and sex.

We found that the global burden of PC, as measured using the ASIR, remained high in 2021 and increased from 1990 to 2021. Differences in ASIR were observed across regions at varying SDI levels, with Central European countries such as Hungary having the highest ASIR [22]. Consistent with previous studies, there was a positive correlation between the SDI and ASIR for PC in different countries [6,23]. We believe that the higher ASIR in the high SDI regions might be attributed to better medical equipment and oral health services. First, the challenge in identifying PC implies that the appropriate selection and use of endoscopic and computed tomography imaging can improve early detection rates [24]. Regions with higher SDI, meaning better economic conditions and advanced medical technologies, may have experienced higher ASIR. Second, in low- and low-middle-income countries with lower educational levels, oral health is often neglected, which could have reduced the detection rates of PC and indirectly led to a lower ASIR [25,26]. Additionally, emerging complementary studies demonstrate a significantly higher incidence of HPV-related PC in regions with high Human Development Index (HDI) [27]. This epidemiological pattern may correlate with prevalent safe sexual practices in high SDI regions, potentially facilitating oropharyngeal HPV transmission [28]. The incidence and ASIR of PC in males was higher than those in females in 2021, which is consistent with the results in 2019 [5]. Notably, the EAPC in ASIR for females was higher than that for males, with this increasing trend being most pronounced in the high SDI regions. The rate of increase in PC incidence among males has slowed, benefiting from recent PC guidelines and restrictions on tobacco and alcohol use [29]. However, the pattern of PC incidence in females varied regionally, and tobacco and alcohol consumption were not the primary factors driving PC in females. We hypothesize that HPV infection may contribute to the observed increase in PC incidence among females, though this requires further virological confirmation [30,31]. Globally, approximately 20–25% of oropharyngeal squamous cell carcinoma cases are caused by HPV [10,32]. Secondly, the incidence of PC was 19-times higher in individuals who were not vaccinated against HPV than in those who were vaccinated. By the way, current global HPV vaccination coverage remains below the 90% target [33], and mortality reductions are anticipated as coverage improves, particularly in low-middle income countries. Universal HPV vaccination is a potential strategy to reduce the incidence of PC [34,35]. We suggest that investing in resources to enhance the public awareness of HPV-related PC and support public health initiatives could help reduce the global burden of PC [36].

Our study showed that the global ASDR for PC in 2021 was 1.13 (1.05–1.21), reflecting a yearly increasing trend, although it slightly decreased compared to that in 2019 [37]. Notably, the ASDR in South Asia, particularly India and Pakistan, was significantly higher than that in other regions. There were significant differences in the ASDR for PC across regions with varying SDI levels. Notably, the low-middle SDI regions had the highest ASDR for PC in 2021, alongside the highest EAPC of ASDR from 1990 to 2021. Therefore, we believe that PC mortality in the low–middle SDI regions continues to worsen. Impoverished regions may have limited diagnostic capabilities for PC, leading to a higher prevalence of advanced-stage disease [38]. Because the survival rate for PC is close to clinical staging, these regions often experience a higher proportion of advanced-stage cases, which contributes to the higher mortality rates observed in low-middle SDI regions [39]. However, advancements in neoadjuvant chemotherapy, chemoradiotherapy, and endoscopic surgery have significantly improved patient outcomes [40,41]. The relatively limited dissemination of these advanced diagnostic and treatment technologies in low-middle SDI regions may impact PC mortality rates locally. Conversely, high-SDI regions exhibit higher incidence but lower mortality rates due to these diagnostic and therapeutic advantages.We recommend the following two priority interventions:(i) Scaling up endoscopic screening programs in high-burden regions, such as South Asia;(ii) Enhancing surgical team training to improve early lesion detection capabilities, thereby reducing the proportion of advanced-stage presentations; (iii) Strengthening oral health resources for low SDI regions, such as such as training community health workers in PC screening, and deploying mobile dental clinics. The ASDR for PC in males was significantly higher than that in females, likely due to differences in lifestyle habits such as smoking and alcohol consumption. First, smoking not only serves as an independent risk factor for PC mortality, but also undermines the effectiveness of PC treatments [40]. Recent studies have shown that smoking increases the risk and burden of HPV-positive oropharyngeal cancer [42]. Second, our results showed that the disparity in ASDR between sexes was greatest in the low–middle SDI regions, such as in South Asia (Supplementary Figure 5). For example, in India, the smoking-related PC mortality rate for males was 35.8%, while for females, it was only 7.56%, indicating the need for intensified efforts to control tobacco use among males in low-middle SDI regions. Alcohol consumption and smoking habits are both predictors of PC mortality [43]. While total alcohol consumption remains stable in high-income countries (potentially due to effective policies such as taxation [44]), it increases in low-middle SDI regions, where regulatory frameworks may be weaker. This divergence underscores that SDI-based correlations alone are insufficient to explain PC mortality trends. Targeted alcohol control measures (e.g. affordability restrictions, public awareness programs) in low-SDI regions could mitigate rising consumption and its health impacts, addressing both mediating factors and equity gaps. Effective control of alcohol consumption in the low-middle and low SDI regions might be a viable strategy for reducing PC mortality rates. Our findings demonstrate that alcohol-attributable PC mortality peaks exceptionally early (30.68% in males aged 35–39), strongly supporting earlier screening initiation at age 30 for high-risk males in endemic regions. Significant variations in age-related PC mortality rates have been observed across countries. Aging regions, generally in high-income countries, often experienced an increase in age-related PC mortality rates, although the annual rate of change in mortality for all ages decreased [45]. In Japan, the proportion of PC deaths related to aging in 2021 was approximately four times higher than that in 1990, while age-related PC mortality rates were similar to non-age-related rates. This indicates that Japan’s aging population may have contributed to the increasing PC mortality rates over the years. Research has elucidated several factors that contribute to the high mortality rates among older patients with PC. Secondary primary malignancies (SPM) are a common cause of death in tonsillar squamous cell carcinoma, and older patients have a higher risk of SPM-related mortality than younger patients [9]. Non-cancer-related deaths and aspiration pneumonia following radical head and neck radiotherapy were more prevalent in older patients, with a significantly higher probability of postoperative aspiration pneumonia than in younger patients [8]. Transitioning to nonsurgical treatment for elderly patients with oropharyngeal and hypopharyngeal cancers improved survival rates from 31 to 51% (*p* < 0.01) and 26–34% (*p* < 0.01), respectively [46]. Selective neck dissection for clinically negative neck lymph nodes was found to be superior to therapeutic neck dissection in improving survival rates and disease-free survival [47]. We considered that implementing non-surgical or selective surgical strategies for elderly PC patients could improve mortality rates.

Utilizing GBD 2021 data, we performed supplementary analyses on COVID-19’s impact on PC, defining pre-pandemic (1990–2019) and pandemic (2020–2022) periods [45]. The results revealed a biphasic effect: short-term reductions in ASIR (−1.13%), ASDR (−1.31%), and age-standardized DALY rate (−1.1%) in 2021 versus 1990 (Supplementary Table 3), likely reflecting diagnostic delays from containment measures. Longitudinal analysis showed increased EAPCs between 2019 and 2021 in ASIR (0.66–0.677%) and ASDR (0.028–0.061%), with attenuated improvement in DALY rate trends (−0.124 to −0.087%), suggesting healthcare system adaptations partially mitigated pandemic disruptions. Similar impacts have been observed in studies of other cancers, as evidenced by the effects on tracheal, bronchus, and lung (TBL) cancer burden during the COVID-19 pandemic [48,49]. Our results demonstrate COVID-19’s dual-phase impact on global PC burden: short-term (2020–2021) declines in ASIR/ASDR, versus long-term (post-2022) incidence acceleration potentially reflecting case backlog clearance and persistent HPV-associated oropharyngeal cancer epidemiology. This study provides quantitative evidence for public health policy, highlighting the critical balance between pandemic control and maintaining cancer early detection resources.

Although the 2021 GBD data provide the most recent and comprehensive epidemiological information on the global burden of PC, it has some limitations. First, the GBD data do not offer detailed information on subtype-specific risk factors for oropharyngeal and hypopharyngeal cancers, limits our analysis of the burden differences across sub-types and prevents a comprehensive evaluation of specific risk factors for different types of PC [22,50]. Second, the GBD estimates are subject to known database limitations, including potential underreporting in low-resource settings and variable quality of cancer registries globally, which may compromise the accuracy of regional cancer risk assessments. Additionally, the GBD dataset lacked information on HPV infection, which limited its ability to conduct homogeneous analyses of the impact of factors, such as alcohol use and smoking, on the PC burden, especially preventing in-depth exploration of the relationship between HPV-related burden and other factors. Lastly, the cross-year aggregation of data may have overlooked the impact of certain short-term public health events on the PC burden, further affecting our analysis of time-sensitive epidemiological trends [51].

## Conclusion

Our study found that the incidence, deaths, and DALYs of global PC increased in 2021 and exhibited significant regional differences. Specifically, low-middle SDI regions, such as South Asia, experienced severe death burdens. Factors affecting females may differ from those affecting males and are not correlated with SDI level. Aging populations may further exacerbate PC mortality rates. Notably, the PC mortality rate related to alcohol in males showed a trend towards death at younger ages, indicating a shift in the epidemiological characteristics. Our study provides a global analysis of the PC burden, revealing incidence trends across different regions and populations. It emphasizes the importance of considering regional differences, gender influences, and aging factors, providing data support for public health interventions in economically disadvantaged regions. We recommend strengthening efforts to control smoking, alcohol consumption, and promote HPV vaccination to reduce the global burden of PC, and offer valuable insights for future research directions.

## Supplementary Material

Supplementary Table 2.docx

3_Supplementary Figures_PC_AM_Revision.docx

Supplementary Table 1.docx

Supplementary Table 3.docx

Supplementary Table 4.docx

Supplementary Table 5.docx

## Data Availability

The data supporting the findings of this study are available upon reasonable request from the corresponding author.

## Patient and public involvement

Patients and/or the public were not involved in the design, conduct, reporting, or dissemination of this research.
