# Supplementary material for: Incidence, mortality, and DALYs of global pharyngeal cancer: systematic analysis and projections Based on global burden of disease study 2021
Source: Ann Med. 2025 Aug 19;57(1):2547092. doi: 10.1080/07853890.2025.2547092 (PMC12366512; doi:10.1080/07853890.2025.2547092)
Supplement: Supplementary Table 2.docx [file IANN_A_2547092_SM9505.docx]

| **Supplementary Table 2 Global burden and trends of pharyngeal cancer from 1990 to 2021** in 204 countries and territories by gender | | | | | | | | | | | | | | | |  |
| --- | --- | --- | --- | --- | --- | --- | --- | --- | --- | --- | --- | --- | --- | --- | --- | --- |
| **Characteristics** | **Gender** | **1990** | | **2021** | |  | **1990** | | **2021** | |  | **1990** | | **2021** | |  |
|  |  | **Incidence cases** | **ASIR** | **Incidence cases** | **ASIR** | **EAPC** | **Death cases** | **ASDR** | **Death cases** | **ASDR** | **EAPC** | **DALYs cases** | **Age_standardised  DALYs Rate** | **DALYs cases** | **Age_standardised  DALYs** | **EAPC** |
|  |  | **(95%UI)** | **per 100,000 (95%UI)** | **(95%UI)** | **per 100,000 (95%UI)** | **(95%CI)** | **(95%UI)** | **per 100,000 (95%UI)** | **(95%UI)** | **per 100,000 (95%UI)** | **(95%CI)** | **(95%UI)** | **per 100,000 (95%UI)** | **(95%UI)** | **per 100,000 (95%UI)** | **(95%CI)** |
| Armenia | Female | 2 (2 - 3) | 0.134 (0.109 - 0.159) | 3 (3 - 4) | 0.143 (0.12 - 0.172) | 0.698 (0.353-1.044) | 2 (1 - 2) | 0.1 (0.083 - 0.119) | 2 (2 - 3) | 0.089 (0.074 - 0.105) | -0.03 (-0.352-0.292) | 47 (37 - 56) | 2.857 (2.308 - 3.411) | 54 (44 - 64) | 2.274 (1.871 - 2.7) | -0.3 (-0.635-0.037) |
|  | Male | 11 (9 - 14) | 0.872 (0.693 - 1.108) | 19 (16 - 23) | 1.031 (0.853 - 1.226) | 0.816 (0.423-1.21) | 9 (7 - 12) | 0.743 (0.587 - 0.943) | 14 (12 - 17) | 0.768 (0.631 - 0.908) | 0.317 (-0.068-0.704) | 279 (221 - 358) | 20.524 (16.25 - 26.226) | 382 (313 - 453) | 20.163 (16.462 - 23.955) | 0.177 (-0.227-0.582) |
| Azerbaijan | Female | 6 (4 - 10) | 0.214 (0.134 - 0.333) | 12 (7 - 20) | 0.205 (0.115 - 0.323) | 0.062 (-0.038-0.163) | 5 (3 - 8) | 0.18 (0.112 - 0.279) | 8 (5 - 13) | 0.142 (0.079 - 0.22) | -0.574 (-0.675to-0.473) | 159 (101 - 245) | 5.15 (3.252 - 7.937) | 252 (139 - 403) | 4.034 (2.248 - 6.355) | -0.667 (-0.781to-0.554) |
|  | Male | 14 (9 - 22) | 0.638 (0.411 - 0.983) | 28 (17 - 45) | 0.561 (0.338 - 0.885) | 0.08 (-0.262-0.424) | 13 (8 - 19) | 0.588 (0.384 - 0.9) | 23 (14 - 35) | 0.469 (0.287 - 0.715) | -0.243 (-0.547-0.062) | 391 (245 - 597) | 16.162 (10.329 - 24.672) | 676 (401 - 1056) | 12.488 (7.491 - 19.279) | -0.432 (-0.724to-0.14) |
| Georgia | Female | 12 (9 - 15) | 0.335 (0.249 - 0.423) | 10 (9 - 12) | 0.324 (0.268 - 0.388) | -0.122 (-0.669-0.429) | 9 (6 - 11) | 0.236 (0.175 - 0.297) | 7 (6 - 9) | 0.213 (0.178 - 0.254) | -0.136 (-0.69-0.421) | 245 (181 - 309) | 6.922 (5.1 - 8.738) | 180 (150 - 215) | 5.971 (4.966 - 7.132) | -0.339 (-0.855-0.179) |
|  | Male | 49 (37 - 67) | 1.861 (1.421 - 2.543) | 63 (54 - 72) | 2.58 (2.246 - 2.953) | 1.518 (0.891-2.149) | 39 (30 - 53) | 1.532 (1.168 - 2.104) | 50 (43 - 57) | 2.059 (1.773 - 2.35) | 1.529 (0.904-2.158) | 1168 (887 - 1606) | 42.966 (32.915 - 59.411) | 1350 (1154 - 1544) | 55.721 (47.909 - 63.646) | 1.393 (0.779-2.01) |
| Kazakhstan | Female | 51 (45 - 57) | 0.647 (0.569 - 0.731) | 55 (44 - 67) | 0.507 (0.411 - 0.614) | -1.197 (-1.754to-0.637) | 39 (35 - 44) | 0.502 (0.445 - 0.565) | 33 (26 - 39) | 0.307 (0.249 - 0.372) | -2.132 (-2.577to-1.685) | 1189 (1050 - 1334) | 15.048 (13.265 - 16.925) | 994 (804 - 1218) | 9.121 (7.39 - 11.161) | -2.167 (-2.6to-1.732) |
|  | Male | 175 (152 - 200) | 3.184 (2.775 - 3.636) | 131 (108 - 161) | 1.609 (1.33 - 1.959) | -2.64 (-2.988to-2.29) | 146 (127 - 166) | 2.77 (2.424 - 3.14) | 97 (80 - 118) | 1.243 (1.026 - 1.504) | -3.082 (-3.487to-2.676) | 4685 (4074 - 5332) | 80.15 (69.846 - 91.331) | 2895 (2376 - 3547) | 33.716 (27.718 - 41.149) | -3.355 (-3.792to-2.917) |
| Kyrgyzstan | Female | 4 (3 - 5) | 0.214 (0.167 - 0.271) | 14 (10 - 18) | 0.466 (0.347 - 0.61) | 1.66 (0.938-2.387) | 3 (2 - 4) | 0.175 (0.138 - 0.218) | 9 (7 - 12) | 0.313 (0.241 - 0.402) | 1.044 (0.29-1.805) | 91 (70 - 116) | 5.122 (3.911 - 6.557) | 289 (213 - 384) | 9.343 (6.962 - 12.335) | 0.997 (0.189-1.811) |
|  | Male | 16 (12 - 20) | 1.181 (0.933 - 1.45) | 27 (21 - 35) | 1.17 (0.907 - 1.477) | -0.533 (-0.992to-0.071) | 13 (10 - 17) | 1.044 (0.82 - 1.274) | 21 (16 - 27) | 0.967 (0.758 - 1.216) | -0.785 (-1.291to-0.275) | 434 (334 - 543) | 31.288 (24.357 - 38.687) | 654 (498 - 835) | 26.346 (20.335 - 33.567) | -1.049 (-1.534to-0.562) |
| Mongolia | Female | 4 (2 - 6) | 0.668 (0.35 - 0.964) | 5 (3 - 8) | 0.355 (0.232 - 0.541) | -2.729 (-3.27to-2.186) | 4 (2 - 5) | 0.609 (0.325 - 0.876) | 4 (2 - 6) | 0.278 (0.183 - 0.425) | -3.318 (-3.851to-2.782) | 116 (58 - 166) | 18.444 (9.352 - 26.279) | 122 (79 - 184) | 7.876 (5.152 - 11.945) | -3.506 (-4.024to-2.984) |
|  | Male | 7 (4 - 10) | 1.298 (0.834 - 1.986) | 12 (8 - 18) | 1.041 (0.663 - 1.528) | -0.941 (-1.17to-0.713) | 6 (4 - 10) | 1.276 (0.831 - 1.988) | 10 (6 - 15) | 0.913 (0.582 - 1.346) | -1.383 (-1.603to-1.163) | 185 (122 - 290) | 34.728 (22.703 - 54.481) | 325 (212 - 473) | 25.73 (16.598 - 37.822) | -1.213 (-1.416to-1.009) |
| Tajikistan | Female | 11 (6 - 17) | 0.681 (0.409 - 1.111) | 18 (10 - 28) | 0.52 (0.3 - 0.811) | -1.123 (-1.244to-1.003) | 9 (6 - 15) | 0.608 (0.372 - 1.014) | 14 (8 - 21) | 0.424 (0.24 - 0.657) | -1.399 (-1.576to-1.222) | 267 (159 - 434) | 16.528 (9.899 - 27.151) | 464 (255 - 744) | 12.298 (6.88 - 19.162) | -1.134 (-1.242to-1.025) |
|  | Male | 10 (6 - 15) | 0.776 (0.488 - 1.208) | 14 (9 - 21) | 0.453 (0.3 - 0.658) | -1.819 (-2.014to-1.624) | 9 (6 - 14) | 0.741 (0.465 - 1.159) | 12 (8 - 18) | 0.419 (0.283 - 0.6) | -1.9 (-2.095to-1.705) | 261 (161 - 410) | 19.572 (12.385 - 30.061) | 376 (234 - 556) | 11.183 (7.129 - 16.221) | -1.887 (-2.075to-1.698) |
| Turkmenistan | Female | 8 (7 - 9) | 0.702 (0.615 - 0.793) | 16 (12 - 22) | 0.668 (0.496 - 0.898) | -0.304 (-1.103-0.501) | 7 (6 - 8) | 0.582 (0.509 - 0.661) | 11 (8 - 15) | 0.478 (0.359 - 0.637) | -0.799 (-1.563to-0.03) | 222 (194 - 251) | 17.926 (15.73 - 20.334) | 369 (273 - 507) | 14.904 (11.081 - 20.379) | -0.711 (-1.469-0.052) |
|  | Male | 24 (21 - 27) | 2.717 (2.397 - 3.036) | 42 (31 - 57) | 2.095 (1.575 - 2.799) | -1.209 (-1.608to-0.808) | 21 (19 - 23) | 2.507 (2.211 - 2.794) | 35 (26 - 47) | 1.801 (1.368 - 2.425) | -1.444 (-1.819to-1.067) | 661 (579 - 738) | 70.348 (61.784 - 78.345) | 1079 (801 - 1477) | 50.352 (37.714 - 68.685) | -1.44 (-1.81to-1.069) |
| Uzbekistan | Female | 40 (30 - 54) | 0.586 (0.436 - 0.802) | 95 (70 - 122) | 0.595 (0.447 - 0.759) | 0.27 (-0.013-0.555) | 32 (24 - 45) | 0.471 (0.349 - 0.672) | 66 (49 - 84) | 0.432 (0.33 - 0.545) | -0.097 (-0.395-0.202) | 1017 (756 - 1331) | 14.429 (10.733 - 19.014) | 2176 (1561 - 2847) | 13.052 (9.553 - 16.889) | -0.146 (-0.433-0.143) |
|  | Male | 53 (38 - 76) | 1.032 (0.73 - 1.529) | 121 (89 - 156) | 0.928 (0.703 - 1.158) | -0.026 (-0.365-0.314) | 46 (33 - 67) | 0.938 (0.662 - 1.418) | 101 (73 - 130) | 0.809 (0.617 - 1.016) | -0.168 (-0.539-0.205) | 1409 (1003 - 1937) | 25.914 (18.461 - 36.898) | 3060 (2211 - 4005) | 21.747 (16.018 - 27.806) | -0.307 (-0.663-0.05) |
| Albania | Female | 7 (5 - 11) | 0.663 (0.482 - 0.986) | 19 (12 - 29) | 0.899 (0.573 - 1.365) | 1.457 (1.221-1.695) | 6 (4 - 8) | 0.515 (0.374 - 0.777) | 10 (6 - 15) | 0.455 (0.293 - 0.687) | -0.131 (-0.266-0.005) | 168 (119 - 243) | 14.609 (10.465 - 21.53) | 259 (165 - 391) | 12.494 (7.954 - 19.028) | -0.217 (-0.374to-0.06) |
|  | Male | 13 (9 - 18) | 1.15 (0.809 - 1.611) | 30 (18 - 48) | 1.546 (0.944 - 2.421) | 1.449 (1.282-1.616) | 11 (7 - 15) | 1.001 (0.711 - 1.4) | 20 (12 - 31) | 0.975 (0.608 - 1.514) | 0.245 (0.102-0.387) | 331 (228 - 472) | 28.083 (19.487 - 39.727) | 557 (335 - 884) | 28.834 (17.487 - 45.601) | 0.451 (0.3-0.602) |
| Bosnia and Herzegovina | Female | 14 (11 - 20) | 0.579 (0.456 - 0.811) | 31 (17 - 42) | 0.99 (0.552 - 1.35) | 2.495 (2.187-2.804) | 10 (8 - 14) | 0.412 (0.327 - 0.576) | 16 (9 - 22) | 0.491 (0.275 - 0.666) | 1.038 (0.857-1.219) | 295 (233 - 414) | 11.867 (9.433 - 16.578) | 413 (232 - 563) | 13.727 (7.682 - 18.561) | 1.009 (0.789-1.23) |
|  | Male | 43 (36 - 52) | 1.99 (1.694 - 2.354) | 95 (71 - 125) | 3.52 (2.629 - 4.619) | 2.495 (2.173-2.818) | 33 (28 - 40) | 1.566 (1.327 - 1.862) | 61 (46 - 79) | 2.207 (1.652 - 2.881) | 1.588 (1.331-1.846) | 1101 (918 - 1324) | 48.807 (41.404 - 57.854) | 1757 (1305 - 2310) | 65.573 (48.731 - 86.834) | 1.435 (1.173-1.697) |
| Bulgaria | Female | 21 (19 - 24) | 0.341 (0.306 - 0.384) | 56 (47 - 65) | 0.875 (0.726 - 1.03) | 2.815 (2.599-3.033) | 14 (13 - 16) | 0.23 (0.207 - 0.257) | 32 (27 - 37) | 0.458 (0.386 - 0.533) | 2.013 (1.784-2.241) | 413 (368 - 461) | 6.672 (5.941 - 7.459) | 840 (702 - 983) | 13.91 (11.53 - 16.353) | 2.171 (1.95-2.393) |
|  | Male | 155 (138 - 174) | 2.664 (2.359 - 2.992) | 300 (250 - 353) | 5.443 (4.528 - 6.397) | 2.296 (2.079-2.514) | 88 (79 - 99) | 1.518 (1.353 - 1.691) | 140 (118 - 163) | 2.468 (2.073 - 2.859) | 1.643 (1.42-1.866) | 2813 (2500 - 3137) | 48.602 (43.187 - 54.157) | 4185 (3519 - 4857) | 76.606 (64.4 - 88.992) | 1.48 (1.243-1.717) |
| Croatia | Female | 19 (17 - 21) | 0.527 (0.468 - 0.592) | 48 (39 - 59) | 1.163 (0.943 - 1.411) | 2.994 (2.436-3.554) | 10 (9 - 11) | 0.287 (0.256 - 0.32) | 19 (16 - 23) | 0.408 (0.333 - 0.49) | 1.457 (0.93-1.987) | 281 (249 - 313) | 8.005 (7.181 - 8.933) | 460 (377 - 557) | 11.378 (9.345 - 13.692) | 1.453 (0.902-2.008) |
|  | Male | 194 (170 - 218) | 6.711 (5.99 - 7.525) | 258 (216 - 304) | 7.299 (6.091 - 8.579) | 0.471 (0.161-0.782) | 134 (118 - 151) | 4.759 (4.252 - 5.308) | 144 (121 - 171) | 3.958 (3.324 - 4.697) | -0.439 (-0.725to-0.152) | 4323 (3789 - 4852) | 145.958 (129.428 - 163.193) | 3961 (3328 - 4665) | 114.134 (94.908 - 134.729) | -0.642 (-0.956to-0.328) |
| Czechia | Female | 45 (39 - 51) | 0.598 (0.525 - 0.691) | 207 (165 - 254) | 2.119 (1.688 - 2.605) | 4.328 (3.996-4.66) | 22 (20 - 26) | 0.285 (0.25 - 0.325) | 59 (48 - 72) | 0.543 (0.438 - 0.662) | 2.39 (1.932-2.849) | 583 (512 - 668) | 7.983 (7.014 - 9.157) | 1499 (1203 - 1824) | 15.736 (12.683 - 19.18) | 2.55 (2.097-3.005) |
|  | Male | 198 (172 - 226) | 3.382 (2.935 - 3.864) | 508 (398 - 639) | 5.749 (4.489 - 7.199) | 1.937 (1.699-2.176) | 149 (129 - 170) | 2.57 (2.226 - 2.931) | 281 (225 - 350) | 3.117 (2.479 - 3.89) | 0.841 (0.693-0.989) | 4651 (4039 - 5299) | 78.98 (68.515 - 90.052) | 7768 (6050 - 9788) | 89.998 (69.941 - 113.366) | 0.625 (0.446-0.805) |
| Hungary | Female | 67 (58 - 76) | 0.87 (0.765 - 0.984) | 220 (181 - 258) | 2.386 (1.97 - 2.814) | 2.636 (2.141-3.134) | 43 (38 - 49) | 0.547 (0.478 - 0.618) | 99 (81 - 116) | 0.97 (0.804 - 1.143) | 1.438 (1.128-1.748) | 1258 (1111 - 1416) | 16.929 (14.988 - 19.03) | 2539 (2111 - 3008) | 28.456 (23.541 - 33.96) | 1.085 (0.689-1.484) |
|  | Male | 469 (408 - 537) | 7.604 (6.663 - 8.657) | 797 (635 - 968) | 10.65 (8.472 - 12.913) | 0.194 (-0.444-0.836) | 333 (287 - 379) | 5.386 (4.676 - 6.118) | 447 (355 - 546) | 5.877 (4.688 - 7.156) | -0.445 (-0.955-0.067) | 11215 (9806 - 12702) | 181.961 (160.025 - 206.135) | 13058 (10496 - 15788) | 176.855 (142.576 - 213.779) | -0.94 (-1.531to-0.346) |
| Montenegro | Female | 1 (1 - 1) | 0.258 (0.183 - 0.36) | 2 (1 - 2) | 0.305 (0.2 - 0.411) | 0.98 (0.88-1.08) | 0 (0 - 1) | 0.126 (0.089 - 0.177) | 1 (0 - 1) | 0.126 (0.087 - 0.166) | 0.166 (0.064-0.267) | 13 (9 - 19) | 3.827 (2.693 - 5.302) | 17 (12 - 23) | 3.561 (2.383 - 4.755) | 0.003 (-0.138-0.145) |
|  | Male | 7 (6 - 9) | 2.388 (1.917 - 2.899) | 12 (9 - 16) | 2.73 (2.006 - 3.712) | 0.719 (0.508-0.931) | 4 (4 - 5) | 1.443 (1.16 - 1.745) | 7 (5 - 9) | 1.489 (1.098 - 1.996) | 0.199 (0.07-0.328) | 142 (115 - 171) | 45.434 (36.629 - 54.79) | 199 (146 - 272) | 44.872 (32.517 - 61.696) | 0.078 (-0.041-0.198) |
| North Macedonia | Female | 3 (3 - 5) | 0.332 (0.261 - 0.504) | 9 (5 - 12) | 0.507 (0.318 - 0.693) | 1.645 (1.34-1.951) | 2 (2 - 4) | 0.24 (0.188 - 0.359) | 4 (3 - 6) | 0.268 (0.174 - 0.361) | 0.446 (0.209-0.684) | 73 (57 - 110) | 7.018 (5.49 - 10.563) | 121 (77 - 165) | 7.263 (4.641 - 9.971) | 0.231 (0.014-0.449) |
|  | Male | 14 (12 - 17) | 1.432 (1.197 - 1.753) | 31 (24 - 42) | 1.869 (1.422 - 2.504) | 1.18 (0.888-1.474) | 11 (9 - 14) | 1.152 (0.963 - 1.415) | 20 (15 - 28) | 1.215 (0.929 - 1.636) | 0.381 (0.132-0.631) | 353 (299 - 433) | 35.365 (29.89 - 43.204) | 602 (452 - 828) | 35.713 (26.986 - 48.877) | 0.208 (-0.042-0.458) |
| Poland | Female | 103 (98 - 109) | 0.417 (0.395 - 0.441) | 465 (410 - 523) | 1.303 (1.145 - 1.469) | 4.083 (3.656-4.513) | 77 (73 - 81) | 0.306 (0.289 - 0.322) | 235 (207 - 260) | 0.602 (0.528 - 0.667) | 2.468 (1.97-2.969) | 1996 (1900 - 2098) | 8.296 (7.904 - 8.726) | 5781 (5074 - 6435) | 16.66 (14.631 - 18.559) | 2.59 (2.131-3.051) |
|  | Male | 605 (575 - 637) | 3.169 (3.007 - 3.328) | 1847 (1608 - 2068) | 6.2 (5.407 - 6.93) | 2.539 (2.244-2.835) | 472 (448 - 496) | 2.509 (2.384 - 2.629) | 1055 (933 - 1181) | 3.506 (3.109 - 3.918) | 1.415 (1.061-1.771) | 14941 (14200 - 15665) | 77.227 (73.472 - 80.865) | 29834 (26355 - 33529) | 101.857 (90.155 - 114.427) | 1.191 (0.874-1.509) |
| Romania | Female | 57 (49 - 65) | 0.378 (0.331 - 0.43) | 174 (137 - 211) | 1.014 (0.803 - 1.226) | 3.071 (2.783-3.36) | 42 (36 - 48) | 0.279 (0.244 - 0.318) | 90 (72 - 109) | 0.479 (0.385 - 0.576) | 1.506 (1.243-1.77) | 1227 (1071 - 1400) | 8.303 (7.272 - 9.441) | 2361 (1883 - 2867) | 14.336 (11.406 - 17.481) | 1.505 (1.228-1.783) |
|  | Male | 386 (337 - 442) | 2.906 (2.564 - 3.315) | 1400 (1136 - 1675) | 9.412 (7.653 - 11.244) | 3.985 (3.708-4.262) | 306 (268 - 350) | 2.324 (2.044 - 2.633) | 865 (703 - 1040) | 5.739 (4.677 - 6.868) | 3.018 (2.772-3.264) | 10118 (8864 - 11539) | 75.734 (66.846 - 85.928) | 26338 (21522 - 31443) | 179.334 (147.011 - 213.722) | 2.848 (2.56-3.137) |
| Serbia | Female | 40 (27 - 58) | 0.663 (0.449 - 0.945) | 72 (46 - 105) | 0.905 (0.58 - 1.325) | 1.169 (1.069-1.269) | 28 (19 - 40) | 0.47 (0.316 - 0.67) | 35 (22 - 51) | 0.406 (0.26 - 0.592) | -0.499 (-0.574to-0.425) | 788 (527 - 1137) | 12.761 (8.615 - 18.299) | 870 (555 - 1274) | 11.34 (7.315 - 16.575) | -0.349 (-0.424to-0.273) |
|  | Male | 200 (154 - 256) | 3.481 (2.718 - 4.403) | 290 (222 - 373) | 4.176 (3.18 - 5.352) | 0.744 (0.612-0.875) | 161 (125 - 207) | 2.872 (2.234 - 3.625) | 184 (141 - 237) | 2.617 (2.01 - 3.327) | -0.206 (-0.33to-0.082) | 5077 (3904 - 6504) | 86.328 (66.96 - 109.712) | 5256 (3986 - 6763) | 77.66 (58.599 - 99.307) | -0.316 (-0.464to-0.167) |
| Slovakia | Female | 18 (13 - 26) | 0.558 (0.412 - 0.798) | 43 (26 - 62) | 0.911 (0.537 - 1.302) | 1.77 (1.587-1.954) | 12 (9 - 17) | 0.353 (0.261 - 0.509) | 20 (12 - 29) | 0.398 (0.242 - 0.572) | 0.514 (0.35-0.678) | 328 (243 - 481) | 10.258 (7.571 - 15.12) | 530 (322 - 770) | 11.392 (6.896 - 16.642) | 0.486 (0.314-0.657) |
|  | Male | 236 (193 - 298) | 9.071 (7.411 - 11.432) | 446 (333 - 598) | 10.912 (8.177 - 14.537) | 0.617 (0.415-0.82) | 162 (132 - 203) | 6.23 (5.097 - 7.778) | 253 (187 - 339) | 6.135 (4.557 - 8.133) | -0.063 (-0.275-0.15) | 5350 (4355 - 6749) | 204.656 (166.746 - 257.835) | 7628 (5603 - 10169) | 186.728 (137.803 - 247.601) | -0.378 (-0.595to-0.161) |
| Slovenia | Female | 10 (8 - 11) | 0.702 (0.611 - 0.822) | 38 (29 - 48) | 1.905 (1.441 - 2.392) | 3.212 (2.741-3.685) | 5 (5 - 6) | 0.363 (0.318 - 0.416) | 13 (10 - 16) | 0.551 (0.429 - 0.689) | 1.008 (0.491-1.528) | 141 (122 - 162) | 10.333 (8.945 - 11.973) | 303 (232 - 379) | 15.381 (11.66 - 19.513) | 0.947 (0.422-1.475) |
|  | Male | 79 (67 - 91) | 7.267 (6.233 - 8.345) | 146 (109 - 190) | 7.959 (5.957 - 10.398) | 0.507 (0.289-0.726) | 59 (51 - 68) | 5.535 (4.788 - 6.359) | 82 (61 - 106) | 4.386 (3.263 - 5.668) | -0.702 (-0.896to-0.508) | 1882 (1616 - 2158) | 170.443 (147.325 - 195.134) | 2257 (1659 - 2941) | 126.54 (92.697 - 165.073) | -0.948 (-1.143to-0.753) |
| Belarus | Female | 13 (11 - 16) | 0.167 (0.137 - 0.205) | 47 (36 - 61) | 0.548 (0.41 - 0.727) | 4.229 (3.99-4.469) | 9 (7 - 11) | 0.111 (0.092 - 0.134) | 22 (17 - 28) | 0.232 (0.181 - 0.301) | 2.57 (2.328-2.812) | 237 (194 - 288) | 3.09 (2.528 - 3.754) | 568 (437 - 752) | 6.667 (5.027 - 8.974) | 2.69 (2.434-2.946) |
|  | Male | 160 (134 - 190) | 3.01 (2.549 - 3.56) | 742 (570 - 953) | 11.28 (8.698 - 14.44) | 3.992 (3.552-4.433) | 124 (104 - 148) | 2.375 (2.004 - 2.811) | 417 (322 - 539) | 6.32 (4.899 - 8.158) | 2.778 (2.293-3.265) | 3983 (3350 - 4731) | 73.157 (61.951 - 86.294) | 12820 (9900 - 16546) | 193.944 (150.131 - 249.818) | 2.665 (2.16-3.173) |
| Estonia | Female | 5 (4 - 6) | 0.408 (0.334 - 0.492) | 10 (8 - 12) | 0.778 (0.603 - 0.963) | 1.769 (1.362-2.177) | 3 (3 - 4) | 0.266 (0.22 - 0.321) | 5 (4 - 6) | 0.313 (0.247 - 0.383) | -0.029 (-0.461-0.405) | 88 (72 - 106) | 7.28 (5.991 - 8.805) | 109 (86 - 134) | 8.438 (6.651 - 10.405) | -0.174 (-0.594-0.248) |
|  | Male | 38 (33 - 44) | 4.617 (4.009 - 5.341) | 65 (53 - 77) | 6.587 (5.361 - 7.807) | 1.193 (0.914-1.474) | 29 (25 - 34) | 3.615 (3.13 - 4.17) | 35 (29 - 42) | 3.556 (2.904 - 4.213) | -0.205 (-0.422-0.011) | 912 (795 - 1052) | 108.546 (94.382 - 125.169) | 954 (783 - 1131) | 98.449 (80.76 - 116.264) | -0.553 (-0.779to-0.327) |
| Latvia | Female | 8 (6 - 9) | 0.356 (0.298 - 0.425) | 14 (11 - 18) | 0.738 (0.576 - 0.928) | 2.167 (1.751-2.585) | 5 (4 - 6) | 0.232 (0.194 - 0.277) | 8 (6 - 10) | 0.347 (0.274 - 0.435) | 1.178 (0.84-1.516) | 136 (114 - 163) | 6.482 (5.428 - 7.777) | 185 (146 - 231) | 9.791 (7.669 - 12.245) | 1.11 (0.79-1.432) |
|  | Male | 55 (46 - 64) | 3.835 (3.209 - 4.501) | 92 (74 - 118) | 6.623 (5.29 - 8.44) | 1.814 (1.387-2.242) | 42 (36 - 50) | 3.03 (2.556 - 3.567) | 57 (46 - 73) | 4.049 (3.264 - 5.166) | 1.025 (0.697-1.354) | 1328 (1118 - 1566) | 91.297 (76.957 - 107.446) | 1652 (1329 - 2118) | 120.058 (96.753 - 153.613) | 0.832 (0.508-1.157) |
| Lithuania | Female | 8 (7 - 10) | 0.314 (0.259 - 0.375) | 18 (14 - 22) | 0.637 (0.491 - 0.784) | 2.184 (1.847-2.523) | 5 (4 - 6) | 0.197 (0.164 - 0.236) | 10 (8 - 13) | 0.307 (0.24 - 0.377) | 1.482 (1.136-1.829) | 144 (119 - 172) | 5.531 (4.571 - 6.599) | 238 (185 - 293) | 8.611 (6.629 - 10.594) | 1.405 (1.071-1.74) |
|  | Male | 66 (56 - 78) | 3.549 (2.987 - 4.149) | 142 (112 - 169) | 6.892 (5.444 - 8.121) | 2.35 (2.217-2.483) | 58 (49 - 67) | 3.12 (2.623 - 3.639) | 115 (90 - 136) | 5.488 (4.326 - 6.469) | 2.123 (1.979-2.268) | 1808 (1524 - 2108) | 95.159 (80.361 - 110.615) | 3322 (2613 - 3941) | 162.82 (128.356 - 191.919) | 1.886 (1.74-2.031) |
| Republic of Moldova | Female | 8 (7 - 9) | 0.305 (0.276 - 0.341) | 14 (12 - 16) | 0.434 (0.371 - 0.5) | 1.326 (0.82-1.834) | 6 (5 - 6) | 0.221 (0.201 - 0.247) | 8 (7 - 9) | 0.235 (0.202 - 0.27) | 0.434 (0.013-0.857) | 168 (153 - 188) | 6.441 (5.862 - 7.224) | 213 (184 - 246) | 6.709 (5.796 - 7.745) | 0.361 (-0.032-0.756) |
|  | Male | 98 (87 - 110) | 4.946 (4.429 - 5.566) | 224 (194 - 263) | 8.873 (7.678 - 10.377) | 2.259 (1.837-2.683) | 82 (73 - 92) | 4.216 (3.785 - 4.724) | 152 (133 - 178) | 6.01 (5.244 - 6.982) | 1.539 (1.175-1.904) | 2581 (2298 - 2907) | 127.569 (113.826 - 143.343) | 4583 (3986 - 5363) | 181.143 (157.537 - 211.498) | 1.497 (1.151-1.845) |
| Russian Federation | Female | 346 (332 - 362) | 0.312 (0.3 - 0.327) | 972 (859 - 1082) | 0.79 (0.697 - 0.885) | 2.984 (2.592-3.377) | 249 (237 - 260) | 0.217 (0.207 - 0.227) | 454 (403 - 502) | 0.334 (0.297 - 0.37) | 1.099 (0.892-1.305) | 6490 (6227 - 6792) | 5.966 (5.725 - 6.235) | 12701 (11250 - 14123) | 10.364 (9.163 - 11.601) | 1.561 (1.343-1.778) |
|  | Male | 3712 (3584 - 3844) | 4.947 (4.785 - 5.124) | 7265 (6308 - 8102) | 7.491 (6.516 - 8.336) | 1.146 (0.828-1.465) | 2151 (2075 - 2230) | 3.014 (2.917 - 3.124) | 3008 (2628 - 3371) | 3.1 (2.716 - 3.468) | -0.252 (-0.47to-0.033) | 69893 (67433 - 72443) | 92.111 (89.059 - 95.444) | 91522 (79888 - 102661) | 93.996 (82.171 - 105.207) | -0.332 (-0.567to-0.098) |
| Ukraine | Female | 170 (141 - 213) | 0.428 (0.353 - 0.543) | 331 (215 - 499) | 0.883 (0.559 - 1.351) | 2.525 (2.091-2.961) | 71 (60 - 88) | 0.165 (0.14 - 0.206) | 108 (73 - 161) | 0.256 (0.168 - 0.389) | 1.36 (1.02-1.701) | 2057 (1719 - 2610) | 5.21 (4.36 - 6.623) | 3184 (2050 - 4857) | 8.505 (5.365 - 13.179) | 1.539 (1.176-1.904) |
|  | Male | 1119 (850 - 1569) | 3.79 (2.897 - 5.296) | 2007 (1259 - 2948) | 6.563 (4.113 - 9.577) | 1.898 (1.659-2.136) | 789 (600 - 1093) | 2.705 (2.069 - 3.735) | 1262 (781 - 1854) | 4.112 (2.569 - 5.993) | 1.391 (1.195-1.587) | 25122 (19101 - 35244) | 83.628 (63.851 - 117.331) | 39370 (24257 - 57837) | 129.576 (79.862 - 189.083) | 1.392 (1.192-1.593) |
| Australia | Female | 117 (105 - 128) | 1.147 (1.034 - 1.261) | 287 (244 - 330) | 1.343 (1.163 - 1.537) | 0.439 (0.199-0.679) | 34 (31 - 37) | 0.318 (0.287 - 0.347) | 58 (48 - 67) | 0.243 (0.206 - 0.278) | -0.943 (-1.299to-0.586) | 859 (778 - 935) | 8.516 (7.715 - 9.279) | 1389 (1197 - 1584) | 6.527 (5.696 - 7.377) | -0.919 (-1.245to-0.591) |
|  | Male | 472 (423 - 523) | 5.199 (4.677 - 5.758) | 1315 (1111 - 1527) | 6.781 (5.725 - 7.888) | 0.825 (0.427-1.225) | 136 (123 - 150) | 1.518 (1.376 - 1.679) | 260 (223 - 301) | 1.272 (1.089 - 1.473) | -0.718 (-1.203to-0.23) | 3884 (3503 - 4306) | 42.78 (38.501 - 47.5) | 6928 (5921 - 7982) | 36.074 (30.938 - 41.564) | -0.62 (-1.092to-0.146) |
| New Zealand | Female | 17 (15 - 20) | 0.829 (0.716 - 0.956) | 41 (34 - 48) | 0.985 (0.821 - 1.162) | 1.025 (0.345-1.709) | 6 (5 - 7) | 0.264 (0.229 - 0.3) | 9 (8 - 11) | 0.205 (0.171 - 0.242) | -0.38 (-1.135-0.381) | 138 (119 - 158) | 6.745 (5.841 - 7.736) | 219 (182 - 259) | 5.294 (4.468 - 6.25) | -0.419 (-1.119-0.287) |
|  | Male | 31 (27 - 36) | 1.737 (1.499 - 2.017) | 73 (61 - 85) | 1.916 (1.605 - 2.251) | 0.613 (0.222-1.005) | 18 (15 - 21) | 0.994 (0.856 - 1.157) | 29 (24 - 34) | 0.74 (0.626 - 0.865) | -0.751 (-1.103to-0.398) | 467 (403 - 541) | 25.97 (22.489 - 30.071) | 733 (620 - 858) | 19.575 (16.613 - 22.904) | -0.692 (-1.06to-0.322) |
| Brunei Darussalam | Female | 0 (0 - 1) | 0.792 (0.582 - 1.118) | 2 (1 - 2) | 0.886 (0.614 - 1.168) | 0.839 (0.716-0.963) | 0 (0 - 0) | 0.605 (0.444 - 0.852) | 1 (1 - 1) | 0.549 (0.378 - 0.718) | 0.232 (0.081-0.384) | 9 (7 - 14) | 16.164 (11.931 - 23.081) | 30 (21 - 40) | 14.512 (10.184 - 19.21) | 0.176 (0.025-0.326) |
|  | Male | 1 (1 - 2) | 2.295 (1.676 - 3.094) | 5 (4 - 6) | 2.605 (2.039 - 3.272) | 1.211 (0.932-1.49) | 1 (1 - 1) | 1.956 (1.428 - 2.639) | 3 (3 - 4) | 1.899 (1.499 - 2.388) | 0.767 (0.458-1.077) | 28 (21 - 38) | 49.464 (35.725 - 66.358) | 95 (74 - 119) | 47.546 (37.326 - 59.578) | 0.642 (0.351-0.933) |
| Japan | Female | 312 (290 - 332) | 0.335 (0.314 - 0.357) | 1093 (920 - 1208) | 0.744 (0.662 - 0.808) | 2.628 (2.321-2.935) | 121 (112 - 128) | 0.128 (0.118 - 0.135) | 382 (301 - 430) | 0.191 (0.163 - 0.207) | 1.304 (1.103-1.504) | 3077 (2894 - 3211) | 3.338 (3.149 - 3.478) | 7390 (6271 - 8025) | 5.124 (4.644 - 5.44) | 1.412 (1.179-1.646) |
|  | Male | 1386 (1323 - 1448) | 1.756 (1.677 - 1.835) | 7168 (6619 - 7628) | 4.922 (4.591 - 5.214) | 3.22 (2.801-3.641) | 628 (607 - 647) | 0.823 (0.794 - 0.848) | 2906 (2679 - 3044) | 1.795 (1.677 - 1.872) | 2.358 (2.038-2.68) | 17255 (16740 - 17754) | 21.614 (20.933 - 22.233) | 58616 (54770 - 61109) | 41.464 (39.342 - 43.103) | 1.925 (1.558-2.294) |
| Republic of Korea | Female | 31 (23 - 39) | 0.175 (0.13 - 0.218) | 131 (84 - 179) | 0.271 (0.177 - 0.371) | 1.747 (1.619-1.875) | 20 (15 - 25) | 0.12 (0.089 - 0.151) | 39 (24 - 52) | 0.075 (0.049 - 0.101) | -1.485 (-1.625to-1.344) | 568 (428 - 721) | 3.082 (2.323 - 3.872) | 923 (602 - 1252) | 1.951 (1.29 - 2.636) | -1.417 (-1.569to-1.266) |
|  | Male | 135 (108 - 164) | 0.986 (0.803 - 1.201) | 1177 (880 - 1476) | 2.65 (1.996 - 3.293) | 3.985 (3.435-4.538) | 95 (76 - 116) | 0.754 (0.614 - 0.916) | 424 (326 - 530) | 0.992 (0.768 - 1.228) | 1.324 (0.915-1.734) | 2858 (2297 - 3459) | 19.458 (15.734 - 23.749) | 10351 (7762 - 13201) | 23.133 (17.52 - 29.093) | 0.987 (0.598-1.378) |
| Singapore | Female | 6 (5 - 6) | 0.436 (0.378 - 0.498) | 22 (19 - 26) | 0.509 (0.434 - 0.591) | 1.138 (0.684-1.593) | 3 (2 - 3) | 0.236 (0.205 - 0.266) | 6 (6 - 8) | 0.146 (0.125 - 0.169) | -1.138 (-1.49to-0.785) | 83 (72 - 94) | 6.34 (5.492 - 7.249) | 162 (139 - 189) | 3.744 (3.238 - 4.351) | -1.182 (-1.569to-0.793) |
|  | Male | 19 (16 - 22) | 1.744 (1.493 - 2.009) | 112 (96 - 130) | 2.542 (2.19 - 2.943) | 1.62 (1.402-1.84) | 12 (10 - 13) | 1.115 (0.955 - 1.278) | 41 (36 - 48) | 0.984 (0.858 - 1.13) | -0.197 (-0.379to-0.014) | 335 (282 - 390) | 29.615 (25.171 - 34.411) | 1025 (883 - 1196) | 23.116 (20.034 - 26.957) | -0.553 (-0.731to-0.375) |
| Canada | Female | 136 (120 - 155) | 0.809 (0.708 - 0.917) | 369 (317 - 429) | 1.049 (0.902 - 1.205) | 1.052 (0.894-1.21) | 46 (41 - 52) | 0.257 (0.229 - 0.291) | 98 (84 - 114) | 0.252 (0.217 - 0.291) | 0.117 (-0.053-0.286) | 1180 (1046 - 1323) | 7.042 (6.241 - 7.89) | 2273 (1956 - 2592) | 6.512 (5.657 - 7.439) | -0.089 (-0.264-0.087) |
|  | Male | 411 (364 - 460) | 2.806 (2.49 - 3.139) | 1139 (975 - 1301) | 3.598 (3.109 - 4.099) | 1.116 (0.89-1.343) | 161 (145 - 178) | 1.115 (1.004 - 1.229) | 353 (308 - 403) | 1.066 (0.933 - 1.215) | 0.082 (-0.204-0.369) | 4539 (4049 - 5043) | 30.931 (27.576 - 34.276) | 8937 (7751 - 10219) | 28.462 (24.786 - 32.432) | -0.02 (-0.319-0.28) |
| Greenland | Female | 0 (0 - 1) | 2.51 (1.691 - 3.145) | 0 (0 - 1) | 0.933 (0.632 - 2.232) | -3.36 (-3.677to-3.042) | 0 (0 - 0) | 1.834 (1.273 - 2.244) | 0 (0 - 0) | 0.512 (0.36 - 1.135) | -4.342 (-4.693to-3.989) | 10 (6 - 12) | 50.338 (33.122 - 63.162) | 5 (3 - 13) | 14.051 (9.448 - 33.807) | -4.313 (-4.619to-4.007) |
|  | Male | 2 (1 - 2) | 8.744 (7.149 - 10.512) | 3 (2 - 4) | 6.342 (4.866 - 8.023) | -0.65 (-0.802to-0.497) | 1 (1 - 2) | 6.837 (5.611 - 8.176) | 2 (1 - 2) | 4.016 (3.136 - 5.115) | -1.413 (-1.562to-1.263) | 46 (37 - 57) | 199.811 (162.759 - 240.352) | 50 (38 - 64) | 112.809 (86.95 - 143.473) | -1.532 (-1.679to-1.384) |
| United States of America | Female | 1756 (1655 - 1819) | 1.061 (1.012 - 1.095) | 3037 (2793 - 3183) | 1.046 (0.974 - 1.093) | 0.002 (-0.178-0.183) | 572 (530 - 596) | 0.323 (0.303 - 0.334) | 799 (717 - 848) | 0.254 (0.231 - 0.268) | -0.753 (-0.996to-0.51) | 14306 (13576 - 14825) | 8.791 (8.429 - 9.077) | 19318 (18000 - 20407) | 6.765 (6.407 - 7.121) | -0.758 (-1.021to-0.495) |
|  | Male | 5623 (5477 - 5753) | 4.253 (4.142 - 4.351) | 13159 (12638 - 13680) | 5.092 (4.894 - 5.294) | 0.78 (0.614-0.947) | 1449 (1402 - 1482) | 1.09 (1.053 - 1.115) | 2740 (2610 - 2856) | 1.032 (0.983 - 1.076) | 0.003 (-0.281-0.289) | 41647 (40442 - 42837) | 31.615 (30.679 - 32.539) | 73987 (70955 - 77239) | 28.884 (27.716 - 30.182) | -0.086 (-0.338-0.167) |
| Argentina | Female | 55 (48 - 63) | 0.31 (0.27 - 0.357) | 77 (61 - 94) | 0.256 (0.206 - 0.312) | -0.037 (-0.597-0.525) | 42 (37 - 49) | 0.239 (0.207 - 0.278) | 49 (38 - 60) | 0.154 (0.122 - 0.19) | -0.748 (-1.342to-0.15) | 1069 (934 - 1229) | 6.118 (5.356 - 7) | 1149 (924 - 1412) | 3.933 (3.175 - 4.817) | -0.762 (-1.337to-0.183) |
|  | Male | 350 (304 - 398) | 2.341 (2.037 - 2.663) | 256 (212 - 304) | 1.037 (0.859 - 1.234) | -2.225 (-2.604to-1.845) | 273 (240 - 309) | 1.858 (1.638 - 2.104) | 169 (141 - 198) | 0.686 (0.571 - 0.81) | -2.707 (-3.106to-2.307) | 8078 (7044 - 9240) | 53.327 (46.532 - 60.976) | 4629 (3837 - 5520) | 18.755 (15.526 - 22.43) | -2.934 (-3.322to-2.543) |
| Chile | Female | 20 (18 - 23) | 0.363 (0.324 - 0.41) | 40 (34 - 47) | 0.293 (0.251 - 0.34) | -0.064 (-0.684-0.559) | 15 (13 - 16) | 0.271 (0.241 - 0.304) | 19 (17 - 23) | 0.135 (0.116 - 0.157) | -1.617 (-2.226to-1.005) | 380 (337 - 430) | 6.776 (6.021 - 7.67) | 452 (394 - 522) | 3.324 (2.905 - 3.844) | -1.702 (-2.334to-1.066) |
|  | Male | 76 (67 - 88) | 1.59 (1.392 - 1.827) | 110 (94 - 128) | 0.946 (0.802 - 1.097) | -1.279 (-1.614to-0.943) | 57 (50 - 66) | 1.223 (1.075 - 1.407) | 58 (49 - 66) | 0.496 (0.423 - 0.572) | -2.561 (-2.888to-2.234) | 1713 (1489 - 1988) | 34.785 (30.226 - 40.314) | 1529 (1310 - 1769) | 13.083 (11.156 - 15.131) | -2.771 (-3.115to-2.425) |
| Uruguay | Female | 10 (8 - 12) | 0.473 (0.379 - 0.575) | 12 (10 - 15) | 0.413 (0.329 - 0.513) | -0.108 (-0.617-0.404) | 7 (6 - 9) | 0.331 (0.27 - 0.401) | 7 (6 - 9) | 0.224 (0.179 - 0.276) | -0.897 (-1.435to-0.355) | 172 (138 - 208) | 8.452 (6.81 - 10.208) | 157 (126 - 194) | 5.676 (4.563 - 7.016) | -0.896 (-1.434to-0.354) |
|  | Male | 75 (64 - 86) | 4.39 (3.773 - 5.033) | 61 (50 - 73) | 2.769 (2.284 - 3.329) | -1.476 (-1.692to-1.258) | 55 (47 - 63) | 3.247 (2.784 - 3.721) | 38 (31 - 45) | 1.676 (1.391 - 1.999) | -2.069 (-2.301to-1.836) | 1559 (1327 - 1791) | 91.497 (78.271 - 105.249) | 986 (815 - 1181) | 45.416 (37.432 - 54.379) | -2.224 (-2.452to-1.996) |
| Andorra | Female | 0 (0 - 0) | 0.074 (0.045 - 0.111) | 0 (0 - 0) | 0.074 (0.042 - 0.111) | 0.28 (0.002-0.559) | 0 (0 - 0) | 0.025 (0.016 - 0.039) | 0 (0 - 0) | 0.017 (0.01 - 0.025) | -0.93 (-1.093to-0.766) | 0 (0 - 0) | 0.741 (0.458 - 1.134) | 0 (0 - 1) | 0.503 (0.291 - 0.784) | -0.898 (-1.063to-0.732) |
|  | Male | 1 (1 - 2) | 3.329 (2.15 - 5.008) | 2 (1 - 3) | 2.979 (1.882 - 4.398) | 0.047 (-0.208-0.302) | 0 (0 - 1) | 1.469 (0.946 - 2.2) | 1 (0 - 1) | 0.898 (0.565 - 1.33) | -1.166 (-1.385to-0.947) | 14 (9 - 21) | 44.218 (28.724 - 66.511) | 21 (13 - 32) | 27.085 (16.821 - 40.099) | -1.15 (-1.35to-0.95) |
| Austria | Female | 47 (42 - 51) | 0.774 (0.693 - 0.853) | 141 (119 - 163) | 1.699 (1.453 - 1.948) | 2.656 (2.423-2.89) | 21 (19 - 23) | 0.318 (0.288 - 0.349) | 41 (35 - 47) | 0.438 (0.378 - 0.5) | 1.186 (1.004-1.368) | 531 (482 - 583) | 9.041 (8.166 - 9.946) | 1000 (866 - 1146) | 12.192 (10.566 - 13.923) | 1.091 (0.917-1.266) |
|  | Male | 205 (185 - 225) | 4.492 (4.048 - 4.94) | 459 (393 - 532) | 6.153 (5.284 - 7.119) | 1.263 (1.042-1.486) | 111 (101 - 121) | 2.426 (2.213 - 2.657) | 177 (153 - 203) | 2.299 (1.986 - 2.636) | 0.052 (-0.081-0.185) | 3430 (3108 - 3780) | 75.237 (68.007 - 82.94) | 4753 (4094 - 5432) | 64.329 (55.512 - 73.336) | -0.29 (-0.436to-0.144) |
| Belgium | Female | 58 (49 - 68) | 0.773 (0.647 - 0.925) | 160 (128 - 198) | 1.593 (1.258 - 1.99) | 2.361 (2.064-2.659) | 25 (22 - 30) | 0.306 (0.263 - 0.364) | 47 (38 - 57) | 0.413 (0.333 - 0.505) | 1.012 (0.792-1.233) | 646 (553 - 773) | 8.858 (7.502 - 10.608) | 1182 (953 - 1459) | 11.9 (9.556 - 14.754) | 0.969 (0.725-1.212) |
|  | Male | 235 (204 - 275) | 3.71 (3.214 - 4.319) | 457 (371 - 554) | 4.905 (3.989 - 5.955) | 0.783 (0.464-1.103) | 117 (101 - 135) | 1.826 (1.585 - 2.119) | 163 (133 - 196) | 1.665 (1.362 - 2.006) | -0.364 (-0.624to-0.102) | 3489 (3012 - 4078) | 55.207 (47.626 - 64.358) | 4473 (3624 - 5425) | 48.535 (39.637 - 58.739) | -0.577 (-0.863to-0.291) |
| Cyprus | Female | 1 (0 - 1) | 0.173 (0.113 - 0.268) | 3 (2 - 4) | 0.301 (0.193 - 0.435) | 2.763 (2.38-3.147) | 0 (0 - 1) | 0.097 (0.065 - 0.145) | 1 (1 - 1) | 0.083 (0.053 - 0.116) | -0.012 (-0.235-0.212) | 11 (7 - 16) | 2.47 (1.625 - 3.853) | 23 (15 - 32) | 2.225 (1.44 - 3.17) | 0.252 (0.009-0.497) |
|  | Male | 2 (2 - 3) | 0.575 (0.438 - 0.724) | 8 (6 - 11) | 0.866 (0.617 - 1.175) | 2.282 (1.952-2.612) | 1 (1 - 2) | 0.399 (0.305 - 0.498) | 3 (2 - 4) | 0.3 (0.217 - 0.406) | -0.398 (-0.555to-0.241) | 36 (27 - 46) | 9.773 (7.406 - 12.427) | 78 (56 - 108) | 8.482 (6.089 - 11.595) | 0.134 (-0.037-0.305) |
| Denmark | Female | 42 (38 - 47) | 1.129 (1.008 - 1.256) | 105 (87 - 125) | 2.006 (1.673 - 2.39) | 1.956 (1.704-2.208) | 20 (17 - 22) | 0.48 (0.427 - 0.53) | 32 (27 - 37) | 0.544 (0.458 - 0.639) | 0.242 (0.036-0.449) | 513 (459 - 564) | 13.982 (12.548 - 15.414) | 776 (659 - 913) | 15.078 (12.8 - 17.704) | 0.116 (-0.094-0.326) |
|  | Male | 99 (88 - 111) | 3.002 (2.656 - 3.36) | 284 (240 - 333) | 5.712 (4.829 - 6.654) | 2.235 (1.816-2.655) | 53 (47 - 58) | 1.578 (1.418 - 1.747) | 107 (91 - 124) | 2.037 (1.744 - 2.365) | 0.69 (0.335-1.045) | 1501 (1344 - 1670) | 46.132 (41.02 - 51.56) | 2754 (2338 - 3215) | 56.194 (47.906 - 65.162) | 0.52 (0.129-0.912) |
| Finland | Female | 18 (16 - 21) | 0.483 (0.418 - 0.553) | 47 (38 - 57) | 0.896 (0.74 - 1.078) | 2.439 (2.262-2.615) | 9 (8 - 10) | 0.209 (0.184 - 0.237) | 12 (10 - 15) | 0.199 (0.167 - 0.234) | 0.064 (-0.063-0.191) | 216 (190 - 246) | 5.794 (5.062 - 6.613) | 295 (248 - 348) | 5.822 (4.893 - 6.914) | 0.251 (0.141-0.361) |
|  | Male | 49 (43 - 56) | 1.684 (1.476 - 1.909) | 103 (87 - 123) | 2.223 (1.888 - 2.664) | 1.342 (1.102-1.583) | 28 (25 - 32) | 0.974 (0.87 - 1.098) | 40 (33 - 47) | 0.79 (0.671 - 0.94) | -0.325 (-0.513to-0.136) | 841 (743 - 951) | 28.389 (25.118 - 32.183) | 1065 (901 - 1275) | 23.281 (19.636 - 27.826) | -0.303 (-0.484to-0.123) |
| France | Female | 399 (347 - 496) | 1.016 (0.874 - 1.264) | 1274 (1084 - 1500) | 2.171 (1.853 - 2.536) | 3.104 (2.831-3.378) | 170 (149 - 208) | 0.396 (0.347 - 0.487) | 306 (258 - 364) | 0.448 (0.382 - 0.522) | 0.843 (0.559-1.127) | 4526 (3957 - 5562) | 11.903 (10.398 - 14.67) | 7794 (6664 - 9158) | 13.573 (11.721 - 15.752) | 0.825 (0.55-1.101) |
|  | Male | 4567 (4113 - 5085) | 13.756 (12.44 - 15.235) | 5056 (4272 - 5887) | 9.578 (8.037 - 11.133) | -1.262 (-1.468to-1.056) | 2274 (2069 - 2488) | 6.785 (6.187 - 7.397) | 1549 (1337 - 1793) | 2.762 (2.371 - 3.206) | -3.108 (-3.361to-2.856) | 69959 (63300 - 76875) | 211.419 (191.867 - 232.461) | 41392 (35323 - 48102) | 79.59 (67.572 - 92.355) | -3.412 (-3.657to-3.166) |
| Germany | Female | 361 (319 - 425) | 0.567 (0.503 - 0.666) | 1427 (1219 - 1672) | 1.683 (1.447 - 1.959) | 2.729 (2.262-3.198) | 223 (196 - 263) | 0.325 (0.287 - 0.38) | 597 (509 - 690) | 0.628 (0.542 - 0.721) | 1.5 (1.158-1.843) | 5939 (5211 - 6949) | 9.778 (8.654 - 11.439) | 14255 (12254 - 16457) | 17.261 (14.923 - 19.797) | 1.144 (0.782-1.506) |
|  | Male | 2397 (2137 - 2699) | 4.656 (4.165 - 5.238) | 4547 (3771 - 5385) | 5.786 (4.79 - 6.833) | -0.02 (-0.423-0.385) | 1471 (1313 - 1654) | 2.881 (2.577 - 3.227) | 2181 (1830 - 2570) | 2.673 (2.243 - 3.155) | -0.753 (-1.026to-0.479) | 49095 (43822 - 54900) | 95.244 (85.316 - 106.169) | 56050 (46511 - 66481) | 72.958 (60.782 - 86.465) | -1.473 (-1.786to-1.159) |
| Greece | Female | 19 (17 - 20) | 0.238 (0.218 - 0.259) | 45 (40 - 49) | 0.454 (0.406 - 0.498) | 2.048 (1.883-2.213) | 7 (7 - 8) | 0.091 (0.084 - 0.097) | 15 (14 - 17) | 0.13 (0.118 - 0.141) | 1.175 (1.006-1.344) | 185 (173 - 198) | 2.41 (2.256 - 2.589) | 363 (328 - 394) | 3.716 (3.365 - 4.024) | 1.453 (1.28-1.626) |
|  | Male | 50 (45 - 54) | 0.72 (0.661 - 0.787) | 108 (97 - 120) | 1.257 (1.127 - 1.402) | 1.521 (1.36-1.682) | 23 (21 - 25) | 0.329 (0.305 - 0.358) | 43 (39 - 47) | 0.458 (0.414 - 0.504) | 0.787 (0.576-0.998) | 659 (606 - 725) | 9.635 (8.864 - 10.561) | 1152 (1036 - 1266) | 13.572 (12.166 - 14.945) | 0.88 (0.709-1.052) |
| Iceland | Female | 1 (1 - 1) | 0.584 (0.502 - 0.67) | 2 (2 - 3) | 0.873 (0.728 - 1.036) | 1.064 (0.888-1.239) | 0 (0 - 0) | 0.194 (0.168 - 0.221) | 1 (0 - 1) | 0.189 (0.157 - 0.222) | -0.185 (-0.336to-0.034) | 8 (7 - 9) | 5.516 (4.765 - 6.286) | 14 (12 - 17) | 5.384 (4.471 - 6.294) | -0.271 (-0.428to-0.113) |
|  | Male | 2 (1 - 2) | 1.382 (1.158 - 1.652) | 4 (3 - 5) | 1.638 (1.355 - 2.005) | 0.597 (0.426-0.768) | 1 (1 - 1) | 0.596 (0.506 - 0.703) | 1 (1 - 1) | 0.445 (0.371 - 0.536) | -0.863 (-1.007to-0.718) | 22 (18 - 26) | 17.126 (14.483 - 20.41) | 34 (28 - 41) | 13.537 (11.207 - 16.564) | -0.726 (-0.86to-0.592) |
| Ireland | Female | 17 (16 - 20) | 0.859 (0.772 - 0.963) | 51 (44 - 60) | 1.378 (1.167 - 1.616) | 2.424 (2.071-2.778) | 8 (7 - 9) | 0.373 (0.334 - 0.418) | 13 (11 - 15) | 0.316 (0.269 - 0.365) | 0.033 (-0.299-0.367) | 206 (184 - 230) | 10.334 (9.301 - 11.49) | 337 (288 - 389) | 9.118 (7.802 - 10.532) | 0.209 (-0.122-0.541) |
|  | Male | 43 (37 - 51) | 2.37 (2.034 - 2.769) | 107 (87 - 129) | 3.013 (2.451 - 3.65) | 1.296 (1.05-1.542) | 23 (20 - 27) | 1.275 (1.099 - 1.49) | 34 (28 - 40) | 0.923 (0.76 - 1.103) | -0.872 (-1.061to-0.684) | 661 (566 - 777) | 36.431 (31.116 - 42.942) | 935 (755 - 1132) | 26.483 (21.332 - 32.147) | -0.759 (-0.963to-0.555) |
| Israel | Female | 5 (4 - 5) | 0.194 (0.17 - 0.221) | 18 (15 - 21) | 0.304 (0.257 - 0.358) | 1.668 (1.445-1.891) | 2 (2 - 3) | 0.096 (0.084 - 0.109) | 6 (5 - 7) | 0.093 (0.078 - 0.108) | -0.074 (-0.267-0.119) | 65 (57 - 73) | 2.644 (2.312 - 3.001) | 149 (127 - 174) | 2.551 (2.162 - 2.996) | -0.009 (-0.204-0.187) |
|  | Male | 11 (9 - 12) | 0.507 (0.431 - 0.593) | 39 (33 - 47) | 0.746 (0.63 - 0.907) | 1.094 (0.915-1.272) | 6 (5 - 8) | 0.303 (0.258 - 0.353) | 15 (13 - 18) | 0.284 (0.24 - 0.344) | -0.515 (-0.695to-0.334) | 182 (155 - 212) | 8.663 (7.381 - 10.129) | 430 (364 - 517) | 8.243 (6.978 - 9.94) | -0.453 (-0.638to-0.267) |
| Italy | Female | 184 (170 - 198) | 0.409 (0.379 - 0.441) | 420 (365 - 471) | 0.667 (0.588 - 0.746) | 1.84 (1.607-2.073) | 112 (103 - 120) | 0.233 (0.215 - 0.249) | 187 (161 - 210) | 0.25 (0.22 - 0.278) | 0.453 (0.246-0.661) | 2853 (2655 - 3037) | 6.525 (6.096 - 6.94) | 4201 (3688 - 4674) | 6.837 (6.096 - 7.573) | 0.366 (0.166-0.566) |
|  | Male | 1274 (1178 - 1373) | 3.396 (3.146 - 3.662) | 1523 (1372 - 1681) | 2.704 (2.427 - 2.985) | -0.889 (-1.023to-0.754) | 745 (697 - 794) | 1.972 (1.846 - 2.1) | 701 (632 - 772) | 1.171 (1.06 - 1.29) | -1.823 (-1.924to-1.722) | 21885 (20427 - 23396) | 58.777 (54.864 - 62.84) | 17353 (15758 - 19157) | 31.411 (28.593 - 34.677) | -2.156 (-2.254to-2.059) |
| Luxembourg | Female | 3 (2 - 3) | 0.967 (0.887 - 1.06) | 8 (7 - 9) | 1.637 (1.435 - 1.861) | 2.14 (1.801-2.48) | 1 (1 - 1) | 0.425 (0.394 - 0.459) | 2 (2 - 3) | 0.417 (0.369 - 0.47) | 0.288 (0.093-0.483) | 34 (31 - 36) | 12.291 (11.28 - 13.31) | 59 (52 - 66) | 11.726 (10.398 - 13.189) | 0.193 (-0.018-0.405) |
|  | Male | 13 (12 - 14) | 5.378 (4.944 - 5.885) | 26 (22 - 30) | 5.322 (4.493 - 6.051) | -0.169 (-0.373-0.035) | 7 (6 - 7) | 2.92 (2.687 - 3.197) | 9 (8 - 10) | 1.763 (1.515 - 1.986) | -1.828 (-2.017to-1.639) | 206 (188 - 226) | 87.064 (79.667 - 95.358) | 251 (213 - 285) | 50.678 (43.238 - 57.559) | -1.979 (-2.164to-1.794) |
| Malta | Female | 1 (1 - 1) | 0.35 (0.311 - 0.394) | 3 (2 - 3) | 0.719 (0.613 - 0.846) | 2.372 (2.181-2.565) | 0 (0 - 0) | 0.158 (0.143 - 0.177) | 1 (1 - 1) | 0.192 (0.164 - 0.224) | 0.573 (0.399-0.748) | 10 (9 - 11) | 4.428 (3.983 - 4.963) | 22 (19 - 26) | 5.573 (4.703 - 6.51) | 0.788 (0.615-0.962) |
|  | Male | 3 (3 - 4) | 1.637 (1.462 - 1.827) | 8 (7 - 10) | 2.291 (1.956 - 2.664) | 1.039 (0.896-1.182) | 2 (2 - 2) | 0.919 (0.828 - 1.026) | 3 (3 - 3) | 0.769 (0.667 - 0.892) | -0.695 (-0.843to-0.547) | 51 (46 - 57) | 26.138 (23.406 - 29.251) | 84 (73 - 98) | 23.52 (20.237 - 27.304) | -0.41 (-0.539to-0.281) |
| Monaco | Female | 0 (0 - 0) | 0.579 (0.353 - 1.007) | 0 (0 - 1) | 1.133 (0.685 - 1.606) | 2.409 (1.974-2.846) | 0 (0 - 0) | 0.191 (0.118 - 0.332) | 0 (0 - 0) | 0.281 (0.17 - 0.387) | 1.463 (1.069-1.858) | 2 (1 - 3) | 5.531 (3.474 - 9.538) | 3 (2 - 4) | 8.154 (4.886 - 11.583) | 1.459 (1.057-1.863) |
|  | Male | 0 (0 - 0) | 1.212 (0.864 - 1.633) | 1 (1 - 1) | 1.973 (1.456 - 2.613) | 1.789 (1.58-1.997) | 0 (0 - 0) | 0.509 (0.372 - 0.679) | 0 (0 - 0) | 0.607 (0.452 - 0.789) | 0.722 (0.546-0.899) | 4 (3 - 5) | 15.411 (11.212 - 20.692) | 6 (5 - 8) | 18.862 (13.917 - 24.941) | 0.81 (0.632-0.988) |
| Netherlands | Female | 118 (104 - 133) | 1.195 (1.046 - 1.357) | 328 (280 - 389) | 2.052 (1.755 - 2.427) | 1.887 (1.601-2.173) | 25 (22 - 28) | 0.228 (0.203 - 0.255) | 49 (42 - 59) | 0.278 (0.239 - 0.331) | 0.64 (0.433-0.846) | 668 (596 - 748) | 6.793 (6.054 - 7.668) | 1296 (1098 - 1542) | 8.269 (7.021 - 9.791) | 0.644 (0.419-0.869) |
|  | Male | 317 (284 - 353) | 3.683 (3.305 - 4.101) | 926 (790 - 1089) | 5.986 (5.124 - 7) | 1.435 (1.264-1.607) | 81 (72 - 89) | 0.953 (0.852 - 1.048) | 163 (141 - 189) | 1.026 (0.893 - 1.187) | -0.111 (-0.361-0.14) | 2367 (2122 - 2607) | 27.539 (24.701 - 30.394) | 4388 (3783 - 5118) | 29.222 (25.312 - 34.065) | -0.136 (-0.376-0.104) |
| Norway | Female | 18 (17 - 20) | 0.559 (0.514 - 0.604) | 36 (31 - 42) | 0.775 (0.676 - 0.887) | 1.149 (0.602-1.7) | 9 (8 - 9) | 0.235 (0.217 - 0.251) | 10 (9 - 12) | 0.2 (0.175 - 0.228) | -0.49 (-0.917to-0.062) | 196 (182 - 211) | 6.238 (5.837 - 6.695) | 238 (209 - 269) | 5.128 (4.524 - 5.787) | -0.604 (-1.055to-0.152) |
|  | Male | 61 (56 - 67) | 2.242 (2.051 - 2.45) | 126 (111 - 143) | 2.83 (2.485 - 3.232) | 1.28 (0.892-1.669) | 28 (26 - 30) | 0.995 (0.917 - 1.07) | 33 (29 - 37) | 0.709 (0.624 - 0.805) | -0.773 (-1.108to-0.438) | 729 (671 - 789) | 27.19 (25.015 - 29.373) | 839 (733 - 956) | 18.98 (16.594 - 21.613) | -0.819 (-1.146to-0.492) |
| Portugal | Female | 24 (22 - 27) | 0.328 (0.294 - 0.368) | 74 (64 - 85) | 0.696 (0.599 - 0.791) | 2.65 (2.429-2.872) | 14 (13 - 16) | 0.183 (0.164 - 0.204) | 24 (20 - 28) | 0.195 (0.167 - 0.223) | 0.246 (0.02-0.472) | 359 (323 - 400) | 5.013 (4.512 - 5.605) | 592 (511 - 673) | 5.661 (4.888 - 6.37) | 0.486 (0.258-0.715) |
|  | Male | 201 (180 - 225) | 3.403 (3.036 - 3.814) | 877 (763 - 989) | 10.151 (8.785 - 11.546) | 3.898 (3.498-4.301) | 121 (109 - 135) | 2.041 (1.835 - 2.267) | 284 (247 - 320) | 3.089 (2.69 - 3.465) | 1.425 (1.195-1.656) | 3626 (3225 - 4046) | 61.641 (54.795 - 68.725) | 8253 (7163 - 9255) | 96.062 (83.219 - 108.357) | 1.578 (1.291-1.866) |
| San Marino | Female | 0 (0 - 0) | 0.362 (0.261 - 0.524) | 0 (0 - 0) | 0.308 (0.171 - 0.48) | 0.552 (0.167-0.939) | 0 (0 - 0) | 0.117 (0.085 - 0.165) | 0 (0 - 0) | 0.074 (0.041 - 0.111) | -0.369 (-0.717to-0.021) | 1 (0 - 1) | 3.233 (2.301 - 4.564) | 1 (0 - 1) | 2.152 (1.16 - 3.346) | -0.17 (-0.525-0.186) |
|  | Male | 0 (0 - 1) | 2.796 (2.147 - 3.542) | 1 (0 - 1) | 1.944 (1.104 - 3.03) | -0.175 (-0.575-0.227) | 0 (0 - 0) | 1.146 (0.882 - 1.455) | 0 (0 - 0) | 0.602 (0.348 - 0.914) | -1.018 (-1.382to-0.653) | 5 (4 - 6) | 33.299 (25.635 - 42.497) | 5 (3 - 8) | 18.067 (10.135 - 27.95) | -0.956 (-1.305to-0.607) |
| Spain | Female | 89 (79 - 100) | 0.322 (0.287 - 0.36) | 359 (303 - 427) | 0.834 (0.708 - 0.995) | 3.635 (3.339-3.931) | 41 (37 - 45) | 0.138 (0.124 - 0.152) | 95 (78 - 112) | 0.191 (0.16 - 0.225) | 1.552 (1.396-1.708) | 1027 (928 - 1127) | 3.78 (3.419 - 4.158) | 2380 (1996 - 2812) | 5.569 (4.688 - 6.577) | 1.708 (1.519-1.898) |
|  | Male | 998 (869 - 1140) | 4.366 (3.797 - 4.996) | 2193 (1808 - 2697) | 5.597 (4.606 - 6.873) | 0.223 (-0.076-0.522) | 497 (434 - 565) | 2.153 (1.885 - 2.439) | 749 (618 - 904) | 1.847 (1.521 - 2.239) | -1.013 (-1.214to-0.812) | 15425 (13501 - 17515) | 68.178 (59.841 - 77.276) | 20145 (16637 - 24577) | 51.905 (42.883 - 63.418) | -1.512 (-1.747to-1.276) |
| Sweden | Female | 41 (36 - 46) | 0.569 (0.506 - 0.643) | 85 (70 - 100) | 0.882 (0.738 - 1.043) | 1.086 (0.795-1.378) | 18 (16 - 21) | 0.224 (0.2 - 0.254) | 28 (23 - 33) | 0.247 (0.206 - 0.292) | -0.045 (-0.337-0.248) | 409 (364 - 460) | 5.826 (5.2 - 6.537) | 589 (490 - 693) | 6.166 (5.144 - 7.291) | -0.098 (-0.373-0.178) |
|  | Male | 109 (98 - 122) | 1.785 (1.593 - 1.988) | 178 (146 - 211) | 1.976 (1.618 - 2.347) | 1.284 (0.928-1.642) | 65 (58 - 73) | 1.033 (0.92 - 1.155) | 79 (65 - 94) | 0.803 (0.661 - 0.953) | -0.048 (-0.314-0.218) | 1629 (1452 - 1821) | 27.098 (24.117 - 30.215) | 1785 (1466 - 2129) | 19.891 (16.314 - 23.759) | -0.119 (-0.423-0.186) |
| Switzerland | Female | 36 (32 - 40) | 0.701 (0.626 - 0.79) | 103 (85 - 123) | 1.265 (1.051 - 1.517) | 2.159 (1.879-2.439) | 16 (14 - 18) | 0.29 (0.26 - 0.329) | 34 (28 - 41) | 0.37 (0.311 - 0.44) | 1.08 (0.834-1.327) | 411 (369 - 465) | 8.3 (7.498 - 9.334) | 808 (682 - 964) | 10.157 (8.688 - 12.1) | 0.912 (0.659-1.166) |
|  | Male | 127 (115 - 143) | 2.984 (2.698 - 3.368) | 290 (248 - 346) | 3.845 (3.291 - 4.578) | 0.591 (0.158-1.026) | 71 (64 - 80) | 1.646 (1.491 - 1.856) | 129 (111 - 155) | 1.628 (1.393 - 1.943) | -0.233 (-0.537-0.072) | 2006 (1818 - 2261) | 47.229 (42.826 - 53.135) | 3297 (2811 - 3943) | 43.849 (37.651 - 52.165) | -0.47 (-0.79to-0.148) |
| United Kingdom | Female | 405 (388 - 418) | 0.884 (0.851 - 0.91) | 963 (899 - 1008) | 1.633 (1.548 - 1.705) | 2.553 (2.221-2.885) | 193 (183 - 199) | 0.386 (0.369 - 0.397) | 301 (275 - 317) | 0.457 (0.424 - 0.479) | 0.867 (0.48-1.254) | 4513 (4335 - 4640) | 10.172 (9.815 - 10.436) | 7187 (6735 - 7532) | 12.426 (11.797 - 12.993) | 1.005 (0.628-1.384) |
|  | Male | 722 (705 - 738) | 1.963 (1.916 - 2.008) | 2582 (2470 - 2681) | 4.849 (4.636 - 5.038) | 3.41 (3.17-3.651) | 358 (350 - 366) | 0.965 (0.942 - 0.986) | 915 (867 - 950) | 1.615 (1.536 - 1.679) | 1.934 (1.633-2.236) | 9537 (9324 - 9756) | 26.168 (25.571 - 26.78) | 23753 (22713 - 24735) | 45.07 (43.244 - 47.001) | 2.031 (1.763-2.299) |
| Bolivia  (Plurinational State of) | Female | 6 (3 - 10) | 0.313 (0.19 - 0.538) | 13 (9 - 21) | 0.272 (0.178 - 0.426) | -0.572 (-0.647to-0.497) | 5 (3 - 9) | 0.302 (0.185 - 0.508) | 11 (7 - 17) | 0.229 (0.151 - 0.35) | -1.01 (-1.073to-0.947) | 155 (94 - 270) | 8.165 (4.961 - 14.027) | 307 (197 - 472) | 5.994 (3.922 - 9.254) | -1.139 (-1.208to-1.07) |
|  | Male | 9 (6 - 13) | 0.607 (0.416 - 0.837) | 22 (15 - 31) | 0.492 (0.346 - 0.704) | -0.659 (-0.832to-0.485) | 9 (6 - 13) | 0.606 (0.418 - 0.837) | 19 (13 - 28) | 0.453 (0.319 - 0.647) | -0.91 (-1.068to-0.752) | 265 (183 - 375) | 15.999 (11.068 - 22.334) | 529 (356 - 778) | 11.391 (7.762 - 16.482) | -1.129 (-1.302to-0.955) |
| Ecuador | Female | 7 (6 - 8) | 0.246 (0.221 - 0.273) | 17 (13 - 22) | 0.192 (0.147 - 0.249) | -0.915 (-1.414to-0.414) | 6 (5 - 7) | 0.222 (0.2 - 0.246) | 12 (9 - 15) | 0.137 (0.107 - 0.176) | -1.588 (-2.111to-1.063) | 176 (159 - 194) | 5.818 (5.252 - 6.406) | 302 (231 - 391) | 3.455 (2.647 - 4.471) | -1.749 (-2.271to-1.225) |
|  | Male | 16 (14 - 18) | 0.603 (0.522 - 0.688) | 34 (25 - 46) | 0.442 (0.321 - 0.583) | -0.931 (-1.72to-0.134) | 15 (13 - 17) | 0.576 (0.5 - 0.661) | 27 (20 - 36) | 0.36 (0.268 - 0.472) | -1.366 (-2.16to-0.566) | 419 (362 - 478) | 14.847 (12.773 - 16.963) | 715 (526 - 953) | 8.946 (6.598 - 11.924) | -1.582 (-2.368to-0.791) |
| Peru | Female | 19 (13 - 24) | 0.291 (0.202 - 0.376) | 45 (30 - 69) | 0.253 (0.172 - 0.382) | -0.686 (-0.987to-0.383) | 16 (11 - 21) | 0.259 (0.178 - 0.337) | 27 (19 - 40) | 0.152 (0.105 - 0.226) | -1.981 (-2.232to-1.729) | 476 (332 - 613) | 6.98 (4.852 - 9.024) | 719 (481 - 1087) | 4.001 (2.686 - 6.052) | -2.067 (-2.321to-1.813) |
|  | Male | 31 (25 - 39) | 0.521 (0.423 - 0.659) | 62 (44 - 86) | 0.385 (0.269 - 0.528) | -1.196 (-1.767to-0.621) | 29 (23 - 36) | 0.496 (0.405 - 0.619) | 45 (32 - 62) | 0.282 (0.2 - 0.386) | -2.063 (-2.579to-1.545) | 802 (643 - 1004) | 12.769 (10.279 - 16.023) | 1154 (808 - 1597) | 6.979 (4.866 - 9.669) | -2.226 (-2.757to-1.692) |
| Antigua and Barbuda | Female | 0 (0 - 0) | 0.414 (0.374 - 0.457) | 0 (0 - 0) | 0.267 (0.245 - 0.29) | -1.49 (-1.911to-1.068) | 0 (0 - 0) | 0.328 (0.296 - 0.361) | 0 (0 - 0) | 0.182 (0.168 - 0.198) | -1.943 (-2.361to-1.524) | 2 (2 - 3) | 7.898 (7.109 - 8.702) | 2 (2 - 3) | 4.146 (3.827 - 4.519) | -2.084 (-2.522to-1.643) |
|  | Male | 1 (1 - 1) | 3.775 (3.448 - 4.094) | 1 (1 - 2) | 2.628 (2.298 - 2.899) | -0.985 (-1.618to-0.348) | 1 (1 - 1) | 3.184 (2.916 - 3.47) | 1 (1 - 1) | 1.984 (1.737 - 2.188) | -1.286 (-1.932to-0.635) | 18 (17 - 20) | 81.819 (74.681 - 89.377) | 26 (23 - 29) | 48.631 (42.91 - 53.912) | -1.489 (-2.098to-0.876) |
| Bahamas | Female | 1 (1 - 1) | 0.953 (0.864 - 1.046) | 1 (1 - 1) | 0.512 (0.413 - 0.633) | -1.748 (-2.118to-1.377) | 1 (1 - 1) | 0.763 (0.689 - 0.836) | 1 (1 - 1) | 0.374 (0.305 - 0.458) | -2.096 (-2.485to-1.705) | 19 (17 - 21) | 20.218 (18.279 - 22.277) | 22 (17 - 28) | 9.415 (7.494 - 11.833) | -2.261 (-2.661to-1.86) |
|  | Male | 4 (3 - 4) | 5.311 (4.765 - 5.902) | 8 (6 - 10) | 3.866 (3.086 - 4.8) | -0.723 (-1.195to-0.249) | 3 (3 - 4) | 4.499 (4.058 - 4.96) | 6 (5 - 7) | 3.049 (2.452 - 3.766) | -1.008 (-1.499to-0.514) | 98 (88 - 109) | 127.662 (114.247 - 141.759) | 172 (136 - 215) | 82.983 (65.973 - 102.841) | -1.124 (-1.629to-0.617) |
| Barbados | Female | 1 (1 - 1) | 0.661 (0.603 - 0.728) | 1 (1 - 2) | 0.443 (0.35 - 0.55) | -1.013 (-1.29to-0.736) | 1 (1 - 1) | 0.521 (0.476 - 0.572) | 1 (1 - 1) | 0.301 (0.24 - 0.369) | -1.459 (-1.733to-1.185) | 19 (17 - 21) | 12.295 (11.218 - 13.514) | 18 (14 - 23) | 6.82 (5.339 - 8.515) | -1.595 (-1.89to-1.299) |
|  | Male | 5 (5 - 6) | 4.517 (4.079 - 4.97) | 7 (5 - 9) | 3.114 (2.371 - 4.02) | -1.212 (-1.55to-0.872) | 4 (4 - 5) | 3.746 (3.403 - 4.119) | 5 (4 - 7) | 2.305 (1.733 - 2.955) | -1.586 (-1.932to-1.239) | 109 (98 - 120) | 97.596 (87.938 - 107.89) | 132 (98 - 171) | 57.606 (42.742 - 74.635) | -1.651 (-1.991to-1.31) |
| Belize | Female | 0 (0 - 0) | 0.455 (0.414 - 0.502) | 1 (0 - 1) | 0.335 (0.293 - 0.387) | -1.038 (-1.306to-0.77) | 0 (0 - 0) | 0.387 (0.353 - 0.427) | 0 (0 - 0) | 0.256 (0.224 - 0.295) | -1.406 (-1.665to-1.147) | 5 (4 - 5) | 9.785 (8.949 - 10.777) | 11 (9 - 12) | 6.473 (5.631 - 7.479) | -1.415 (-1.671to-1.158) |
|  | Male | 1 (0 - 1) | 1.079 (0.989 - 1.174) | 2 (1 - 2) | 0.987 (0.836 - 1.15) | -0.088 (-0.29-0.113) | 0 (0 - 0) | 0.969 (0.888 - 1.059) | 1 (1 - 1) | 0.811 (0.689 - 0.94) | -0.411 (-0.624to-0.198) | 12 (11 - 14) | 25.696 (23.566 - 28.029) | 37 (32 - 43) | 22.017 (18.667 - 25.415) | -0.306 (-0.506to-0.106) |
| Bermuda | Female | 0 (0 - 0) | 0.656 (0.569 - 0.743) | 0 (0 - 0) | 0.278 (0.218 - 0.364) | -2.998 (-3.383to-2.611) | 0 (0 - 0) | 0.459 (0.397 - 0.521) | 0 (0 - 0) | 0.119 (0.094 - 0.154) | -4.531 (-4.975to-4.084) | 4 (3 - 4) | 10.407 (8.997 - 11.879) | 2 (1 - 2) | 2.633 (2.077 - 3.437) | -4.632 (-5.1to-4.162) |
|  | Male | 2 (2 - 2) | 6.968 (6.188 - 7.871) | 3 (2 - 4) | 4.945 (4.074 - 6.111) | -0.571 (-1.173-0.035) | 1 (1 - 2) | 5.23 (4.67 - 5.873) | 1 (1 - 2) | 2.433 (2.005 - 3.017) | -1.955 (-2.621to-1.284) | 38 (34 - 43) | 133.023 (118.325 - 150.276) | 35 (28 - 43) | 60.158 (49.102 - 74.792) | -2.072 (-2.752to-1.388) |
| Cuba | Female | 41 (38 - 45) | 0.805 (0.73 - 0.879) | 45 (37 - 54) | 0.441 (0.362 - 0.521) | -1.734 (-2.126to-1.339) | 29 (26 - 31) | 0.565 (0.513 - 0.615) | 27 (22 - 32) | 0.246 (0.201 - 0.292) | -2.394 (-2.821to-1.966) | 715 (655 - 779) | 13.767 (12.555 - 15.013) | 579 (474 - 686) | 5.77 (4.741 - 6.806) | -2.533 (-2.932to-2.132) |
|  | Male | 161 (146 - 178) | 3.21 (2.905 - 3.541) | 335 (278 - 402) | 3.648 (3.031 - 4.364) | 0.664 (0.031-1.3) | 125 (113 - 137) | 2.502 (2.271 - 2.766) | 210 (174 - 252) | 2.277 (1.891 - 2.717) | -0.023 (-0.667-0.625) | 3195 (2892 - 3500) | 63.303 (57.256 - 69.486) | 5463 (4540 - 6588) | 59.827 (49.841 - 72.289) | 0.08 (-0.54-0.704) |
| Dominica | Female | 0 (0 - 0) | 0.848 (0.594 - 1.09) | 0 (0 - 0) | 0.509 (0.342 - 0.725) | -1.558 (-1.858to-1.256) | 0 (0 - 0) | 0.761 (0.533 - 0.983) | 0 (0 - 0) | 0.424 (0.289 - 0.592) | -1.737 (-2.055to-1.418) | 6 (4 - 8) | 17.252 (12.095 - 21.787) | 4 (3 - 6) | 9.846 (6.558 - 13.997) | -1.68 (-2to-1.359) |
|  | Male | 1 (1 - 1) | 5.148 (4.304 - 6.164) | 2 (1 - 2) | 3.755 (2.794 - 4.955) | -0.965 (-1.213to-0.716) | 1 (1 - 1) | 4.721 (3.945 - 5.65) | 1 (1 - 2) | 3.271 (2.424 - 4.309) | -1.112 (-1.361to-0.862) | 27 (23 - 33) | 118.812 (99.561 - 141.53) | 36 (27 - 48) | 83.531 (61.553 - 111.986) | -1.036 (-1.313to-0.758) |
| Dominican Republic | Female | 17 (12 - 21) | 0.895 (0.636 - 1.152) | 44 (30 - 62) | 0.841 (0.571 - 1.19) | -0.036 (-0.148-0.075) | 15 (11 - 19) | 0.844 (0.601 - 1.083) | 36 (24 - 51) | 0.683 (0.46 - 0.983) | -0.435 (-0.6to-0.268) | 410 (295 - 521) | 20.087 (14.583 - 25.67) | 884 (596 - 1244) | 16.709 (11.25 - 23.476) | -0.37 (-0.507to-0.233) |
|  | Male | 37 (29 - 46) | 1.973 (1.551 - 2.477) | 134 (89 - 194) | 2.726 (1.811 - 3.922) | 1.403 (1.286-1.521) | 34 (27 - 42) | 1.878 (1.475 - 2.361) | 113 (74 - 161) | 2.33 (1.546 - 3.312) | 1.091 (0.96-1.221) | 970 (774 - 1203) | 49.144 (39.025 - 61.231) | 3144 (2040 - 4656) | 62.158 (40.512 - 91.126) | 1.161 (1.011-1.311) |
| Grenada | Female | 0 (0 - 0) | 0.887 (0.75 - 1.044) | 0 (0 - 0) | 0.526 (0.437 - 0.619) | -1.274 (-1.888to-0.657) | 0 (0 - 0) | 0.77 (0.653 - 0.907) | 0 (0 - 0) | 0.403 (0.336 - 0.476) | -1.567 (-2.211to-0.918) | 8 (6 - 9) | 19.926 (16.877 - 23.326) | 6 (5 - 7) | 9.681 (7.971 - 11.605) | -1.792 (-2.433to-1.147) |
|  | Male | 1 (1 - 1) | 3.333 (2.85 - 3.918) | 1 (1 - 1) | 2.106 (1.78 - 2.509) | -1.242 (-1.654to-0.829) | 1 (1 - 1) | 3.02 (2.598 - 3.558) | 1 (1 - 1) | 1.767 (1.506 - 2.085) | -1.371 (-1.857to-0.882) | 23 (20 - 27) | 82.008 (69.311 - 96.989) | 26 (21 - 32) | 44.115 (36.738 - 52.752) | -1.698 (-2.05to-1.344) |
| Guyana | Female | 1 (1 - 1) | 0.408 (0.348 - 0.475) | 1 (1 - 1) | 0.273 (0.201 - 0.357) | -1.118 (-1.506to-0.727) | 1 (1 - 1) | 0.388 (0.333 - 0.451) | 1 (1 - 1) | 0.238 (0.179 - 0.308) | -1.315 (-1.698to-0.932) | 21 (18 - 25) | 9.841 (8.33 - 11.582) | 23 (16 - 30) | 6.175 (4.457 - 8.115) | -1.277 (-1.65to-0.902) |
|  | Male | 3 (2 - 3) | 1.333 (1.113 - 1.579) | 3 (3 - 5) | 1.048 (0.779 - 1.412) | -0.35 (-0.726-0.028) | 2 (2 - 3) | 1.277 (1.071 - 1.514) | 3 (2 - 4) | 0.942 (0.699 - 1.263) | -0.519 (-0.89to-0.148) | 72 (59 - 86) | 35.406 (29.27 - 42.331) | 92 (66 - 126) | 26.636 (19.242 - 36.231) | -0.426 (-0.793to-0.057) |
| Haiti | Female | 10 (5 - 19) | 0.607 (0.335 - 1.096) | 18 (9 - 32) | 0.461 (0.259 - 0.818) | -0.855 (-0.892to-0.817) | 10 (5 - 18) | 0.619 (0.346 - 1.09) | 16 (9 - 29) | 0.455 (0.263 - 0.781) | -0.962 (-1to-0.924) | 282 (147 - 551) | 15.613 (8.385 - 29.585) | 479 (252 - 886) | 11.235 (6.119 - 20.386) | -1.028 (-1.068to-0.988) |
|  | Male | 34 (21 - 54) | 2.046 (1.305 - 3.24) | 57 (34 - 95) | 1.573 (0.947 - 2.596) | -0.702 (-0.879to-0.524) | 33 (21 - 53) | 2.076 (1.344 - 3.281) | 54 (32 - 90) | 1.556 (0.948 - 2.541) | -0.783 (-0.957to-0.609) | 992 (605 - 1601) | 55.963 (34.958 - 90.047) | 1630 (956 - 2716) | 41.519 (24.758 - 68.657) | -0.81 (-0.993to-0.628) |
| Jamaica | Female | 5 (4 - 6) | 0.515 (0.448 - 0.59) | 7 (5 - 9) | 0.4 (0.297 - 0.524) | -0.801 (-1.276to-0.323) | 4 (3 - 4) | 0.407 (0.355 - 0.462) | 5 (4 - 6) | 0.278 (0.21 - 0.361) | -1.205 (-1.709to-0.699) | 91 (79 - 104) | 9.903 (8.578 - 11.343) | 111 (81 - 145) | 6.893 (5.074 - 9.05) | -1.127 (-1.627to-0.624) |
|  | Male | 12 (11 - 14) | 1.511 (1.343 - 1.707) | 20 (15 - 27) | 1.319 (0.97 - 1.788) | -0.184 (-0.724-0.36) | 10 (9 - 12) | 1.287 (1.144 - 1.446) | 15 (11 - 20) | 1.026 (0.757 - 1.368) | -0.44 (-0.981-0.104) | 261 (231 - 295) | 32.6 (28.769 - 36.937) | 397 (290 - 543) | 26.242 (19.147 - 35.762) | -0.481 (-1.023-0.064) |
| Puerto Rico | Female | 10 (9 - 12) | 0.54 (0.469 - 0.618) | 10 (8 - 13) | 0.282 (0.228 - 0.35) | -2.033 (-2.437to-1.627) | 7 (6 - 8) | 0.389 (0.335 - 0.445) | 6 (5 - 7) | 0.135 (0.11 - 0.166) | -3.427 (-3.832to-3.02) | 170 (147 - 194) | 8.878 (7.65 - 10.079) | 112 (91 - 139) | 3.23 (2.609 - 4.028) | -3.277 (-3.682to-2.871) |
|  | Male | 69 (61 - 77) | 4.203 (3.725 - 4.671) | 58 (46 - 71) | 2.102 (1.678 - 2.59) | -1.676 (-2.074to-1.277) | 52 (46 - 57) | 3.158 (2.823 - 3.504) | 33 (27 - 41) | 1.134 (0.905 - 1.394) | -2.817 (-3.197to-2.436) | 1394 (1239 - 1555) | 84.247 (75.077 - 93.942) | 819 (654 - 1012) | 31.005 (24.697 - 38.184) | -2.748 (-3.132to-2.362) |
| Saint Kitts and Nevis | Female | 0 (0 - 0) | 0.742 (0.666 - 0.825) | 0 (0 - 0) | 0.335 (0.274 - 0.403) | -2.158 (-2.61to-1.704) | 0 (0 - 0) | 0.683 (0.614 - 0.753) | 0 (0 - 0) | 0.265 (0.222 - 0.309) | -2.578 (-3.044to-2.111) | 3 (3 - 4) | 17.033 (15.36 - 18.926) | 2 (2 - 3) | 5.778 (4.736 - 6.992) | -3.152 (-3.673to-2.627) |
|  | Male | 0 (0 - 0) | 2.679 (2.436 - 2.947) | 1 (1 - 1) | 1.927 (1.516 - 2.325) | -0.633 (-1.225to-0.038) | 0 (0 - 0) | 2.557 (2.326 - 2.794) | 1 (0 - 1) | 1.587 (1.257 - 1.892) | -1.134 (-1.734to-0.529) | 10 (9 - 11) | 66.435 (60.162 - 73.152) | 15 (11 - 18) | 39.701 (30.848 - 48.429) | -1.256 (-1.897to-0.612) |
| Saint Lucia | Female | 0 (0 - 0) | 0.615 (0.566 - 0.666) | 0 (0 - 0) | 0.25 (0.205 - 0.307) | -3.374 (-3.9to-2.845) | 0 (0 - 0) | 0.549 (0.507 - 0.591) | 0 (0 - 0) | 0.188 (0.154 - 0.226) | -3.999 (-4.552to-3.442) | 6 (6 - 7) | 13.086 (12.005 - 14.175) | 6 (5 - 7) | 4.448 (3.645 - 5.427) | -3.813 (-4.365to-3.257) |
|  | Male | 1 (1 - 1) | 3.561 (3.289 - 3.857) | 3 (2 - 3) | 2.289 (1.839 - 2.802) | -1.331 (-1.819to-0.84) | 1 (1 - 1) | 3.302 (3.065 - 3.584) | 2 (2 - 3) | 1.844 (1.489 - 2.234) | -1.798 (-2.325to-1.268) | 32 (30 - 35) | 83.809 (77.502 - 90.774) | 57 (45 - 70) | 47.633 (38.293 - 58.351) | -1.688 (-2.183to-1.19) |
| Saint Vincent and the Grenadines | Female | 0 (0 - 0) | 1.173 (1.06 - 1.277) | 0 (0 - 0) | 0.587 (0.507 - 0.682) | -2.13 (-2.616to-1.642) | 0 (0 - 0) | 1.035 (0.94 - 1.131) | 0 (0 - 0) | 0.457 (0.392 - 0.525) | -2.461 (-2.952to-1.967) | 10 (9 - 11) | 24.923 (22.418 - 27.342) | 8 (7 - 9) | 11.59 (9.901 - 13.364) | -2.385 (-2.843to-1.925) |
|  | Male | 1 (1 - 1) | 3.493 (3.153 - 3.86) | 2 (2 - 3) | 2.993 (2.572 - 3.463) | -0.309 (-0.857-0.242) | 1 (1 - 1) | 3.207 (2.902 - 3.541) | 2 (2 - 2) | 2.524 (2.189 - 2.891) | -0.547 (-1.097-0.005) | 26 (24 - 29) | 82.392 (74.333 - 91.21) | 50 (43 - 58) | 66.54 (57.124 - 77.082) | -0.502 (-1.032-0.031) |
| Suriname | Female | 1 (0 - 1) | 0.446 (0.283 - 0.558) | 1 (1 - 1) | 0.277 (0.196 - 0.399) | -1.157 (-1.38to-0.932) | 1 (0 - 1) | 0.407 (0.261 - 0.511) | 1 (1 - 1) | 0.231 (0.164 - 0.332) | -1.419 (-1.634to-1.203) | 14 (9 - 18) | 10.163 (6.319 - 12.697) | 20 (14 - 29) | 5.82 (4.147 - 8.347) | -1.472 (-1.69to-1.253) |
|  | Male | 1 (1 - 2) | 1.084 (0.869 - 1.322) | 3 (2 - 4) | 0.857 (0.61 - 1.167) | -0.311 (-0.586to-0.036) | 1 (1 - 2) | 1.01 (0.815 - 1.226) | 2 (2 - 3) | 0.745 (0.531 - 1.007) | -0.53 (-0.792to-0.267) | 37 (30 - 46) | 27.524 (21.879 - 33.886) | 66 (46 - 91) | 20.523 (14.539 - 27.823) | -0.54 (-0.811to-0.268) |
| Trinidad and Tobago | Female | 2 (2 - 2) | 0.539 (0.499 - 0.58) | 3 (3 - 4) | 0.347 (0.273 - 0.437) | -1.408 (-1.98to-0.833) | 2 (2 - 2) | 0.47 (0.437 - 0.504) | 3 (2 - 3) | 0.248 (0.198 - 0.312) | -2.071 (-2.63to-1.51) | 50 (47 - 54) | 11.405 (10.598 - 12.208) | 62 (49 - 79) | 6.331 (4.946 - 8.043) | -2.001 (-2.544to-1.454) |
|  | Male | 9 (8 - 9) | 2.163 (1.996 - 2.336) | 14 (11 - 19) | 1.531 (1.13 - 1.998) | -1.06 (-1.498to-0.621) | 8 (7 - 8) | 1.964 (1.811 - 2.118) | 11 (8 - 15) | 1.201 (0.891 - 1.559) | -1.579 (-2to-1.156) | 211 (194 - 229) | 51.039 (47.049 - 55.302) | 309 (226 - 404) | 32.503 (23.951 - 42.504) | -1.485 (-1.892to-1.075) |
| United States Virgin Islands | Female | 1 (0 - 1) | 1.316 (0.866 - 1.686) | 1 (0 - 1) | 0.773 (0.449 - 1.132) | -1.657 (-1.923to-1.39) | 0 (0 - 1) | 1.051 (0.683 - 1.352) | 0 (0 - 1) | 0.506 (0.3 - 0.721) | -2.226 (-2.465to-1.986) | 12 (8 - 15) | 24.889 (16.553 - 32.011) | 11 (6 - 16) | 12.929 (7.425 - 19.16) | -1.937 (-2.214to-1.659) |
|  | Male | 1 (1 - 1) | 2.23 (1.716 - 2.84) | 1 (1 - 2) | 1.478 (1.059 - 2.092) | -1.272 (-1.442to-1.102) | 1 (1 - 1) | 1.867 (1.452 - 2.38) | 1 (1 - 1) | 1.101 (0.804 - 1.541) | -1.632 (-1.8to-1.463) | 22 (17 - 28) | 49.441 (37.847 - 63.896) | 21 (15 - 30) | 29.228 (21.018 - 41.608) | -1.528 (-1.682to-1.374) |
| Colombia | Female | 31 (27 - 36) | 0.337 (0.291 - 0.386) | 73 (59 - 90) | 0.242 (0.196 - 0.298) | -1.708 (-2.208to-1.205) | 26 (22 - 29) | 0.29 (0.249 - 0.33) | 46 (37 - 56) | 0.149 (0.121 - 0.182) | -2.706 (-3.174to-2.235) | 715 (625 - 822) | 7.195 (6.252 - 8.253) | 1090 (885 - 1335) | 3.634 (2.953 - 4.45) | -2.82 (-3.316to-2.322) |
|  | Male | 62 (56 - 70) | 0.716 (0.641 - 0.796) | 150 (123 - 184) | 0.604 (0.498 - 0.738) | -1.037 (-1.372to-0.701) | 55 (49 - 61) | 0.658 (0.589 - 0.733) | 108 (88 - 131) | 0.438 (0.359 - 0.531) | -1.799 (-2.126to-1.47) | 1561 (1391 - 1757) | 16.659 (14.865 - 18.657) | 2646 (2149 - 3269) | 10.504 (8.555 - 12.937) | -1.959 (-2.282to-1.635) |
| Costa Rica | Female | 4 (3 - 4) | 0.411 (0.359 - 0.463) | 10 (8 - 12) | 0.325 (0.274 - 0.385) | -1.597 (-2.119to-1.073) | 3 (2 - 3) | 0.284 (0.247 - 0.32) | 6 (5 - 7) | 0.182 (0.154 - 0.216) | -2.144 (-2.614to-1.673) | 70 (62 - 79) | 7.338 (6.462 - 8.221) | 132 (113 - 156) | 4.448 (3.798 - 5.261) | -2.499 (-3.032to-1.963) |
|  | Male | 12 (11 - 13) | 1.404 (1.247 - 1.566) | 37 (31 - 43) | 1.452 (1.244 - 1.676) | -0.181 (-0.397-0.036) | 9 (8 - 10) | 1.094 (0.978 - 1.221) | 24 (21 - 28) | 0.961 (0.835 - 1.109) | -0.646 (-0.833to-0.458) | 253 (224 - 283) | 28.578 (25.308 - 31.974) | 610 (522 - 708) | 23.836 (20.374 - 27.649) | -0.966 (-1.183to-0.749) |
| El Salvador | Female | 5 (3 - 6) | 0.284 (0.204 - 0.346) | 9 (7 - 13) | 0.258 (0.19 - 0.371) | -0.34 (-0.472to-0.208) | 4 (3 - 5) | 0.259 (0.188 - 0.317) | 6 (5 - 9) | 0.177 (0.131 - 0.251) | -1.254 (-1.435to-1.074) | 109 (78 - 132) | 6.522 (4.654 - 7.878) | 156 (115 - 222) | 4.401 (3.236 - 6.261) | -1.302 (-1.484to-1.12) |
|  | Male | 10 (9 - 12) | 0.728 (0.612 - 0.872) | 21 (16 - 28) | 0.829 (0.633 - 1.097) | 0.169 (-0.072-0.41) | 9 (8 - 11) | 0.69 (0.581 - 0.829) | 16 (12 - 21) | 0.628 (0.481 - 0.825) | -0.567 (-0.817to-0.318) | 263 (222 - 316) | 18.105 (15.22 - 21.722) | 427 (325 - 562) | 16.741 (12.792 - 22.078) | -0.5 (-0.775to-0.225) |
| Guatemala | Female | 7 (7 - 8) | 0.414 (0.38 - 0.446) | 17 (14 - 19) | 0.276 (0.238 - 0.319) | -1.782 (-2.25to-1.312) | 7 (6 - 7) | 0.411 (0.379 - 0.442) | 13 (11 - 15) | 0.23 (0.2 - 0.264) | -2.328 (-2.79to-1.863) | 200 (185 - 215) | 10.039 (9.285 - 10.83) | 362 (308 - 420) | 5.766 (4.915 - 6.676) | -2.239 (-2.744to-1.732) |
|  | Male | 14 (13 - 15) | 0.862 (0.805 - 0.917) | 29 (25 - 35) | 0.571 (0.479 - 0.682) | -1.821 (-2.047to-1.593) | 14 (13 - 15) | 0.884 (0.824 - 0.942) | 26 (22 - 31) | 0.515 (0.436 - 0.609) | -2.21 (-2.437to-1.983) | 398 (372 - 424) | 21.347 (19.959 - 22.735) | 694 (584 - 824) | 12.924 (10.832 - 15.351) | -2.096 (-2.338to-1.854) |
| Honduras | Female | 4 (3 - 6) | 0.351 (0.256 - 0.515) | 15 (9 - 23) | 0.417 (0.266 - 0.634) | 0.541 (0.44-0.643) | 3 (3 - 5) | 0.331 (0.242 - 0.483) | 12 (8 - 18) | 0.359 (0.236 - 0.536) | 0.265 (0.154-0.376) | 105 (76 - 148) | 8.739 (6.227 - 12.612) | 342 (213 - 529) | 9.121 (5.766 - 14.036) | 0.117 (0.028-0.207) |
|  | Male | 4 (3 - 6) | 0.45 (0.341 - 0.578) | 18 (13 - 27) | 0.63 (0.46 - 0.92) | 1.363 (1.215-1.512) | 4 (3 - 6) | 0.447 (0.343 - 0.573) | 17 (12 - 24) | 0.594 (0.425 - 0.865) | 1.209 (1.055-1.363) | 120 (91 - 155) | 11.283 (8.572 - 14.555) | 437 (317 - 636) | 14.182 (10.403 - 20.565) | 0.978 (0.851-1.105) |
| Mexico | Female | 40 (38 - 41) | 0.186 (0.179 - 0.194) | 104 (88 - 120) | 0.154 (0.129 - 0.177) | -1.284 (-1.758to-0.807) | 35 (33 - 36) | 0.174 (0.167 - 0.182) | 74 (63 - 85) | 0.112 (0.095 - 0.128) | -1.979 (-2.441to-1.515) | 908 (879 - 940) | 3.878 (3.739 - 4.019) | 1849 (1555 - 2147) | 2.674 (2.254 - 3.096) | -1.808 (-2.284to-1.33) |
|  | Male | 110 (106 - 114) | 0.54 (0.52 - 0.56) | 290 (241 - 345) | 0.487 (0.406 - 0.577) | -0.577 (-0.794to-0.36) | 101 (97 - 104) | 0.512 (0.492 - 0.531) | 227 (190 - 269) | 0.392 (0.329 - 0.463) | -1.053 (-1.263to-0.842) | 2678 (2584 - 2775) | 12.395 (11.952 - 12.845) | 5894 (4901 - 7035) | 9.629 (8.022 - 11.479) | -1.015 (-1.24to-0.789) |
| Nicaragua | Female | 1 (1 - 2) | 0.153 (0.109 - 0.195) | 4 (3 - 5) | 0.141 (0.099 - 0.196) | -0.109 (-0.231-0.013) | 1 (1 - 1) | 0.136 (0.098 - 0.174) | 3 (2 - 4) | 0.101 (0.072 - 0.138) | -0.793 (-0.884to-0.703) | 32 (23 - 40) | 3.45 (2.435 - 4.363) | 72 (50 - 99) | 2.536 (1.785 - 3.489) | -0.882 (-0.984to-0.78) |
|  | Male | 4 (3 - 5) | 0.594 (0.478 - 0.721) | 13 (10 - 18) | 0.593 (0.437 - 0.795) | 0.236 (0.064-0.407) | 4 (3 - 5) | 0.57 (0.458 - 0.692) | 10 (8 - 14) | 0.482 (0.358 - 0.64) | -0.34 (-0.51to-0.169) | 106 (84 - 129) | 13.776 (10.975 - 16.818) | 282 (207 - 383) | 12.036 (8.833 - 16.271) | -0.23 (-0.396to-0.063) |
| Panama | Female | 4 (3 - 4) | 0.499 (0.452 - 0.545) | 11 (9 - 13) | 0.462 (0.377 - 0.553) | -0.277 (-0.702-0.15) | 3 (3 - 3) | 0.407 (0.368 - 0.445) | 7 (5 - 8) | 0.29 (0.233 - 0.347) | -0.99 (-1.365to-0.615) | 78 (71 - 84) | 9.855 (8.956 - 10.761) | 161 (130 - 192) | 7.097 (5.772 - 8.481) | -0.953 (-1.346to-0.558) |
|  | Male | 12 (11 - 13) | 1.565 (1.441 - 1.698) | 29 (22 - 35) | 1.351 (1.044 - 1.659) | -0.251 (-0.494to-0.006) | 10 (9 - 11) | 1.394 (1.283 - 1.513) | 21 (16 - 26) | 1.001 (0.779 - 1.22) | -0.762 (-0.994to-0.529) | 256 (236 - 278) | 33.293 (30.718 - 36.066) | 512 (393 - 631) | 23.769 (18.286 - 29.236) | -0.787 (-1.022to-0.552) |
| Venezuela  (Bolivarian Republic of) | Female | 20 (19 - 22) | 0.396 (0.366 - 0.425) | 48 (35 - 62) | 0.293 (0.218 - 0.383) | -1.292 (-1.626to-0.957) | 17 (16 - 18) | 0.347 (0.319 - 0.374) | 34 (26 - 44) | 0.209 (0.159 - 0.271) | -1.967 (-2.339to-1.594) | 460 (431 - 490) | 8.37 (7.8 - 8.931) | 851 (636 - 1111) | 5.233 (3.916 - 6.84) | -1.898 (-2.27to-1.524) |
|  | Male | 54 (50 - 57) | 1.157 (1.076 - 1.235) | 161 (115 - 222) | 1.139 (0.818 - 1.562) | 0.072 (-0.145-0.289) | 47 (44 - 51) | 1.057 (0.987 - 1.128) | 125 (90 - 171) | 0.911 (0.658 - 1.24) | -0.414 (-0.635to-0.193) | 1331 (1245 - 1423) | 27.04 (25.193 - 28.881) | 3350 (2389 - 4630) | 23.041 (16.47 - 31.756) | -0.434 (-0.67to-0.196) |
| Brazil | Female | 310 (292 - 329) | 0.637 (0.597 - 0.679) | 848 (781 - 908) | 0.611 (0.562 - 0.654) | -0.14 (-0.266to-0.013) | 255 (240 - 271) | 0.548 (0.513 - 0.585) | 580 (529 - 623) | 0.417 (0.38 - 0.448) | -0.798 (-0.941to-0.655) | 7304 (6917 - 7748) | 14.202 (13.422 - 15.073) | 15296 (14164 - 16345) | 11.062 (10.246 - 11.818) | -0.815 (-0.958to-0.672) |
|  | Male | 1577 (1493 - 1665) | 3.471 (3.284 - 3.655) | 4361 (4046 - 4658) | 3.663 (3.397 - 3.911) | 0.051 (-0.113-0.215) | 1337 (1268 - 1410) | 3.047 (2.88 - 3.211) | 3188 (2958 - 3400) | 2.725 (2.518 - 2.908) | -0.445 (-0.605to-0.284) | 42081 (39889 - 44323) | 88.104 (83.484 - 92.77) | 94124 (87238 - 100803) | 77.845 (72.158 - 83.272) | -0.535 (-0.722to-0.348) |
| Paraguay | Female | 3 (2 - 4) | 0.255 (0.188 - 0.363) | 10 (6 - 14) | 0.315 (0.21 - 0.436) | 0.812 (0.706-0.918) | 2 (2 - 4) | 0.214 (0.157 - 0.304) | 7 (5 - 9) | 0.223 (0.151 - 0.306) | 0.34 (0.198-0.483) | 69 (51 - 96) | 5.586 (4.129 - 7.837) | 181 (123 - 250) | 5.735 (3.9 - 7.926) | 0.251 (0.103-0.4) |
|  | Male | 16 (12 - 20) | 1.409 (1.094 - 1.812) | 71 (48 - 103) | 2.424 (1.655 - 3.471) | 1.71 (1.56-1.861) | 13 (10 - 17) | 1.237 (0.959 - 1.577) | 54 (36 - 77) | 1.874 (1.287 - 2.672) | 1.336 (1.168-1.504) | 396 (307 - 516) | 34.448 (26.758 - 44.642) | 1574 (1051 - 2295) | 52.047 (34.76 - 75.416) | 1.293 (1.112-1.474) |
| Afghanistan | Female | 16 (7 - 38) | 0.47 (0.221 - 1.082) | 25 (12 - 59) | 0.424 (0.229 - 0.908) | -0.428 (-0.498to-0.357) | 15 (7 - 35) | 0.464 (0.224 - 1.018) | 21 (11 - 49) | 0.396 (0.217 - 0.811) | -0.599 (-0.64to-0.558) | 440 (194 - 1126) | 12.368 (5.532 - 30.737) | 712 (343 - 1763) | 10.399 (5.33 - 23.76) | -0.667 (-0.72to-0.613) |
|  | Male | 11 (5 - 20) | 0.285 (0.141 - 0.533) | 12 (6 - 21) | 0.241 (0.133 - 0.405) | -0.452 (-0.624to-0.28) | 10 (5 - 20) | 0.286 (0.146 - 0.533) | 11 (6 - 18) | 0.234 (0.13 - 0.382) | -0.554 (-0.703to-0.406) | 295 (138 - 566) | 7.759 (3.666 - 14.843) | 356 (187 - 631) | 6.174 (3.341 - 10.655) | -0.684 (-0.828to-0.54) |
| Algeria | Female | 24 (16 - 32) | 0.4 (0.269 - 0.526) | 84 (49 - 122) | 0.454 (0.269 - 0.653) | 0.857 (0.716-0.998) | 19 (13 - 25) | 0.344 (0.235 - 0.453) | 47 (28 - 68) | 0.284 (0.176 - 0.407) | 0.025 (-0.164-0.214) | 591 (380 - 766) | 8.794 (5.866 - 11.438) | 1423 (852 - 2026) | 7.308 (4.382 - 10.434) | -0.267 (-0.383to-0.151) |
|  | Male | 72 (55 - 92) | 1.153 (0.888 - 1.476) | 244 (174 - 342) | 1.265 (0.916 - 1.774) | 0.423 (0.351-0.495) | 62 (47 - 80) | 1.031 (0.803 - 1.309) | 165 (118 - 232) | 0.901 (0.648 - 1.252) | -0.292 (-0.355to-0.23) | 1834 (1383 - 2387) | 27.989 (21.175 - 36.275) | 4789 (3373 - 6781) | 23.96 (17.05 - 33.782) | -0.391 (-0.449to-0.332) |
| Bahrain | Female | 0 (0 - 0) | 0.321 (0.248 - 0.468) | 2 (1 - 3) | 0.457 (0.301 - 0.636) | 1.883 (1.307-2.462) | 0 (0 - 0) | 0.257 (0.198 - 0.368) | 1 (1 - 1) | 0.241 (0.159 - 0.326) | 0.443 (-0.178-1.067) | 6 (5 - 9) | 6.35 (4.845 - 9.263) | 25 (16 - 36) | 5.725 (3.75 - 8.026) | 0.281 (-0.297-0.862) |
|  | Male | 1 (1 - 1) | 0.804 (0.654 - 1.008) | 4 (2 - 6) | 0.691 (0.468 - 1.075) | -0.526 (-0.819to-0.233) | 1 (0 - 1) | 0.737 (0.599 - 0.927) | 2 (1 - 3) | 0.475 (0.326 - 0.729) | -1.503 (-1.844to-1.16) | 19 (15 - 24) | 16.419 (13.213 - 20.625) | 64 (41 - 103) | 10.032 (6.644 - 15.695) | -1.746 (-2.06to-1.431) |
| Egypt | Female | 20 (13 - 54) | 0.157 (0.103 - 0.422) | 88 (63 - 135) | 0.305 (0.22 - 0.455) | 3.003 (2.46-3.549) | 17 (11 - 46) | 0.149 (0.096 - 0.403) | 56 (40 - 85) | 0.231 (0.168 - 0.332) | 2.483 (1.891-3.078) | 533 (354 - 1385) | 3.554 (2.338 - 9.377) | 1734 (1237 - 2649) | 5.333 (3.807 - 7.924) | 2.163 (1.615-2.713) |
|  | Male | 32 (26 - 40) | 0.225 (0.182 - 0.281) | 99 (71 - 133) | 0.279 (0.202 - 0.366) | 0.768 (0.566-0.97) | 29 (23 - 36) | 0.215 (0.176 - 0.268) | 74 (54 - 99) | 0.224 (0.165 - 0.29) | 0.286 (0.063-0.509) | 886 (716 - 1093) | 5.589 (4.494 - 6.942) | 2202 (1590 - 2969) | 5.716 (4.144 - 7.589) | 0.204 (-0.034-0.443) |
| Iran  (Islamic Republic of) | Female | 27 (22 - 38) | 0.217 (0.168 - 0.286) | 88 (73 - 139) | 0.223 (0.185 - 0.337) | 0.004 (-0.139-0.148) | 21 (17 - 28) | 0.186 (0.14 - 0.233) | 49 (40 - 69) | 0.134 (0.106 - 0.181) | -1.057 (-1.162to-0.952) | 639 (507 - 885) | 4.534 (3.598 - 6.117) | 1291 (1067 - 1977) | 3.139 (2.571 - 4.636) | -1.188 (-1.304to-1.072) |
|  | Male | 24 (17 - 30) | 0.171 (0.126 - 0.215) | 76 (65 - 91) | 0.191 (0.163 - 0.228) | 0.538 (0.381-0.695) | 20 (15 - 26) | 0.156 (0.114 - 0.196) | 50 (43 - 60) | 0.132 (0.113 - 0.157) | -0.313 (-0.446to-0.179) | 601 (435 - 754) | 3.917 (2.853 - 4.926) | 1367 (1171 - 1633) | 3.274 (2.812 - 3.907) | -0.349 (-0.474to-0.225) |
| Iraq | Female | 14 (10 - 20) | 0.322 (0.22 - 0.456) | 53 (35 - 76) | 0.385 (0.261 - 0.552) | 0.651 (0.546-0.756) | 11 (7 - 15) | 0.255 (0.174 - 0.372) | 29 (20 - 41) | 0.226 (0.156 - 0.321) | -0.395 (-0.458to-0.333) | 343 (235 - 471) | 7.526 (5.154 - 10.446) | 939 (623 - 1341) | 6.437 (4.322 - 9.045) | -0.501 (-0.575to-0.427) |
|  | Male | 15 (10 - 20) | 0.361 (0.253 - 0.501) | 64 (44 - 90) | 0.487 (0.34 - 0.67) | 1.127 (0.939-1.316) | 13 (9 - 17) | 0.313 (0.223 - 0.433) | 42 (29 - 59) | 0.35 (0.248 - 0.475) | 0.446 (0.311-0.582) | 389 (277 - 533) | 9.022 (6.354 - 12.379) | 1305 (889 - 1856) | 9.25 (6.403 - 12.779) | 0.181 (0.069-0.294) |
| Jordan | Female | 2 (1 - 3) | 0.269 (0.185 - 0.355) | 10 (6 - 15) | 0.264 (0.164 - 0.376) | -0.062 (-0.282-0.158) | 1 (1 - 2) | 0.207 (0.145 - 0.273) | 5 (3 - 7) | 0.142 (0.089 - 0.204) | -1.356 (-1.657to-1.053) | 45 (31 - 62) | 5.67 (3.874 - 7.577) | 151 (92 - 217) | 3.639 (2.22 - 5.141) | -1.661 (-1.927to-1.395) |
|  | Male | 3 (2 - 4) | 0.42 (0.313 - 0.552) | 20 (13 - 31) | 0.45 (0.303 - 0.671) | 0.522 (0.341-0.703) | 3 (2 - 3) | 0.355 (0.268 - 0.462) | 12 (8 - 18) | 0.287 (0.196 - 0.421) | -0.558 (-0.687to-0.428) | 81 (59 - 108) | 9.719 (7.192 - 12.893) | 364 (235 - 560) | 7.554 (4.989 - 11.467) | -0.725 (-0.864to-0.585) |
| Kuwait | Female | 2 (2 - 2) | 0.745 (0.659 - 0.844) | 3 (2 - 4) | 0.165 (0.133 - 0.201) | -3.488 (-5.552to-1.379) | 1 (1 - 1) | 0.402 (0.354 - 0.456) | 1 (1 - 1) | 0.068 (0.054 - 0.082) | -4.46 (-6.519to-2.354) | 33 (30 - 37) | 11.226 (9.88 - 12.669) | 31 (25 - 38) | 1.666 (1.352 - 2.02) | -4.958 (-7.002to-2.87) |
|  | Male | 4 (4 - 5) | 0.858 (0.732 - 0.986) | 6 (5 - 8) | 0.312 (0.238 - 0.395) | -2.289 (-3.082to-1.489) | 2 (2 - 3) | 0.557 (0.475 - 0.636) | 3 (2 - 3) | 0.167 (0.129 - 0.209) | -3.118 (-3.883to-2.347) | 82 (70 - 94) | 15.656 (13.303 - 18.042) | 81 (62 - 104) | 3.877 (2.959 - 4.916) | -3.543 (-4.335to-2.745) |
| Lebanon | Female | 2 (1 - 3) | 0.21 (0.101 - 0.293) | 7 (4 - 9) | 0.213 (0.123 - 0.292) | 0.029 (-0.135-0.194) | 2 (1 - 2) | 0.155 (0.078 - 0.218) | 3 (2 - 4) | 0.1 (0.06 - 0.135) | -1.49 (-1.673to-1.306) | 51 (24 - 75) | 4.25 (1.994 - 6.085) | 82 (49 - 113) | 2.586 (1.524 - 3.579) | -1.73 (-1.887to-1.573) |
|  | Male | 6 (4 - 9) | 0.515 (0.317 - 0.735) | 16 (11 - 21) | 0.586 (0.415 - 0.804) | 1.203 (0.909-1.497) | 5 (3 - 7) | 0.421 (0.26 - 0.603) | 9 (6 - 12) | 0.335 (0.238 - 0.461) | -0.051 (-0.317-0.217) | 138 (82 - 201) | 11.673 (7.058 - 16.81) | 234 (166 - 319) | 8.854 (6.25 - 12.075) | -0.223 (-0.491-0.046) |
| Libya | Female | 3 (2 - 4) | 0.279 (0.179 - 0.409) | 9 (5 - 13) | 0.296 (0.175 - 0.431) | 0.422 (0.294-0.551) | 2 (1 - 3) | 0.209 (0.137 - 0.3) | 5 (3 - 7) | 0.179 (0.111 - 0.255) | -0.384 (-0.45to-0.319) | 59 (38 - 85) | 5.962 (3.852 - 8.679) | 161 (91 - 240) | 5.078 (2.988 - 7.445) | -0.442 (-0.495to-0.388) |
|  | Male | 5 (3 - 7) | 0.466 (0.304 - 0.64) | 18 (11 - 26) | 0.57 (0.376 - 0.838) | 0.945 (0.734-1.156) | 4 (3 - 6) | 0.389 (0.254 - 0.539) | 12 (8 - 17) | 0.403 (0.268 - 0.598) | 0.283 (0.112-0.454) | 127 (82 - 173) | 10.957 (7.071 - 15.051) | 378 (242 - 565) | 11.454 (7.523 - 17.037) | 0.314 (0.142-0.487) |
| Morocco | Female | 10 (6 - 14) | 0.13 (0.076 - 0.182) | 28 (15 - 43) | 0.153 (0.084 - 0.23) | 0.563 (0.51-0.616) | 8 (5 - 12) | 0.113 (0.067 - 0.157) | 19 (10 - 29) | 0.106 (0.06 - 0.157) | -0.103 (-0.137to-0.068) | 255 (150 - 354) | 3.217 (1.89 - 4.495) | 582 (320 - 885) | 3.074 (1.701 - 4.646) | -0.086 (-0.122to-0.05) |
|  | Male | 28 (20 - 38) | 0.378 (0.266 - 0.517) | 84 (56 - 119) | 0.459 (0.309 - 0.644) | 0.722 (0.512-0.933) | 25 (18 - 34) | 0.349 (0.248 - 0.471) | 66 (44 - 91) | 0.369 (0.253 - 0.509) | 0.314 (0.145-0.484) | 747 (532 - 1023) | 9.837 (6.973 - 13.514) | 1869 (1232 - 2695) | 9.969 (6.67 - 14.194) | 0.157 (-0.001-0.315) |
| Oman | Female | 1 (0 - 1) | 0.234 (0.151 - 0.326) | 2 (2 - 4) | 0.243 (0.152 - 0.336) | 0.302 (0.119-0.485) | 1 (0 - 1) | 0.175 (0.112 - 0.244) | 1 (1 - 1) | 0.115 (0.073 - 0.159) | -1.039 (-1.271to-0.806) | 17 (11 - 24) | 4.916 (3.119 - 6.928) | 34 (21 - 49) | 3.191 (1.977 - 4.479) | -1.114 (-1.35to-0.877) |
|  | Male | 2 (1 - 3) | 0.525 (0.327 - 0.767) | 8 (5 - 12) | 0.567 (0.394 - 0.823) | 0.627 (0.409-0.845) | 2 (1 - 3) | 0.441 (0.278 - 0.643) | 4 (3 - 6) | 0.348 (0.246 - 0.498) | -0.306 (-0.549to-0.064) | 56 (34 - 83) | 12.067 (7.437 - 17.953) | 141 (94 - 217) | 8.984 (6.132 - 13.253) | -0.528 (-0.8to-0.256) |
| Palestine | Female | 1 (0 - 1) | 0.149 (0.098 - 0.21) | 2 (2 - 3) | 0.159 (0.109 - 0.208) | 0.395 (0.23-0.56) | 1 (0 - 1) | 0.125 (0.083 - 0.176) | 1 (1 - 2) | 0.103 (0.071 - 0.134) | -0.361 (-0.596to-0.126) | 16 (11 - 24) | 3.132 (2.076 - 4.59) | 38 (26 - 51) | 2.53 (1.753 - 3.317) | -0.463 (-0.674to-0.252) |
|  | Male | 1 (0 - 1) | 0.172 (0.11 - 0.261) | 2 (1 - 2) | 0.147 (0.114 - 0.191) | -0.658 (-0.869to-0.446) | 1 (0 - 1) | 0.161 (0.106 - 0.244) | 1 (1 - 2) | 0.117 (0.09 - 0.149) | -1.141 (-1.421to-0.861) | 16 (10 - 25) | 3.761 (2.368 - 5.903) | 36 (27 - 48) | 2.628 (2.021 - 3.42) | -1.306 (-1.538to-1.074) |
| Qatar | Female | 0 (0 - 0) | 0.315 (0.148 - 0.419) | 1 (0 - 1) | 0.187 (0.124 - 0.274) | -1.798 (-2.005to-1.59) | 0 (0 - 0) | 0.235 (0.109 - 0.308) | 0 (0 - 0) | 0.082 (0.054 - 0.115) | -3.678 (-3.889to-3.466) | 3 (2 - 4) | 5.68 (2.753 - 7.479) | 8 (5 - 14) | 1.878 (1.254 - 2.769) | -3.787 (-3.961to-3.614) |
|  | Male | 1 (1 - 1) | 1.221 (0.919 - 1.627) | 6 (4 - 10) | 0.789 (0.511 - 1.259) | -1.619 (-1.837to-1.401) | 1 (1 - 1) | 1.06 (0.803 - 1.403) | 2 (2 - 4) | 0.461 (0.299 - 0.722) | -3.062 (-3.322to-2.801) | 24 (17 - 32) | 23.355 (17.338 - 31.446) | 89 (57 - 140) | 9.617 (6.138 - 15.587) | -3.151 (-3.329to-2.973) |
| Saudi Arabia | Female | 13 (8 - 21) | 0.459 (0.294 - 0.801) | 71 (45 - 113) | 0.618 (0.404 - 0.991) | 1.075 (0.971-1.18) | 10 (6 - 17) | 0.376 (0.245 - 0.67) | 30 (20 - 48) | 0.311 (0.201 - 0.52) | -0.609 (-0.809to-0.408) | 319 (204 - 525) | 10.673 (6.857 - 17.838) | 1123 (709 - 1860) | 8.952 (5.88 - 14.16) | -0.533 (-0.731to-0.333) |
|  | Male | 13 (9 - 18) | 0.352 (0.248 - 0.484) | 66 (46 - 95) | 0.421 (0.3 - 0.595) | 0.481 (0.345-0.618) | 11 (8 - 16) | 0.316 (0.223 - 0.437) | 36 (25 - 51) | 0.268 (0.191 - 0.38) | -0.692 (-0.922to-0.462) | 360 (248 - 502) | 8.646 (6.038 - 12.002) | 1259 (865 - 1789) | 7.242 (5.056 - 10.282) | -0.732 (-0.939to-0.525) |
| Sudan | Female | 12 (7 - 21) | 0.255 (0.155 - 0.427) | 25 (15 - 38) | 0.239 (0.149 - 0.356) | -0.295 (-0.361to-0.228) | 11 (6 - 18) | 0.24 (0.147 - 0.386) | 18 (11 - 27) | 0.192 (0.122 - 0.276) | -0.769 (-0.789to-0.749) | 319 (183 - 588) | 6.323 (3.749 - 11.147) | 576 (346 - 896) | 4.976 (3.121 - 7.465) | -0.825 (-0.853to-0.796) |
|  | Male | 13 (7 - 26) | 0.256 (0.151 - 0.514) | 24 (15 - 37) | 0.22 (0.14 - 0.333) | -0.555 (-0.664to-0.445) | 12 (7 - 24) | 0.248 (0.147 - 0.485) | 20 (13 - 30) | 0.193 (0.123 - 0.29) | -0.849 (-0.926to-0.772) | 355 (204 - 701) | 6.701 (3.858 - 13.164) | 599 (366 - 920) | 4.984 (3.12 - 7.561) | -1.009 (-1.077to-0.941) |
| Syrian Arab Republic | Female | 5 (4 - 7) | 0.198 (0.145 - 0.271) | 14 (10 - 23) | 0.218 (0.149 - 0.334) | 0.161 (0.078-0.243) | 4 (3 - 6) | 0.169 (0.124 - 0.23) | 8 (5 - 12) | 0.134 (0.094 - 0.196) | -0.879 (-0.987to-0.771) | 131 (91 - 176) | 4.4 (3.166 - 5.89) | 228 (154 - 357) | 3.303 (2.283 - 5.048) | -1.094 (-1.206to-0.982) |
|  | Male | 11 (9 - 15) | 0.406 (0.302 - 0.531) | 32 (22 - 45) | 0.461 (0.321 - 0.647) | 0.222 (0.117-0.327) | 10 (8 - 13) | 0.371 (0.279 - 0.484) | 22 (15 - 31) | 0.332 (0.233 - 0.466) | -0.565 (-0.669to-0.461) | 293 (221 - 386) | 9.579 (7.176 - 12.536) | 593 (410 - 844) | 8.167 (5.677 - 11.602) | -0.738 (-0.835to-0.641) |
| Tunisia | Female | 13 (9 - 18) | 0.514 (0.339 - 0.69) | 42 (25 - 61) | 0.59 (0.347 - 0.864) | 0.358 (0.319-0.397) | 9 (6 - 13) | 0.383 (0.253 - 0.515) | 21 (13 - 31) | 0.304 (0.182 - 0.454) | -0.804 (-0.846to-0.761) | 286 (192 - 384) | 10.528 (6.972 - 14.164) | 594 (353 - 893) | 8.365 (4.989 - 12.463) | -0.814 (-0.857to-0.77) |
|  | Male | 16 (12 - 21) | 0.602 (0.455 - 0.784) | 55 (35 - 82) | 0.808 (0.518 - 1.21) | 0.764 (0.697-0.83) | 13 (10 - 17) | 0.502 (0.383 - 0.653) | 34 (21 - 50) | 0.513 (0.328 - 0.751) | -0.111 (-0.198to-0.025) | 377 (281 - 498) | 13.536 (10.118 - 17.786) | 946 (589 - 1418) | 13.769 (8.596 - 20.474) | -0.147 (-0.232to-0.061) |
| Türkiye | Female | 45 (27 - 61) | 0.233 (0.145 - 0.317) | 113 (78 - 154) | 0.228 (0.157 - 0.31) | -0.125 (-0.262-0.013) | 36 (22 - 49) | 0.198 (0.123 - 0.272) | 60 (41 - 79) | 0.123 (0.085 - 0.162) | -1.678 (-1.844to-1.511) | 1096 (664 - 1520) | 5.402 (3.281 - 7.4) | 1559 (1072 - 2115) | 3.144 (2.162 - 4.253) | -1.964 (-2.092to-1.836) |
|  | Male | 69 (48 - 95) | 0.402 (0.289 - 0.543) | 189 (137 - 253) | 0.42 (0.307 - 0.557) | 0.005 (-0.144-0.154) | 60 (43 - 84) | 0.372 (0.267 - 0.502) | 119 (86 - 156) | 0.276 (0.203 - 0.358) | -1.207 (-1.357to-1.056) | 1786 (1263 - 2516) | 9.639 (6.802 - 13.432) | 3154 (2286 - 4227) | 6.822 (4.955 - 9.005) | -1.341 (-1.481to-1.202) |
| United Arab Emirates | Female | 3 (2 - 4) | 1.338 (0.855 - 2.049) | 17 (12 - 28) | 2.424 (1.758 - 3.324) | 3.303 (2.668-3.942) | 2 (1 - 3) | 1.104 (0.719 - 1.644) | 9 (6 - 14) | 1.816 (1.325 - 2.464) | 3.255 (2.588-3.926) | 64 (40 - 96) | 28.644 (18.133 - 43.995) | 305 (212 - 507) | 34.528 (25.035 - 47.659) | 1.759 (1.259-2.261) |
|  | Male | 4 (2 - 7) | 1.05 (0.537 - 1.75) | 27 (19 - 37) | 0.636 (0.465 - 0.841) | -0.646 (-1.149to-0.14) | 3 (2 - 5) | 0.954 (0.491 - 1.583) | 16 (11 - 22) | 0.499 (0.367 - 0.655) | -0.987 (-1.55to-0.421) | 118 (63 - 194) | 24.095 (12.212 - 40.818) | 594 (416 - 817) | 11.476 (8.382 - 14.988) | -1.566 (-2.08to-1.049) |
| Yemen | Female | 7 (4 - 12) | 0.267 (0.16 - 0.462) | 19 (11 - 29) | 0.248 (0.152 - 0.372) | -0.27 (-0.312to-0.228) | 6 (4 - 11) | 0.252 (0.153 - 0.428) | 15 (9 - 23) | 0.216 (0.134 - 0.315) | -0.59 (-0.637to-0.542) | 180 (102 - 336) | 6.48 (3.73 - 11.686) | 456 (266 - 714) | 5.399 (3.268 - 8.182) | -0.675 (-0.729to-0.621) |
|  | Male | 8 (4 - 12) | 0.309 (0.165 - 0.489) | 19 (10 - 32) | 0.257 (0.142 - 0.427) | -0.51 (-0.594to-0.425) | 7 (4 - 12) | 0.3 (0.161 - 0.477) | 16 (9 - 27) | 0.237 (0.133 - 0.402) | -0.703 (-0.779to-0.627) | 225 (121 - 359) | 8.054 (4.334 - 12.822) | 484 (274 - 830) | 6.064 (3.385 - 10.279) | -0.883 (-0.951to-0.814) |
| Bangladesh | Female | 332 (230 - 448) | 1.451 (1.017 - 1.953) | 1092 (693 - 1635) | 1.508 (0.965 - 2.26) | 0.123 (0.011-0.236) | 299 (210 - 403) | 1.354 (0.961 - 1.81) | 785 (504 - 1160) | 1.122 (0.728 - 1.649) | -0.605 (-0.668to-0.542) | 9713 (6648 - 13437) | 38.971 (27.067 - 52.914) | 24408 (15127 - 36579) | 32.542 (20.424 - 48.674) | -0.569 (-0.637to-0.501) |
|  | Male | 1412 (900 - 1993) | 5.173 (3.312 - 7.321) | 3949 (2322 - 6606) | 5.408 (3.199 - 8.984) | 0.14 (0.039-0.24) | 1331 (853 - 1885) | 4.954 (3.127 - 7.056) | 3254 (1906 - 5369) | 4.516 (2.664 - 7.382) | -0.315 (-0.439to-0.192) | 41126 (26356 - 57830) | 145.305 (92.924 - 204.727) | 93901 (54681 - 160641) | 126.798 (74.026 - 216.033) | -0.426 (-0.518to-0.333) |
| Bhutan | Female | 2 (1 - 3) | 1.402 (0.922 - 2.074) | 5 (3 - 8) | 1.606 (1.01 - 2.624) | 0.385 (0.311-0.459) | 2 (1 - 2) | 1.306 (0.873 - 1.896) | 4 (2 - 6) | 1.24 (0.795 - 2.015) | -0.223 (-0.261to-0.185) | 54 (36 - 80) | 37.475 (24.706 - 55.142) | 113 (69 - 185) | 35.19 (21.821 - 57.602) | -0.272 (-0.314to-0.231) |
|  | Male | 6 (3 - 9) | 4.088 (2.344 - 6.449) | 16 (10 - 23) | 4.847 (3.206 - 7) | 0.608 (0.502-0.714) | 5 (3 - 9) | 3.94 (2.284 - 6.299) | 13 (9 - 19) | 4.166 (2.788 - 6.043) | 0.245 (0.173-0.316) | 173 (94 - 283) | 114.244 (63.876 - 185.632) | 379 (244 - 561) | 114.352 (74.44 - 169.479) | 0.037 (-0.035-0.109) |
| India | Female | 3199 (2348 - 4256) | 1.275 (0.938 - 1.704) | 9528 (7646 - 12453) | 1.476 (1.186 - 1.936) | 0.496 (0.358-0.634) | 2798 (2054 - 3723) | 1.154 (0.85 - 1.543) | 7154 (5727 - 9348) | 1.134 (0.907 - 1.49) | -0.012 (-0.101-0.076) | 92208 (68201 - 122975) | 34.402 (25.266 - 45.69) | 216247 (171762 - 280453) | 32.704 (26.026 - 42.487) | -0.107 (-0.199to-0.016) |
|  | Male | 11807 (9660 - 14875) | 4.318 (3.518 - 5.439) | 41305 (34958 - 47944) | 6.643 (5.635 - 7.675) | 1.443 (1.337-1.55) | 10797 (8799 - 13668) | 4.066 (3.3 - 5.142) | 33931 (28708 - 39300) | 5.6 (4.75 - 6.452) | 1.103 (1.032-1.173) | 349943 (286389 - 442015) | 120.872 (98.561 - 152.754) | 1025884 (866643 - 1193707) | 159.626 (134.921 - 185.26) | 0.952 (0.885-1.019) |
| Nepal | Female | 77 (54 - 107) | 1.535 (1.115 - 2.107) | 201 (140 - 318) | 1.527 (1.065 - 2.41) | -0.049 (-0.223-0.124) | 69 (50 - 96) | 1.445 (1.064 - 2.002) | 157 (108 - 247) | 1.232 (0.842 - 1.927) | -0.523 (-0.68to-0.365) | 2226 (1575 - 3183) | 41.138 (29.661 - 56.78) | 4812 (3317 - 7568) | 35.038 (24.146 - 55.064) | -0.529 (-0.681to-0.376) |
|  | Male | 189 (124 - 287) | 3.552 (2.318 - 5.468) | 572 (399 - 811) | 4.969 (3.472 - 7.062) | 1.31 (1.058-1.562) | 178 (117 - 268) | 3.444 (2.233 - 5.211) | 494 (342 - 718) | 4.387 (3.067 - 6.332) | 1.012 (0.779-1.247) | 5588 (3729 - 8451) | 99.064 (65.412 - 149.347) | 14391 (10002 - 20911) | 121.739 (84.827 - 176.672) | 0.892 (0.65-1.135) |
| Pakistan | Female | 491 (358 - 650) | 1.813 (1.319 - 2.403) | 1420 (929 - 2042) | 2.111 (1.408 - 3.006) | 0.29 (0.156-0.425) | 440 (324 - 586) | 1.677 (1.236 - 2.234) | 1158 (758 - 1678) | 1.819 (1.214 - 2.63) | 0.075 (-0.096-0.245) | 13968 (9867 - 18622) | 48.206 (34.877 - 64.051) | 38959 (25135 - 56224) | 52.635 (34.507 - 76.053) | 0.083 (-0.089-0.254) |
|  | Male | 1160 (865 - 1494) | 3.571 (2.67 - 4.588) | 3283 (2279 - 4562) | 4.603 (3.218 - 6.369) | 0.6 (0.405-0.795) | 1097 (821 - 1411) | 3.425 (2.567 - 4.392) | 2894 (2027 - 3998) | 4.209 (2.976 - 5.825) | 0.451 (0.229-0.673) | 32876 (24491 - 42573) | 98.583 (73.242 - 127.726) | 91966 (63802 - 127817) | 120.955 (84.67 - 167.204) | 0.432 (0.206-0.659) |
| China | Female | 751 (534 - 936) | 0.175 (0.128 - 0.216) | 2261 (1544 - 3169) | 0.207 (0.142 - 0.291) | 0.484 (0.282-0.687) | 622 (449 - 769) | 0.152 (0.113 - 0.188) | 1014 (684 - 1371) | 0.093 (0.063 - 0.126) | -1.884 (-2.02to-1.748) | 17280 (11749 - 21738) | 3.783 (2.651 - 4.728) | 23854 (16292 - 33362) | 2.219 (1.513 - 3.115) | -2.002 (-2.127to-1.876) |
|  | Male | 4323 (3374 - 5485) | 1.013 (0.806 - 1.27) | 9802 (7293 - 12916) | 0.932 (0.702 - 1.221) | -0.678 (-1.198to-0.155) | 3697 (2892 - 4676) | 0.917 (0.733 - 1.14) | 4866 (3650 - 6347) | 0.485 (0.369 - 0.628) | -2.622 (-3.079to-2.163) | 111873 (86832 - 142219) | 24.278 (18.977 - 30.767) | 131816 (98013 - 174031) | 12.427 (9.266 - 16.249) | -2.746 (-3.226to-2.265) |
| Democratic People's Republic of Korea | Female | 17 (11 - 26) | 0.174 (0.114 - 0.267) | 33 (21 - 58) | 0.177 (0.112 - 0.308) | 0.185 (0.113-0.257) | 14 (9 - 22) | 0.15 (0.097 - 0.231) | 25 (16 - 43) | 0.129 (0.082 - 0.223) | -0.417 (-0.47to-0.364) | 394 (256 - 605) | 3.857 (2.502 - 5.851) | 602 (376 - 1052) | 3.257 (2.021 - 5.701) | -0.496 (-0.528to-0.464) |
|  | Male | 50 (35 - 69) | 0.686 (0.485 - 0.936) | 81 (55 - 114) | 0.526 (0.358 - 0.733) | -0.744 (-0.871to-0.617) | 42 (29 - 58) | 0.612 (0.436 - 0.828) | 61 (42 - 87) | 0.419 (0.282 - 0.59) | -1.142 (-1.228to-1.056) | 1363 (945 - 1910) | 17.202 (12.028 - 23.798) | 1895 (1279 - 2712) | 11.799 (8.017 - 16.593) | -1.171 (-1.257to-1.084) |
| Taiwan  (Province of China) | Female | 31 (28 - 34) | 0.392 (0.353 - 0.437) | 87 (73 - 101) | 0.413 (0.35 - 0.48) | 0.281 (0.12-0.443) | 17 (16 - 19) | 0.237 (0.213 - 0.262) | 33 (27 - 38) | 0.146 (0.124 - 0.17) | -1.537 (-1.703to-1.371) | 497 (449 - 552) | 6.139 (5.536 - 6.811) | 810 (691 - 938) | 3.91 (3.336 - 4.527) | -1.394 (-1.55to-1.238) |
|  | Male | 277 (254 - 301) | 3.099 (2.842 - 3.361) | 2014 (1781 - 2250) | 10.478 (9.347 - 11.695) | 4.164 (3.732-4.597) | 177 (163 - 192) | 2.069 (1.906 - 2.242) | 889 (783 - 984) | 4.587 (4.064 - 5.066) | 2.683 (2.354-3.014) | 5506 (5069 - 5962) | 60.088 (55.323 - 65.121) | 26988 (23786 - 29785) | 142.292 (126.22 - 156.56) | 2.855 (2.463-3.248) |
| American Samoa | Female | 0 (0 - 0) | 0.042 (0.024 - 0.057) | 0 (0 - 0) | 0.021 (0.015 - 0.034) | -2.226 (-3.108to-1.337) | 0 (0 - 0) | 0.035 (0.02 - 0.047) | 0 (0 - 0) | 0.016 (0.011 - 0.026) | -2.443 (-3.333to-1.545) | 0 (0 - 0) | 0.927 (0.542 - 1.292) | 0 (0 - 0) | 0.441 (0.307 - 0.726) | -2.299 (-3.205to-1.385) |
|  | Male | 0 (0 - 0) | 0.274 (0.199 - 0.362) | 0 (0 - 0) | 0.568 (0.41 - 0.758) | 3.678 (3.012-4.348) | 0 (0 - 0) | 0.242 (0.176 - 0.318) | 0 (0 - 0) | 0.479 (0.344 - 0.642) | 3.538 (2.87-4.211) | 1 (1 - 1) | 6.913 (5.108 - 9.067) | 4 (3 - 5) | 14.034 (10.047 - 18.756) | 3.645 (2.969-4.326) |
| Cook Islands | Female | 0 (0 - 0) | 0.046 (0.027 - 0.063) | 0 (0 - 0) | 0.027 (0.019 - 0.041) | -2.025 (-2.623to-1.423) | 0 (0 - 0) | 0.036 (0.021 - 0.048) | 0 (0 - 0) | 0.015 (0.01 - 0.021) | -3.152 (-3.743to-2.556) | 0 (0 - 0) | 0.928 (0.55 - 1.303) | 0 (0 - 0) | 0.389 (0.266 - 0.601) | -3.01 (-3.59to-2.426) |
|  | Male | 0 (0 - 0) | 0.085 (0.065 - 0.111) | 0 (0 - 0) | 0.223 (0.158 - 0.298) | 4.18 (3.514-4.85) | 0 (0 - 0) | 0.072 (0.055 - 0.094) | 0 (0 - 0) | 0.146 (0.103 - 0.198) | 3.272 (2.629-3.919) | 0 (0 - 0) | 1.951 (1.512 - 2.523) | 0 (0 - 1) | 4.129 (2.919 - 5.482) | 3.533 (2.834-4.237) |
| Fiji | Female | 0 (0 - 0) | 0.114 (0.082 - 0.205) | 1 (0 - 1) | 0.139 (0.093 - 0.193) | 1.201 (0.877-1.526) | 0 (0 - 0) | 0.098 (0.07 - 0.179) | 0 (0 - 1) | 0.114 (0.076 - 0.156) | 1.069 (0.743-1.396) | 7 (5 - 13) | 2.889 (2.015 - 5.188) | 15 (10 - 21) | 3.32 (2.182 - 4.684) | 1.111 (0.758-1.465) |
|  | Male | 1 (0 - 1) | 0.302 (0.216 - 0.395) | 2 (1 - 2) | 0.426 (0.306 - 0.572) | 1.498 (1.172-1.825) | 1 (0 - 1) | 0.282 (0.201 - 0.369) | 1 (1 - 2) | 0.391 (0.281 - 0.526) | 1.446 (1.119-1.773) | 18 (13 - 23) | 7.869 (5.715 - 10.176) | 44 (31 - 60) | 10.586 (7.566 - 14.203) | 1.391 (1.06-1.724) |
| Guam | Female | 0 (0 - 0) | 0.075 (0.049 - 0.095) | 0 (0 - 0) | 0.023 (0.017 - 0.047) | -2.918 (-3.878to-1.948) | 0 (0 - 0) | 0.054 (0.035 - 0.068) | 0 (0 - 0) | 0.013 (0.009 - 0.025) | -3.638 (-4.539to-2.728) | 1 (0 - 1) | 1.353 (0.903 - 1.738) | 0 (0 - 1) | 0.436 (0.314 - 0.87) | -2.929 (-3.831to-2.018) |
|  | Male | 0 (0 - 0) | 0.327 (0.27 - 0.393) | 1 (0 - 1) | 0.518 (0.429 - 0.612) | 2.978 (2.436-3.524) | 0 (0 - 0) | 0.255 (0.209 - 0.309) | 0 (0 - 0) | 0.364 (0.297 - 0.433) | 2.582 (1.999-3.168) | 4 (3 - 4) | 7.073 (5.952 - 8.345) | 11 (9 - 13) | 11.519 (9.588 - 13.459) | 2.985 (2.417-3.556) |
| Kiribati | Female | 0 (0 - 0) | 0.071 (0.04 - 0.098) | 0 (0 - 0) | 0.071 (0.039 - 0.099) | 0.085 (-0.012-0.181) | 0 (0 - 0) | 0.07 (0.04 - 0.096) | 0 (0 - 0) | 0.068 (0.037 - 0.094) | 0.033 (-0.068-0.133) | 0 (0 - 1) | 1.993 (1.078 - 2.764) | 1 (0 - 1) | 1.876 (0.991 - 2.658) | -0.08 (-0.183-0.023) |
|  | Male | 0 (0 - 0) | 0.715 (0.554 - 0.905) | 0 (0 - 0) | 0.77 (0.54 - 1.081) | 0.228 (0.191-0.266) | 0 (0 - 0) | 0.707 (0.543 - 0.906) | 0 (0 - 0) | 0.745 (0.523 - 1.058) | 0.162 (0.127-0.198) | 4 (3 - 6) | 20.862 (16.498 - 26.056) | 9 (6 - 13) | 22.28 (15.335 - 31.317) | 0.197 (0.152-0.242) |
| Marshall Islands | Female | 0 (0 - 0) | 0.089 (0.056 - 0.13) | 0 (0 - 0) | 0.074 (0.047 - 0.127) | -0.746 (-0.845to-0.648) | 0 (0 - 0) | 0.084 (0.052 - 0.12) | 0 (0 - 0) | 0.066 (0.043 - 0.109) | -0.891 (-1.023to-0.758) | 0 (0 - 0) | 2.353 (1.446 - 3.548) | 0 (0 - 1) | 1.878 (1.13 - 3.314) | -0.866 (-0.997to-0.735) |
|  | Male | 0 (0 - 0) | 0.259 (0.168 - 0.412) | 0 (0 - 0) | 0.269 (0.172 - 0.413) | 0.236 (0.129-0.343) | 0 (0 - 0) | 0.249 (0.162 - 0.395) | 0 (0 - 0) | 0.25 (0.161 - 0.382) | 0.124 (0.031-0.217) | 1 (0 - 1) | 7.197 (4.623 - 11.451) | 2 (1 - 3) | 7.441 (4.727 - 11.477) | 0.194 (0.123-0.265) |
| Micronesia  (Federated States of) | Female | 0 (0 - 0) | 0.104 (0.065 - 0.151) | 0 (0 - 0) | 0.085 (0.055 - 0.126) | -0.69 (-0.73to-0.649) | 0 (0 - 0) | 0.097 (0.061 - 0.14) | 0 (0 - 0) | 0.074 (0.048 - 0.108) | -0.965 (-1.004to-0.927) | 1 (0 - 1) | 2.778 (1.708 - 4.143) | 1 (1 - 1) | 2.08 (1.336 - 3.134) | -1 (-1.039to-0.961) |
|  | Male | 0 (0 - 0) | 0.277 (0.206 - 0.396) | 0 (0 - 0) | 0.271 (0.191 - 0.388) | -0.072 (-0.177-0.033) | 0 (0 - 0) | 0.267 (0.198 - 0.377) | 0 (0 - 0) | 0.248 (0.173 - 0.357) | -0.258 (-0.35to-0.165) | 2 (2 - 3) | 7.849 (5.815 - 11.103) | 3 (2 - 5) | 7.276 (5.205 - 10.552) | -0.26 (-0.349to-0.171) |
| Nauru | Female | 0 (0 - 0) | 0.117 (0.071 - 0.178) | 0 (0 - 0) | 0.093 (0.059 - 0.154) | -0.812 (-0.879to-0.744) | 0 (0 - 0) | 0.105 (0.065 - 0.157) | 0 (0 - 0) | 0.078 (0.05 - 0.127) | -0.973 (-1.11to-0.836) | 0 (0 - 0) | 3.05 (1.808 - 4.768) | 0 (0 - 0) | 2.279 (1.376 - 3.914) | -0.981 (-1.123to-0.84) |
|  | Male | 0 (0 - 0) | 0.402 (0.209 - 0.612) | 0 (0 - 0) | 0.397 (0.225 - 0.581) | -0.05 (-0.174-0.074) | 0 (0 - 0) | 0.378 (0.201 - 0.567) | 0 (0 - 0) | 0.359 (0.208 - 0.529) | -0.152 (-0.227to-0.077) | 0 (0 - 1) | 11.139 (5.573 - 17.406) | 0 (0 - 1) | 10.588 (5.919 - 15.576) | -0.16 (-0.23to-0.09) |
| Niue | Female | 0 (0 - 0) | 0.097 (0.066 - 0.132) | 0 (0 - 0) | 0.084 (0.057 - 0.113) | -0.762 (-0.847to-0.676) | 0 (0 - 0) | 0.081 (0.055 - 0.111) | 0 (0 - 0) | 0.061 (0.042 - 0.081) | -1.172 (-1.253to-1.091) | 0 (0 - 0) | 2.244 (1.494 - 3.141) | 0 (0 - 0) | 1.745 (1.194 - 2.329) | -1.198 (-1.312to-1.084) |
|  | Male | 0 (0 - 0) | 0.278 (0.203 - 0.37) | 0 (0 - 0) | 0.304 (0.22 - 0.42) | 0.232 (0.17-0.294) | 0 (0 - 0) | 0.251 (0.184 - 0.332) | 0 (0 - 0) | 0.253 (0.18 - 0.345) | -0.03 (-0.083-0.023) | 0 (0 - 0) | 7.054 (5.044 - 9.632) | 0 (0 - 0) | 6.967 (4.92 - 9.846) | -0.124 (-0.176to-0.072) |
| Northern Mariana Islands | Female | 0 (0 - 0) | 0.437 (0.289 - 0.664) | 0 (0 - 0) | 0.296 (0.214 - 0.552) | -1.117 (-2.66-0.451) | 0 (0 - 0) | 0.312 (0.215 - 0.462) | 0 (0 - 0) | 0.193 (0.14 - 0.356) | -1.223 (-2.751-0.329) | 1 (1 - 2) | 8.404 (5.641 - 12.502) | 1 (1 - 3) | 5.155 (3.782 - 9.908) | -1.223 (-2.728-0.305) |
|  | Male | 0 (0 - 0) | 1.636 (1.232 - 2.176) | 0 (0 - 0) | 1.338 (1.058 - 1.679) | -0.793 (-0.901to-0.685) | 0 (0 - 0) | 1.307 (1.005 - 1.705) | 0 (0 - 0) | 1.013 (0.797 - 1.265) | -0.844 (-0.969to-0.718) | 6 (4 - 8) | 37.183 (27.503 - 48.808) | 8 (6 - 11) | 27.94 (21.968 - 34.843) | -0.968 (-1.086to-0.85) |
| Palau | Female | 0 (0 - 0) | 0.083 (0.057 - 0.12) | 0 (0 - 0) | 0.074 (0.05 - 0.108) | -0.301 (-0.344to-0.259) | 0 (0 - 0) | 0.07 (0.049 - 0.099) | 0 (0 - 0) | 0.058 (0.04 - 0.083) | -0.472 (-0.54to-0.404) | 0 (0 - 0) | 1.817 (1.229 - 2.655) | 0 (0 - 0) | 1.446 (0.966 - 2.101) | -0.665 (-0.724to-0.607) |
|  | Male | 0 (0 - 0) | 0 (0 - 0) | 0 (0 - 0) | 0 (0 - 0) | -0.248 (-0.306to-0.189) | 0 (0 - 0) | 0 (0 - 0) | 0 (0 - 0) | 0 (0 - 0) | -0.511 (-0.569to-0.453) | 0 (0 - 0) | 0.002 (0.001 - 0.003) | 0 (0 - 0) | 0.002 (0.001 - 0.002) | -0.373 (-0.435to-0.31) |
| Papua New Guinea | Female | 1 (0 - 1) | 0.085 (0.051 - 0.124) | 2 (1 - 4) | 0.079 (0.05 - 0.122) | -0.406 (-0.504to-0.307) | 1 (0 - 1) | 0.078 (0.047 - 0.111) | 2 (1 - 3) | 0.071 (0.045 - 0.107) | -0.424 (-0.503to-0.345) | 25 (14 - 37) | 2.262 (1.281 - 3.317) | 68 (41 - 108) | 2.04 (1.253 - 3.188) | -0.464 (-0.561to-0.367) |
|  | Male | 2 (1 - 3) | 0.156 (0.096 - 0.262) | 5 (3 - 7) | 0.153 (0.097 - 0.23) | -0.053 (-0.229-0.123) | 2 (1 - 3) | 0.15 (0.094 - 0.253) | 4 (3 - 6) | 0.146 (0.092 - 0.22) | -0.066 (-0.227-0.095) | 50 (30 - 87) | 4.283 (2.639 - 7.328) | 144 (90 - 217) | 4.143 (2.606 - 6.25) | -0.085 (-0.255-0.086) |
| Samoa | Female | 0 (0 - 0) | 0.096 (0.062 - 0.136) | 0 (0 - 0) | 0.091 (0.061 - 0.127) | -0.261 (-0.317to-0.205) | 0 (0 - 0) | 0.084 (0.054 - 0.117) | 0 (0 - 0) | 0.071 (0.049 - 0.1) | -0.575 (-0.621to-0.529) | 1 (1 - 2) | 2.321 (1.453 - 3.332) | 2 (1 - 2) | 2 (1.344 - 2.781) | -0.505 (-0.55to-0.46) |
|  | Male | 0 (0 - 0) | 0.374 (0.291 - 0.483) | 0 (0 - 0) | 0.365 (0.251 - 0.51) | -0.083 (-0.162to-0.004) | 0 (0 - 0) | 0.344 (0.27 - 0.441) | 0 (0 - 0) | 0.313 (0.216 - 0.438) | -0.293 (-0.366to-0.219) | 4 (3 - 6) | 9.519 (7.242 - 12.53) | 7 (5 - 10) | 8.773 (6.033 - 12.759) | -0.247 (-0.341to-0.153) |
| Solomon Islands | Female | 0 (0 - 0) | 0.084 (0.043 - 0.124) | 0 (0 - 0) | 0.081 (0.052 - 0.122) | -0.209 (-0.323to-0.095) | 0 (0 - 0) | 0.08 (0.042 - 0.117) | 0 (0 - 0) | 0.073 (0.048 - 0.108) | -0.379 (-0.463to-0.294) | 2 (1 - 3) | 2.21 (1.077 - 3.411) | 5 (3 - 7) | 2.082 (1.312 - 3.249) | -0.237 (-0.347to-0.128) |
|  | Male | 0 (0 - 0) | 0.207 (0.133 - 0.312) | 0 (0 - 1) | 0.223 (0.15 - 0.333) | 0.261 (0.089-0.433) | 0 (0 - 0) | 0.201 (0.128 - 0.299) | 0 (0 - 1) | 0.209 (0.14 - 0.31) | 0.151 (-0.008-0.31) | 5 (4 - 8) | 5.902 (3.805 - 8.968) | 14 (10 - 22) | 6.289 (4.295 - 9.534) | 0.232 (0.079-0.386) |
| Tokelau | Female | 0 (0 - 0) | 0.103 (0.064 - 0.146) | 0 (0 - 0) | 0.086 (0.058 - 0.119) | -0.814 (-0.91to-0.718) | 0 (0 - 0) | 0.092 (0.058 - 0.13) | 0 (0 - 0) | 0.064 (0.043 - 0.087) | -1.35 (-1.446to-1.254) | 0 (0 - 0) | 2.582 (1.621 - 3.749) | 0 (0 - 0) | 1.874 (1.263 - 2.633) | -1.324 (-1.441to-1.207) |
|  | Male | 0 (0 - 0) | 0.232 (0.157 - 0.349) | 0 (0 - 0) | 0.232 (0.155 - 0.376) | 0.071 (-0.034-0.176) | 0 (0 - 0) | 0.22 (0.149 - 0.328) | 0 (0 - 0) | 0.196 (0.13 - 0.318) | -0.28 (-0.363to-0.197) | 0 (0 - 0) | 6.096 (4.089 - 9.399) | 0 (0 - 0) | 5.552 (3.67 - 9.141) | -0.258 (-0.346to-0.17) |
| Tonga | Female | 0 (0 - 0) | 0.085 (0.058 - 0.118) | 0 (0 - 0) | 0.071 (0.047 - 0.104) | -0.618 (-0.664to-0.572) | 0 (0 - 0) | 0.071 (0.048 - 0.099) | 0 (0 - 0) | 0.055 (0.036 - 0.081) | -0.786 (-0.849to-0.723) | 1 (0 - 1) | 1.952 (1.328 - 2.755) | 1 (0 - 1) | 1.48 (0.966 - 2.185) | -0.927 (-0.987to-0.866) |
|  | Male | 0 (0 - 0) | 0.188 (0.123 - 0.293) | 0 (0 - 0) | 0.22 (0.141 - 0.365) | 0.521 (0.423-0.619) | 0 (0 - 0) | 0.17 (0.111 - 0.266) | 0 (0 - 0) | 0.188 (0.119 - 0.31) | 0.337 (0.216-0.458) | 1 (1 - 2) | 4.62 (3.087 - 7.217) | 2 (1 - 3) | 5.204 (3.328 - 8.461) | 0.422 (0.327-0.517) |
| Tuvalu | Female | 0 (0 - 0) | 0.099 (0.06 - 0.151) | 0 (0 - 0) | 0.083 (0.055 - 0.118) | -0.739 (-0.831to-0.646) | 0 (0 - 0) | 0.093 (0.057 - 0.139) | 0 (0 - 0) | 0.07 (0.047 - 0.096) | -1.052 (-1.14to-0.965) | 0 (0 - 0) | 2.657 (1.569 - 4.145) | 0 (0 - 0) | 1.95 (1.265 - 2.857) | -1.116 (-1.2to-1.032) |
|  | Male | 0 (0 - 0) | 0.234 (0.168 - 0.341) | 0 (0 - 0) | 0.242 (0.166 - 0.351) | 0.135 (0.08-0.191) | 0 (0 - 0) | 0.227 (0.164 - 0.329) | 0 (0 - 0) | 0.219 (0.148 - 0.323) | -0.085 (-0.138to-0.031) | 0 (0 - 0) | 6.53 (4.656 - 9.474) | 0 (0 - 1) | 6.331 (4.3 - 9.255) | -0.072 (-0.129to-0.015) |
| Vanuatu | Female | 0 (0 - 0) | 0.079 (0.047 - 0.117) | 0 (0 - 0) | 0.071 (0.046 - 0.099) | -0.633 (-0.746to-0.519) | 0 (0 - 0) | 0.074 (0.045 - 0.107) | 0 (0 - 0) | 0.064 (0.042 - 0.088) | -0.686 (-0.797to-0.574) | 1 (0 - 1) | 2.027 (1.145 - 3.014) | 2 (1 - 3) | 1.799 (1.132 - 2.566) | -0.685 (-0.819to-0.552) |
|  | Male | 0 (0 - 0) | 0.201 (0.138 - 0.303) | 0 (0 - 0) | 0.207 (0.153 - 0.296) | 0.067 (-0.058-0.192) | 0 (0 - 0) | 0.192 (0.132 - 0.287) | 0 (0 - 0) | 0.195 (0.145 - 0.273) | 0.019 (-0.092-0.13) | 2 (2 - 4) | 5.563 (3.733 - 8.69) | 6 (4 - 9) | 5.729 (4.181 - 8.19) | 0.062 (-0.059-0.183) |
| Cambodia | Female | 10 (6 - 17) | 0.366 (0.23 - 0.617) | 26 (17 - 45) | 0.359 (0.238 - 0.618) | -0.113 (-0.193to-0.034) | 9 (6 - 16) | 0.358 (0.227 - 0.597) | 22 (14 - 37) | 0.311 (0.207 - 0.52) | -0.515 (-0.565to-0.465) | 272 (170 - 490) | 9.373 (5.922 - 16.527) | 597 (392 - 1018) | 7.802 (5.167 - 13.28) | -0.67 (-0.722to-0.617) |
|  | Male | 18 (13 - 25) | 0.883 (0.624 - 1.241) | 57 (40 - 82) | 1.048 (0.734 - 1.487) | 0.628 (0.376-0.88) | 17 (12 - 24) | 0.881 (0.619 - 1.226) | 50 (35 - 72) | 0.974 (0.684 - 1.388) | 0.389 (0.16-0.618) | 519 (373 - 732) | 23.841 (16.974 - 33.616) | 1467 (1003 - 2144) | 24.956 (17.32 - 36.032) | 0.18 (-0.042-0.402) |
| Indonesia | Female | 165 (110 - 270) | 0.309 (0.204 - 0.523) | 380 (255 - 682) | 0.303 (0.207 - 0.533) | -0.257 (-0.323to-0.19) | 145 (96 - 240) | 0.286 (0.187 - 0.488) | 298 (200 - 529) | 0.255 (0.175 - 0.44) | -0.524 (-0.612to-0.435) | 4438 (2978 - 7014) | 7.596 (5.046 - 12.447) | 8486 (5647 - 15213) | 6.331 (4.261 - 11.203) | -0.754 (-0.839to-0.67) |
|  | Male | 321 (231 - 418) | 0.634 (0.454 - 0.823) | 889 (567 - 1252) | 0.723 (0.466 - 0.994) | 0.365 (0.26-0.47) | 296 (217 - 386) | 0.609 (0.445 - 0.785) | 762 (488 - 1071) | 0.656 (0.424 - 0.893) | 0.191 (0.118-0.265) | 9161 (6733 - 11919) | 16.687 (12.189 - 21.765) | 22210 (14084 - 31700) | 16.728 (10.705 - 23.477) | -0.052 (-0.131-0.028) |
| Lao People's Democratic Republic | Female | 4 (2 - 8) | 0.35 (0.201 - 0.666) | 7 (5 - 13) | 0.299 (0.191 - 0.52) | -0.593 (-0.628to-0.558) | 4 (2 - 7) | 0.349 (0.202 - 0.64) | 6 (4 - 11) | 0.272 (0.175 - 0.474) | -0.884 (-0.924to-0.845) | 110 (62 - 214) | 9.16 (5.237 - 17.514) | 185 (115 - 333) | 6.894 (4.338 - 12.266) | -1.022 (-1.064to-0.981) |
|  | Male | 9 (6 - 14) | 0.882 (0.594 - 1.344) | 17 (12 - 24) | 0.725 (0.513 - 1) | -0.659 (-0.887to-0.429) | 9 (6 - 14) | 0.884 (0.598 - 1.346) | 15 (11 - 22) | 0.693 (0.492 - 0.952) | -0.815 (-1.02to-0.61) | 273 (177 - 423) | 24.579 (16.266 - 37.805) | 459 (310 - 650) | 18.043 (12.506 - 25.285) | -1.042 (-1.249to-0.835) |
| Malaysia | Female | 27 (16 - 35) | 0.538 (0.322 - 0.696) | 81 (54 - 102) | 0.55 (0.369 - 0.701) | -0.15 (-0.268to-0.033) | 22 (13 - 28) | 0.452 (0.276 - 0.585) | 51 (35 - 65) | 0.364 (0.248 - 0.464) | -0.928 (-1.032to-0.824) | 628 (367 - 811) | 11.927 (7.038 - 15.562) | 1402 (934 - 1778) | 9.336 (6.223 - 11.79) | -1.058 (-1.194to-0.921) |
|  | Male | 70 (55 - 87) | 1.512 (1.196 - 1.885) | 249 (196 - 314) | 1.675 (1.332 - 2.101) | -0.069 (-0.348-0.21) | 62 (49 - 76) | 1.361 (1.083 - 1.701) | 184 (146 - 231) | 1.272 (1.02 - 1.58) | -0.684 (-0.941to-0.427) | 1807 (1418 - 2250) | 36.715 (29.089 - 45.914) | 5194 (4053 - 6560) | 33.953 (26.638 - 42.699) | -0.659 (-0.96to-0.357) |
| Maldives | Female | 0 (0 - 0) | 0.254 (0.143 - 0.478) | 0 (0 - 0) | 0.208 (0.14 - 0.298) | -0.822 (-0.925to-0.72) | 0 (0 - 0) | 0.236 (0.13 - 0.426) | 0 (0 - 0) | 0.129 (0.087 - 0.178) | -2.267 (-2.397to-2.137) | 2 (1 - 5) | 5.768 (3.204 - 11.505) | 5 (3 - 7) | 3.075 (2.088 - 4.416) | -2.311 (-2.428to-2.193) |
|  | Male | 1 (0 - 1) | 1.114 (0.744 - 1.564) | 2 (1 - 3) | 1.004 (0.742 - 1.346) | -0.55 (-0.666to-0.434) | 1 (0 - 1) | 1.083 (0.733 - 1.496) | 1 (1 - 2) | 0.763 (0.567 - 1) | -1.432 (-1.564to-1.3) | 16 (10 - 23) | 27.299 (17.484 - 38.9) | 39 (28 - 53) | 17.741 (12.946 - 24.285) | -1.727 (-1.869to-1.585) |
| Mauritius | Female | 0 (0 - 0) | 0.094 (0.087 - 0.101) | 2 (2 - 2) | 0.213 (0.192 - 0.232) | 0.448 (-1.461-2.393) | 0 (0 - 0) | 0.075 (0.069 - 0.081) | 1 (1 - 2) | 0.14 (0.127 - 0.153) | -0.03 (-1.889-1.864) | 8 (7 - 8) | 1.867 (1.736 - 2.003) | 35 (31 - 38) | 3.554 (3.184 - 3.836) | -0.03 (-1.882-1.856) |
|  | Male | 2 (2 - 3) | 0.675 (0.627 - 0.725) | 13 (12 - 14) | 1.513 (1.377 - 1.63) | 1.399 (0.157-2.656) | 2 (2 - 2) | 0.581 (0.54 - 0.623) | 10 (9 - 11) | 1.139 (1.044 - 1.23) | 1.015 (-0.198-2.242) | 57 (53 - 61) | 15.471 (14.378 - 16.621) | 276 (250 - 299) | 30.691 (27.894 - 33.242) | 1.076 (-0.123-2.289) |
| Myanmar | Female | 49 (30 - 93) | 0.391 (0.243 - 0.71) | 85 (55 - 158) | 0.304 (0.199 - 0.561) | -1.067 (-1.161to-0.972) | 46 (29 - 86) | 0.379 (0.241 - 0.676) | 71 (46 - 129) | 0.263 (0.17 - 0.472) | -1.423 (-1.521to-1.324) | 1383 (847 - 2622) | 10.137 (6.298 - 19.006) | 1919 (1210 - 3549) | 6.653 (4.25 - 12.204) | -1.635 (-1.741to-1.529) |
|  | Male | 80 (54 - 117) | 0.699 (0.493 - 1.004) | 145 (100 - 198) | 0.66 (0.461 - 0.901) | -0.154 (-0.353-0.045) | 77 (53 - 112) | 0.693 (0.496 - 0.996) | 129 (89 - 177) | 0.611 (0.428 - 0.835) | -0.376 (-0.541to-0.21) | 2301 (1554 - 3412) | 18.954 (12.973 - 27.708) | 3683 (2495 - 5032) | 15.785 (10.823 - 21.552) | -0.577 (-0.74to-0.413) |
| Philippines | Female | 52 (42 - 73) | 0.35 (0.274 - 0.481) | 130 (98 - 222) | 0.292 (0.223 - 0.496) | -0.594 (-0.634to-0.554) | 44 (36 - 62) | 0.326 (0.252 - 0.444) | 104 (79 - 179) | 0.246 (0.188 - 0.416) | -0.766 (-0.834to-0.697) | 1309 (1059 - 1793) | 7.68 (6.174 - 10.706) | 2861 (2113 - 4961) | 6.105 (4.551 - 10.541) | -0.746 (-0.797to-0.695) |
|  | Male | 90 (71 - 122) | 0.578 (0.454 - 0.787) | 274 (214 - 340) | 0.677 (0.535 - 0.831) | 0.412 (0.335-0.489) | 80 (63 - 107) | 0.532 (0.42 - 0.723) | 235 (184 - 292) | 0.607 (0.481 - 0.746) | 0.393 (0.34-0.446) | 2536 (2033 - 3359) | 14.894 (11.821 - 20.083) | 7033 (5466 - 8745) | 16.163 (12.672 - 20.068) | 0.146 (0.075-0.216) |
| Seychelles | Female | 0 (0 - 0) | 0.473 (0.363 - 0.767) | 0 (0 - 0) | 0.577 (0.43 - 0.759) | 0.018 (-0.515-0.554) | 0 (0 - 0) | 0.394 (0.301 - 0.643) | 0 (0 - 0) | 0.409 (0.298 - 0.535) | -0.49 (-1.026-0.05) | 3 (2 - 5) | 10.352 (8.06 - 16.585) | 6 (5 - 8) | 10.217 (7.66 - 13.536) | -0.635 (-1.135to-0.133) |
|  | Male | 1 (1 - 2) | 5.664 (4.558 - 6.887) | 5 (4 - 6) | 7.519 (6.029 - 9.126) | 0.858 (0.421-1.296) | 1 (1 - 2) | 5.028 (4.071 - 6.148) | 3 (3 - 4) | 6.014 (4.833 - 7.271) | 0.543 (0.111-0.976) | 36 (28 - 44) | 139.592 (110.583 - 171.329) | 100 (80 - 122) | 156.95 (124.79 - 189.91) | 0.257 (-0.177-0.694) |
| Sri Lanka | Female | 48 (30 - 62) | 0.89 (0.541 - 1.132) | 96 (55 - 150) | 0.633 (0.364 - 0.988) | -1.718 (-2.051to-1.384) | 39 (24 - 49) | 0.755 (0.454 - 0.946) | 56 (32 - 85) | 0.373 (0.217 - 0.568) | -2.939 (-3.27to-2.607) | 1097 (689 - 1400) | 18.77 (11.723 - 23.914) | 1404 (783 - 2156) | 9.324 (5.237 - 14.444) | -2.906 (-3.228to-2.582) |
|  | Male | 154 (119 - 201) | 2.794 (2.179 - 3.616) | 325 (187 - 490) | 2.589 (1.505 - 3.875) | -0.382 (-0.678to-0.085) | 135 (105 - 173) | 2.555 (1.994 - 3.26) | 219 (128 - 327) | 1.797 (1.062 - 2.649) | -1.336 (-1.66to-1.012) | 3832 (2893 - 4997) | 65.165 (49.591 - 85.137) | 6050 (3492 - 9137) | 47.67 (27.806 - 71.491) | -1.194 (-1.516to-0.871) |
| Thailand | Female | 76 (55 - 111) | 0.377 (0.271 - 0.561) | 168 (109 - 377) | 0.29 (0.189 - 0.645) | -1.423 (-1.62to-1.224) | 57 (41 - 84) | 0.296 (0.215 - 0.441) | 97 (63 - 209) | 0.163 (0.107 - 0.352) | -2.492 (-2.703to-2.282) | 1702 (1250 - 2465) | 7.969 (5.812 - 11.598) | 2353 (1528 - 5129) | 4.155 (2.717 - 8.946) | -2.733 (-2.981to-2.484) |
|  | Male | 256 (180 - 353) | 1.403 (0.989 - 1.923) | 844 (611 - 1172) | 1.668 (1.224 - 2.274) | 0.023 (-0.212-0.258) | 209 (147 - 286) | 1.204 (0.856 - 1.63) | 539 (393 - 741) | 1.074 (0.792 - 1.449) | -0.965 (-1.202to-0.728) | 6574 (4635 - 9101) | 33.686 (23.827 - 46.082) | 15757 (11413 - 21629) | 31.319 (22.929 - 42.435) | -0.845 (-1.103to-0.586) |
| Timor-Leste | Female | 0 (0 - 1) | 0.311 (0.2 - 0.532) | 1 (1 - 2) | 0.3 (0.203 - 0.514) | -0.093 (-0.25-0.065) | 0 (0 - 1) | 0.303 (0.198 - 0.506) | 1 (1 - 2) | 0.27 (0.182 - 0.458) | -0.392 (-0.526to-0.258) | 12 (8 - 21) | 7.679 (4.835 - 13.154) | 31 (20 - 55) | 6.736 (4.436 - 11.891) | -0.456 (-0.637to-0.275) |
|  | Male | 1 (0 - 1) | 0.487 (0.291 - 0.743) | 2 (1 - 3) | 0.506 (0.324 - 0.78) | 0.24 (-0.062-0.543) | 1 (0 - 1) | 0.485 (0.287 - 0.735) | 2 (1 - 3) | 0.481 (0.313 - 0.735) | 0.055 (-0.23-0.341) | 23 (13 - 36) | 13.014 (7.58 - 20.013) | 56 (35 - 88) | 12.482 (7.875 - 19.423) | -0.083 (-0.398-0.232) |
| Viet Nam | Female | 164 (113 - 224) | 0.698 (0.482 - 0.95) | 497 (314 - 697) | 0.861 (0.547 - 1.191) | 0.779 (0.712-0.845) | 138 (94 - 188) | 0.59 (0.405 - 0.806) | 298 (194 - 413) | 0.528 (0.346 - 0.723) | -0.257 (-0.31to-0.205) | 3872 (2575 - 5392) | 16.216 (10.922 - 22.533) | 8332 (5161 - 11672) | 14.264 (8.883 - 19.915) | -0.304 (-0.362to-0.245) |
|  | Male | 623 (438 - 851) | 3.464 (2.465 - 4.714) | 2609 (1893 - 3447) | 5.337 (3.915 - 6.945) | 1.471 (1.346-1.597) | 546 (387 - 749) | 3.1 (2.229 - 4.196) | 1792 (1309 - 2354) | 3.867 (2.883 - 4.995) | 0.775 (0.695-0.854) | 16508 (11736 - 22591) | 89.383 (64.039 - 121.872) | 54802 (39368 - 73159) | 107.116 (78.031 - 140.598) | 0.645 (0.545-0.746) |
| Angola | Female | 4 (2 - 5) | 0.168 (0.11 - 0.235) | 14 (9 - 20) | 0.2 (0.124 - 0.282) | 0.582 (0.513-0.65) | 3 (2 - 5) | 0.165 (0.109 - 0.225) | 12 (7 - 17) | 0.182 (0.114 - 0.253) | 0.347 (0.303-0.391) | 107 (66 - 154) | 4.534 (2.899 - 6.434) | 391 (234 - 558) | 4.895 (2.957 - 6.951) | 0.289 (0.231-0.347) |
|  | Male | 12 (7 - 17) | 0.524 (0.346 - 0.742) | 40 (28 - 57) | 0.647 (0.454 - 0.905) | 0.997 (0.794-1.201) | 11 (7 - 16) | 0.517 (0.343 - 0.731) | 35 (25 - 51) | 0.609 (0.424 - 0.866) | 0.848 (0.664-1.033) | 358 (225 - 519) | 14.803 (9.615 - 21.281) | 1148 (816 - 1669) | 17.036 (12.016 - 24.643) | 0.783 (0.591-0.976) |
| Central African Republic | Female | 1 (1 - 2) | 0.187 (0.115 - 0.29) | 2 (1 - 4) | 0.189 (0.113 - 0.298) | 0.016 (-0.025-0.057) | 1 (1 - 2) | 0.186 (0.116 - 0.283) | 2 (1 - 4) | 0.188 (0.112 - 0.298) | 0.019 (-0.013-0.051) | 37 (22 - 59) | 5.073 (3.083 - 7.973) | 74 (43 - 119) | 5.036 (2.971 - 8.124) | -0.057 (-0.101to-0.013) |
|  | Male | 3 (2 - 5) | 0.556 (0.352 - 0.773) | 6 (4 - 9) | 0.486 (0.345 - 0.716) | -0.43 (-0.469to-0.392) | 3 (2 - 5) | 0.551 (0.354 - 0.766) | 6 (4 - 9) | 0.48 (0.345 - 0.696) | -0.432 (-0.47to-0.395) | 105 (61 - 151) | 16.074 (9.766 - 22.762) | 191 (127 - 295) | 13.886 (9.72 - 20.841) | -0.487 (-0.524to-0.45) |
| Congo | Female | 1 (1 - 2) | 0.2 (0.131 - 0.295) | 3 (2 - 5) | 0.216 (0.136 - 0.305) | 0.238 (0.173-0.304) | 1 (1 - 2) | 0.193 (0.13 - 0.276) | 3 (2 - 4) | 0.191 (0.122 - 0.267) | -0.042 (-0.102-0.017) | 35 (22 - 54) | 5.239 (3.358 - 8.03) | 88 (53 - 131) | 5.164 (3.209 - 7.558) | -0.063 (-0.139-0.013) |
|  | Male | 4 (2 - 5) | 0.71 (0.437 - 1.016) | 10 (7 - 14) | 0.636 (0.428 - 0.93) | -0.438 (-0.662to-0.214) | 3 (2 - 5) | 0.691 (0.428 - 0.981) | 9 (6 - 12) | 0.588 (0.397 - 0.863) | -0.599 (-0.794to-0.403) | 110 (64 - 161) | 19.937 (11.868 - 28.873) | 279 (184 - 406) | 16.425 (10.92 - 23.92) | -0.727 (-0.936to-0.519) |
| Democratic Republic of the Congo | Female | 16 (10 - 23) | 0.174 (0.112 - 0.252) | 43 (26 - 62) | 0.203 (0.125 - 0.292) | 0.63 (0.515-0.745) | 14 (9 - 21) | 0.168 (0.11 - 0.24) | 38 (23 - 54) | 0.188 (0.117 - 0.268) | 0.5 (0.414-0.585) | 449 (292 - 667) | 4.551 (2.963 - 6.631) | 1155 (692 - 1665) | 4.995 (2.997 - 7.203) | 0.436 (0.343-0.529) |
|  | Male | 33 (23 - 45) | 0.411 (0.296 - 0.566) | 77 (48 - 126) | 0.396 (0.243 - 0.657) | -0.095 (-0.415-0.226) | 31 (22 - 43) | 0.404 (0.292 - 0.552) | 70 (43 - 115) | 0.377 (0.229 - 0.629) | -0.177 (-0.47-0.116) | 963 (691 - 1323) | 11.403 (8.247 - 15.663) | 2266 (1390 - 3732) | 10.519 (6.528 - 17.475) | -0.21 (-0.506-0.086) |
| Equatorial Guinea | Female | 0 (0 - 0) | 0.174 (0.112 - 0.259) | 1 (0 - 1) | 0.204 (0.103 - 0.336) | 0.643 (0.561-0.724) | 0 (0 - 0) | 0.171 (0.112 - 0.251) | 0 (0 - 1) | 0.164 (0.087 - 0.274) | -0.075 (-0.15-0.001) | 6 (4 - 9) | 4.668 (2.989 - 7.185) | 16 (7 - 27) | 4.383 (2.147 - 7.444) | -0.178 (-0.254to-0.102) |
|  | Male | 1 (0 - 1) | 0.542 (0.349 - 0.745) | 2 (1 - 3) | 0.782 (0.451 - 1.203) | 1.466 (1.325-1.608) | 1 (0 - 1) | 0.535 (0.35 - 0.726) | 2 (1 - 3) | 0.675 (0.394 - 1.038) | 0.987 (0.877-1.097) | 16 (10 - 23) | 15.511 (9.682 - 21.797) | 52 (29 - 84) | 18.759 (10.521 - 29.691) | 0.819 (0.688-0.95) |
| Gabon | Female | 1 (0 - 1) | 0.192 (0.126 - 0.278) | 1 (1 - 2) | 0.206 (0.12 - 0.305) | 0.116 (0.052-0.18) | 1 (0 - 1) | 0.181 (0.121 - 0.263) | 1 (1 - 1) | 0.173 (0.101 - 0.258) | -0.217 (-0.312to-0.121) | 16 (10 - 23) | 4.84 (3.185 - 7.14) | 28 (16 - 44) | 4.532 (2.556 - 6.883) | -0.305 (-0.408to-0.203) |
|  | Male | 2 (1 - 3) | 0.879 (0.483 - 1.26) | 5 (3 - 7) | 0.885 (0.602 - 1.191) | -0.06 (-0.136-0.016) | 2 (1 - 3) | 0.843 (0.468 - 1.207) | 4 (3 - 6) | 0.783 (0.541 - 1.049) | -0.3 (-0.353to-0.248) | 67 (35 - 98) | 23.865 (12.624 - 34.713) | 132 (86 - 185) | 21.791 (14.553 - 29.925) | -0.378 (-0.437to-0.32) |
| Burundi | Female | 6 (2 - 9) | 0.418 (0.168 - 0.627) | 11 (5 - 17) | 0.416 (0.174 - 0.609) | -0.089 (-0.131to-0.046) | 5 (2 - 8) | 0.398 (0.161 - 0.591) | 10 (4 - 15) | 0.381 (0.165 - 0.566) | -0.206 (-0.24to-0.173) | 173 (66 - 266) | 11.86 (4.59 - 18.125) | 340 (141 - 505) | 11.162 (4.713 - 16.619) | -0.278 (-0.318to-0.239) |
|  | Male | 17 (12 - 26) | 1.518 (1.063 - 2.284) | 27 (17 - 44) | 0.897 (0.583 - 1.47) | -2.119 (-2.392to-1.845) | 16 (11 - 25) | 1.473 (1.043 - 2.225) | 25 (16 - 41) | 0.858 (0.553 - 1.417) | -2.167 (-2.431to-1.904) | 521 (361 - 801) | 44.416 (30.687 - 68.131) | 803 (495 - 1344) | 25.057 (15.774 - 41.729) | -2.287 (-2.561to-2.012) |
| Comoros | Female | 1 (0 - 1) | 0.473 (0.201 - 0.688) | 1 (1 - 2) | 0.508 (0.24 - 0.732) | 0.132 (0.056-0.207) | 0 (0 - 1) | 0.434 (0.188 - 0.628) | 1 (1 - 2) | 0.44 (0.211 - 0.629) | -0.057 (-0.131-0.018) | 16 (6 - 24) | 13.126 (5.383 - 19.289) | 39 (18 - 57) | 13.008 (6.131 - 18.874) | -0.181 (-0.297to-0.066) |
|  | Male | 1 (1 - 2) | 1.085 (0.688 - 1.595) | 2 (1 - 4) | 0.986 (0.586 - 1.726) | -0.543 (-0.715to-0.371) | 1 (1 - 2) | 1.04 (0.651 - 1.523) | 2 (1 - 4) | 0.913 (0.539 - 1.617) | -0.651 (-0.815to-0.487) | 35 (22 - 53) | 30.754 (19.511 - 46.164) | 71 (41 - 126) | 26.349 (15.379 - 47.286) | -0.764 (-0.956to-0.572) |
| Djibouti | Female | 0 (0 - 1) | 0.451 (0.203 - 0.698) | 2 (1 - 3) | 0.504 (0.219 - 0.822) | 0.376 (0.331-0.421) | 0 (0 - 0) | 0.411 (0.191 - 0.632) | 1 (1 - 2) | 0.433 (0.191 - 0.71) | 0.21 (0.124-0.296) | 11 (5 - 17) | 12.094 (5.412 - 18.978) | 49 (21 - 84) | 12.505 (5.433 - 21.038) | 0.135 (0.04-0.23) |
|  | Male | 1 (1 - 2) | 1.257 (0.712 - 1.982) | 5 (3 - 10) | 1.24 (0.704 - 2.27) | -0.194 (-0.249to-0.139) | 1 (1 - 2) | 1.196 (0.677 - 1.886) | 5 (3 - 9) | 1.133 (0.645 - 2.057) | -0.317 (-0.39to-0.245) | 34 (19 - 54) | 35.256 (19.814 - 56.694) | 152 (84 - 291) | 32.77 (18.337 - 61.294) | -0.388 (-0.473to-0.302) |
| Eritrea | Female | 4 (1 - 6) | 0.457 (0.192 - 0.732) | 10 (4 - 14) | 0.529 (0.233 - 0.759) | 0.539 (0.501-0.578) | 3 (1 - 5) | 0.433 (0.185 - 0.688) | 9 (4 - 13) | 0.483 (0.21 - 0.699) | 0.441 (0.39-0.493) | 111 (43 - 183) | 13.009 (5.238 - 21.131) | 287 (125 - 427) | 14.185 (6.139 - 20.852) | 0.365 (0.318-0.412) |
|  | Male | 9 (6 - 11) | 1.293 (0.89 - 1.753) | 18 (12 - 27) | 1.21 (0.824 - 1.746) | -0.55 (-0.763to-0.338) | 8 (5 - 11) | 1.247 (0.87 - 1.668) | 16 (11 - 24) | 1.142 (0.784 - 1.623) | -0.603 (-0.818to-0.388) | 280 (187 - 380) | 38.543 (26.642 - 51.418) | 574 (376 - 848) | 34.242 (23.018 - 49.884) | -0.718 (-0.938to-0.497) |
| Ethiopia | Female | 43 (24 - 81) | 0.409 (0.23 - 0.742) | 93 (60 - 127) | 0.386 (0.252 - 0.534) | -0.354 (-0.449to-0.26) | 40 (22 - 75) | 0.399 (0.229 - 0.713) | 77 (51 - 106) | 0.337 (0.219 - 0.464) | -0.69 (-0.759to-0.621) | 1323 (724 - 2551) | 11.487 (6.355 - 21.53) | 2514 (1619 - 3618) | 9.586 (6.194 - 13.172) | -0.754 (-0.828to-0.679) |
|  | Male | 54 (35 - 92) | 0.459 (0.301 - 0.777) | 92 (54 - 155) | 0.379 (0.225 - 0.641) | -0.731 (-1.057to-0.403) | 52 (34 - 88) | 0.452 (0.298 - 0.759) | 82 (48 - 140) | 0.35 (0.208 - 0.598) | -0.937 (-1.234to-0.638) | 1665 (1072 - 2857) | 13.484 (8.808 - 22.995) | 2574 (1505 - 4393) | 10.025 (5.859 - 17.076) | -1.091 (-1.392to-0.789) |
| Kenya | Female | 21 (14 - 31) | 0.463 (0.315 - 0.67) | 78 (48 - 114) | 0.566 (0.353 - 0.817) | 0.956 (0.817-1.096) | 18 (13 - 27) | 0.415 (0.284 - 0.601) | 63 (39 - 93) | 0.48 (0.301 - 0.696) | 0.861 (0.699-1.024) | 589 (402 - 866) | 11.905 (8.122 - 17.394) | 2056 (1252 - 3033) | 13.863 (8.513 - 20.341) | 0.872 (0.713-1.032) |
|  | Male | 31 (20 - 45) | 0.702 (0.446 - 1.031) | 136 (90 - 190) | 1.072 (0.715 - 1.5) | 1.523 (1.303-1.743) | 28 (18 - 41) | 0.652 (0.407 - 0.957) | 116 (77 - 163) | 0.961 (0.64 - 1.344) | 1.457 (1.21-1.705) | 878 (557 - 1294) | 18.937 (12.002 - 27.918) | 3771 (2477 - 5360) | 27.784 (18.403 - 39.18) | 1.443 (1.17-1.716) |
| Madagascar | Female | 12 (5 - 16) | 0.416 (0.184 - 0.557) | 28 (12 - 42) | 0.385 (0.172 - 0.568) | -0.208 (-0.325to-0.09) | 11 (5 - 14) | 0.385 (0.174 - 0.514) | 23 (10 - 35) | 0.341 (0.152 - 0.504) | -0.325 (-0.435to-0.214) | 351 (146 - 477) | 11.531 (4.919 - 15.508) | 829 (360 - 1267) | 10.276 (4.516 - 15.58) | -0.334 (-0.438to-0.23) |
|  | Male | 27 (18 - 39) | 0.961 (0.664 - 1.387) | 49 (31 - 81) | 0.745 (0.478 - 1.211) | -0.836 (-0.958to-0.714) | 25 (17 - 36) | 0.921 (0.638 - 1.311) | 44 (28 - 73) | 0.695 (0.443 - 1.116) | -0.912 (-1.023to-0.801) | 793 (552 - 1129) | 27.398 (19.068 - 38.939) | 1475 (924 - 2439) | 20.496 (12.866 - 33.816) | -0.946 (-1.054to-0.839) |
| Malawi | Female | 1 (0 - 1) | 0.025 (0.016 - 0.033) | 1 (1 - 2) | 0.028 (0.015 - 0.041) | 0.419 (0.345-0.494) | 1 (0 - 1) | 0.023 (0.015 - 0.031) | 1 (1 - 2) | 0.024 (0.013 - 0.036) | 0.228 (0.131-0.324) | 17 (10 - 22) | 0.678 (0.424 - 0.905) | 36 (18 - 55) | 0.711 (0.374 - 1.071) | 0.196 (0.106-0.285) |
|  | Male | 4 (3 - 5) | 0.179 (0.129 - 0.248) | 10 (6 - 16) | 0.261 (0.165 - 0.405) | 1.243 (0.968-1.519) | 3 (2 - 5) | 0.173 (0.126 - 0.239) | 9 (6 - 14) | 0.24 (0.155 - 0.37) | 1.085 (0.796-1.374) | 109 (77 - 154) | 5.099 (3.638 - 7.165) | 307 (186 - 477) | 7.184 (4.426 - 11.181) | 1.112 (0.809-1.416) |
| Mozambique | Female | 16 (7 - 22) | 0.45 (0.194 - 0.617) | 37 (14 - 58) | 0.517 (0.196 - 0.791) | 0.63 (0.57-0.691) | 15 (6 - 20) | 0.425 (0.185 - 0.577) | 32 (12 - 51) | 0.47 (0.185 - 0.727) | 0.56 (0.481-0.639) | 484 (200 - 672) | 12.65 (5.334 - 17.335) | 1092 (418 - 1737) | 13.88 (5.342 - 21.734) | 0.509 (0.438-0.58) |
|  | Male | 17 (12 - 23) | 0.544 (0.398 - 0.737) | 45 (32 - 60) | 0.79 (0.574 - 1.041) | 1.669 (1.499-1.839) | 16 (12 - 22) | 0.538 (0.396 - 0.725) | 41 (29 - 55) | 0.756 (0.552 - 0.995) | 1.573 (1.396-1.75) | 491 (356 - 681) | 14.909 (10.852 - 20.505) | 1318 (926 - 1811) | 21.499 (15.416 - 28.7) | 1.683 (1.492-1.874) |
| Rwanda | Female | 9 (3 - 14) | 0.506 (0.201 - 0.781) | 20 (9 - 31) | 0.49 (0.222 - 0.747) | -0.354 (-0.461to-0.247) | 8 (3 - 13) | 0.484 (0.194 - 0.74) | 17 (8 - 26) | 0.424 (0.195 - 0.652) | -0.694 (-0.786to-0.602) | 278 (104 - 435) | 14.598 (5.608 - 22.724) | 544 (244 - 851) | 12.349 (5.561 - 19.177) | -0.845 (-0.955to-0.736) |
|  | Male | 24 (18 - 33) | 1.712 (1.288 - 2.333) | 39 (24 - 63) | 1.269 (0.791 - 2.051) | -1.778 (-2.111to-1.443) | 23 (17 - 32) | 1.669 (1.265 - 2.269) | 35 (21 - 57) | 1.171 (0.727 - 1.91) | -1.948 (-2.267to-1.627) | 744 (551 - 1023) | 50.307 (37.478 - 68.654) | 1126 (680 - 1869) | 33.602 (20.518 - 55.024) | -2.178 (-2.519to-1.835) |
| Somalia | Female | 6 (3 - 12) | 0.429 (0.18 - 0.756) | 19 (8 - 29) | 0.456 (0.198 - 0.706) | 0.264 (0.236-0.293) | 6 (2 - 11) | 0.409 (0.179 - 0.712) | 17 (7 - 26) | 0.434 (0.187 - 0.664) | 0.27 (0.232-0.309) | 207 (82 - 374) | 12.156 (5.028 - 21.884) | 576 (243 - 897) | 12.691 (5.407 - 19.589) | 0.191 (0.158-0.224) |
|  | Male | 13 (8 - 21) | 0.941 (0.589 - 1.475) | 25 (15 - 44) | 0.758 (0.46 - 1.362) | -0.733 (-0.773to-0.693) | 12 (8 - 20) | 0.917 (0.579 - 1.446) | 23 (14 - 41) | 0.737 (0.447 - 1.301) | -0.733 (-0.77to-0.695) | 426 (262 - 682) | 27.629 (17.011 - 44.443) | 793 (466 - 1433) | 21.944 (13.246 - 39.484) | -0.807 (-0.85to-0.764) |
| South Sudan | Female | 5 (2 - 7) | 0.412 (0.191 - 0.626) | 10 (4 - 16) | 0.451 (0.205 - 0.678) | 0.289 (0.16-0.418) | 4 (2 - 6) | 0.382 (0.184 - 0.577) | 8 (4 - 13) | 0.402 (0.184 - 0.596) | 0.179 (0.07-0.288) | 137 (61 - 212) | 11.223 (5.198 - 17.562) | 291 (123 - 467) | 11.726 (5.094 - 18.104) | 0.148 (0.017-0.28) |
|  | Male | 18 (11 - 28) | 1.116 (0.676 - 1.716) | 23 (15 - 36) | 0.976 (0.627 - 1.504) | -0.526 (-0.648to-0.404) | 17 (10 - 26) | 1.072 (0.645 - 1.654) | 21 (13 - 32) | 0.911 (0.583 - 1.377) | -0.615 (-0.724to-0.506) | 517 (307 - 801) | 31.564 (18.793 - 49.011) | 682 (430 - 1068) | 26.742 (17.203 - 41.162) | -0.641 (-0.765to-0.516) |
| Uganda | Female | 35 (23 - 51) | 0.982 (0.654 - 1.456) | 114 (73 - 172) | 1.221 (0.809 - 1.831) | 0.449 (0.239-0.659) | 31 (20 - 47) | 0.916 (0.612 - 1.378) | 95 (61 - 141) | 1.068 (0.71 - 1.573) | 0.263 (0.03-0.498) | 971 (633 - 1449) | 25.642 (16.75 - 37.736) | 3083 (1902 - 4691) | 30.092 (19.426 - 45.451) | 0.201 (-0.051-0.453) |
|  | Male | 73 (51 - 105) | 2.122 (1.497 - 3.006) | 213 (135 - 317) | 2.783 (1.829 - 4.026) | 0.516 (0.274-0.76) | 68 (48 - 97) | 2.023 (1.425 - 2.844) | 186 (118 - 278) | 2.541 (1.681 - 3.667) | 0.391 (0.129-0.653) | 2152 (1458 - 3117) | 59.559 (40.796 - 85.531) | 6202 (3909 - 9362) | 74.488 (47.314 - 111.352) | 0.318 (0.04-0.597) |
| United Republic of Tanzania | Female | 28 (13 - 40) | 0.462 (0.21 - 0.638) | 70 (30 - 103) | 0.464 (0.203 - 0.676) | 0.013 (-0.035-0.061) | 25 (11 - 35) | 0.422 (0.19 - 0.582) | 58 (26 - 85) | 0.398 (0.182 - 0.585) | -0.145 (-0.179to-0.11) | 821 (349 - 1172) | 12.453 (5.392 - 17.698) | 1894 (835 - 2841) | 11.584 (5.17 - 17.15) | -0.21 (-0.247to-0.174) |
|  | Male | 70 (46 - 118) | 1.205 (0.798 - 2.006) | 151 (89 - 262) | 1.08 (0.647 - 1.861) | -0.602 (-0.686to-0.518) | 65 (43 - 110) | 1.142 (0.762 - 1.903) | 133 (79 - 230) | 0.983 (0.6 - 1.7) | -0.708 (-0.793to-0.623) | 2055 (1327 - 3508) | 33.964 (22.121 - 57.901) | 4294 (2474 - 7484) | 28.891 (16.992 - 50.028) | -0.752 (-0.839to-0.665) |
| Zambia | Female | 7 (3 - 10) | 0.467 (0.197 - 0.636) | 34 (11 - 56) | 0.769 (0.269 - 1.241) | 2.041 (1.665-2.418) | 7 (3 - 9) | 0.435 (0.186 - 0.592) | 28 (9 - 46) | 0.654 (0.227 - 1.055) | 1.745 (1.427-2.064) | 233 (92 - 325) | 13.089 (5.422 - 18.18) | 999 (309 - 1690) | 20.212 (6.615 - 33.049) | 1.843 (1.491-2.196) |
|  | Male | 19 (14 - 27) | 1.182 (0.853 - 1.643) | 82 (33 - 213) | 1.971 (0.815 - 5.083) | 1.828 (1.642-2.015) | 18 (13 - 26) | 1.139 (0.824 - 1.599) | 72 (28 - 188) | 1.788 (0.748 - 4.516) | 1.629 (1.466-1.792) | 584 (432 - 812) | 33.848 (24.741 - 47.379) | 2477 (961 - 6579) | 54.444 (21.712 - 142.825) | 1.725 (1.55-1.9) |
| Botswana | Female | 1 (1 - 2) | 0.401 (0.25 - 0.588) | 4 (2 - 6) | 0.411 (0.234 - 0.623) | 0.329 (0.094-0.564) | 1 (1 - 2) | 0.36 (0.225 - 0.522) | 3 (2 - 5) | 0.341 (0.193 - 0.518) | 0.066 (-0.128-0.26) | 37 (23 - 55) | 10.376 (6.326 - 15.136) | 93 (50 - 146) | 9.678 (5.327 - 15.003) | 0.015 (-0.193-0.224) |
|  | Male | 3 (2 - 5) | 1.163 (0.775 - 1.675) | 9 (5 - 16) | 1.236 (0.719 - 2.04) | -0.249 (-0.681-0.186) | 3 (2 - 4) | 1.089 (0.734 - 1.558) | 8 (4 - 13) | 1.1 (0.646 - 1.78) | -0.402 (-0.874-0.072) | 94 (61 - 140) | 31.824 (20.932 - 46.346) | 255 (142 - 442) | 31.433 (17.926 - 53.092) | -0.53 (-1.02to-0.039) |
| Eswatini | Female | 1 (0 - 1) | 0.393 (0.268 - 0.562) | 2 (1 - 3) | 0.461 (0.263 - 0.728) | 0.651 (0.443-0.86) | 1 (0 - 1) | 0.354 (0.244 - 0.502) | 1 (1 - 2) | 0.398 (0.231 - 0.619) | 0.555 (0.299-0.812) | 19 (13 - 27) | 10.098 (6.84 - 14.404) | 44 (23 - 71) | 11.442 (6.244 - 18.328) | 0.563 (0.284-0.843) |
|  | Male | 2 (1 - 2) | 1.154 (0.787 - 1.616) | 5 (3 - 7) | 1.72 (1.016 - 2.658) | 1.596 (1.15-2.044) | 2 (1 - 2) | 1.082 (0.743 - 1.512) | 4 (2 - 6) | 1.545 (0.924 - 2.363) | 1.485 (1.013-1.959) | 50 (33 - 69) | 31.429 (21.097 - 43.989) | 141 (79 - 217) | 46.822 (27.426 - 72.177) | 1.603 (1.084-2.126) |
| Lesotho | Female | 2 (1 - 3) | 0.332 (0.22 - 0.492) | 3 (2 - 5) | 0.453 (0.259 - 0.743) | 1.42 (1.182-1.659) | 2 (1 - 2) | 0.305 (0.205 - 0.454) | 3 (2 - 5) | 0.413 (0.245 - 0.67) | 1.438 (1.181-1.697) | 49 (32 - 72) | 8.585 (5.703 - 12.654) | 84 (47 - 138) | 11.779 (6.642 - 19.344) | 1.491 (1.22-1.762) |
|  | Male | 3 (2 - 4) | 0.824 (0.56 - 1.247) | 8 (6 - 10) | 1.574 (1.166 - 2.137) | 2.38 (2.066-2.695) | 3 (2 - 4) | 0.787 (0.539 - 1.166) | 7 (5 - 10) | 1.473 (1.096 - 2.006) | 2.342 (2.017-2.669) | 77 (51 - 118) | 22.389 (14.972 - 34.155) | 229 (166 - 317) | 44.273 (32.425 - 61.068) | 2.517 (2.146-2.889) |
| Namibia | Female | 3 (2 - 4) | 0.806 (0.604 - 1.093) | 7 (5 - 11) | 0.863 (0.527 - 1.306) | 0.101 (0.003-0.198) | 3 (2 - 4) | 0.727 (0.548 - 0.983) | 6 (4 - 9) | 0.696 (0.431 - 1.05) | -0.24 (-0.367to-0.112) | 83 (61 - 112) | 20.876 (15.481 - 28.276) | 180 (108 - 279) | 19.841 (11.989 - 30.799) | -0.29 (-0.427to-0.154) |
|  | Male | 6 (4 - 8) | 1.778 (1.231 - 2.469) | 18 (12 - 24) | 2.543 (1.822 - 3.478) | 1.16 (0.942-1.378) | 5 (4 - 7) | 1.66 (1.151 - 2.301) | 15 (10 - 20) | 2.181 (1.576 - 2.958) | 0.902 (0.66-1.144) | 173 (118 - 241) | 49.467 (33.909 - 68.643) | 483 (339 - 679) | 65.456 (46.334 - 91.289) | 0.883 (0.606-1.16) |
| South Africa | Female | 39 (28 - 52) | 0.312 (0.224 - 0.42) | 106 (75 - 130) | 0.382 (0.269 - 0.464) | 1.019 (0.815-1.223) | 32 (23 - 42) | 0.258 (0.183 - 0.347) | 78 (56 - 95) | 0.286 (0.203 - 0.346) | 0.654 (0.518-0.79) | 996 (731 - 1305) | 7.615 (5.493 - 10.176) | 2303 (1704 - 2890) | 8.09 (5.95 - 10.048) | 0.583 (0.403-0.764) |
|  | Male | 107 (85 - 142) | 1.086 (0.858 - 1.456) | 317 (265 - 377) | 1.439 (1.216 - 1.698) | 0.926 (0.721-1.131) | 91 (72 - 122) | 0.955 (0.753 - 1.295) | 250 (211 - 296) | 1.181 (1.004 - 1.383) | 0.689 (0.423-0.955) | 2999 (2402 - 3941) | 28.831 (22.924 - 38.587) | 7909 (6589 - 9482) | 34.289 (28.824 - 40.746) | 0.549 (0.293-0.806) |
| Zimbabwe | Female | 5 (3 - 6) | 0.22 (0.151 - 0.293) | 14 (7 - 20) | 0.308 (0.166 - 0.438) | 1.67 (1.134-2.208) | 4 (3 - 6) | 0.194 (0.135 - 0.258) | 12 (6 - 17) | 0.276 (0.148 - 0.388) | 1.846 (1.305-2.389) | 125 (85 - 169) | 5.367 (3.675 - 7.2) | 387 (197 - 558) | 8.016 (4.096 - 11.27) | 2.06 (1.461-2.662) |
|  | Male | 12 (9 - 16) | 0.564 (0.435 - 0.737) | 30 (21 - 42) | 0.844 (0.606 - 1.161) | 1.086 (0.61-1.564) | 11 (8 - 15) | 0.521 (0.402 - 0.682) | 27 (19 - 37) | 0.778 (0.558 - 1.072) | 1.172 (0.723-1.624) | 344 (260 - 464) | 14.902 (11.32 - 19.822) | 921 (644 - 1312) | 23.877 (16.906 - 33.277) | 1.411 (0.893-1.932) |
| Benin | Female | 1 (0 - 1) | 0.055 (0.036 - 0.073) | 1 (1 - 2) | 0.049 (0.03 - 0.066) | -0.528 (-0.652to-0.403) | 1 (0 - 1) | 0.054 (0.036 - 0.072) | 1 (1 - 2) | 0.047 (0.029 - 0.063) | -0.546 (-0.653to-0.44) | 16 (10 - 21) | 1.377 (0.888 - 1.805) | 36 (22 - 49) | 1.108 (0.687 - 1.491) | -0.923 (-1.066to-0.78) |
|  | Male | 2 (1 - 3) | 0.184 (0.132 - 0.254) | 6 (4 - 9) | 0.209 (0.138 - 0.308) | 0.254 (0.122-0.386) | 2 (1 - 2) | 0.176 (0.127 - 0.243) | 5 (3 - 8) | 0.19 (0.126 - 0.279) | 0.106 (-0.027-0.239) | 54 (39 - 74) | 5.28 (3.783 - 7.234) | 176 (114 - 262) | 5.735 (3.757 - 8.509) | 0.129 (-0.007-0.266) |
| Burkina Faso | Female | 1 (1 - 2) | 0.063 (0.039 - 0.086) | 3 (2 - 4) | 0.054 (0.033 - 0.073) | -0.707 (-0.887to-0.528) | 1 (1 - 2) | 0.063 (0.04 - 0.086) | 2 (2 - 3) | 0.053 (0.033 - 0.074) | -0.683 (-0.852to-0.514) | 38 (22 - 53) | 1.55 (0.927 - 2.137) | 68 (41 - 94) | 1.207 (0.753 - 1.655) | -1.002 (-1.213to-0.791) |
|  | Male | 4 (3 - 6) | 0.184 (0.13 - 0.268) | 12 (8 - 18) | 0.24 (0.166 - 0.358) | 0.793 (0.666-0.92) | 4 (3 - 6) | 0.175 (0.125 - 0.257) | 11 (7 - 16) | 0.221 (0.151 - 0.328) | 0.689 (0.56-0.818) | 123 (84 - 178) | 5.302 (3.655 - 7.629) | 361 (236 - 544) | 6.773 (4.532 - 10.169) | 0.732 (0.593-0.87) |
| Cabo Verde | Female | 0 (0 - 0) | 0.019 (0.011 - 0.059) | 0 (0 - 1) | 0.153 (0.04 - 0.231) | 5.53 (3.896-7.19) | 0 (0 - 0) | 0.019 (0.011 - 0.054) | 0 (0 - 1) | 0.135 (0.031 - 0.208) | 5.102 (3.408-6.824) | 1 (0 - 2) | 0.393 (0.208 - 1.296) | 6 (2 - 10) | 2.452 (0.75 - 3.611) | 4.696 (3.062-6.355) |
|  | Male | 0 (0 - 0) | 0.045 (0.033 - 0.058) | 2 (2 - 3) | 0.967 (0.682 - 1.331) | 8.464 (6.246-10.727) | 0 (0 - 0) | 0.038 (0.028 - 0.049) | 2 (1 - 2) | 0.717 (0.503 - 0.984) | 7.882 (5.625-10.186) | 1 (1 - 1) | 1.238 (0.898 - 1.617) | 54 (38 - 76) | 22.126 (15.828 - 31.079) | 7.782 (5.528-10.084) |
| Cameroon | Female | 1 (1 - 2) | 0.063 (0.04 - 0.085) | 4 (2 - 5) | 0.053 (0.032 - 0.077) | -0.686 (-0.829to-0.542) | 1 (1 - 2) | 0.061 (0.04 - 0.082) | 3 (2 - 4) | 0.05 (0.03 - 0.072) | -0.718 (-0.816to-0.619) | 41 (24 - 56) | 1.54 (0.968 - 2.081) | 93 (52 - 139) | 1.183 (0.704 - 1.732) | -1.022 (-1.153to-0.892) |
|  | Male | 6 (4 - 8) | 0.228 (0.16 - 0.315) | 21 (12 - 31) | 0.278 (0.165 - 0.421) | 0.492 (0.388-0.596) | 5 (4 - 7) | 0.214 (0.152 - 0.291) | 18 (10 - 27) | 0.247 (0.148 - 0.371) | 0.333 (0.206-0.46) | 170 (117 - 236) | 6.494 (4.505 - 8.925) | 609 (354 - 946) | 7.663 (4.469 - 11.701) | 0.402 (0.27-0.535) |
| Chad | Female | 1 (0 - 1) | 0.051 (0.033 - 0.07) | 1 (1 - 2) | 0.054 (0.037 - 0.076) | 0.077 (0.017-0.136) | 1 (0 - 1) | 0.051 (0.033 - 0.07) | 1 (1 - 2) | 0.054 (0.037 - 0.077) | 0.128 (0.083-0.173) | 20 (13 - 28) | 1.296 (0.846 - 1.766) | 41 (27 - 58) | 1.268 (0.845 - 1.771) | -0.18 (-0.245to-0.115) |
|  | Male | 2 (1 - 3) | 0.137 (0.096 - 0.19) | 8 (5 - 12) | 0.221 (0.142 - 0.333) | 1.485 (1.32-1.651) | 2 (1 - 3) | 0.132 (0.093 - 0.182) | 7 (5 - 11) | 0.207 (0.137 - 0.31) | 1.39 (1.207-1.574) | 57 (40 - 81) | 3.974 (2.751 - 5.572) | 244 (154 - 381) | 6.406 (4.084 - 9.829) | 1.496 (1.312-1.681) |
| Côte d'Ivoire | Female | 1 (1 - 2) | 0.056 (0.038 - 0.074) | 3 (2 - 5) | 0.055 (0.034 - 0.081) | 0.117 (0.001-0.233) | 1 (1 - 1) | 0.053 (0.037 - 0.072) | 3 (2 - 4) | 0.049 (0.031 - 0.071) | -0.097 (-0.185to-0.01) | 33 (21 - 45) | 1.409 (0.919 - 1.901) | 87 (48 - 135) | 1.297 (0.746 - 1.95) | -0.086 (-0.199-0.027) |
|  | Male | 9 (7 - 13) | 0.361 (0.269 - 0.488) | 27 (16 - 48) | 0.377 (0.234 - 0.645) | -0.244 (-0.402to-0.086) | 8 (6 - 12) | 0.341 (0.252 - 0.457) | 23 (14 - 41) | 0.338 (0.212 - 0.582) | -0.402 (-0.568to-0.235) | 284 (203 - 393) | 10.201 (7.366 - 13.963) | 787 (455 - 1396) | 10.12 (6.044 - 17.935) | -0.415 (-0.592to-0.237) |
| Gambia | Female | 0 (0 - 0) | 0.116 (0.08 - 0.156) | 1 (0 - 1) | 0.129 (0.084 - 0.187) | 0.095 (-0.108-0.299) | 0 (0 - 0) | 0.108 (0.074 - 0.148) | 1 (0 - 1) | 0.112 (0.076 - 0.163) | -0.064 (-0.235-0.106) | 6 (4 - 8) | 2.934 (1.945 - 3.997) | 19 (12 - 27) | 3.071 (1.961 - 4.407) | -0.106 (-0.324-0.113) |
|  | Male | 1 (0 - 1) | 0.302 (0.219 - 0.405) | 2 (1 - 3) | 0.345 (0.23 - 0.5) | 0.311 (0.168-0.454) | 1 (0 - 1) | 0.283 (0.205 - 0.381) | 2 (1 - 2) | 0.309 (0.207 - 0.442) | 0.177 (0.05-0.305) | 19 (13 - 26) | 8.364 (6.004 - 11.65) | 52 (34 - 76) | 9.152 (5.937 - 13.282) | 0.165 (0.023-0.306) |
| Ghana | Female | 0 (0 - 0) | 0.01 (0.005 - 0.014) | 1 (0 - 1) | 0.011 (0.005 - 0.016) | 0.305 (0.239-0.372) | 0 (0 - 0) | 0.01 (0.005 - 0.015) | 1 (0 - 1) | 0.011 (0.005 - 0.016) | 0.18 (0.141-0.219) | 7 (5 - 10) | 0.21 (0.127 - 0.293) | 21 (12 - 30) | 0.211 (0.111 - 0.309) | -0.001 (-0.064-0.061) |
|  | Male | 2 (1 - 3) | 0.057 (0.04 - 0.082) | 2 (1 - 3) | 0.02 (0.013 - 0.029) | -5.085 (-6.229to-3.927) | 2 (1 - 3) | 0.052 (0.037 - 0.074) | 1 (1 - 2) | 0.018 (0.012 - 0.024) | -5.247 (-6.373to-4.107) | 62 (43 - 91) | 1.631 (1.144 - 2.365) | 51 (34 - 73) | 0.545 (0.357 - 0.771) | -5.256 (-6.382to-4.116) |
| Guinea | Female | 4 (2 - 5) | 0.219 (0.147 - 0.302) | 7 (4 - 10) | 0.225 (0.136 - 0.327) | 0.064 (0.026-0.102) | 3 (2 - 5) | 0.21 (0.14 - 0.292) | 6 (4 - 9) | 0.208 (0.127 - 0.311) | -0.019 (-0.051-0.013) | 105 (68 - 144) | 5.846 (3.854 - 7.915) | 197 (108 - 286) | 5.768 (3.29 - 8.192) | -0.055 (-0.095to-0.015) |
|  | Male | 6 (5 - 8) | 0.38 (0.274 - 0.492) | 15 (10 - 22) | 0.488 (0.34 - 0.725) | 0.86 (0.775-0.945) | 6 (4 - 8) | 0.369 (0.265 - 0.473) | 14 (10 - 20) | 0.459 (0.321 - 0.671) | 0.771 (0.678-0.865) | 188 (134 - 244) | 10.792 (7.673 - 13.97) | 433 (293 - 645) | 13.562 (9.304 - 20.181) | 0.802 (0.705-0.899) |
| Guinea-Bissau | Female | 0 (0 - 0) | 0.062 (0.039 - 0.091) | 0 (0 - 0) | 0.061 (0.039 - 0.085) | -0.171 (-0.254to-0.088) | 0 (0 - 0) | 0.062 (0.039 - 0.09) | 0 (0 - 0) | 0.061 (0.039 - 0.085) | -0.162 (-0.231to-0.094) | 4 (2 - 6) | 1.66 (0.999 - 2.465) | 8 (5 - 11) | 1.49 (0.968 - 2.077) | -0.496 (-0.579to-0.413) |
|  | Male | 1 (0 - 1) | 0.272 (0.158 - 0.39) | 1 (1 - 2) | 0.304 (0.212 - 0.427) | 0.239 (0.164-0.314) | 1 (0 - 1) | 0.263 (0.156 - 0.374) | 1 (1 - 2) | 0.283 (0.197 - 0.392) | 0.128 (0.045-0.211) | 18 (10 - 26) | 8.166 (4.734 - 11.843) | 40 (27 - 58) | 8.944 (6.051 - 12.546) | 0.187 (0.108-0.267) |
| Liberia | Female | 0 (0 - 0) | 0.053 (0.033 - 0.077) | 1 (0 - 1) | 0.052 (0.031 - 0.078) | -0.117 (-0.24-0.006) | 0 (0 - 0) | 0.053 (0.033 - 0.076) | 0 (0 - 1) | 0.049 (0.03 - 0.073) | -0.305 (-0.414to-0.195) | 8 (4 - 11) | 1.325 (0.798 - 1.892) | 15 (9 - 23) | 1.157 (0.688 - 1.728) | -0.555 (-0.702to-0.408) |
|  | Male | 1 (1 - 2) | 0.174 (0.114 - 0.272) | 3 (1 - 5) | 0.19 (0.101 - 0.352) | 0.314 (0.135-0.493) | 1 (1 - 2) | 0.167 (0.11 - 0.256) | 2 (1 - 4) | 0.168 (0.09 - 0.313) | 0.035 (-0.134-0.204) | 33 (21 - 52) | 4.998 (3.217 - 7.896) | 76 (39 - 146) | 5.167 (2.716 - 9.759) | 0.148 (-0.034-0.33) |
| Mali | Female | 2 (1 - 3) | 0.094 (0.063 - 0.122) | 4 (2 - 6) | 0.087 (0.052 - 0.125) | -0.212 (-0.326to-0.098) | 2 (1 - 2) | 0.089 (0.06 - 0.117) | 4 (2 - 5) | 0.078 (0.047 - 0.114) | -0.348 (-0.453to-0.242) | 60 (35 - 78) | 2.546 (1.584 - 3.316) | 118 (65 - 173) | 2.188 (1.252 - 3.146) | -0.435 (-0.549to-0.321) |
|  | Male | 9 (7 - 11) | 0.386 (0.302 - 0.509) | 21 (14 - 30) | 0.411 (0.272 - 0.578) | 0.502 (0.258-0.746) | 8 (6 - 11) | 0.373 (0.293 - 0.493) | 19 (12 - 27) | 0.383 (0.257 - 0.542) | 0.384 (0.147-0.623) | 253 (197 - 337) | 10.904 (8.531 - 14.523) | 596 (397 - 858) | 11.103 (7.376 - 15.928) | 0.351 (0.11-0.592) |
| Mauritania | Female | 0 (0 - 0) | 0.059 (0.037 - 0.076) | 1 (0 - 1) | 0.058 (0.037 - 0.082) | -0.255 (-0.377to-0.133) | 0 (0 - 0) | 0.058 (0.036 - 0.075) | 1 (0 - 1) | 0.051 (0.032 - 0.075) | -0.535 (-0.615to-0.456) | 8 (5 - 10) | 1.436 (0.902 - 1.889) | 14 (9 - 21) | 1.184 (0.758 - 1.702) | -0.855 (-0.95to-0.761) |
|  | Male | 1 (1 - 1) | 0.188 (0.127 - 0.274) | 2 (1 - 5) | 0.207 (0.112 - 0.424) | 0.04 (-0.156-0.236) | 1 (1 - 1) | 0.177 (0.122 - 0.258) | 2 (1 - 4) | 0.174 (0.095 - 0.366) | -0.3 (-0.46to-0.139) | 29 (20 - 42) | 5.354 (3.66 - 7.822) | 63 (34 - 138) | 5.22 (2.82 - 11.462) | -0.318 (-0.479to-0.158) |
| Niger | Female | 1 (0 - 1) | 0.053 (0.033 - 0.072) | 2 (1 - 3) | 0.051 (0.034 - 0.069) | -0.296 (-0.403to-0.189) | 1 (0 - 1) | 0.053 (0.034 - 0.073) | 2 (1 - 3) | 0.051 (0.034 - 0.07) | -0.272 (-0.37to-0.174) | 22 (13 - 30) | 1.349 (0.821 - 1.851) | 56 (37 - 78) | 1.162 (0.779 - 1.575) | -0.731 (-0.868to-0.594) |
|  | Male | 2 (1 - 3) | 0.131 (0.088 - 0.19) | 6 (4 - 11) | 0.139 (0.084 - 0.24) | 0.286 (0.187-0.385) | 2 (1 - 3) | 0.126 (0.087 - 0.183) | 6 (4 - 10) | 0.13 (0.08 - 0.223) | 0.166 (0.072-0.26) | 66 (44 - 98) | 3.785 (2.585 - 5.609) | 191 (112 - 332) | 3.904 (2.313 - 6.803) | 0.176 (0.067-0.285) |
| Nigeria | Female | 3 (2 - 5) | 0.012 (0.008 - 0.023) | 8 (5 - 14) | 0.013 (0.008 - 0.026) | 0.512 (0.353-0.673) | 3 (2 - 5) | 0.011 (0.008 - 0.023) | 6 (4 - 11) | 0.012 (0.007 - 0.025) | 0.28 (0.151-0.409) | 79 (54 - 129) | 0.325 (0.22 - 0.569) | 222 (128 - 358) | 0.333 (0.198 - 0.584) | 0.209 (0.062-0.357) |
|  | Male | 94 (62 - 135) | 0.345 (0.229 - 0.489) | 203 (144 - 282) | 0.389 (0.277 - 0.541) | 0.554 (0.376-0.732) | 86 (57 - 124) | 0.326 (0.218 - 0.46) | 173 (123 - 240) | 0.345 (0.249 - 0.476) | 0.345 (0.187-0.504) | 2945 (1956 - 4250) | 10.127 (6.753 - 14.533) | 6023 (4252 - 8338) | 10.743 (7.612 - 14.967) | 0.344 (0.181-0.508) |
| Sao Tome and Principe | Female | 0 (0 - 0) | 0.023 (0.012 - 0.033) | 0 (0 - 0) | 0.026 (0.015 - 0.038) | 0.419 (0.381-0.457) | 0 (0 - 0) | 0.024 (0.012 - 0.035) | 0 (0 - 0) | 0.025 (0.013 - 0.038) | 0.118 (0.078-0.158) | 0 (0 - 0) | 0.47 (0.291 - 0.634) | 0 (0 - 0) | 0.488 (0.311 - 0.696) | 0.095 (0.019-0.17) |
|  | Male | 0 (0 - 0) | 0.007 (0.005 - 0.01) | 0 (0 - 0) | 0.009 (0.006 - 0.014) | 0.943 (0.815-1.071) | 0 (0 - 0) | 0.007 (0.005 - 0.009) | 0 (0 - 0) | 0.008 (0.005 - 0.012) | 0.654 (0.547-0.76) | 0 (0 - 0) | 0.205 (0.138 - 0.287) | 0 (0 - 0) | 0.231 (0.144 - 0.368) | 0.561 (0.472-0.65) |
| Senegal | Female | 1 (1 - 1) | 0.055 (0.034 - 0.073) | 2 (2 - 3) | 0.057 (0.038 - 0.079) | 0.018 (-0.092-0.127) | 1 (1 - 1) | 0.054 (0.034 - 0.072) | 2 (1 - 3) | 0.054 (0.035 - 0.077) | -0.026 (-0.123-0.071) | 25 (15 - 34) | 1.374 (0.824 - 1.815) | 58 (38 - 83) | 1.269 (0.83 - 1.788) | -0.322 (-0.442to-0.203) |
|  | Male | 3 (2 - 5) | 0.192 (0.143 - 0.259) | 10 (6 - 15) | 0.237 (0.153 - 0.355) | 0.583 (0.49-0.677) | 3 (2 - 4) | 0.181 (0.136 - 0.245) | 9 (6 - 13) | 0.211 (0.138 - 0.312) | 0.427 (0.34-0.514) | 99 (73 - 138) | 5.484 (4.047 - 7.622) | 282 (180 - 443) | 6.431 (4.139 - 9.98) | 0.476 (0.384-0.568) |
| Sierra Leone | Female | 0 (0 - 1) | 0.048 (0.03 - 0.066) | 1 (1 - 1) | 0.05 (0.031 - 0.068) | 0.056 (-0.02-0.132) | 0 (0 - 1) | 0.048 (0.03 - 0.065) | 1 (1 - 1) | 0.048 (0.029 - 0.066) | 0.015 (-0.033-0.063) | 13 (8 - 18) | 1.196 (0.71 - 1.679) | 26 (15 - 37) | 1.142 (0.691 - 1.561) | -0.189 (-0.259to-0.119) |
|  | Male | 2 (1 - 3) | 0.184 (0.126 - 0.267) | 5 (3 - 7) | 0.202 (0.122 - 0.324) | 0.304 (0.211-0.397) | 2 (1 - 3) | 0.175 (0.121 - 0.252) | 4 (2 - 7) | 0.184 (0.111 - 0.29) | 0.158 (0.085-0.232) | 59 (40 - 88) | 5.263 (3.555 - 7.764) | 134 (77 - 225) | 5.649 (3.297 - 9.345) | 0.269 (0.188-0.35) |
| Togo | Female | 0 (0 - 1) | 0.057 (0.037 - 0.076) | 1 (1 - 2) | 0.055 (0.035 - 0.08) | -0.225 (-0.366to-0.083) | 0 (0 - 0) | 0.055 (0.036 - 0.074) | 1 (1 - 1) | 0.052 (0.033 - 0.075) | -0.236 (-0.341to-0.132) | 11 (7 - 15) | 1.408 (0.892 - 1.882) | 31 (19 - 44) | 1.237 (0.783 - 1.788) | -0.573 (-0.704to-0.442) |
|  | Male | 1 (1 - 2) | 0.192 (0.13 - 0.28) | 6 (4 - 10) | 0.273 (0.172 - 0.44) | 0.939 (0.785-1.094) | 1 (1 - 2) | 0.18 (0.124 - 0.265) | 5 (3 - 8) | 0.242 (0.154 - 0.39) | 0.78 (0.605-0.956) | 38 (26 - 57) | 5.433 (3.687 - 8.03) | 176 (109 - 287) | 7.475 (4.669 - 12.272) | 0.857 (0.674-1.04) |
