# Supplementary material for: Incidence, mortality, and DALYs of global pharyngeal cancer: systematic analysis and projections Based on global burden of disease study 2021
Source: Ann Med. 2025 Aug 19;57(1):2547092. doi: 10.1080/07853890.2025.2547092 (PMC12366512; doi:10.1080/07853890.2025.2547092)
Supplement: 3_Supplementary Figures_PC_AM_Revision.docx [file IANN_A_2547092_SM9504.docx]

**Supplementary Figure 1. Pharyngeal cancer burden across different age groups in 2021.**

**
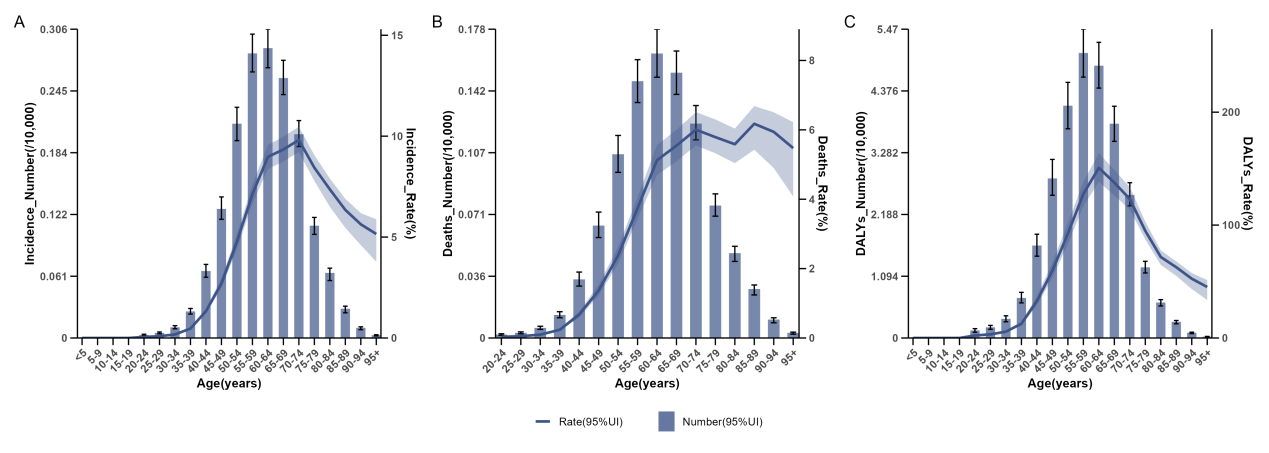
**

**Supplementary Figure 2. Trends of pharyngeal cancer burden across different age groups in global and 7 GBD super-regions from 1990 to 2021.**

**
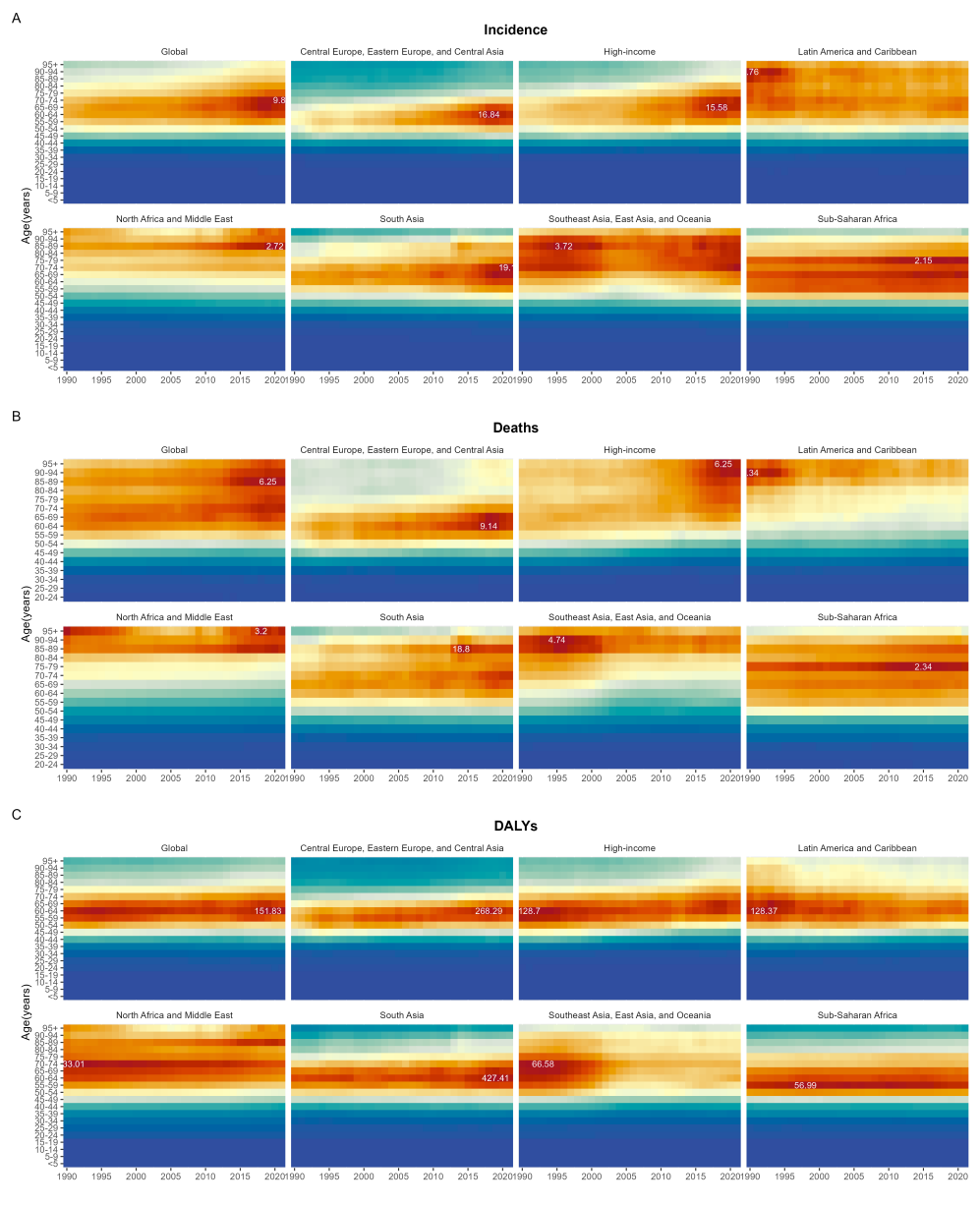
**

**Supplementary Figure 3. Proportion of deaths attributable to risk factors for pharyngeal cancer in females and males by age groups from 1990 to 2021.**

**
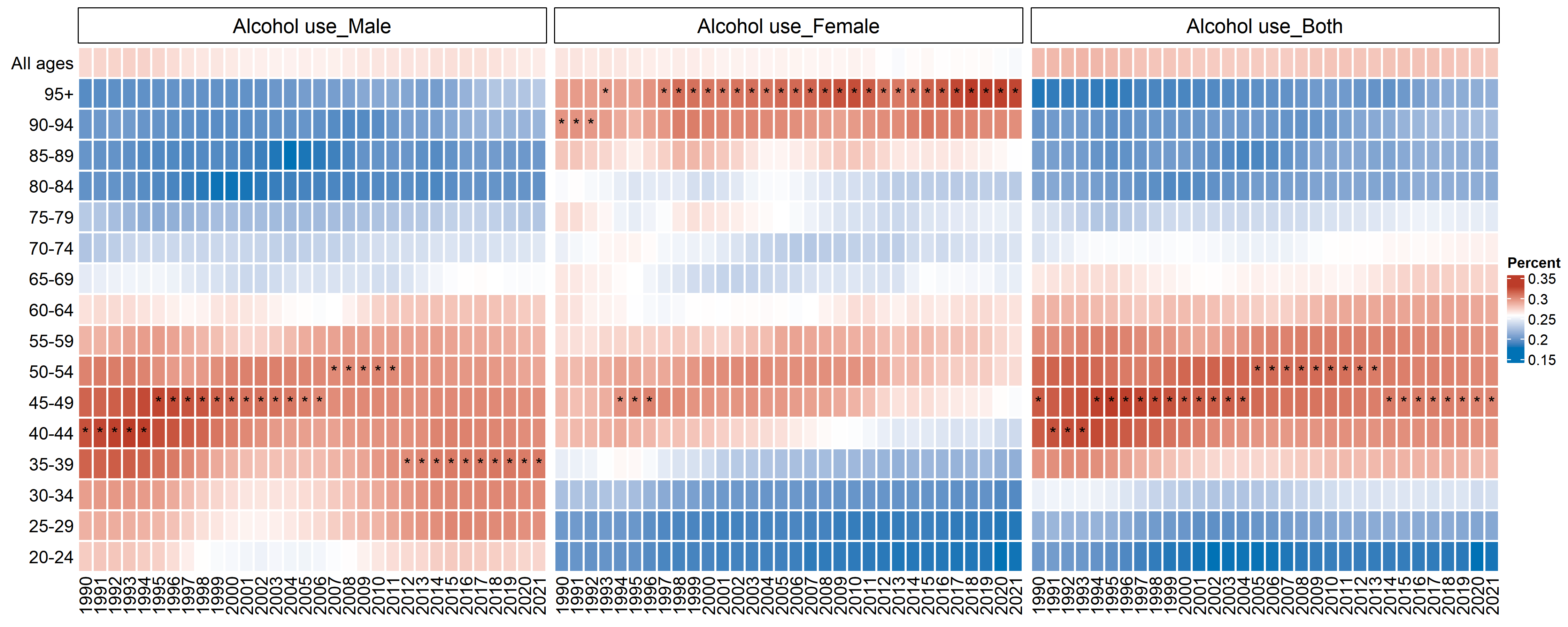
**

**
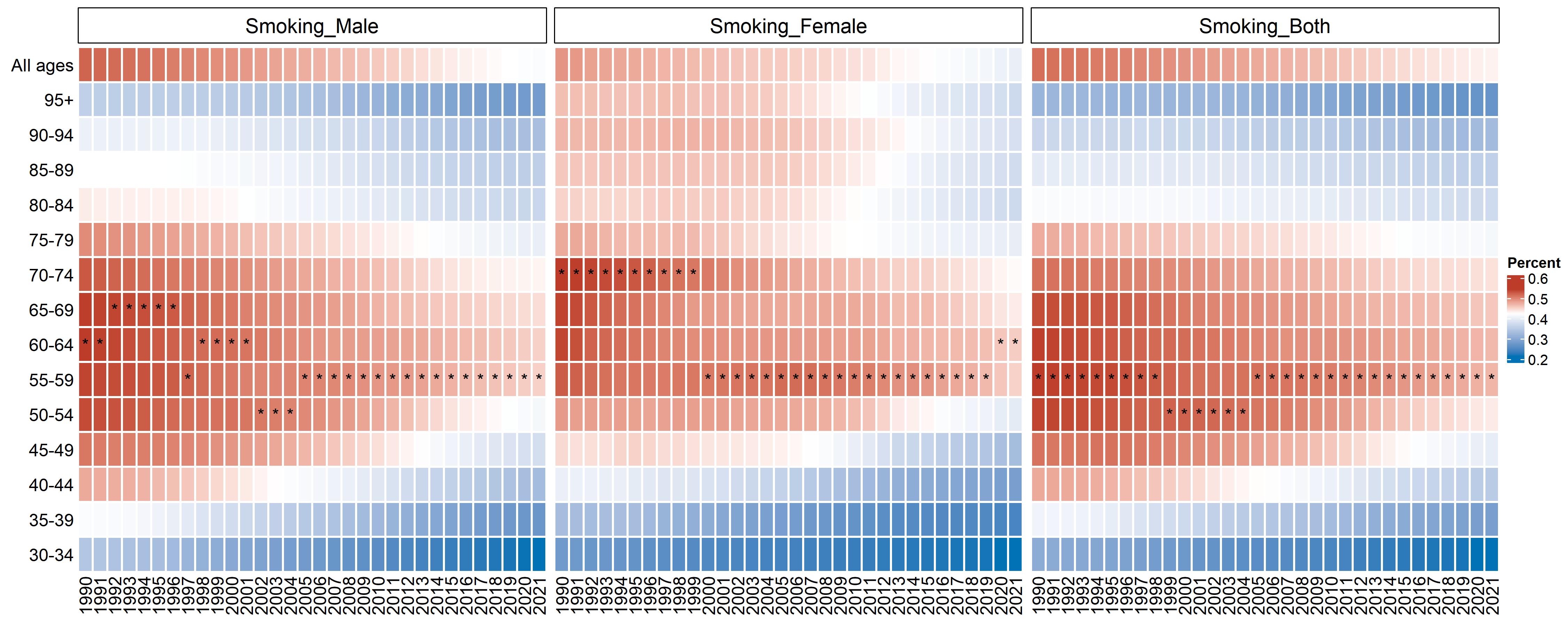
**

**Supplementary Figure 4.Projection of global pharyngeal cancer burden using BAPC and Nordpred methods (2022–2050)**

**
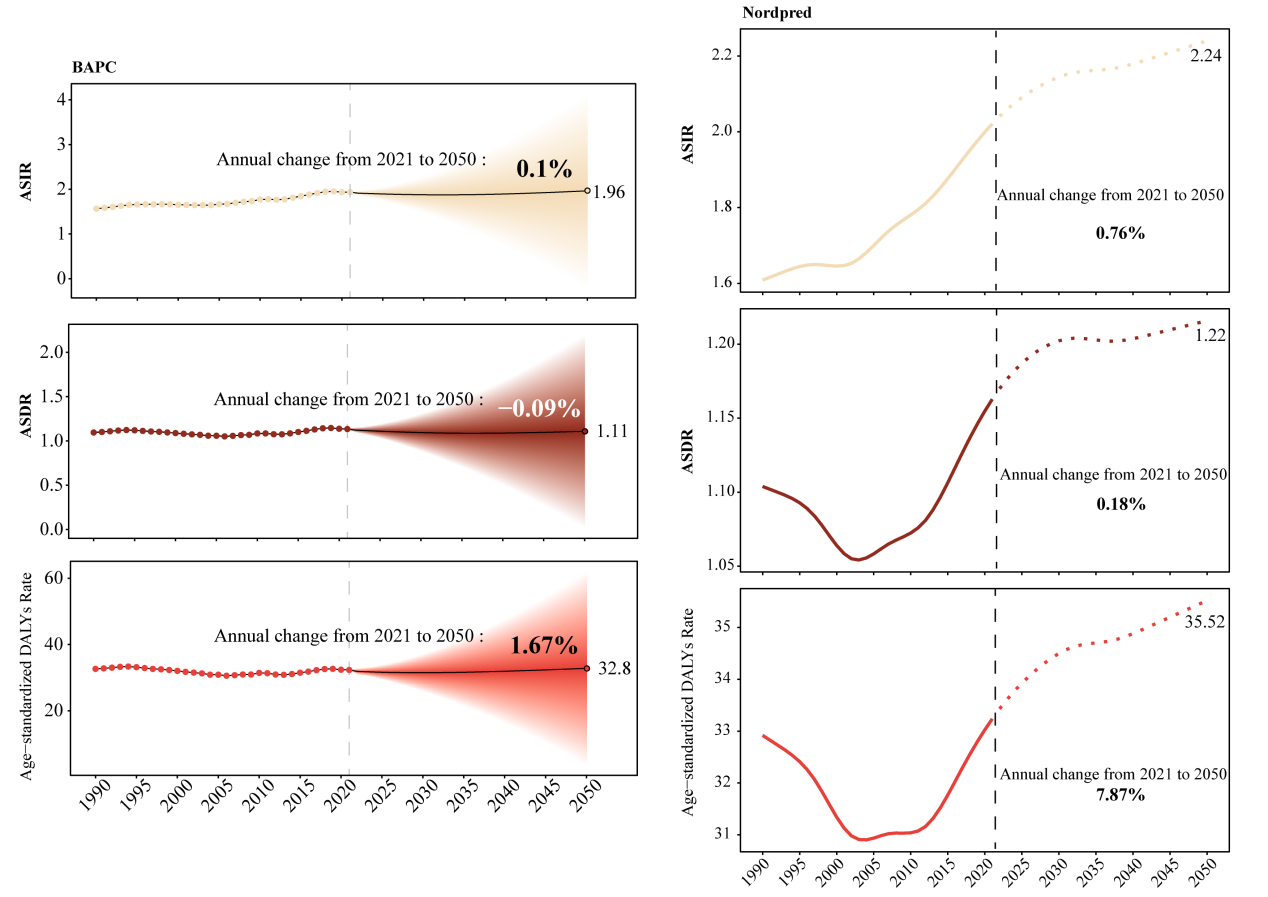
**

**Supplementary Figure 5. ASDR and deaths of PC by gender across SDI regions, 1990-2021**

**
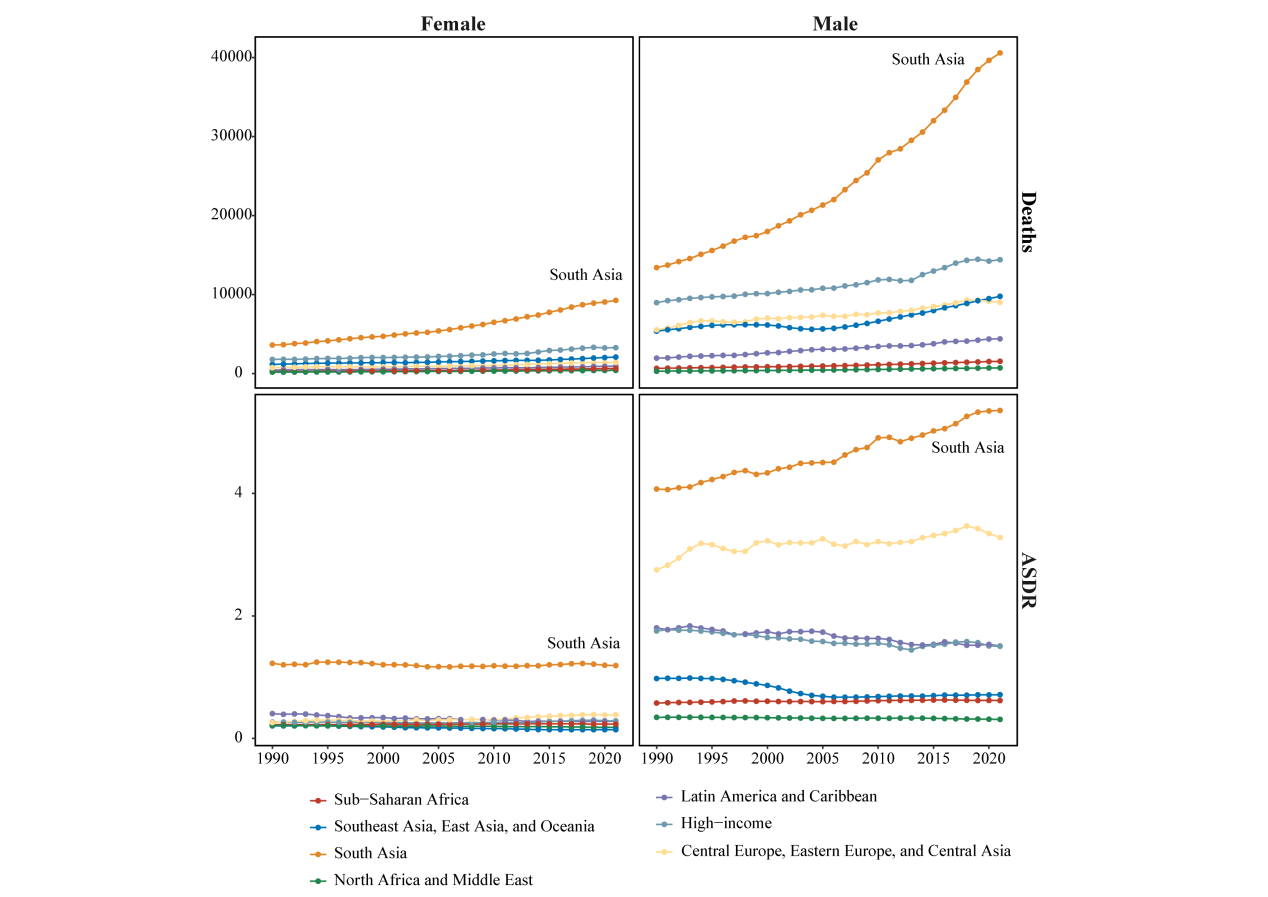
**

**Supplementary Table 1. Global burden and trends of pharyngeal cancer from 1990 to 2021 in 204 countries and territories**

**Supplementary Table 2. Global burden and trends of pharyngeal cancer from 1990 to 2021 in 204 countries and territories by gender**

**Supplementary Table 3. Comparative global burden of pharyngeal cancer across pre-COVID-19 (2019) and post-COVID-19 (2021) periods in GBD 2021**

**Supplementary Table 4.Projection of global pharyngeal cancer burden using BAPC and Nordpred methods (2022–2050)**

**Supplementary Table 5. Risk factors associated with mortality of pharyngeal cancer by SDI regions and gender**
