# Supplementary material for: Incidence, mortality, and DALYs of global pharyngeal cancer: systematic analysis and projections Based on global burden of disease study 2021
Source: Ann Med. 2025 Aug 19;57(1):2547092. doi: 10.1080/07853890.2025.2547092 (PMC12366512; doi:10.1080/07853890.2025.2547092)
Supplement: Supplementary Table 1.docx [file IANN_A_2547092_SM9503.docx]

| Supplementary Table 1 Global burden and trends of pharyngeal cancer from 1990 to 2021 in 204 countries and territories | | | | | | | | | | | | | | | |
| --- | --- | --- | --- | --- | --- | --- | --- | --- | --- | --- | --- | --- | --- | --- | --- |
| **Characteristics** | **1990** | | **2021** | | **1990-2021** | **1990** | | **2021** | | **1990-2021** | **1990** | | **2021** | | **1990-2021** |
|  | **Incidence cases** | **ASIR** | **Incidence cases** | **ASIR** | **EAPC** | **Death cases** | **ASDR** | **Death cases** | **ASDR** | **EAPC** | **DALYs cases** | **Age_standardised  DALYs Rate** | **DALYs cases** | **Age_standardised  DALYs** | **EAPC** |
|  | **(95%UI)** | **per 100,000 (95%UI)** | **(95%UI)** | **per 100,000 (95%UI)** | **(95%CI)** | **(95%UI)** | **per 100,000 (95%UI)** | **(95%UI)** | **per 100,000 (95%UI)** | **(95%CI)** | **(95%UI)** | **per 100,000 (95%UI)** | **(95%UI)** | **per 100,000 (95%UI)** | **(95%CI)** |
| Afghanistan | 26 (14 - 52) | 0.374 (0.203 - 0.712) | 37 (21 - 72) | 0.338 (0.203 - 0.601) | -0.357 (-0.452to-0.263) | 25 (13 - 49) | 0.371 (0.206 - 0.682) | 32 (18 - 60) | 0.318 (0.193 - 0.543) | -0.511 (-0.572to-0.449) | 735 (369 - 1539) | 10.068 (5.163 - 20.628) | 1068 (587 - 2152) | 8.403 (4.836 - 15.596) | -0.629 (-0.693to-0.565) |
| Albania | 20 (16 - 26) | 0.906 (0.703 - 1.183) | 50 (36 - 71) | 1.21 (0.867 - 1.725) | 1.425 (1.237-1.612) | 16 (13 - 21) | 0.754 (0.583 - 0.99) | 30 (21 - 42) | 0.706 (0.504 - 1.004) | 0.105 (-0.03-0.239) | 499 (382 - 655) | 21.479 (16.484 - 28.191) | 816 (580 - 1180) | 20.343 (14.45 - 29.323) | 0.162 (0.016-0.307) |
| Algeria | 96 (76 - 121) | 0.771 (0.619 - 0.957) | 328 (240 - 450) | 0.867 (0.64 - 1.175) | 0.599 (0.509-0.689) | 81 (64 - 101) | 0.685 (0.551 - 0.843) | 213 (156 - 291) | 0.601 (0.445 - 0.806) | -0.146 (-0.242to-0.05) | 2425 (1919 - 3002) | 18.2 (14.464 - 22.631) | 6212 (4531 - 8540) | 15.763 (11.48 - 21.591) | -0.287 (-0.36to-0.213) |
| American Samoa | 0 (0 - 0) | 0.16 (0.121 - 0.204) | 0 (0 - 0) | 0.297 (0.216 - 0.397) | 3.2 (2.577-3.827) | 0 (0 - 0) | 0.139 (0.105 - 0.178) | 0 (0 - 0) | 0.248 (0.18 - 0.331) | 3.095 (2.461-3.732) | 1 (1 - 1) | 4.002 (3.047 - 5.097) | 4 (3 - 5) | 7.333 (5.289 - 9.749) | 3.197 (2.557-3.84) |
| Andorra | 1 (1 - 2) | 1.784 (1.153 - 2.677) | 2 (2 - 4) | 1.583 (0.997 - 2.331) | -0.043 (-0.294-0.209) | 0 (0 - 1) | 0.775 (0.497 - 1.156) | 1 (0 - 1) | 0.47 (0.295 - 0.695) | -1.256 (-1.451to-1.061) | 14 (9 - 21) | 23.639 (15.383 - 35.553) | 22 (13 - 32) | 14.324 (8.917 - 21.17) | -1.245 (-1.426to-1.064) |
| Angola | 15 (10 - 21) | 0.347 (0.243 - 0.48) | 54 (39 - 74) | 0.4 (0.3 - 0.541) | 0.703 (0.538-0.868) | 14 (10 - 20) | 0.342 (0.243 - 0.471) | 47 (35 - 66) | 0.372 (0.277 - 0.51) | 0.525 (0.381-0.669) | 465 (308 - 668) | 9.734 (6.636 - 13.865) | 1539 (1105 - 2139) | 10.407 (7.585 - 14.473) | 0.476 (0.321-0.631) |
| Antigua and Barbuda | 1 (1 - 1) | 1.917 (1.755 - 2.073) | 2 (1 - 2) | 1.376 (1.218 - 1.509) | -0.957 (-1.545to-0.365) | 1 (1 - 1) | 1.584 (1.456 - 1.716) | 1 (1 - 1) | 1.017 (0.898 - 1.117) | -1.295 (-1.887to-0.7) | 21 (19 - 22) | 41.551 (38.001 - 45.218) | 29 (25 - 32) | 25.221 (22.399 - 27.84) | -1.456 (-2.042to-0.867) |
| Argentina | 404 (352 - 460) | 1.246 (1.085 - 1.42) | 332 (275 - 396) | 0.617 (0.511 - 0.736) | -1.851 (-2.269to-1.432) | 315 (277 - 357) | 0.975 (0.858 - 1.107) | 217 (181 - 257) | 0.396 (0.329 - 0.472) | -2.39 (-2.827to-1.951) | 9147 (7993 - 10438) | 28.155 (24.547 - 32.15) | 5778 (4803 - 6936) | 10.844 (8.992 - 13.068) | -2.618 (-3.041to-2.192) |
| Armenia | 13 (11 - 17) | 0.452 (0.369 - 0.564) | 23 (19 - 27) | 0.526 (0.434 - 0.624) | 0.807 (0.448-1.168) | 11 (9 - 13) | 0.373 (0.3 - 0.464) | 17 (14 - 20) | 0.379 (0.312 - 0.448) | 0.293 (-0.062-0.649) | 326 (264 - 407) | 10.654 (8.622 - 13.279) | 436 (358 - 517) | 10.112 (8.28 - 12.013) | 0.096 (-0.277-0.47) |
| Australia | 589 (532 - 648) | 3.089 (2.803 - 3.394) | 1602 (1369 - 1848) | 3.973 (3.394 - 4.584) | 0.778 (0.424-1.134) | 170 (155 - 187) | 0.88 (0.801 - 0.967) | 318 (270 - 367) | 0.735 (0.629 - 0.845) | -0.706 (-1.16to-0.251) | 4743 (4315 - 5220) | 25.084 (22.773 - 27.628) | 8317 (7146 - 9564) | 20.821 (17.969 - 23.855) | -0.666 (-1.103to-0.228) |
| Austria | 251 (229 - 275) | 2.464 (2.242 - 2.707) | 600 (519 - 688) | 3.837 (3.327 - 4.391) | 1.641 (1.422-1.86) | 132 (121 - 144) | 1.248 (1.14 - 1.367) | 218 (187 - 248) | 1.317 (1.138 - 1.496) | 0.39 (0.268-0.512) | 3962 (3616 - 4339) | 39.61 (35.946 - 43.439) | 5752 (4985 - 6555) | 37.335 (32.418 - 42.537) | -0.004 (-0.138-0.13) |
| Azerbaijan | 21 (14 - 30) | 0.388 (0.258 - 0.567) | 41 (25 - 64) | 0.362 (0.222 - 0.56) | 0.162 (-0.084-0.409) | 18 (12 - 26) | 0.343 (0.231 - 0.501) | 31 (19 - 47) | 0.285 (0.179 - 0.431) | -0.219 (-0.432to-0.006) | 550 (354 - 797) | 9.844 (6.4 - 14.428) | 928 (571 - 1438) | 7.846 (4.851 - 12.064) | -0.437 (-0.641to-0.232) |
| Bahamas | 5 (4 - 5) | 2.916 (2.655 - 3.201) | 9 (7 - 11) | 2.039 (1.631 - 2.527) | -0.844 (-1.278to-0.407) | 4 (3 - 4) | 2.425 (2.206 - 2.654) | 7 (5 - 8) | 1.576 (1.277 - 1.946) | -1.126 (-1.588to-0.661) | 117 (106 - 129) | 69.405 (62.963 - 76.147) | 194 (154 - 243) | 43.307 (34.683 - 53.831) | -1.256 (-1.73to-0.779) |
| Bahrain | 1 (1 - 1) | 0.562 (0.474 - 0.689) | 6 (4 - 8) | 0.57 (0.413 - 0.804) | 0.199 (-0.146-0.545) | 1 (1 - 1) | 0.491 (0.414 - 0.605) | 3 (2 - 4) | 0.357 (0.262 - 0.504) | -0.96 (-1.352to-0.566) | 25 (21 - 31) | 11.598 (9.797 - 14.288) | 89 (62 - 132) | 8.01 (5.76 - 11.556) | -1.18 (-1.533to-0.825) |
| Bangladesh | 1744 (1226 - 2365) | 3.501 (2.468 - 4.758) | 5041 (3209 - 7943) | 3.492 (2.219 - 5.455) | -0.02 (-0.105-0.064) | 1631 (1149 - 2217) | 3.341 (2.327 - 4.551) | 4039 (2542 - 6326) | 2.856 (1.804 - 4.426) | -0.526 (-0.637to-0.415) | 50839 (35687 - 68556) | 97.303 (68.243 - 131.468) | 118310 (74487 - 190043) | 80.112 (50.475 - 127.717) | -0.622 (-0.709to-0.536) |
| Barbados | 6 (6 - 7) | 2.321 (2.104 - 2.547) | 8 (6 - 11) | 1.664 (1.272 - 2.135) | -1.038 (-1.349to-0.726) | 5 (5 - 6) | 1.887 (1.721 - 2.062) | 6 (5 - 8) | 1.207 (0.915 - 1.538) | -1.415 (-1.737to-1.092) | 128 (116 - 140) | 49.679 (44.973 - 54.606) | 150 (113 - 195) | 30.223 (22.611 - 39.049) | -1.515 (-1.833to-1.196) |
| Belarus | 173 (145 - 205) | 1.332 (1.125 - 1.572) | 789 (608 - 1008) | 5.148 (3.977 - 6.561) | 4.055 (3.619-4.493) | 133 (112 - 158) | 1.018 (0.86 - 1.198) | 439 (340 - 567) | 2.794 (2.169 - 3.6) | 2.826 (2.335-3.319) | 4220 (3556 - 5000) | 32.742 (27.648 - 38.521) | 13388 (10359 - 17281) | 88.329 (68.316 - 113.916) | 2.677 (2.172-3.184) |
| Belgium | 293 (255 - 341) | 2.16 (1.864 - 2.517) | 617 (504 - 749) | 3.214 (2.617 - 3.914) | 1.185 (0.901-1.471) | 142 (123 - 163) | 1.003 (0.869 - 1.161) | 210 (174 - 251) | 1.016 (0.839 - 1.221) | -0.013 (-0.235-0.21) | 4135 (3584 - 4799) | 31 (26.846 - 36.146) | 5655 (4643 - 6818) | 29.879 (24.487 - 36.102) | -0.263 (-0.513to-0.012) |
| Belize | 1 (1 - 1) | 0.772 (0.715 - 0.83) | 2 (2 - 2) | 0.664 (0.574 - 0.772) | -0.351 (-0.509to-0.194) | 1 (1 - 1) | 0.681 (0.631 - 0.732) | 2 (1 - 2) | 0.537 (0.463 - 0.62) | -0.664 (-0.828to-0.499) | 17 (16 - 18) | 17.955 (16.652 - 19.332) | 48 (41 - 55) | 14.297 (12.259 - 16.615) | -0.607 (-0.769to-0.445) |
| Benin | 2 (2 - 3) | 0.117 (0.089 - 0.154) | 7 (5 - 10) | 0.126 (0.088 - 0.176) | 0.097 (-0.028-0.222) | 2 (2 - 3) | 0.113 (0.088 - 0.148) | 7 (4 - 9) | 0.116 (0.081 - 0.162) | -0.038 (-0.159-0.083) | 70 (52 - 93) | 3.244 (2.454 - 4.335) | 212 (143 - 306) | 3.354 (2.283 - 4.795) | -0.036 (-0.167-0.095) |
| Bermuda | 2 (2 - 2) | 3.436 (3.063 - 3.848) | 3 (3 - 4) | 2.438 (1.997 - 3.026) | -0.669 (-1.25to-0.086) | 2 (1 - 2) | 2.504 (2.245 - 2.813) | 2 (1 - 2) | 1.156 (0.946 - 1.432) | -2.071 (-2.724to-1.414) | 42 (37 - 47) | 65.63 (58.413 - 73.898) | 37 (30 - 45) | 29.54 (24.09 - 36.64) | -2.164 (-2.834to-1.489) |
| Bhutan | 8 (5 - 11) | 2.761 (1.773 - 4.057) | 21 (15 - 28) | 3.263 (2.321 - 4.456) | 0.559 (0.481-0.636) | 7 (4 - 10) | 2.63 (1.681 - 3.85) | 17 (12 - 23) | 2.731 (1.953 - 3.739) | 0.146 (0.106-0.186) | 227 (141 - 341) | 76.851 (48.963 - 115.494) | 492 (345 - 698) | 75.887 (53.539 - 107.134) | -0.04 (-0.085-0.004) |
| Bolivia  (Plurinational State of) | 15 (11 - 21) | 0.451 (0.32 - 0.617) | 35 (25 - 48) | 0.376 (0.272 - 0.516) | -0.621 (-0.751to-0.491) | 14 (10 - 20) | 0.444 (0.315 - 0.602) | 30 (22 - 41) | 0.333 (0.241 - 0.455) | -0.941 (-1.057to-0.825) | 421 (298 - 582) | 11.866 (8.409 - 16.378) | 836 (600 - 1170) | 8.552 (6.175 - 11.806) | -1.126 (-1.253to-0.999) |
| Bosnia and Herzegovina | 58 (50 - 67) | 1.226 (1.068 - 1.434) | 126 (96 - 160) | 2.185 (1.672 - 2.783) | 2.544 (2.229-2.86) | 43 (37 - 51) | 0.936 (0.813 - 1.091) | 77 (59 - 98) | 1.293 (0.988 - 1.64) | 1.524 (1.288-1.761) | 1396 (1195 - 1647) | 29.031 (25.076 - 33.96) | 2169 (1655 - 2760) | 38.432 (29.353 - 49.493) | 1.396 (1.146-1.645) |
| Botswana | 5 (3 - 6) | 0.745 (0.522 - 1.008) | 13 (8 - 20) | 0.776 (0.492 - 1.179) | -0.171 (-0.438-0.097) | 4 (3 - 6) | 0.688 (0.489 - 0.926) | 11 (7 - 17) | 0.673 (0.43 - 1.006) | -0.368 (-0.682to-0.053) | 132 (89 - 185) | 20.185 (13.917 - 27.919) | 348 (208 - 557) | 19.506 (12.03 - 30.362) | -0.462 (-0.799to-0.123) |
| Brazil | 1887 (1790 - 1985) | 1.977 (1.869 - 2.083) | 5209 (4835 - 5542) | 2.017 (1.872 - 2.147) | -0.038 (-0.179-0.104) | 1592 (1509 - 1678) | 1.722 (1.623 - 1.815) | 3768 (3485 - 4013) | 1.471 (1.359 - 1.566) | -0.565 (-0.705to-0.425) | 49385 (46853 - 51976) | 49.465 (46.954 - 52.125) | 109421 (101909 - 116656) | 42.115 (39.243 - 44.89) | -0.633 (-0.799to-0.466) |
| Brunei Darussalam | 2 (1 - 2) | 1.575 (1.188 - 2.026) | 7 (5 - 8) | 1.709 (1.366 - 2.079) | 0.968 (0.743-1.194) | 1 (1 - 2) | 1.304 (0.981 - 1.7) | 4 (3 - 5) | 1.179 (0.951 - 1.441) | 0.443 (0.193-0.694) | 38 (29 - 49) | 33.626 (25.29 - 43.309) | 125 (103 - 152) | 30.604 (25.052 - 36.999) | 0.399 (0.146-0.652) |
| Bulgaria | 176 (157 - 197) | 1.455 (1.295 - 1.624) | 356 (297 - 419) | 3.053 (2.54 - 3.586) | 2.361 (2.168-2.555) | 103 (92 - 114) | 0.843 (0.754 - 0.936) | 172 (145 - 199) | 1.393 (1.175 - 1.614) | 1.66 (1.47-1.851) | 3226 (2879 - 3574) | 26.869 (23.993 - 29.795) | 5025 (4231 - 5844) | 43.949 (36.93 - 51.279) | 1.577 (1.372-1.782) |
| Burkina Faso | 6 (4 - 8) | 0.121 (0.09 - 0.166) | 15 (10 - 21) | 0.143 (0.103 - 0.208) | 0.45 (0.312-0.589) | 5 (4 - 7) | 0.117 (0.087 - 0.159) | 13 (9 - 19) | 0.134 (0.095 - 0.192) | 0.358 (0.221-0.495) | 162 (116 - 226) | 3.343 (2.428 - 4.66) | 429 (291 - 634) | 3.882 (2.687 - 5.692) | 0.398 (0.244-0.552) |
| Burundi | 23 (15 - 32) | 0.918 (0.622 - 1.294) | 38 (24 - 57) | 0.671 (0.431 - 1) | -1.315 (-1.512to-1.118) | 22 (15 - 31) | 0.885 (0.605 - 1.268) | 35 (22 - 53) | 0.632 (0.403 - 0.956) | -1.389 (-1.577to-1.201) | 694 (466 - 999) | 26.66 (17.905 - 38.482) | 1143 (705 - 1767) | 18.543 (11.637 - 28.252) | -1.495 (-1.691to-1.299) |
| Cabo Verde | 0 (0 - 0) | 0.029 (0.021 - 0.052) | 3 (2 - 4) | 0.547 (0.38 - 0.752) | 8.189 (6.18-10.237) | 0 (0 - 0) | 0.026 (0.019 - 0.047) | 2 (1 - 3) | 0.414 (0.289 - 0.568) | 7.498 (5.457-9.579) | 2 (1 - 3) | 0.728 (0.527 - 1.268) | 61 (43 - 85) | 12.034 (8.571 - 16.751) | 7.74 (5.668-9.853) |
| Cambodia | 28 (21 - 38) | 0.586 (0.444 - 0.784) | 83 (60 - 112) | 0.643 (0.468 - 0.87) | 0.318 (0.124-0.513) | 26 (20 - 35) | 0.58 (0.439 - 0.774) | 72 (52 - 98) | 0.581 (0.429 - 0.782) | 0.015 (-0.15-0.181) | 791 (593 - 1086) | 15.601 (11.813 - 21.091) | 2064 (1479 - 2855) | 15.075 (10.906 - 20.659) | -0.126 (-0.299-0.047) |
| Cameroon | 7 (5 - 9) | 0.144 (0.104 - 0.19) | 24 (15 - 37) | 0.164 (0.102 - 0.242) | 0.277 (0.19-0.364) | 6 (5 - 9) | 0.136 (0.099 - 0.177) | 21 (13 - 32) | 0.147 (0.091 - 0.216) | 0.131 (0.025-0.238) | 211 (147 - 280) | 3.975 (2.839 - 5.259) | 702 (416 - 1088) | 4.379 (2.622 - 6.714) | 0.181 (0.062-0.3) |
| Canada | 547 (488 - 610) | 1.747 (1.558 - 1.951) | 1508 (1308 - 1723) | 2.276 (1.986 - 2.585) | 1.15 (0.96-1.341) | 207 (185 - 228) | 0.65 (0.584 - 0.716) | 451 (394 - 512) | 0.638 (0.557 - 0.725) | 0.172 (-0.085-0.429) | 5719 (5102 - 6323) | 18.353 (16.426 - 20.33) | 11209 (9764 - 12777) | 17.094 (14.909 - 19.459) | 0.009 (-0.259-0.277) |
| Central African Republic | 5 (3 - 6) | 0.359 (0.24 - 0.494) | 8 (6 - 12) | 0.326 (0.231 - 0.454) | -0.3 (-0.338to-0.263) | 4 (3 - 6) | 0.355 (0.242 - 0.479) | 8 (5 - 11) | 0.321 (0.232 - 0.437) | -0.308 (-0.342to-0.275) | 142 (88 - 200) | 10.225 (6.538 - 14.159) | 265 (179 - 378) | 9.194 (6.399 - 12.947) | -0.347 (-0.382to-0.311) |
| Chad | 3 (2 - 4) | 0.093 (0.069 - 0.121) | 9 (6 - 14) | 0.143 (0.097 - 0.203) | 1.422 (1.293-1.552) | 3 (2 - 3) | 0.09 (0.067 - 0.117) | 9 (6 - 13) | 0.136 (0.094 - 0.193) | 1.341 (1.196-1.486) | 78 (57 - 104) | 2.576 (1.895 - 3.419) | 285 (190 - 425) | 3.989 (2.689 - 5.82) | 1.443 (1.288-1.598) |
| Chile | 96 (84 - 110) | 0.929 (0.816 - 1.062) | 151 (129 - 175) | 0.595 (0.51 - 0.689) | -0.984 (-1.368to-0.598) | 72 (63 - 82) | 0.707 (0.627 - 0.805) | 77 (66 - 88) | 0.299 (0.258 - 0.342) | -2.352 (-2.724to-1.978) | 2093 (1829 - 2406) | 19.816 (17.316 - 22.766) | 1981 (1693 - 2292) | 7.87 (6.719 - 9.109) | -2.55 (-2.935to-2.163) |
| China | 5074 (4142 - 6175) | 0.58 (0.477 - 0.702) | 12063 (9529 - 15280) | 0.558 (0.442 - 0.703) | -0.477 (-0.925to-0.027) | 4320 (3533 - 5249) | 0.513 (0.425 - 0.621) | 5881 (4692 - 7407) | 0.278 (0.222 - 0.347) | -2.519 (-2.91to-2.127) | 129153 (104815 - 158416) | 13.965 (11.399 - 17.065) | 155670 (122186 - 199433) | 7.207 (5.695 - 9.158) | -2.682 (-3.096to-2.267) |
| Colombia | 94 (83 - 104) | 0.52 (0.464 - 0.577) | 224 (185 - 272) | 0.405 (0.335 - 0.493) | -1.345 (-1.714to-0.974) | 81 (72 - 89) | 0.467 (0.417 - 0.515) | 154 (127 - 186) | 0.278 (0.23 - 0.337) | -2.182 (-2.533to-1.829) | 2276 (2031 - 2535) | 11.803 (10.513 - 13.125) | 3736 (3041 - 4577) | 6.754 (5.511 - 8.27) | -2.314 (-2.669to-1.958) |
| Comoros | 2 (1 - 2) | 0.774 (0.516 - 1.082) | 4 (3 - 6) | 0.731 (0.468 - 1.119) | -0.369 (-0.515to-0.222) | 2 (1 - 2) | 0.732 (0.492 - 1.029) | 3 (2 - 5) | 0.66 (0.423 - 1.03) | -0.518 (-0.656to-0.38) | 51 (33 - 73) | 21.812 (14.161 - 30.806) | 110 (71 - 173) | 19.3 (12.395 - 30.475) | -0.616 (-0.789to-0.443) |
| Congo | 5 (3 - 7) | 0.427 (0.28 - 0.589) | 13 (9 - 18) | 0.421 (0.298 - 0.589) | -0.075 (-0.258-0.109) | 5 (3 - 6) | 0.413 (0.276 - 0.562) | 11 (8 - 16) | 0.384 (0.271 - 0.539) | -0.27 (-0.425to-0.116) | 145 (91 - 208) | 11.846 (7.531 - 16.756) | 367 (252 - 509) | 10.753 (7.518 - 15.097) | -0.367 (-0.532to-0.201) |
| Cook Islands | 0 (0 - 0) | 0.067 (0.054 - 0.084) | 0 (0 - 0) | 0.123 (0.09 - 0.161) | 2.643 (2.208-3.08) | 0 (0 - 0) | 0.055 (0.045 - 0.069) | 0 (0 - 0) | 0.079 (0.057 - 0.105) | 1.801 (1.363-2.241) | 0 (0 - 0) | 1.477 (1.191 - 1.85) | 1 (0 - 1) | 2.214 (1.598 - 2.893) | 2.094 (1.595-2.595) |
| Costa Rica | 16 (14 - 18) | 0.895 (0.798 - 0.989) | 47 (40 - 54) | 0.844 (0.726 - 0.975) | -0.602 (-0.862to-0.341) | 12 (11 - 13) | 0.677 (0.605 - 0.749) | 30 (26 - 34) | 0.538 (0.467 - 0.621) | -1.068 (-1.29to-0.846) | 323 (288 - 357) | 17.724 (15.768 - 19.616) | 742 (641 - 856) | 13.409 (11.57 - 15.483) | -1.379 (-1.636to-1.121) |
| Côte d'Ivoire | 10 (8 - 14) | 0.22 (0.168 - 0.291) | 30 (18 - 52) | 0.226 (0.142 - 0.377) | -0.237 (-0.374to-0.1) | 9 (7 - 13) | 0.208 (0.159 - 0.271) | 26 (16 - 44) | 0.201 (0.13 - 0.337) | -0.41 (-0.551to-0.269) | 317 (234 - 437) | 6.163 (4.616 - 8.286) | 874 (520 - 1523) | 6.007 (3.639 - 10.354) | -0.398 (-0.549to-0.248) |
| Croatia | 212 (187 - 237) | 3.288 (2.932 - 3.669) | 307 (258 - 360) | 4.044 (3.373 - 4.708) | 0.89 (0.566-1.215) | 144 (127 - 161) | 2.236 (1.995 - 2.485) | 164 (138 - 193) | 2.043 (1.719 - 2.412) | -0.127 (-0.427-0.174) | 4604 (4058 - 5149) | 70.991 (63.083 - 79.206) | 4421 (3708 - 5174) | 60.227 (49.895 - 70.652) | -0.374 (-0.695to-0.051) |
| Cuba | 203 (185 - 221) | 1.991 (1.816 - 2.177) | 380 (316 - 452) | 1.963 (1.635 - 2.333) | 0.196 (-0.383-0.778) | 153 (141 - 167) | 1.519 (1.387 - 1.658) | 237 (196 - 281) | 1.2 (0.992 - 1.428) | -0.47 (-1.073-0.137) | 3910 (3566 - 4250) | 38.258 (34.883 - 41.679) | 6042 (5022 - 7243) | 31.587 (26.319 - 37.766) | -0.358 (-0.937-0.225) |
| Cyprus | 3 (2 - 3) | 0.355 (0.284 - 0.429) | 11 (8 - 15) | 0.57 (0.426 - 0.748) | 2.48 (2.141-2.819) | 2 (1 - 2) | 0.23 (0.182 - 0.278) | 4 (3 - 5) | 0.186 (0.139 - 0.243) | -0.23 (-0.375to-0.085) | 46 (37 - 57) | 5.838 (4.676 - 7.168) | 101 (74 - 134) | 5.212 (3.841 - 6.936) | 0.216 (0.042-0.39) |
| Czechia | 243 (212 - 275) | 1.855 (1.621 - 2.109) | 715 (578 - 885) | 3.86 (3.1 - 4.787) | 2.598 (2.432-2.765) | 171 (149 - 195) | 1.297 (1.127 - 1.476) | 340 (275 - 421) | 1.764 (1.421 - 2.185) | 1.23 (1.113-1.348) | 5234 (4550 - 5951) | 40.732 (35.426 - 46.283) | 9267 (7439 - 11518) | 51.837 (41.247 - 64.699) | 1.002 (0.864-1.141) |
| Democratic People's Republic of Korea | 67 (48 - 90) | 0.378 (0.278 - 0.506) | 114 (83 - 153) | 0.334 (0.244 - 0.446) | -0.288 (-0.4to-0.176) | 56 (40 - 75) | 0.331 (0.243 - 0.441) | 86 (62 - 117) | 0.256 (0.184 - 0.346) | -0.747 (-0.819to-0.676) | 1758 (1257 - 2407) | 9.396 (6.766 - 12.679) | 2497 (1757 - 3388) | 7.2 (5.131 - 9.72) | -0.823 (-0.894to-0.752) |
| Democratic Republic of the Congo | 48 (37 - 63) | 0.283 (0.215 - 0.366) | 120 (81 - 176) | 0.293 (0.197 - 0.424) | 0.204 (-0.055-0.464) | 45 (34 - 59) | 0.277 (0.211 - 0.36) | 107 (72 - 159) | 0.275 (0.184 - 0.403) | 0.091 (-0.14-0.322) | 1412 (1067 - 1852) | 7.697 (5.884 - 9.985) | 3421 (2289 - 5134) | 7.608 (5.081 - 11.289) | 0.078 (-0.157-0.314) |
| Denmark | 142 (129 - 155) | 2.008 (1.819 - 2.207) | 389 (334 - 446) | 3.823 (3.299 - 4.378) | 2.229 (1.858-2.602) | 72 (66 - 79) | 0.985 (0.895 - 1.078) | 139 (120 - 158) | 1.268 (1.091 - 1.447) | 0.683 (0.372-0.996) | 2014 (1825 - 2210) | 29.328 (26.433 - 32.31) | 3530 (3038 - 4034) | 35.288 (30.489 - 40.259) | 0.483 (0.141-0.827) |
| Djibouti | 1 (1 - 2) | 0.867 (0.593 - 1.306) | 7 (4 - 12) | 0.907 (0.535 - 1.556) | 0.055 (0.009-0.102) | 1 (1 - 2) | 0.813 (0.558 - 1.236) | 6 (4 - 11) | 0.814 (0.482 - 1.394) | -0.078 (-0.151to-0.004) | 44 (29 - 68) | 24.174 (16.246 - 37.45) | 201 (116 - 365) | 23.671 (13.939 - 41.906) | -0.157 (-0.247to-0.068) |
| Dominica | 1 (1 - 2) | 2.553 (2.141 - 2.972) | 2 (1 - 2) | 2.072 (1.577 - 2.686) | -0.488 (-0.794to-0.182) | 1 (1 - 2) | 2.31 (1.941 - 2.705) | 1 (1 - 2) | 1.756 (1.347 - 2.269) | -0.687 (-1.007to-0.367) | 33 (28 - 39) | 58.285 (49.711 - 67.654) | 40 (30 - 53) | 46.458 (34.946 - 60.857) | -0.48 (-0.794to-0.164) |
| Dominican Republic | 53 (44 - 64) | 1.429 (1.171 - 1.724) | 178 (130 - 243) | 1.757 (1.284 - 2.39) | 0.992 (0.89-1.094) | 48 (39 - 59) | 1.356 (1.104 - 1.633) | 148 (107 - 201) | 1.478 (1.072 - 2.007) | 0.654 (0.527-0.781) | 1381 (1137 - 1680) | 34.458 (28.207 - 41.877) | 4028 (2873 - 5561) | 38.962 (27.852 - 53.713) | 0.774 (0.634-0.914) |
| Ecuador | 23 (20 - 26) | 0.42 (0.371 - 0.475) | 51 (38 - 67) | 0.31 (0.234 - 0.404) | -0.961 (-1.603to-0.314) | 21 (18 - 24) | 0.395 (0.349 - 0.447) | 39 (30 - 51) | 0.241 (0.186 - 0.311) | -1.486 (-2.145to-0.823) | 595 (523 - 669) | 10.262 (9.025 - 11.578) | 1017 (767 - 1336) | 6.075 (4.592 - 7.971) | -1.665 (-2.32to-1.007) |
| Egypt | 52 (42 - 87) | 0.191 (0.152 - 0.321) | 187 (141 - 244) | 0.284 (0.219 - 0.364) | 1.657 (1.329-1.985) | 46 (37 - 76) | 0.182 (0.145 - 0.305) | 130 (98 - 169) | 0.219 (0.169 - 0.277) | 1.076 (0.722-1.433) | 1419 (1137 - 2266) | 4.578 (3.644 - 7.525) | 3937 (2957 - 5140) | 5.447 (4.157 - 7.042) | 0.969 (0.619-1.321) |
| El Salvador | 15 (13 - 17) | 0.491 (0.427 - 0.564) | 30 (24 - 39) | 0.497 (0.386 - 0.64) | -0.139 (-0.336-0.059) | 14 (12 - 16) | 0.46 (0.4 - 0.531) | 23 (18 - 29) | 0.366 (0.285 - 0.468) | -0.922 (-1.14to-0.704) | 372 (322 - 428) | 11.944 (10.334 - 13.808) | 583 (451 - 752) | 9.586 (7.386 - 12.382) | -0.895 (-1.133to-0.656) |
| Equatorial Guinea | 1 (0 - 1) | 0.34 (0.236 - 0.458) | 3 (2 - 4) | 0.452 (0.273 - 0.663) | 1.191 (1.057-1.324) | 1 (0 - 1) | 0.335 (0.236 - 0.447) | 2 (1 - 3) | 0.382 (0.23 - 0.562) | 0.648 (0.547-0.749) | 22 (14 - 30) | 9.609 (6.455 - 13.249) | 68 (39 - 105) | 10.623 (6.243 - 16.027) | 0.513 (0.394-0.633) |
| Eritrea | 12 (9 - 16) | 0.825 (0.599 - 1.064) | 28 (18 - 39) | 0.82 (0.543 - 1.138) | -0.246 (-0.378to-0.114) | 11 (8 - 15) | 0.788 (0.58 - 1.017) | 25 (16 - 35) | 0.761 (0.501 - 1.052) | -0.32 (-0.455to-0.184) | 391 (284 - 519) | 24.493 (17.981 - 32.11) | 860 (546 - 1228) | 22.994 (14.903 - 32.517) | -0.432 (-0.571to-0.293) |
| Estonia | 43 (37 - 50) | 2.113 (1.824 - 2.45) | 74 (61 - 89) | 3.29 (2.68 - 3.896) | 1.388 (1.093-1.684) | 33 (28 - 38) | 1.592 (1.38 - 1.84) | 40 (33 - 48) | 1.689 (1.382 - 2.004) | -0.055 (-0.296-0.187) | 1000 (868 - 1154) | 49.641 (43.031 - 57.297) | 1062 (867 - 1258) | 48.294 (39.316 - 57.413) | -0.409 (-0.653to-0.165) |
| Eswatini | 2 (2 - 3) | 0.737 (0.532 - 0.98) | 6 (4 - 9) | 0.99 (0.611 - 1.425) | 1.184 (0.803-1.566) | 2 (2 - 3) | 0.68 (0.49 - 0.904) | 6 (3 - 8) | 0.873 (0.547 - 1.25) | 1.071 (0.654-1.49) | 69 (49 - 92) | 19.964 (14.217 - 26.753) | 185 (110 - 270) | 26.696 (16.162 - 39.111) | 1.192 (0.739-1.646) |
| Ethiopia | 97 (66 - 140) | 0.436 (0.296 - 0.621) | 184 (124 - 254) | 0.383 (0.256 - 0.53) | -0.557 (-0.77to-0.344) | 92 (62 - 132) | 0.428 (0.291 - 0.605) | 159 (107 - 222) | 0.344 (0.228 - 0.485) | -0.832 (-1.014to-0.651) | 2987 (2008 - 4346) | 12.557 (8.453 - 17.998) | 5088 (3438 - 7010) | 9.819 (6.555 - 13.692) | -0.941 (-1.127to-0.755) |
| Fiji | 1 (1 - 1) | 0.207 (0.158 - 0.271) | 2 (2 - 3) | 0.271 (0.201 - 0.357) | 1.339 (1.039-1.64) | 1 (1 - 1) | 0.188 (0.144 - 0.246) | 2 (1 - 3) | 0.24 (0.179 - 0.314) | 1.252 (0.951-1.555) | 25 (19 - 32) | 5.363 (4.101 - 6.974) | 59 (43 - 79) | 6.765 (4.906 - 9.024) | 1.266 (0.953-1.581) |
| Finland | 68 (61 - 76) | 1.025 (0.917 - 1.14) | 150 (127 - 176) | 1.548 (1.311 - 1.834) | 1.772 (1.553-1.991) | 37 (33 - 41) | 0.542 (0.487 - 0.605) | 52 (44 - 60) | 0.484 (0.414 - 0.57) | -0.04 (-0.203-0.122) | 1057 (946 - 1181) | 16.148 (14.386 - 18.091) | 1360 (1161 - 1600) | 14.397 (12.242 - 17.117) | -0.062 (-0.217-0.094) |
| France | 4966 (4492 - 5484) | 7.025 (6.348 - 7.74) | 6330 (5399 - 7383) | 5.688 (4.859 - 6.603) | -0.699 (-0.911to-0.486) | 2443 (2224 - 2662) | 3.342 (3.045 - 3.641) | 1856 (1601 - 2149) | 1.527 (1.318 - 1.777) | -2.691 (-2.95to-2.43) | 74485 (67376 - 81617) | 106.934 (96.95 - 117.269) | 49186 (42420 - 57219) | 45.036 (38.827 - 52.276) | -3.005 (-3.257to-2.752) |
| Gabon | 3 (2 - 4) | 0.505 (0.306 - 0.693) | 6 (4 - 8) | 0.531 (0.369 - 0.705) | 0.13 (0.086-0.173) | 3 (2 - 4) | 0.48 (0.289 - 0.649) | 5 (4 - 7) | 0.462 (0.322 - 0.605) | -0.141 (-0.176to-0.105) | 83 (49 - 116) | 13.612 (8.18 - 18.944) | 160 (108 - 220) | 12.923 (8.815 - 17.363) | -0.201 (-0.249to-0.153) |
| Gambia | 1 (1 - 1) | 0.218 (0.164 - 0.286) | 3 (2 - 4) | 0.237 (0.167 - 0.319) | 0.068 (-0.107-0.244) | 1 (1 - 1) | 0.204 (0.154 - 0.268) | 2 (2 - 3) | 0.21 (0.148 - 0.282) | -0.073 (-0.227-0.082) | 24 (18 - 33) | 5.95 (4.37 - 7.895) | 71 (48 - 96) | 6.136 (4.215 - 8.375) | -0.104 (-0.279-0.073) |
| Georgia | 61 (47 - 81) | 0.955 (0.735 - 1.266) | 73 (64 - 83) | 1.284 (1.123 - 1.467) | 1.31 (0.713-1.91) | 48 (37 - 64) | 0.748 (0.577 - 0.998) | 57 (49 - 65) | 0.985 (0.852 - 1.123) | 1.383 (0.783-1.987) | 1414 (1082 - 1884) | 22.088 (17.008 - 29.458) | 1531 (1318 - 1748) | 27.678 (23.872 - 31.588) | 1.19 (0.606-1.777) |
| Germany | 2758 (2485 - 3040) | 2.487 (2.247 - 2.75) | 5974 (5138 - 6899) | 3.665 (3.154 - 4.261) | 0.487 (0.071-0.904) | 1694 (1533 - 1865) | 1.495 (1.354 - 1.642) | 2777 (2398 - 3194) | 1.604 (1.384 - 1.852) | -0.338 (-0.628to-0.046) | 55034 (49603 - 60725) | 50.677 (45.713 - 55.663) | 70304 (60367 - 81203) | 44.375 (38.045 - 51.356) | -1.081 (-1.403to-0.759) |
| Ghana | 2 (2 - 3) | 0.033 (0.024 - 0.047) | 3 (2 - 4) | 0.015 (0.011 - 0.021) | -3.857 (-4.73to-2.976) | 2 (1 - 3) | 0.031 (0.022 - 0.043) | 2 (2 - 3) | 0.014 (0.01 - 0.02) | -3.838 (-4.663to-3.006) | 69 (49 - 100) | 0.917 (0.65 - 1.305) | 72 (49 - 99) | 0.365 (0.253 - 0.509) | -4.397 (-5.313to-3.471) |
| Greece | 68 (63 - 73) | 0.468 (0.435 - 0.506) | 154 (140 - 167) | 0.838 (0.761 - 0.921) | 1.671 (1.52-1.822) | 30 (28 - 33) | 0.203 (0.191 - 0.219) | 58 (53 - 63) | 0.286 (0.26 - 0.311) | 0.889 (0.7-1.079) | 844 (787 - 915) | 5.872 (5.476 - 6.345) | 1515 (1375 - 1649) | 8.441 (7.668 - 9.214) | 1.012 (0.852-1.172) |
| Greenland | 2 (2 - 3) | 5.721 (4.833 - 6.696) | 3 (2 - 4) | 3.841 (3.005 - 4.903) | -0.926 (-1.094to-0.758) | 2 (1 - 2) | 4.345 (3.691 - 5.053) | 2 (1 - 2) | 2.377 (1.858 - 3.043) | -1.66 (-1.832to-1.487) | 56 (46 - 67) | 129.388 (108.61 - 152.675) | 55 (42 - 70) | 67.393 (52.434 - 86.621) | -1.809 (-1.972to-1.647) |
| Grenada | 1 (1 - 2) | 1.959 (1.684 - 2.284) | 1 (1 - 2) | 1.251 (1.051 - 1.479) | -1.308 (-1.598to-1.017) | 1 (1 - 1) | 1.746 (1.507 - 2.039) | 1 (1 - 1) | 1.001 (0.85 - 1.175) | -1.616 (-1.902to-1.33) | 31 (26 - 36) | 47.622 (40.665 - 55.745) | 32 (27 - 39) | 26.189 (21.883 - 31.398) | -1.644 (-1.996to-1.292) |
| Guam | 0 (0 - 0) | 0.202 (0.172 - 0.236) | 1 (0 - 1) | 0.266 (0.221 - 0.312) | 2.25 (1.773-2.729) | 0 (0 - 0) | 0.154 (0.131 - 0.183) | 0 (0 - 0) | 0.184 (0.151 - 0.216) | 1.877 (1.355-2.402) | 4 (4 - 5) | 4.285 (3.697 - 4.979) | 12 (10 - 13) | 5.941 (4.962 - 6.929) | 2.355 (1.836-2.876) |
| Guatemala | 22 (20 - 23) | 0.634 (0.596 - 0.67) | 46 (39 - 54) | 0.413 (0.352 - 0.482) | -1.868 (-2.152to-1.584) | 21 (19 - 22) | 0.641 (0.605 - 0.679) | 39 (33 - 46) | 0.361 (0.31 - 0.423) | -2.312 (-2.593to-2.03) | 598 (563 - 634) | 15.635 (14.702 - 16.553) | 1056 (897 - 1242) | 9.087 (7.739 - 10.658) | -2.216 (-2.518to-1.914) |
| Guinea | 10 (8 - 13) | 0.301 (0.232 - 0.379) | 22 (15 - 31) | 0.358 (0.25 - 0.503) | 0.637 (0.579-0.695) | 10 (8 - 12) | 0.29 (0.226 - 0.365) | 20 (14 - 28) | 0.335 (0.231 - 0.466) | 0.557 (0.489-0.625) | 293 (228 - 371) | 8.357 (6.471 - 10.564) | 630 (426 - 887) | 9.668 (6.619 - 13.7) | 0.564 (0.494-0.634) |
| Guinea-Bissau | 1 (0 - 1) | 0.163 (0.102 - 0.23) | 2 (1 - 2) | 0.174 (0.124 - 0.234) | 0.137 (0.083-0.191) | 1 (0 - 1) | 0.159 (0.101 - 0.223) | 1 (1 - 2) | 0.164 (0.117 - 0.217) | 0.038 (-0.019-0.094) | 22 (13 - 31) | 4.758 (2.922 - 6.722) | 48 (33 - 66) | 4.953 (3.452 - 6.755) | 0.069 (0.008-0.131) |
| Guyana | 3 (3 - 4) | 0.856 (0.735 - 0.988) | 4 (3 - 6) | 0.639 (0.479 - 0.848) | -0.568 (-0.93to-0.204) | 3 (3 - 4) | 0.817 (0.705 - 0.941) | 4 (3 - 5) | 0.569 (0.43 - 0.748) | -0.747 (-1.104to-0.389) | 93 (79 - 108) | 22.292 (18.959 - 25.99) | 114 (84 - 154) | 15.924 (11.785 - 21.238) | -0.644 (-0.995to-0.292) |
| Haiti | 44 (28 - 66) | 1.308 (0.853 - 1.947) | 74 (47 - 117) | 0.99 (0.642 - 1.518) | -0.768 (-0.904to-0.632) | 43 (27 - 64) | 1.324 (0.861 - 1.961) | 70 (45 - 110) | 0.979 (0.632 - 1.489) | -0.843 (-0.974to-0.711) | 1274 (790 - 1930) | 35.472 (22.415 - 53.504) | 2109 (1290 - 3383) | 25.618 (16.227 - 40.458) | -0.914 (-1.059to-0.768) |
| Honduras | 8 (7 - 10) | 0.399 (0.321 - 0.499) | 33 (25 - 44) | 0.516 (0.392 - 0.677) | 0.987 (0.905-1.07) | 8 (6 - 10) | 0.387 (0.313 - 0.477) | 29 (22 - 38) | 0.468 (0.358 - 0.606) | 0.795 (0.702-0.888) | 225 (182 - 278) | 9.984 (8.064 - 12.276) | 779 (584 - 1044) | 11.481 (8.728 - 15.081) | 0.584 (0.513-0.655) |
| Hungary | 536 (469 - 610) | 3.944 (3.48 - 4.48) | 1016 (816 - 1213) | 6.192 (5.013 - 7.386) | 0.58 (-0.029-1.192) | 376 (326 - 427) | 2.724 (2.388 - 3.089) | 545 (440 - 660) | 3.195 (2.592 - 3.85) | -0.196 (-0.676-0.286) | 12473 (10976 - 14112) | 93.167 (82.508 - 105.088) | 15597 (12703 - 18678) | 97.503 (79.925 - 116.412) | -0.69 (-1.252to-0.126) |
| Iceland | 3 (2 - 3) | 0.971 (0.84 - 1.126) | 6 (5 - 7) | 1.256 (1.057 - 1.477) | 0.789 (0.638-0.941) | 1 (1 - 1) | 0.386 (0.336 - 0.445) | 2 (1 - 2) | 0.317 (0.268 - 0.371) | -0.618 (-0.738to-0.498) | 29 (25 - 34) | 11.177 (9.664 - 12.99) | 48 (40 - 56) | 9.471 (7.962 - 11.234) | -0.562 (-0.681to-0.443) |
| India | 15007 (12541 - 18010) | 2.86 (2.388 - 3.44) | 50833 (44158 - 57647) | 4.002 (3.477 - 4.53) | 1.12 (1.001-1.24) | 13595 (11357 - 16390) | 2.666 (2.226 - 3.224) | 41084 (35623 - 46642) | 3.304 (2.87 - 3.745) | 0.742 (0.664-0.821) | 442152 (370021 - 532320) | 79.679 (66.69 - 95.875) | 1242130 (1075828 - 1416056) | 95.275 (82.554 - 108.59) | 0.623 (0.547-0.699) |
| Indonesia | 486 (360 - 645) | 0.465 (0.344 - 0.618) | 1270 (883 - 1748) | 0.506 (0.355 - 0.693) | 0.164 (0.093-0.235) | 441 (329 - 586) | 0.441 (0.327 - 0.586) | 1061 (731 - 1455) | 0.447 (0.314 - 0.613) | -0.041 (-0.081to-0.001) | 13599 (10246 - 17799) | 11.995 (8.991 - 15.882) | 30697 (21169 - 41877) | 11.401 (7.889 - 15.579) | -0.254 (-0.301to-0.208) |
| Iran  (Islamic Republic of) | 51 (43 - 67) | 0.195 (0.163 - 0.244) | 165 (144 - 213) | 0.207 (0.181 - 0.26) | 0.229 (0.085-0.373) | 42 (35 - 53) | 0.172 (0.144 - 0.21) | 99 (87 - 118) | 0.132 (0.116 - 0.155) | -0.746 (-0.86to-0.632) | 1239 (1040 - 1610) | 4.239 (3.548 - 5.394) | 2658 (2327 - 3308) | 3.2 (2.815 - 3.902) | -0.8 (-0.912to-0.689) |
| Iraq | 29 (22 - 37) | 0.34 (0.261 - 0.438) | 118 (84 - 156) | 0.433 (0.315 - 0.565) | 0.893 (0.748-1.039) | 23 (18 - 30) | 0.283 (0.216 - 0.367) | 71 (52 - 93) | 0.286 (0.21 - 0.369) | 0.068 (-0.022-0.157) | 732 (556 - 944) | 8.254 (6.238 - 10.674) | 2244 (1597 - 3023) | 7.811 (5.645 - 10.291) | -0.124 (-0.205to-0.043) |
| Ireland | 61 (54 - 69) | 1.586 (1.403 - 1.805) | 158 (135 - 187) | 2.179 (1.839 - 2.568) | 1.671 (1.398-1.944) | 31 (28 - 36) | 0.799 (0.703 - 0.91) | 46 (39 - 54) | 0.611 (0.514 - 0.711) | -0.573 (-0.794to-0.352) | 867 (761 - 994) | 22.989 (20.133 - 26.446) | 1272 (1061 - 1498) | 17.628 (14.654 - 20.856) | -0.492 (-0.72to-0.264) |
| Israel | 16 (14 - 18) | 0.339 (0.296 - 0.384) | 57 (49 - 67) | 0.516 (0.446 - 0.608) | 1.325 (1.147-1.504) | 9 (8 - 10) | 0.192 (0.167 - 0.217) | 21 (18 - 25) | 0.184 (0.159 - 0.218) | -0.34 (-0.509to-0.17) | 247 (215 - 280) | 5.454 (4.753 - 6.192) | 579 (504 - 686) | 5.294 (4.62 - 6.246) | -0.278 (-0.453to-0.102) |
| Italy | 1458 (1353 - 1564) | 1.793 (1.667 - 1.925) | 1943 (1746 - 2151) | 1.633 (1.474 - 1.803) | -0.38 (-0.513to-0.248) | 857 (802 - 912) | 1.023 (0.956 - 1.087) | 888 (793 - 980) | 0.679 (0.611 - 0.749) | -1.401 (-1.506to-1.295) | 24738 (23109 - 26412) | 30.967 (28.917 - 33.056) | 21554 (19501 - 23830) | 18.545 (16.847 - 20.488) | -1.733 (-1.832to-1.634) |
| Jamaica | 17 (15 - 19) | 0.984 (0.884 - 1.1) | 26 (20 - 35) | 0.847 (0.638 - 1.129) | -0.293 (-0.78-0.196) | 14 (13 - 16) | 0.818 (0.732 - 0.913) | 20 (15 - 26) | 0.639 (0.482 - 0.842) | -0.577 (-1.07to-0.081) | 352 (316 - 393) | 20.687 (18.51 - 23.158) | 508 (377 - 682) | 16.386 (12.144 - 21.983) | -0.578 (-1.075to-0.078) |
| Japan | 1698 (1628 - 1769) | 0.979 (0.938 - 1.02) | 8261 (7569 - 8775) | 2.718 (2.528 - 2.877) | 3.224 (2.814-3.635) | 749 (720 - 773) | 0.435 (0.416 - 0.448) | 3289 (2980 - 3467) | 0.928 (0.863 - 0.97) | 2.331 (2.024-2.639) | 20332 (19661 - 20936) | 11.71 (11.316 - 12.058) | 66005 (61225 - 68963) | 22.391 (21.174 - 23.281) | 1.944 (1.587-2.302) |
| Jordan | 5 (4 - 6) | 0.346 (0.273 - 0.432) | 31 (22 - 43) | 0.362 (0.259 - 0.498) | 0.324 (0.188-0.46) | 4 (3 - 5) | 0.282 (0.223 - 0.352) | 17 (12 - 23) | 0.218 (0.157 - 0.299) | -0.808 (-0.937to-0.679) | 126 (97 - 161) | 7.761 (6.043 - 9.766) | 515 (362 - 736) | 5.717 (4.025 - 8.151) | -1.013 (-1.145to-0.88) |
| Kazakhstan | 226 (200 - 255) | 1.658 (1.469 - 1.875) | 186 (152 - 225) | 0.957 (0.784 - 1.15) | -2.245 (-2.536to-1.954) | 185 (163 - 209) | 1.385 (1.224 - 1.559) | 129 (107 - 157) | 0.684 (0.567 - 0.826) | -2.823 (-3.162to-2.483) | 5874 (5172 - 6629) | 42.002 (36.976 - 47.475) | 3889 (3201 - 4736) | 19.529 (16.077 - 23.735) | -3.073 (-3.433to-2.712) |
| Kenya | 52 (39 - 68) | 0.581 (0.436 - 0.755) | 214 (152 - 277) | 0.807 (0.578 - 1.039) | 1.293 (1.133-1.454) | 46 (35 - 61) | 0.531 (0.399 - 0.692) | 179 (128 - 231) | 0.707 (0.505 - 0.908) | 1.213 (1.02-1.406) | 1468 (1101 - 1922) | 15.362 (11.493 - 20.136) | 5827 (4122 - 7558) | 20.599 (14.686 - 26.602) | 1.231 (1.019-1.444) |
| Kiribati | 0 (0 - 0) | 0.357 (0.282 - 0.445) | 0 (0 - 0) | 0.369 (0.264 - 0.51) | 0.108 (0.083-0.133) | 0 (0 - 0) | 0.349 (0.277 - 0.437) | 0 (0 - 0) | 0.352 (0.253 - 0.489) | 0.035 (0.011-0.06) | 5 (4 - 6) | 10.598 (8.424 - 13.175) | 10 (7 - 14) | 10.904 (7.602 - 15.231) | 0.084 (0.053-0.115) |
| Kuwait | 6 (5 - 7) | 0.816 (0.719 - 0.916) | 9 (7 - 11) | 0.251 (0.201 - 0.308) | -2.787 (-3.804to-1.759) | 3 (3 - 4) | 0.499 (0.439 - 0.562) | 4 (3 - 5) | 0.125 (0.1 - 0.154) | -3.6 (-4.569to-2.622) | 115 (102 - 129) | 14.085 (12.392 - 15.797) | 112 (89 - 140) | 2.946 (2.331 - 3.642) | -4.096 (-5.082to-3.101) |
| Kyrgyzstan | 19 (15 - 24) | 0.624 (0.494 - 0.769) | 41 (31 - 52) | 0.767 (0.587 - 0.972) | 0.09 (-0.409-0.591) | 17 (13 - 21) | 0.535 (0.426 - 0.658) | 30 (23 - 38) | 0.59 (0.461 - 0.741) | -0.28 (-0.831-0.273) | 525 (408 - 654) | 16.584 (12.993 - 20.623) | 943 (722 - 1207) | 16.807 (12.987 - 21.434) | -0.542 (-1.076to-0.005) |
| Lao People's Democratic Republic | 13 (9 - 19) | 0.603 (0.403 - 0.872) | 24 (17 - 33) | 0.504 (0.361 - 0.679) | -0.623 (-0.78to-0.465) | 13 (9 - 19) | 0.603 (0.409 - 0.866) | 22 (15 - 30) | 0.474 (0.34 - 0.639) | -0.826 (-0.957to-0.694) | 383 (253 - 573) | 16.531 (11.059 - 24.571) | 644 (447 - 887) | 12.31 (8.686 - 16.885) | -1.011 (-1.149to-0.873) |
| Latvia | 62 (52 - 73) | 1.754 (1.469 - 2.067) | 106 (85 - 134) | 3.269 (2.618 - 4.146) | 1.966 (1.535-2.398) | 48 (40 - 56) | 1.334 (1.124 - 1.573) | 65 (52 - 83) | 1.913 (1.543 - 2.441) | 1.167 (0.84-1.495) | 1463 (1234 - 1726) | 41.672 (35.144 - 49.118) | 1837 (1482 - 2343) | 58.233 (46.982 - 73.967) | 0.944 (0.616-1.274) |
| Lebanon | 8 (6 - 12) | 0.36 (0.248 - 0.504) | 22 (16 - 30) | 0.383 (0.282 - 0.504) | 0.729 (0.526-0.932) | 6 (4 - 9) | 0.285 (0.196 - 0.388) | 12 (9 - 16) | 0.207 (0.153 - 0.275) | -0.557 (-0.756to-0.357) | 190 (127 - 266) | 7.917 (5.334 - 10.957) | 316 (235 - 419) | 5.445 (4.023 - 7.199) | -0.774 (-0.96to-0.588) |
| Lesotho | 5 (3 - 6) | 0.514 (0.393 - 0.683) | 11 (8 - 15) | 0.907 (0.66 - 1.215) | 2.215 (1.937-2.494) | 4 (3 - 6) | 0.482 (0.363 - 0.638) | 10 (7 - 13) | 0.837 (0.611 - 1.131) | 2.196 (1.902-2.49) | 126 (94 - 168) | 13.773 (10.358 - 18.399) | 312 (222 - 431) | 25.287 (18.124 - 34.712) | 2.375 (2.038-2.712) |
| Liberia | 1 (1 - 2) | 0.118 (0.082 - 0.173) | 3 (2 - 6) | 0.124 (0.073 - 0.218) | 0.191 (0.007-0.376) | 1 (1 - 2) | 0.115 (0.08 - 0.166) | 3 (2 - 5) | 0.11 (0.066 - 0.192) | -0.081 (-0.255-0.093) | 41 (28 - 60) | 3.307 (2.258 - 4.925) | 91 (51 - 164) | 3.252 (1.88 - 5.883) | 0.006 (-0.184-0.196) |
| Libya | 8 (6 - 10) | 0.379 (0.289 - 0.495) | 27 (18 - 38) | 0.435 (0.3 - 0.611) | 0.699 (0.523-0.875) | 6 (5 - 8) | 0.305 (0.232 - 0.396) | 17 (12 - 24) | 0.292 (0.205 - 0.413) | -0.003 (-0.137-0.131) | 185 (139 - 244) | 8.67 (6.549 - 11.328) | 539 (367 - 774) | 8.315 (5.744 - 11.912) | -0.013 (-0.142-0.116) |
| Lithuania | 75 (63 - 88) | 1.659 (1.402 - 1.941) | 160 (125 - 191) | 3.325 (2.612 - 3.94) | 2.366 (2.245-2.487) | 63 (53 - 74) | 1.395 (1.18 - 1.627) | 125 (98 - 148) | 2.5 (1.962 - 2.957) | 2.111 (1.98-2.241) | 1952 (1648 - 2279) | 43.733 (37.029 - 50.905) | 3561 (2799 - 4232) | 76.298 (60.136 - 90.388) | 1.884 (1.75-2.019) |
| Luxembourg | 15 (14 - 17) | 3.013 (2.786 - 3.291) | 35 (30 - 39) | 3.484 (2.994 - 3.941) | 0.456 (0.237-0.675) | 8 (7 - 9) | 1.549 (1.428 - 1.681) | 11 (10 - 12) | 1.078 (0.943 - 1.211) | -1.277 (-1.45to-1.105) | 240 (221 - 261) | 47.5 (43.797 - 51.822) | 310 (268 - 349) | 31.304 (27.102 - 35.268) | -1.485 (-1.656to-1.315) |
| Madagascar | 39 (26 - 51) | 0.69 (0.472 - 0.918) | 77 (49 - 118) | 0.56 (0.351 - 0.848) | -0.658 (-0.783to-0.532) | 36 (24 - 47) | 0.655 (0.451 - 0.863) | 67 (42 - 104) | 0.512 (0.324 - 0.773) | -0.759 (-0.875to-0.643) | 1144 (774 - 1514) | 19.469 (13.285 - 25.866) | 2304 (1421 - 3572) | 15.247 (9.493 - 23.329) | -0.767 (-0.876to-0.659) |
| Malawi | 4 (3 - 6) | 0.097 (0.072 - 0.13) | 12 (8 - 17) | 0.134 (0.091 - 0.202) | 1.017 (0.762-1.272) | 4 (3 - 5) | 0.094 (0.07 - 0.126) | 10 (7 - 15) | 0.122 (0.082 - 0.182) | 0.838 (0.57-1.107) | 126 (92 - 173) | 2.78 (2.048 - 3.798) | 342 (220 - 519) | 3.729 (2.456 - 5.57) | 0.905 (0.62-1.19) |
| Malaysia | 97 (80 - 117) | 1.01 (0.834 - 1.225) | 330 (273 - 395) | 1.109 (0.925 - 1.326) | -0.029 (-0.255-0.198) | 83 (69 - 100) | 0.892 (0.736 - 1.085) | 235 (195 - 283) | 0.814 (0.682 - 0.974) | -0.677 (-0.887to-0.466) | 2435 (2007 - 2928) | 24.015 (19.806 - 29.028) | 6596 (5430 - 7996) | 21.638 (17.824 - 26.155) | -0.696 (-0.949to-0.443) |
| Maldives | 1 (0 - 1) | 0.77 (0.531 - 1.047) | 2 (2 - 3) | 0.643 (0.487 - 0.839) | -0.831 (-0.944to-0.718) | 1 (0 - 1) | 0.751 (0.522 - 1.011) | 2 (1 - 2) | 0.472 (0.359 - 0.613) | -1.833 (-1.967to-1.699) | 18 (11 - 26) | 18.419 (12.206 - 25.21) | 44 (32 - 58) | 11.221 (8.362 - 14.912) | -1.964 (-2.117to-1.81) |
| Mali | 11 (8 - 14) | 0.241 (0.193 - 0.308) | 25 (17 - 35) | 0.254 (0.175 - 0.353) | 0.432 (0.217-0.647) | 10 (8 - 13) | 0.232 (0.187 - 0.294) | 22 (15 - 31) | 0.235 (0.162 - 0.327) | 0.314 (0.107-0.523) | 313 (249 - 402) | 6.754 (5.389 - 8.687) | 714 (480 - 993) | 6.766 (4.601 - 9.41) | 0.277 (0.061-0.493) |
| Malta | 4 (4 - 4) | 0.939 (0.848 - 1.042) | 11 (10 - 13) | 1.501 (1.287 - 1.736) | 1.508 (1.391-1.625) | 2 (2 - 2) | 0.502 (0.455 - 0.555) | 4 (3 - 5) | 0.475 (0.412 - 0.551) | -0.269 (-0.382to-0.157) | 61 (55 - 68) | 14.422 (13.019 - 16.017) | 106 (92 - 123) | 14.513 (12.464 - 16.835) | -0.012 (-0.117-0.094) |
| Marshall Islands | 0 (0 - 0) | 0.174 (0.121 - 0.254) | 0 (0 - 0) | 0.173 (0.115 - 0.259) | 0.055 (0-0.109) | 0 (0 - 0) | 0.166 (0.117 - 0.243) | 0 (0 - 0) | 0.16 (0.108 - 0.236) | -0.046 (-0.099-0.007) | 1 (1 - 1) | 4.799 (3.295 - 7.133) | 2 (1 - 3) | 4.709 (3.114 - 7.113) | -0.02 (-0.063-0.024) |
| Mauritania | 1 (1 - 2) | 0.122 (0.089 - 0.164) | 3 (2 - 6) | 0.131 (0.081 - 0.242) | -0.018 (-0.178-0.144) | 1 (1 - 2) | 0.116 (0.085 - 0.157) | 2 (2 - 5) | 0.112 (0.069 - 0.213) | -0.337 (-0.462to-0.213) | 37 (27 - 51) | 3.353 (2.44 - 4.564) | 77 (46 - 155) | 3.155 (1.888 - 6.334) | -0.429 (-0.557to-0.3) |
| Mauritius | 3 (3 - 3) | 0.356 (0.332 - 0.38) | 16 (14 - 17) | 0.82 (0.746 - 0.882) | 1.272 (-0.07-2.632) | 2 (2 - 2) | 0.298 (0.279 - 0.319) | 11 (10 - 12) | 0.598 (0.548 - 0.644) | 0.895 (-0.408-2.214) | 65 (60 - 69) | 8.17 (7.621 - 8.735) | 310 (283 - 337) | 16.424 (14.963 - 17.749) | 0.946 (-0.332-2.241) |
| Mexico | 150 (144 - 155) | 0.357 (0.343 - 0.369) | 394 (341 - 455) | 0.308 (0.266 - 0.354) | -0.817 (-1.061to-0.572) | 135 (130 - 140) | 0.337 (0.324 - 0.349) | 301 (259 - 346) | 0.241 (0.208 - 0.277) | -1.352 (-1.585to-1.118) | 3586 (3463 - 3714) | 7.981 (7.698 - 8.265) | 7744 (6679 - 8960) | 5.921 (5.109 - 6.84) | -1.258 (-1.504to-1.011) |
| Micronesia  (Federated States of) | 0 (0 - 0) | 0.189 (0.146 - 0.255) | 0 (0 - 0) | 0.173 (0.125 - 0.239) | -0.317 (-0.42to-0.215) | 0 (0 - 0) | 0.18 (0.141 - 0.243) | 0 (0 - 0) | 0.155 (0.113 - 0.214) | -0.538 (-0.628to-0.448) | 3 (2 - 4) | 5.307 (4.072 - 7.188) | 4 (3 - 6) | 4.596 (3.308 - 6.34) | -0.499 (-0.588to-0.411) |
| Monaco | 0 (0 - 1) | 0.877 (0.651 - 1.154) | 1 (1 - 1) | 1.544 (1.129 - 1.991) | 2.053 (1.754-2.354) | 0 (0 - 0) | 0.339 (0.253 - 0.438) | 0 (0 - 0) | 0.44 (0.333 - 0.563) | 1.017 (0.763-1.271) | 5 (4 - 7) | 10.209 (7.685 - 13.386) | 10 (7 - 12) | 13.394 (9.966 - 17.144) | 1.053 (0.798-1.308) |
| Mongolia | 11 (7 - 14) | 0.953 (0.674 - 1.31) | 17 (13 - 23) | 0.649 (0.475 - 0.877) | -1.616 (-1.937to-1.294) | 10 (7 - 14) | 0.909 (0.652 - 1.274) | 14 (10 - 19) | 0.548 (0.396 - 0.742) | -2.076 (-2.385to-1.766) | 301 (218 - 419) | 25.968 (18.606 - 36.419) | 447 (326 - 606) | 15.677 (11.467 - 21.199) | -2.039 (-2.336to-1.742) |
| Montenegro | 8 (7 - 10) | 1.247 (1.032 - 1.482) | 14 (10 - 18) | 1.452 (1.096 - 1.938) | 0.826 (0.636-1.016) | 5 (4 - 6) | 0.729 (0.602 - 0.87) | 7 (6 - 10) | 0.761 (0.576 - 1.002) | 0.28 (0.17-0.391) | 156 (128 - 186) | 23.29 (19.212 - 27.922) | 217 (161 - 292) | 23.284 (17.33 - 31.426) | 0.149 (0.042-0.256) |
| Morocco | 38 (29 - 48) | 0.254 (0.193 - 0.327) | 112 (76 - 156) | 0.306 (0.212 - 0.42) | 0.691 (0.509-0.874) | 34 (26 - 43) | 0.231 (0.177 - 0.297) | 85 (58 - 117) | 0.237 (0.166 - 0.322) | 0.217 (0.073-0.361) | 1002 (758 - 1288) | 6.509 (4.917 - 8.368) | 2450 (1649 - 3454) | 6.522 (4.41 - 9.15) | 0.121 (-0.006-0.249) |
| Mozambique | 33 (21 - 43) | 0.496 (0.326 - 0.646) | 82 (50 - 114) | 0.638 (0.403 - 0.883) | 1.158 (1.043-1.274) | 31 (20 - 40) | 0.479 (0.32 - 0.623) | 73 (45 - 104) | 0.596 (0.381 - 0.829) | 1.077 (0.947-1.207) | 974 (616 - 1279) | 13.781 (8.873 - 18.042) | 2410 (1442 - 3445) | 17.329 (10.744 - 24.6) | 1.116 (0.982-1.25) |
| Myanmar | 129 (90 - 188) | 0.536 (0.38 - 0.777) | 230 (161 - 322) | 0.458 (0.326 - 0.642) | -0.596 (-0.723to-0.47) | 123 (88 - 180) | 0.527 (0.377 - 0.762) | 200 (140 - 281) | 0.412 (0.294 - 0.577) | -0.872 (-0.961to-0.782) | 3684 (2570 - 5427) | 14.318 (10.13 - 21.108) | 5602 (3935 - 7876) | 10.659 (7.536 - 14.949) | -1.055 (-1.153to-0.956) |
| Namibia | 9 (7 - 12) | 1.257 (0.987 - 1.598) | 25 (18 - 34) | 1.594 (1.145 - 2.162) | 0.72 (0.546-0.894) | 8 (6 - 10) | 1.157 (0.913 - 1.466) | 20 (14 - 28) | 1.336 (0.962 - 1.8) | 0.431 (0.228-0.635) | 256 (200 - 331) | 34.303 (26.723 - 44.061) | 664 (463 - 926) | 40.046 (28.372 - 54.857) | 0.435 (0.207-0.663) |
| Nauru | 0 (0 - 0) | 0.278 (0.161 - 0.412) | 0 (0 - 0) | 0.228 (0.143 - 0.323) | -0.653 (-0.757to-0.548) | 0 (0 - 0) | 0.26 (0.152 - 0.382) | 0 (0 - 0) | 0.202 (0.129 - 0.284) | -0.793 (-0.842to-0.745) | 0 (0 - 1) | 7.568 (4.257 - 11.43) | 0 (0 - 1) | 6.072 (3.695 - 8.756) | -0.718 (-0.769to-0.668) |
| Nepal | 265 (194 - 369) | 2.576 (1.881 - 3.567) | 773 (569 - 1063) | 3.149 (2.332 - 4.3) | 0.816 (0.602-1.031) | 247 (180 - 337) | 2.476 (1.811 - 3.359) | 651 (477 - 906) | 2.719 (2.011 - 3.758) | 0.485 (0.286-0.685) | 7814 (5679 - 10769) | 71.093 (51.613 - 97.628) | 19203 (13933 - 26462) | 75.815 (55.22 - 104.712) | 0.384 (0.181-0.586) |
| Netherlands | 435 (394 - 482) | 2.337 (2.109 - 2.594) | 1254 (1076 - 1471) | 3.972 (3.424 - 4.633) | 1.666 (1.502-1.83) | 106 (95 - 116) | 0.553 (0.496 - 0.607) | 212 (185 - 246) | 0.635 (0.555 - 0.733) | 0.191 (-0.025-0.408) | 3034 (2725 - 3336) | 16.526 (14.849 - 18.207) | 5684 (4881 - 6677) | 18.52 (15.957 - 21.699) | 0.124 (-0.091-0.338) |
| New Zealand | 48 (42 - 55) | 1.258 (1.097 - 1.432) | 114 (96 - 133) | 1.433 (1.209 - 1.669) | 0.749 (0.517-0.982) | 23 (20 - 27) | 0.602 (0.521 - 0.693) | 38 (32 - 44) | 0.46 (0.391 - 0.535) | -0.625 (-0.877to-0.372) | 605 (522 - 692) | 15.929 (13.769 - 18.221) | 952 (807 - 1116) | 12.19 (10.341 - 14.266) | -0.634 (-0.89to-0.376) |
| Nicaragua | 6 (5 - 7) | 0.355 (0.294 - 0.42) | 17 (13 - 23) | 0.343 (0.266 - 0.446) | 0.128 (-0.02-0.276) | 5 (4 - 6) | 0.331 (0.275 - 0.391) | 13 (10 - 17) | 0.27 (0.209 - 0.352) | -0.441 (-0.594to-0.289) | 138 (115 - 164) | 8.289 (6.837 - 9.851) | 354 (275 - 473) | 6.842 (5.303 - 9.053) | -0.433 (-0.571to-0.295) |
| Niger | 3 (2 - 4) | 0.095 (0.068 - 0.128) | 8 (5 - 14) | 0.093 (0.06 - 0.144) | -0.009 (-0.119-0.102) | 3 (2 - 4) | 0.093 (0.068 - 0.125) | 8 (5 - 12) | 0.089 (0.058 - 0.136) | -0.091 (-0.195-0.013) | 87 (60 - 122) | 2.655 (1.887 - 3.688) | 247 (155 - 401) | 2.476 (1.581 - 3.958) | -0.191 (-0.314to-0.068) |
| Nigeria | 97 (65 - 139) | 0.195 (0.131 - 0.278) | 211 (151 - 293) | 0.185 (0.134 - 0.255) | -0.055 (-0.16-0.05) | 89 (60 - 127) | 0.182 (0.123 - 0.258) | 179 (129 - 247) | 0.164 (0.121 - 0.224) | -0.198 (-0.299to-0.096) | 3024 (2031 - 4353) | 5.851 (3.92 - 8.42) | 6246 (4458 - 8580) | 5.07 (3.66 - 7.001) | -0.381 (-0.48to-0.281) |
| Niue | 0 (0 - 0) | 0.179 (0.136 - 0.232) | 0 (0 - 0) | 0.184 (0.137 - 0.245) | -0.057 (-0.098to-0.016) | 0 (0 - 0) | 0.157 (0.12 - 0.203) | 0 (0 - 0) | 0.147 (0.109 - 0.192) | -0.357 (-0.397to-0.318) | 0 (0 - 0) | 4.475 (3.294 - 5.952) | 0 (0 - 0) | 4.198 (3.058 - 5.807) | -0.382 (-0.429to-0.335) |
| North Macedonia | 17 (15 - 21) | 0.863 (0.741 - 1.016) | 40 (31 - 54) | 1.181 (0.916 - 1.577) | 1.341 (1.052-1.63) | 14 (12 - 16) | 0.678 (0.579 - 0.801) | 25 (19 - 33) | 0.733 (0.571 - 0.978) | 0.443 (0.206-0.679) | 427 (367 - 506) | 20.746 (17.874 - 24.539) | 723 (558 - 976) | 21.462 (16.517 - 28.792) | 0.294 (0.054-0.533) |
| Northern Mariana Islands | 0 (0 - 0) | 1.133 (0.889 - 1.471) | 0 (0 - 1) | 0.832 (0.679 - 1.006) | -1.051 (-1.406to-0.693) | 0 (0 - 0) | 0.884 (0.694 - 1.145) | 0 (0 - 0) | 0.61 (0.496 - 0.733) | -1.118 (-1.417to-0.818) | 7 (5 - 10) | 25.298 (19.072 - 32.931) | 10 (8 - 12) | 17.086 (13.92 - 20.559) | -1.223 (-1.505to-0.941) |
| Norway | 79 (73 - 86) | 1.353 (1.251 - 1.47) | 162 (143 - 184) | 1.799 (1.586 - 2.036) | 1.365 (0.975-1.757) | 37 (34 - 39) | 0.585 (0.541 - 0.628) | 43 (38 - 49) | 0.449 (0.395 - 0.507) | -0.585 (-0.899to-0.271) | 926 (855 - 997) | 16.199 (15.001 - 17.386) | 1077 (950 - 1217) | 12.036 (10.608 - 13.624) | -0.672 (-0.986to-0.357) |
| Oman | 3 (2 - 4) | 0.394 (0.269 - 0.57) | 11 (7 - 15) | 0.419 (0.289 - 0.59) | 0.489 (0.269-0.709) | 2 (2 - 3) | 0.319 (0.218 - 0.459) | 5 (4 - 8) | 0.238 (0.168 - 0.33) | -0.56 (-0.817to-0.302) | 73 (49 - 105) | 8.958 (6.034 - 13.076) | 176 (120 - 260) | 6.458 (4.429 - 9.174) | -0.716 (-0.986to-0.445) |
| Pakistan | 1650 (1338 - 2003) | 2.781 (2.244 - 3.374) | 4703 (3556 - 6196) | 3.417 (2.582 - 4.51) | 0.46 (0.286-0.633) | 1537 (1242 - 1859) | 2.643 (2.129 - 3.192) | 4053 (3069 - 5344) | 3.071 (2.336 - 4.039) | 0.287 (0.084-0.491) | 46844 (38165 - 57040) | 75.648 (61.325 - 92.091) | 130925 (98365 - 175028) | 88.362 (66.739 - 116.724) | 0.293 (0.085-0.501) |
| Palau | 0 (0 - 0) | 0.043 (0.029 - 0.062) | 0 (0 - 0) | 0.035 (0.023 - 0.05) | -0.554 (-0.689to-0.42) | 0 (0 - 0) | 0.037 (0.026 - 0.052) | 0 (0 - 0) | 0.027 (0.019 - 0.039) | -0.758 (-0.91to-0.606) | 0 (0 - 0) | 0.925 (0.628 - 1.35) | 0 (0 - 0) | 0.66 (0.442 - 0.968) | -0.922 (-1.079to-0.764) |
| Palestine | 1 (1 - 2) | 0.159 (0.117 - 0.219) | 4 (3 - 5) | 0.152 (0.116 - 0.189) | -0.128 (-0.297-0.042) | 1 (1 - 2) | 0.14 (0.106 - 0.194) | 3 (2 - 3) | 0.108 (0.083 - 0.134) | -0.779 (-1.021to-0.537) | 32 (23 - 45) | 3.403 (2.478 - 4.771) | 74 (57 - 92) | 2.559 (1.97 - 3.166) | -0.881 (-1.085to-0.675) |
| Panama | 15 (14 - 17) | 1.036 (0.961 - 1.123) | 39 (31 - 48) | 0.892 (0.706 - 1.082) | -0.314 (-0.579to-0.048) | 13 (12 - 14) | 0.902 (0.837 - 0.977) | 28 (22 - 34) | 0.632 (0.498 - 0.764) | -0.878 (-1.12to-0.635) | 334 (311 - 361) | 21.733 (20.273 - 23.482) | 673 (525 - 820) | 15.228 (11.885 - 18.561) | -0.885 (-1.13to-0.64) |
| Papua New Guinea | 2 (2 - 4) | 0.122 (0.082 - 0.182) | 7 (5 - 10) | 0.119 (0.082 - 0.167) | -0.123 (-0.244to0) | 2 (1 - 3) | 0.116 (0.078 - 0.175) | 6 (4 - 9) | 0.111 (0.077 - 0.156) | -0.127 (-0.231to-0.023) | 75 (48 - 115) | 3.315 (2.154 - 4.985) | 211 (141 - 299) | 3.162 (2.14 - 4.453) | -0.171 (-0.29to-0.052) |
| Paraguay | 19 (15 - 23) | 0.814 (0.653 - 1.024) | 81 (56 - 114) | 1.336 (0.94 - 1.869) | 1.589 (1.443-1.735) | 16 (13 - 20) | 0.706 (0.567 - 0.882) | 60 (42 - 84) | 1.015 (0.712 - 1.408) | 1.198 (1.031-1.365) | 465 (374 - 589) | 19.698 (15.863 - 24.98) | 1755 (1206 - 2482) | 28.369 (19.525 - 39.909) | 1.17 (0.99-1.351) |
| Peru | 50 (41 - 60) | 0.404 (0.333 - 0.489) | 107 (78 - 146) | 0.316 (0.23 - 0.428) | -0.987 (-1.432to-0.539) | 45 (37 - 55) | 0.374 (0.31 - 0.458) | 72 (53 - 97) | 0.214 (0.158 - 0.288) | -2.037 (-2.434to-1.637) | 1278 (1052 - 1561) | 9.823 (8.128 - 12.025) | 1873 (1356 - 2546) | 5.435 (3.939 - 7.383) | -2.167 (-2.572to-1.76) |
| Philippines | 143 (120 - 175) | 0.457 (0.385 - 0.56) | 404 (328 - 498) | 0.47 (0.384 - 0.58) | 0.003 (-0.054-0.06) | 124 (104 - 152) | 0.42 (0.354 - 0.518) | 340 (276 - 416) | 0.411 (0.337 - 0.503) | -0.069 (-0.105to-0.033) | 3846 (3267 - 4676) | 11.149 (9.437 - 13.683) | 9894 (7930 - 12114) | 10.85 (8.764 - 13.301) | -0.189 (-0.243to-0.136) |
| Poland | 708 (673 - 745) | 1.65 (1.57 - 1.734) | 2313 (2057 - 2546) | 3.578 (3.191 - 3.945) | 2.882 (2.569-3.196) | 550 (522 - 577) | 1.276 (1.214 - 1.337) | 1289 (1158 - 1426) | 1.928 (1.731 - 2.132) | 1.662 (1.291-2.035) | 16937 (16110 - 17738) | 39.832 (37.954 - 41.658) | 35614 (31939 - 39551) | 56.772 (50.969 - 63.04) | 1.452 (1.117-1.787) |
| Portugal | 225 (202 - 251) | 1.731 (1.546 - 1.936) | 951 (828 - 1068) | 5.114 (4.465 - 5.791) | 3.869 (3.474-4.266) | 135 (122 - 150) | 1.013 (0.912 - 1.125) | 308 (269 - 346) | 1.529 (1.33 - 1.717) | 1.432 (1.203-1.662) | 3985 (3561 - 4426) | 31.113 (27.721 - 34.648) | 8844 (7686 - 9921) | 47.938 (41.584 - 53.976) | 1.549 (1.265-1.833) |
| Puerto Rico | 80 (71 - 88) | 2.234 (1.999 - 2.47) | 68 (54 - 83) | 1.115 (0.894 - 1.365) | -1.742 (-2.13to-1.352) | 59 (53 - 66) | 1.661 (1.494 - 1.842) | 39 (31 - 48) | 0.586 (0.47 - 0.719) | -2.929 (-3.303to-2.553) | 1564 (1394 - 1736) | 43.886 (39.127 - 48.708) | 932 (745 - 1147) | 16.003 (12.773 - 19.657) | -2.821 (-3.202to-2.439) |
| Qatar | 1 (1 - 1) | 0.876 (0.668 - 1.144) | 7 (5 - 11) | 0.551 (0.367 - 0.865) | -1.645 (-1.815to-1.475) | 1 (1 - 1) | 0.734 (0.56 - 0.969) | 3 (2 - 4) | 0.299 (0.2 - 0.466) | -3.231 (-3.431to-3.032) | 27 (20 - 36) | 17.035 (12.902 - 22.514) | 97 (64 - 149) | 6.701 (4.374 - 10.784) | -3.215 (-3.357to-3.073) |
| Republic of Korea | 166 (139 - 196) | 0.512 (0.431 - 0.606) | 1308 (989 - 1615) | 1.375 (1.048 - 1.687) | 3.956 (3.478-4.435) | 115 (96 - 137) | 0.374 (0.314 - 0.44) | 463 (360 - 569) | 0.483 (0.376 - 0.592) | 1.242 (0.909-1.577) | 3427 (2866 - 4069) | 10.125 (8.477 - 12.058) | 11274 (8551 - 14124) | 11.868 (9.09 - 14.69) | 0.921 (0.601-1.243) |
| Republic of Moldova | 106 (95 - 119) | 2.271 (2.035 - 2.54) | 238 (206 - 279) | 4.116 (3.562 - 4.802) | 2.273 (1.86-2.687) | 87 (78 - 98) | 1.893 (1.699 - 2.115) | 160 (140 - 187) | 2.727 (2.379 - 3.168) | 1.564 (1.209-1.921) | 2749 (2457 - 3084) | 58.77 (52.666 - 65.951) | 4796 (4174 - 5607) | 83.879 (72.981 - 97.934) | 1.49 (1.153-1.827) |
| Romania | 443 (391 - 505) | 1.569 (1.397 - 1.777) | 1574 (1279 - 1880) | 5.011 (4.095 - 5.961) | 3.908 (3.667-4.15) | 348 (307 - 397) | 1.233 (1.09 - 1.39) | 955 (780 - 1137) | 2.949 (2.411 - 3.503) | 2.885 (2.671-3.099) | 11345 (10007 - 12884) | 40.448 (35.996 - 45.527) | 28700 (23485 - 34050) | 93.638 (76.768 - 111.182) | 2.752 (2.502-3.002) |
| Russian Federation | 4058 (3922 - 4204) | 2.202 (2.128 - 2.28) | 8236 (7308 - 9110) | 3.681 (3.27 - 4.064) | 1.402 (1.062-1.744) | 2399 (2316 - 2487) | 1.296 (1.252 - 1.343) | 3462 (3098 - 3841) | 1.48 (1.325 - 1.639) | -0.006 (-0.241-0.23) | 76383 (73729 - 79189) | 41.499 (40.118 - 43.015) | 104223 (92774 - 115436) | 46.371 (41.302 - 51.254) | -0.109 (-0.358-0.141) |
| Rwanda | 33 (24 - 44) | 1.044 (0.746 - 1.366) | 60 (37 - 88) | 0.822 (0.526 - 1.22) | -1.398 (-1.672to-1.124) | 31 (22 - 41) | 1.011 (0.72 - 1.321) | 52 (32 - 78) | 0.74 (0.474 - 1.106) | -1.644 (-1.903to-1.384) | 1022 (722 - 1376) | 30.533 (21.806 - 40.806) | 1670 (1035 - 2554) | 21.571 (13.492 - 32.779) | -1.816 (-2.095to-1.536) |
| Saint Kitts and Nevis | 1 (1 - 1) | 1.584 (1.45 - 1.715) | 1 (1 - 1) | 1.077 (0.856 - 1.297) | -0.805 (-1.337to-0.271) | 1 (0 - 1) | 1.48 (1.357 - 1.602) | 1 (0 - 1) | 0.866 (0.692 - 1.032) | -1.279 (-1.823to-0.732) | 13 (12 - 14) | 39.168 (35.845 - 42.546) | 17 (13 - 21) | 21.962 (17.234 - 26.687) | -1.466 (-2.062to-0.867) |
| Saint Lucia | 2 (2 - 2) | 1.897 (1.768 - 2.042) | 3 (2 - 4) | 1.221 (0.986 - 1.499) | -1.387 (-1.866to-0.906) | 1 (1 - 2) | 1.721 (1.607 - 1.847) | 2 (2 - 3) | 0.962 (0.779 - 1.17) | -1.85 (-2.372to-1.326) | 39 (36 - 42) | 44.725 (41.666 - 48.072) | 62 (50 - 76) | 25.343 (20.435 - 30.849) | -1.747 (-2.236to-1.255) |
| Saint Vincent and the Grenadines | 2 (1 - 2) | 2.203 (2.009 - 2.408) | 3 (2 - 3) | 1.809 (1.557 - 2.088) | -0.492 (-0.996-0.016) | 1 (1 - 2) | 1.979 (1.803 - 2.168) | 2 (2 - 2) | 1.493 (1.294 - 1.714) | -0.739 (-1.244to-0.231) | 36 (33 - 39) | 51.118 (46.453 - 56.098) | 58 (49 - 67) | 39.797 (34.14 - 46.009) | -0.654 (-1.144to-0.162) |
| Samoa | 0 (0 - 0) | 0.235 (0.184 - 0.298) | 0 (0 - 0) | 0.229 (0.166 - 0.302) | -0.111 (-0.194to-0.028) | 0 (0 - 0) | 0.213 (0.17 - 0.269) | 0 (0 - 0) | 0.192 (0.14 - 0.258) | -0.344 (-0.425to-0.264) | 6 (4 - 7) | 5.943 (4.561 - 7.668) | 9 (6 - 12) | 5.439 (3.909 - 7.489) | -0.273 (-0.368to-0.177) |
| San Marino | 0 (0 - 1) | 1.54 (1.217 - 1.912) | 1 (0 - 1) | 1.084 (0.634 - 1.66) | -0.13 (-0.539-0.28) | 0 (0 - 0) | 0.604 (0.476 - 0.754) | 0 (0 - 0) | 0.325 (0.194 - 0.492) | -0.936 (-1.303to-0.567) | 6 (4 - 7) | 17.858 (14.065 - 22.399) | 6 (3 - 9) | 9.698 (5.523 - 14.7) | -0.944 (-1.3to-0.586) |
| Sao Tome and Principe | 0 (0 - 0) | 0.017 (0.01 - 0.024) | 0 (0 - 0) | 0.019 (0.012 - 0.027) | 0.476 (0.371-0.581) | 0 (0 - 0) | 0.018 (0.009 - 0.024) | 0 (0 - 0) | 0.018 (0.01 - 0.026) | 0.161 (0.07-0.252) | 0 (0 - 0) | 0.366 (0.236 - 0.477) | 0 (0 - 1) | 0.379 (0.25 - 0.523) | 0.186 (0.134-0.237) |
| Saudi Arabia | 26 (18 - 36) | 0.395 (0.282 - 0.546) | 137 (96 - 190) | 0.498 (0.366 - 0.668) | 0.755 (0.64-0.87) | 21 (15 - 29) | 0.342 (0.244 - 0.474) | 66 (47 - 89) | 0.285 (0.21 - 0.387) | -0.659 (-0.87to-0.448) | 679 (481 - 942) | 9.442 (6.729 - 13.069) | 2382 (1663 - 3275) | 7.916 (5.724 - 10.636) | -0.636 (-0.834to-0.437) |
| Senegal | 4 (3 - 6) | 0.124 (0.096 - 0.16) | 12 (8 - 18) | 0.143 (0.097 - 0.203) | 0.389 (0.296-0.482) | 4 (3 - 5) | 0.118 (0.092 - 0.151) | 11 (7 - 15) | 0.129 (0.088 - 0.182) | 0.25 (0.166-0.335) | 125 (94 - 166) | 3.431 (2.615 - 4.519) | 341 (229 - 513) | 3.743 (2.522 - 5.549) | 0.257 (0.164-0.351) |
| Serbia | 240 (196 - 299) | 1.986 (1.629 - 2.421) | 362 (289 - 454) | 2.458 (1.967 - 3.083) | 0.833 (0.718-0.948) | 189 (153 - 235) | 1.587 (1.301 - 1.942) | 219 (175 - 274) | 1.446 (1.163 - 1.809) | -0.234 (-0.343to-0.126) | 5864 (4705 - 7270) | 47.769 (38.477 - 59.056) | 6126 (4846 - 7702) | 43.161 (34.128 - 54.134) | -0.313 (-0.447to-0.18) |
| Seychelles | 2 (1 - 2) | 2.823 (2.298 - 3.427) | 5 (4 - 6) | 3.943 (3.199 - 4.736) | 0.981 (0.558-1.405) | 1 (1 - 2) | 2.454 (2.011 - 2.988) | 4 (3 - 4) | 3.059 (2.498 - 3.667) | 0.629 (0.215-1.044) | 39 (32 - 47) | 70.485 (56.941 - 85.807) | 107 (86 - 129) | 83.209 (66.886 - 100.017) | 0.391 (-0.031-0.814) |
| Sierra Leone | 3 (2 - 4) | 0.119 (0.087 - 0.167) | 6 (3 - 9) | 0.129 (0.082 - 0.195) | 0.267 (0.154-0.38) | 2 (2 - 3) | 0.114 (0.084 - 0.159) | 5 (3 - 7) | 0.118 (0.075 - 0.176) | 0.132 (0.043-0.221) | 72 (51 - 104) | 3.313 (2.344 - 4.76) | 159 (97 - 256) | 3.478 (2.135 - 5.448) | 0.222 (0.118-0.325) |
| Singapore | 25 (21 - 28) | 1.045 (0.902 - 1.187) | 134 (115 - 155) | 1.513 (1.306 - 1.747) | 1.665 (1.43-1.901) | 14 (12 - 17) | 0.64 (0.552 - 0.727) | 48 (42 - 55) | 0.548 (0.478 - 0.63) | -0.249 (-0.423to-0.074) | 418 (355 - 478) | 17.271 (14.717 - 19.75) | 1187 (1029 - 1387) | 13.388 (11.617 - 15.64) | -0.509 (-0.693to-0.325) |
| Slovakia | 254 (210 - 320) | 4.457 (3.685 - 5.59) | 490 (371 - 641) | 5.644 (4.272 - 7.391) | 0.796 (0.612-0.981) | 174 (144 - 217) | 3.008 (2.495 - 3.748) | 274 (204 - 356) | 3.053 (2.29 - 3.972) | 0.041 (-0.148-0.231) | 5678 (4674 - 7087) | 100.05 (82.421 - 124.474) | 8158 (6073 - 10688) | 95.052 (71.189 - 124.339) | -0.235 (-0.433to-0.035) |
| Slovenia | 89 (76 - 102) | 3.604 (3.113 - 4.134) | 184 (140 - 237) | 4.853 (3.66 - 6.277) | 1.132 (0.932-1.333) | 64 (56 - 73) | 2.609 (2.265 - 2.969) | 95 (71 - 122) | 2.386 (1.786 - 3.072) | -0.276 (-0.464to-0.088) | 2023 (1744 - 2316) | 82.709 (71.513 - 94.595) | 2560 (1903 - 3319) | 70.276 (51.975 - 91.473) | -0.551 (-0.743to-0.359) |
| Solomon Islands | 0 (0 - 0) | 0.153 (0.106 - 0.214) | 1 (0 - 1) | 0.152 (0.11 - 0.211) | -0.022 (-0.177-0.133) | 0 (0 - 0) | 0.148 (0.103 - 0.208) | 1 (0 - 1) | 0.141 (0.102 - 0.194) | -0.157 (-0.293to-0.02) | 7 (5 - 10) | 4.247 (2.882 - 5.972) | 19 (13 - 27) | 4.193 (3.043 - 5.896) | -0.026 (-0.166-0.114) |
| Somalia | 20 (13 - 28) | 0.672 (0.446 - 0.961) | 43 (27 - 65) | 0.586 (0.375 - 0.856) | -0.44 (-0.469to-0.411) | 18 (12 - 26) | 0.65 (0.434 - 0.926) | 40 (25 - 59) | 0.563 (0.364 - 0.818) | -0.446 (-0.473to-0.419) | 633 (418 - 932) | 19.545 (12.929 - 28.224) | 1369 (863 - 2048) | 16.718 (10.601 - 24.773) | -0.527 (-0.557to-0.498) |
| South Africa | 146 (122 - 181) | 0.651 (0.54 - 0.812) | 423 (362 - 487) | 0.84 (0.721 - 0.963) | 0.934 (0.809-1.058) | 122 (102 - 153) | 0.561 (0.464 - 0.706) | 328 (281 - 377) | 0.669 (0.573 - 0.766) | 0.66 (0.474-0.846) | 3996 (3382 - 4929) | 17.085 (14.349 - 21.308) | 10212 (8712 - 11825) | 19.673 (16.88 - 22.741) | 0.54 (0.366-0.714) |
| South Sudan | 23 (15 - 33) | 0.831 (0.549 - 1.224) | 33 (21 - 49) | 0.726 (0.471 - 1.048) | -0.51 (-0.633to-0.387) | 21 (14 - 32) | 0.793 (0.52 - 1.174) | 29 (19 - 43) | 0.669 (0.438 - 0.96) | -0.615 (-0.721to-0.51) | 654 (432 - 976) | 23.308 (15.219 - 34.766) | 973 (613 - 1448) | 19.549 (12.523 - 28.771) | -0.64 (-0.766to-0.514) |
| Spain | 1087 (954 - 1235) | 2.23 (1.955 - 2.54) | 2552 (2148 - 3085) | 3.113 (2.62 - 3.775) | 0.584 (0.295-0.874) | 538 (472 - 609) | 1.071 (0.943 - 1.207) | 844 (705 - 1008) | 0.97 (0.811 - 1.166) | -0.786 (-0.977to-0.595) | 16452 (14487 - 18558) | 34.431 (30.35 - 38.779) | 22524 (18884 - 27042) | 27.839 (23.344 - 33.469) | -1.26 (-1.483to-1.036) |
| Sri Lanka | 203 (165 - 250) | 1.848 (1.515 - 2.265) | 421 (256 - 613) | 1.514 (0.931 - 2.189) | -0.902 (-1.183to-0.62) | 174 (142 - 215) | 1.66 (1.369 - 2.031) | 275 (171 - 395) | 1.004 (0.63 - 1.425) | -1.938 (-2.24to-1.635) | 4930 (3979 - 6104) | 42.107 (34.278 - 52.173) | 7454 (4516 - 10956) | 26.842 (16.205 - 39.305) | -1.741 (-2.036to-1.444) |
| Sudan | 25 (17 - 39) | 0.256 (0.179 - 0.404) | 49 (33 - 69) | 0.23 (0.159 - 0.317) | -0.435 (-0.524to-0.345) | 23 (16 - 36) | 0.245 (0.173 - 0.38) | 38 (26 - 52) | 0.193 (0.134 - 0.268) | -0.819 (-0.869to-0.769) | 674 (459 - 1073) | 6.539 (4.499 - 10.395) | 1174 (771 - 1679) | 5 (3.37 - 6.936) | -0.919 (-0.968to-0.87) |
| Suriname | 2 (2 - 2) | 0.758 (0.624 - 0.896) | 4 (3 - 5) | 0.55 (0.409 - 0.731) | -0.607 (-0.865to-0.349) | 2 (1 - 2) | 0.701 (0.575 - 0.822) | 3 (2 - 4) | 0.47 (0.353 - 0.627) | -0.849 (-1.095to-0.602) | 51 (42 - 62) | 18.654 (15.176 - 22.287) | 86 (63 - 115) | 12.813 (9.442 - 17.008) | -0.82 (-1.076to-0.563) |
| Sweden | 150 (134 - 168) | 1.14 (1.019 - 1.273) | 263 (224 - 306) | 1.421 (1.203 - 1.655) | 1.316 (1.039-1.593) | 84 (75 - 94) | 0.597 (0.533 - 0.667) | 107 (90 - 125) | 0.515 (0.435 - 0.6) | 0.068 (-0.14-0.276) | 2038 (1823 - 2278) | 15.921 (14.228 - 17.748) | 2374 (1997 - 2758) | 12.933 (10.814 - 15.028) | -0.024 (-0.268-0.219) |
| Switzerland | 163 (148 - 181) | 1.766 (1.607 - 1.97) | 393 (335 - 465) | 2.531 (2.161 - 2.975) | 1.039 (0.652-1.427) | 87 (79 - 96) | 0.901 (0.818 - 1.005) | 164 (139 - 195) | 0.975 (0.838 - 1.159) | 0.149 (-0.119-0.418) | 2418 (2196 - 2696) | 26.579 (24.189 - 29.647) | 4105 (3514 - 4873) | 26.711 (22.912 - 31.621) | -0.131 (-0.415-0.155) |
| Syrian Arab Republic | 17 (13 - 21) | 0.307 (0.231 - 0.391) | 47 (33 - 63) | 0.345 (0.249 - 0.463) | 0.196 (0.096-0.295) | 14 (11 - 18) | 0.275 (0.212 - 0.351) | 29 (21 - 40) | 0.239 (0.174 - 0.322) | -0.638 (-0.742to-0.533) | 424 (321 - 545) | 7.111 (5.408 - 9.112) | 822 (584 - 1130) | 5.803 (4.196 - 7.901) | -0.856 (-0.946to-0.765) |
| Taiwan  (Province of China) | 307 (283 - 331) | 1.81 (1.669 - 1.945) | 2102 (1864 - 2344) | 5.252 (4.705 - 5.843) | 3.66 (3.27-4.052) | 195 (180 - 209) | 1.188 (1.102 - 1.277) | 922 (813 - 1021) | 2.258 (2.004 - 2.489) | 2.172 (1.873-2.472) | 6003 (5562 - 6446) | 34.414 (31.792 - 37.158) | 27798 (24512 - 30696) | 70.687 (62.673 - 77.73) | 2.41 (2.059-2.763) |
| Tajikistan | 20 (15 - 28) | 0.719 (0.531 - 1.007) | 32 (22 - 45) | 0.487 (0.346 - 0.669) | -1.454 (-1.583to-1.324) | 18 (13 - 26) | 0.665 (0.493 - 0.931) | 26 (18 - 36) | 0.422 (0.303 - 0.573) | -1.642 (-1.805to-1.478) | 528 (379 - 753) | 17.784 (12.958 - 24.905) | 841 (565 - 1155) | 11.726 (8.132 - 16.161) | -1.496 (-1.616to-1.375) |
| Thailand | 332 (251 - 427) | 0.856 (0.646 - 1.096) | 1012 (747 - 1407) | 0.934 (0.69 - 1.289) | -0.261 (-0.472to-0.05) | 266 (202 - 342) | 0.715 (0.547 - 0.909) | 636 (465 - 876) | 0.584 (0.428 - 0.798) | -1.245 (-1.465to-1.025) | 8276 (6299 - 10747) | 20.139 (15.406 - 25.935) | 18110 (13253 - 24942) | 16.943 (12.461 - 23.091) | -1.167 (-1.41to-0.923) |
| Timor-Leste | 1 (1 - 2) | 0.401 (0.27 - 0.56) | 4 (2 - 5) | 0.403 (0.287 - 0.568) | 0.083 (-0.161-0.327) | 1 (1 - 2) | 0.397 (0.266 - 0.552) | 3 (2 - 5) | 0.376 (0.265 - 0.527) | -0.147 (-0.371-0.078) | 36 (23 - 51) | 10.421 (6.932 - 14.717) | 87 (61 - 125) | 9.64 (6.721 - 13.71) | -0.244 (-0.51-0.023) |
| Togo | 2 (1 - 2) | 0.121 (0.088 - 0.166) | 7 (5 - 11) | 0.155 (0.103 - 0.235) | 0.636 (0.548-0.723) | 2 (1 - 2) | 0.114 (0.084 - 0.157) | 6 (4 - 10) | 0.138 (0.093 - 0.214) | 0.478 (0.376-0.58) | 50 (35 - 70) | 3.316 (2.36 - 4.657) | 207 (131 - 328) | 4.119 (2.679 - 6.499) | 0.574 (0.466-0.682) |
| Tokelau | 0 (0 - 0) | 0.165 (0.119 - 0.227) | 0 (0 - 0) | 0.155 (0.11 - 0.233) | -0.263 (-0.331to-0.195) | 0 (0 - 0) | 0.153 (0.11 - 0.215) | 0 (0 - 0) | 0.126 (0.089 - 0.19) | -0.666 (-0.706to-0.625) | 0 (0 - 0) | 4.263 (3.012 - 6.066) | 0 (0 - 0) | 3.656 (2.581 - 5.529) | -0.58 (-0.642to-0.518) |
| Tonga | 0 (0 - 0) | 0.136 (0.097 - 0.189) | 0 (0 - 0) | 0.142 (0.097 - 0.215) | 0.127 (0.051-0.204) | 0 (0 - 0) | 0.12 (0.085 - 0.168) | 0 (0 - 0) | 0.118 (0.081 - 0.182) | -0.045 (-0.145-0.055) | 2 (1 - 3) | 3.262 (2.33 - 4.567) | 3 (2 - 4) | 3.257 (2.2 - 4.967) | 0.012 (-0.067-0.09) |
| Trinidad and Tobago | 11 (10 - 12) | 1.31 (1.223 - 1.404) | 18 (13 - 23) | 0.92 (0.688 - 1.184) | -1.098 (-1.547to-0.647) | 10 (9 - 10) | 1.17 (1.095 - 1.248) | 14 (10 - 18) | 0.704 (0.526 - 0.903) | -1.641 (-2.074to-1.206) | 262 (243 - 281) | 30.52 (28.367 - 32.761) | 371 (277 - 482) | 19.153 (14.336 - 24.884) | -1.548 (-1.965to-1.13) |
| Tunisia | 30 (23 - 37) | 0.56 (0.439 - 0.699) | 96 (64 - 136) | 0.696 (0.467 - 0.979) | 0.564 (0.514-0.615) | 23 (18 - 28) | 0.446 (0.351 - 0.553) | 55 (37 - 76) | 0.405 (0.277 - 0.561) | -0.426 (-0.489to-0.362) | 662 (517 - 827) | 12.084 (9.387 - 15.1) | 1540 (1029 - 2174) | 11.009 (7.368 - 15.485) | -0.44 (-0.503to-0.377) |
| Türkiye | 113 (86 - 145) | 0.312 (0.24 - 0.396) | 302 (227 - 381) | 0.317 (0.24 - 0.398) | -0.028 (-0.175-0.118) | 96 (73 - 124) | 0.279 (0.214 - 0.355) | 179 (136 - 225) | 0.193 (0.147 - 0.242) | -1.358 (-1.507to-1.208) | 2882 (2160 - 3769) | 7.421 (5.609 - 9.619) | 4714 (3534 - 5952) | 4.887 (3.663 - 6.154) | -1.55 (-1.68to-1.42) |
| Turkmenistan | 32 (28 - 36) | 1.546 (1.371 - 1.732) | 58 (44 - 78) | 1.295 (0.974 - 1.722) | -0.905 (-1.383to-0.425) | 28 (25 - 31) | 1.376 (1.214 - 1.538) | 46 (34 - 62) | 1.055 (0.8 - 1.412) | -1.197 (-1.64to-0.753) | 883 (780 - 986) | 40.632 (35.923 - 45.389) | 1448 (1077 - 1971) | 30.858 (23.096 - 41.784) | -1.222 (-1.665to-0.778) |
| Tuvalu | 0 (0 - 0) | 0.157 (0.119 - 0.207) | 0 (0 - 0) | 0.158 (0.121 - 0.214) | -0.062 (-0.097to-0.028) | 0 (0 - 0) | 0.15 (0.114 - 0.197) | 0 (0 - 0) | 0.139 (0.107 - 0.189) | -0.312 (-0.34to-0.284) | 0 (0 - 0) | 4.344 (3.251 - 5.756) | 0 (0 - 1) | 4.078 (3.046 - 5.569) | -0.249 (-0.283to-0.215) |
| Uganda | 108 (78 - 141) | 1.545 (1.133 - 2.021) | 327 (214 - 453) | 1.923 (1.296 - 2.645) | 0.381 (0.161-0.602) | 99 (73 - 131) | 1.462 (1.087 - 1.907) | 281 (186 - 396) | 1.725 (1.174 - 2.39) | 0.224 (-0.017-0.466) | 3123 (2248 - 4176) | 42.434 (30.842 - 56.351) | 9285 (6019 - 13178) | 50.304 (33.027 - 71.099) | 0.175 (-0.082-0.433) |
| Ukraine | 1289 (1005 - 1771) | 1.791 (1.399 - 2.457) | 2338 (1560 - 3312) | 3.302 (2.215 - 4.68) | 2.031 (1.791-2.272) | 860 (666 - 1174) | 1.182 (0.914 - 1.617) | 1370 (879 - 1955) | 1.886 (1.211 - 2.685) | 1.46 (1.265-1.655) | 27179 (21011 - 37566) | 38.021 (29.385 - 53.001) | 42554 (27232 - 60680) | 61.197 (39.134 - 87.033) | 1.436 (1.235-1.637) |
| United Arab Emirates | 6 (4 - 10) | 1.153 (0.693 - 1.732) | 44 (32 - 58) | 1.005 (0.766 - 1.282) | 0.859 (0.442-1.278) | 5 (3 - 8) | 1.011 (0.618 - 1.495) | 25 (18 - 32) | 0.755 (0.578 - 0.962) | 0.536 (0.061-1.014) | 182 (108 - 275) | 25.614 (15.197 - 39.159) | 899 (655 - 1192) | 16.294 (12.409 - 20.864) | -0.377 (-0.761-0.008) |
| United Kingdom | 1127 (1098 - 1151) | 1.384 (1.349 - 1.414) | 3544 (3390 - 3673) | 3.195 (3.067 - 3.306) | 3.233 (2.977-3.49) | 552 (535 - 564) | 0.645 (0.627 - 0.659) | 1216 (1148 - 1267) | 1.011 (0.961 - 1.05) | 1.757 (1.44-2.075) | 14050 (13711 - 14374) | 17.659 (17.237 - 18.07) | 30940 (29559 - 32153) | 28.288 (27.112 - 29.373) | 1.836 (1.548-2.124) |
| United Republic of Tanzania | 99 (64 - 148) | 0.826 (0.54 - 1.222) | 221 (132 - 351) | 0.762 (0.464 - 1.208) | -0.413 (-0.464to-0.362) | 91 (59 - 137) | 0.774 (0.507 - 1.164) | 191 (115 - 307) | 0.68 (0.422 - 1.087) | -0.543 (-0.591to-0.495) | 2876 (1830 - 4439) | 22.982 (14.738 - 35.072) | 6188 (3631 - 9995) | 19.986 (11.961 - 32.323) | -0.584 (-0.638to-0.531) |
| United States of America | 7379 (7140 - 7556) | 2.528 (2.456 - 2.587) | 16196 (15447 - 16863) | 2.97 (2.842 - 3.086) | 0.697 (0.538-0.855) | 2022 (1934 - 2073) | 0.665 (0.639 - 0.682) | 3539 (3346 - 3700) | 0.617 (0.586 - 0.645) | -0.089 (-0.355-0.178) | 55953 (54253 - 57603) | 19.337 (18.741 - 19.895) | 93305 (89355 - 97601) | 17.3 (16.612 - 18.073) | -0.175 (-0.421-0.071) |
| United States Virgin Islands | 2 (1 - 2) | 1.751 (1.374 - 2.148) | 2 (1 - 2) | 1.098 (0.813 - 1.484) | -1.444 (-1.615to-1.274) | 1 (1 - 1) | 1.437 (1.131 - 1.765) | 1 (1 - 2) | 0.773 (0.584 - 1.028) | -1.894 (-2.056to-1.732) | 34 (27 - 42) | 36.59 (29.005 - 44.889) | 32 (24 - 43) | 20.623 (15.228 - 28.055) | -1.68 (-1.841to-1.518) |
| Uruguay | 85 (73 - 98) | 2.265 (1.935 - 2.617) | 73 (60 - 88) | 1.477 (1.216 - 1.775) | -1.34 (-1.581to-1.098) | 63 (53 - 72) | 1.645 (1.406 - 1.895) | 45 (37 - 54) | 0.865 (0.717 - 1.038) | -1.984 (-2.241to-1.725) | 1731 (1471 - 1996) | 46.946 (39.982 - 53.991) | 1144 (946 - 1373) | 23.825 (19.699 - 28.632) | -2.122 (-2.373to-1.87) |
| Uzbekistan | 93 (68 - 126) | 0.766 (0.567 - 1.06) | 216 (162 - 277) | 0.741 (0.564 - 0.926) | 0.157 (-0.14-0.455) | 78 (57 - 109) | 0.66 (0.484 - 0.953) | 166 (124 - 212) | 0.599 (0.459 - 0.746) | -0.066 (-0.402-0.272) | 2426 (1780 - 3200) | 19.217 (14.184 - 25.645) | 5236 (3833 - 6845) | 16.97 (12.7 - 21.747) | -0.194 (-0.51-0.124) |
| Vanuatu | 0 (0 - 0) | 0.145 (0.105 - 0.203) | 0 (0 - 0) | 0.137 (0.105 - 0.184) | -0.254 (-0.354to-0.155) | 0 (0 - 0) | 0.139 (0.102 - 0.193) | 0 (0 - 0) | 0.128 (0.099 - 0.17) | -0.308 (-0.392to-0.223) | 3 (2 - 5) | 3.935 (2.744 - 5.738) | 8 (6 - 11) | 3.731 (2.802 - 5.06) | -0.254 (-0.356to-0.151) |
| Venezuela  (Bolivarian Republic of) | 74 (69 - 79) | 0.757 (0.707 - 0.806) | 209 (151 - 282) | 0.683 (0.497 - 0.918) | -0.301 (-0.53to-0.071) | 65 (61 - 69) | 0.683 (0.637 - 0.725) | 159 (115 - 213) | 0.529 (0.386 - 0.705) | -0.828 (-1.072to-0.583) | 1791 (1680 - 1907) | 17.316 (16.222 - 18.379) | 4201 (3006 - 5675) | 13.548 (9.724 - 18.265) | -0.79 (-1.042to-0.538) |
| Viet Nam | 787 (572 - 1048) | 1.897 (1.385 - 2.518) | 3106 (2270 - 4056) | 2.865 (2.11 - 3.692) | 1.417 (1.289-1.545) | 684 (498 - 909) | 1.666 (1.22 - 2.195) | 2090 (1539 - 2725) | 1.988 (1.468 - 2.549) | 0.637 (0.554-0.72) | 20380 (14779 - 27409) | 48.408 (35.216 - 64.938) | 63135 (45728 - 83645) | 56.736 (41.488 - 74.621) | 0.591 (0.489-0.694) |
| Yemen | 15 (9 - 22) | 0.29 (0.189 - 0.429) | 38 (24 - 55) | 0.253 (0.165 - 0.374) | -0.421 (-0.478to-0.363) | 13 (9 - 20) | 0.278 (0.184 - 0.409) | 31 (21 - 46) | 0.227 (0.149 - 0.34) | -0.681 (-0.732to-0.63) | 405 (253 - 618) | 7.301 (4.642 - 11.013) | 940 (605 - 1372) | 5.727 (3.758 - 8.461) | -0.811 (-0.865to-0.758) |
| Zambia | 27 (19 - 36) | 0.839 (0.607 - 1.11) | 117 (50 - 262) | 1.358 (0.612 - 2.998) | 1.781 (1.536-2.028) | 25 (18 - 33) | 0.803 (0.581 - 1.061) | 99 (43 - 227) | 1.206 (0.548 - 2.683) | 1.532 (1.325-1.74) | 817 (588 - 1095) | 23.786 (17.346 - 31.896) | 3476 (1442 - 8025) | 37.228 (16.117 - 85.184) | 1.693 (1.465-1.921) |
| Zimbabwe | 17 (14 - 22) | 0.39 (0.31 - 0.488) | 44 (31 - 60) | 0.539 (0.382 - 0.722) | 1.025 (0.575-1.477) | 15 (12 - 19) | 0.355 (0.279 - 0.442) | 39 (27 - 52) | 0.491 (0.348 - 0.648) | 1.131 (0.698-1.566) | 469 (367 - 599) | 10.141 (7.942 - 12.809) | 1308 (908 - 1804) | 14.951 (10.453 - 20.284) | 1.343 (0.857-1.832) |
