# Supplementary material for: Incidence, mortality, and DALYs of global pharyngeal cancer: systematic analysis and projections Based on global burden of disease study 2021
Source: Ann Med. 2025 Aug 19;57(1):2547092. doi: 10.1080/07853890.2025.2547092 (PMC12366512; doi:10.1080/07853890.2025.2547092)
Supplement: Supplementary Table 3.docx [file IANN_A_2547092_SM9502.docx]

| **Supplementary Table 3. Comparative global burden of pharyngeal cancer across pre-COVID-19 (2019) and post-COVID-19 (2021) periods in GBD 2021** | | | | | |
| --- | --- | --- | --- | --- | --- |
| Measure | Metric | 1990 | 2019* | 2021** | COVID-19 change(%) |
| Incidence | cases | 64529 (60829 - 68920) | 164154 (156477 - 172781) | 169820 (159847 - 179704) | 3.45 |
|  | ASR | 1.552 (1.463 - 1.657) | 1.955 (1.864 - 2.057) | 1.933 (1.818 - 2.047) | -1.13 |
|  | EAPC | — | 0.665 (0.581-0.748) | 0.677 (0.603-0.751) | — |
| Deaths | cases | 44344 (40994 - 48190) | 95286 (90000 - 101055) | 98435 (91567 - 105485) | 3.30 |
|  | ASR | 1.082 (1.001 - 1.176) | 1.142 (1.078 - 1.211) | 1.127 (1.048 - 1.207) | -1.31 |
|  | EAPC | — | 0.028 (-0.057-0.114) | 0.061 (-0.018-0.141) | — |
| DALYs | cases | 1372478 (1270671 - 1494393) | 2758289 (2591479 - 2942783) | 2843781 (2622259 - 3063043) | 3.10 |
|  | ASR | 32.302 (29.919 - 35.17) | 32.738 (30.755 - 34.926) | 32.377 (29.854 - 34.87) | -1.1 |
|  | EAPC | — | -0.124 (-0.207to-0.042) | -0.087 (-0.165to-0.009) | — |
| ASR: age-standardized rate; EAPC:estimated annual percentage change; *: EAPC from 1990 to 2019; **: EAPC from 1990 to 2021 | | | | | |
