# Supplementary material for: Incidence, mortality, and DALYs of global pharyngeal cancer: systematic analysis and projections Based on global burden of disease study 2021
Source: Ann Med. 2025 Aug 19;57(1):2547092. doi: 10.1080/07853890.2025.2547092 (PMC12366512; doi:10.1080/07853890.2025.2547092)
Supplement: Supplementary Table 4.docx [file IANN_A_2547092_SM9501.docx]

| **Supplementary Table 4.Projection of global pharyngeal cancer burden using BAPC and Nordpred methods (2022–2050)** | | | | | | |
| --- | --- | --- | --- | --- | --- | --- |
| **Year** | **ASIR** | | **ASDR** | | **Age-standardized DALYs rate** | |
|  | **BAPC** | **Nordpred** | **BAPC** | **Nordpred** | **BAPC** | **Nordpred** |
| 1990 | 1.566 | 1.609 | 1.095 | 1.104 | 32.636 | 32.919 |
| 1991 | 1.584 | 1.616 | 1.101 | 1.102 | 32.786 | 32.827 |
| 1992 | 1.605 | 1.624 | 1.109 | 1.100 | 33.007 | 32.734 |
| 1993 | 1.630 | 1.632 | 1.118 | 1.098 | 33.261 | 32.640 |
| 1994 | 1.651 | 1.639 | 1.123 | 1.096 | 33.352 | 32.536 |
| 1995 | 1.660 | 1.644 | 1.120 | 1.093 | 33.152 | 32.413 |
| 1996 | 1.663 | 1.648 | 1.113 | 1.089 | 32.890 | 32.261 |
| 1997 | 1.664 | 1.650 | 1.107 | 1.084 | 32.640 | 32.071 |
| 1998 | 1.667 | 1.649 | 1.103 | 1.078 | 32.515 | 31.839 |
| 1999 | 1.663 | 1.647 | 1.096 | 1.071 | 32.299 | 31.587 |
| 2000 | 1.654 | 1.646 | 1.089 | 1.064 | 32.030 | 31.342 |
| 2001 | 1.649 | 1.647 | 1.081 | 1.058 | 31.739 | 31.130 |
| 2002 | 1.646 | 1.653 | 1.074 | 1.055 | 31.520 | 30.980 |
| 2003 | 1.646 | 1.665 | 1.069 | 1.054 | 31.282 | 30.910 |
| 2004 | 1.645 | 1.681 | 1.059 | 1.056 | 30.881 | 30.904 |
| 2005 | 1.668 | 1.700 | 1.059 | 1.058 | 30.883 | 30.937 |
| 2006 | 1.673 | 1.720 | 1.051 | 1.062 | 30.572 | 30.985 |
| 2007 | 1.697 | 1.738 | 1.058 | 1.065 | 30.732 | 31.023 |
| 2008 | 1.720 | 1.754 | 1.066 | 1.068 | 30.944 | 31.034 |
| 2009 | 1.734 | 1.767 | 1.069 | 1.070 | 30.934 | 31.033 |
| 2010 | 1.771 | 1.781 | 1.085 | 1.072 | 31.431 | 31.039 |
| 2011 | 1.778 | 1.795 | 1.085 | 1.076 | 31.325 | 31.075 |
| 2012 | 1.766 | 1.811 | 1.076 | 1.081 | 30.941 | 31.162 |
| 2013 | 1.776 | 1.830 | 1.075 | 1.088 | 30.849 | 31.315 |
| 2014 | 1.811 | 1.853 | 1.086 | 1.097 | 31.086 | 31.523 |
| 2015 | 1.848 | 1.877 | 1.101 | 1.106 | 31.482 | 31.768 |
| 2016 | 1.881 | 1.902 | 1.114 | 1.117 | 31.817 | 32.034 |
| 2017 | 1.916 | 1.927 | 1.130 | 1.127 | 32.252 | 32.302 |
| 2018 | 1.945 | 1.952 | 1.143 | 1.137 | 32.588 | 32.559 |
| 2019 | 1.953 | 1.976 | 1.146 | 1.146 | 32.649 | 32.800 |
| 2020 | 1.934 | 1.999 | 1.138 | 1.155 | 32.389 | 33.027 |
| 2021 | 1.935 | 2.021 | 1.134 | 1.163 | 32.318 | 33.238 |
| 2022 | 1.912 | 2.041 | 1.123 | 1.170 | 31.875 | 33.433 |
| 2023 | 1.906 | 2.059 | 1.118 | 1.176 | 31.784 | 33.612 |
| 2024 | 1.900 | 2.076 | 1.114 | 1.182 | 31.701 | 33.776 |
| 2025 | 1.895 | 2.091 | 1.110 | 1.187 | 31.626 | 33.927 |
| 2026 | 1.890 | 2.104 | 1.106 | 1.191 | 31.569 | 34.064 |
| 2027 | 1.886 | 2.116 | 1.103 | 1.195 | 31.531 | 34.191 |
| 2028 | 1.882 | 2.127 | 1.100 | 1.198 | 31.507 | 34.307 |
| 2029 | 1.879 | 2.136 | 1.097 | 1.200 | 31.490 | 34.411 |
| 2030 | 1.877 | 2.145 | 1.094 | 1.202 | 31.482 | 34.500 |
| 2031 | 1.876 | 2.151 | 1.092 | 1.203 | 31.487 | 34.574 |
| 2032 | 1.876 | 2.156 | 1.091 | 1.204 | 31.510 | 34.629 |
| 2033 | 1.876 | 2.159 | 1.089 | 1.204 | 31.542 | 34.665 |
| 2034 | 1.876 | 2.161 | 1.088 | 1.203 | 31.576 | 34.688 |
| 2035 | 1.877 | 2.162 | 1.087 | 1.203 | 31.615 | 34.704 |
| 2036 | 1.880 | 2.164 | 1.087 | 1.202 | 31.665 | 34.721 |
| 2037 | 1.883 | 2.166 | 1.087 | 1.202 | 31.728 | 34.744 |
| 2038 | 1.886 | 2.169 | 1.088 | 1.202 | 31.793 | 34.780 |
| 2039 | 1.890 | 2.174 | 1.088 | 1.203 | 31.857 | 34.827 |
| 2040 | 1.895 | 2.179 | 1.089 | 1.204 | 31.925 | 34.882 |
| 2041 | 1.900 | 2.185 | 1.090 | 1.205 | 32.000 | 34.943 |
| 2042 | 1.906 | 2.191 | 1.092 | 1.206 | 32.084 | 35.007 |
| 2043 | 1.913 | 2.197 | 1.093 | 1.207 | 32.166 | 35.071 |
| 2044 | 1.919 | 2.203 | 1.095 | 1.208 | 32.246 | 35.135 |
| 2045 | 1.925 | 2.209 | 1.097 | 1.210 | 32.328 | 35.200 |
| 2046 | 1.933 | 2.216 | 1.099 | 1.211 | 32.418 | 35.264 |
| 2047 | 1.941 | 2.222 | 1.102 | 1.212 | 32.514 | 35.328 |
| 2048 | 1.949 | 2.228 | 1.104 | 1.213 | 32.610 | 35.392 |
| 2049 | 1.957 | 2.234 | 1.107 | 1.215 | 32.703 | 35.457 |
| 2050 | 1.965 | 2.240 | 1.109 | 1.216 | 32.800 | 35.521 |
