# Supplementary material for: Incidence, mortality, and DALYs of global pharyngeal cancer: systematic analysis and projections Based on global burden of disease study 2021
Source: Ann Med. 2025 Aug 19;57(1):2547092. doi: 10.1080/07853890.2025.2547092 (PMC12366512; doi:10.1080/07853890.2025.2547092)
Supplement: Supplementary Table 5.docx [file IANN_A_2547092_SM9500.docx]

| **Supplementary Table 5. Risk factors associated with mortality of pharyngeal cancer by SDI regions and gender** | | | | |
| --- | --- | --- | --- | --- |
| **Location** | **Smoking (%)** | | **Alcohol use (%)** | |
|  | **Male** | **Female** | **Male** | **Female** |
| Global | **41.93 (34.19-48.99)** | 12.79 (9.5-16.14) | **26.48 (19.38-33.1)** | 6.98 (4.77-9.28) |
| Low SDI | 31.42 (24.81-37.75) | 9.33 (6.74-12.43) | 17.37 (10.66-23.63) | 3.4 (2.02-4.86) |
| Low-middle SDI | 42.66 (35.26-49.38) | 8.38 (6.17-11.06) | 17.27 (10.89-23.28) | 1.73 (0.97-2.58) |
| Middle SDI | 37.17 (30.06-44.01) | 8.28 (6.1-10.89) | 25.8 (18.51-32.45) | 2.96 (1.92-3.97) |
| High-middle SDI | **51.73 (43.28-59.38)** | 16.23 (12.27-20.22) | **37.56 (28.49-45.69)** | 11.6 (7.88-15.55) |
| High SDI | 42.92 (33.99-51.58) | **26.92 (20.5-33.76)** | 37.37 (28.29-45.3) | **21.48 (15.71-27.38)** |
